# Nontraditional Synthesis of Disaccharides via Acyclic Vinylic Ether Intermediates: Catalytic C–O Cross-Coupling as the Enabling Link

**DOI:** 10.1021/acs.joc.4c02176

**Published:** 2024-11-27

**Authors:** Taehee Kim, Eric J. Meindl, Frank E. McDonald

**Affiliations:** Department of Chemistry, Emory University, 1515 Dickey Drive NE, Atlanta, Georgia 30322, United States

## Abstract

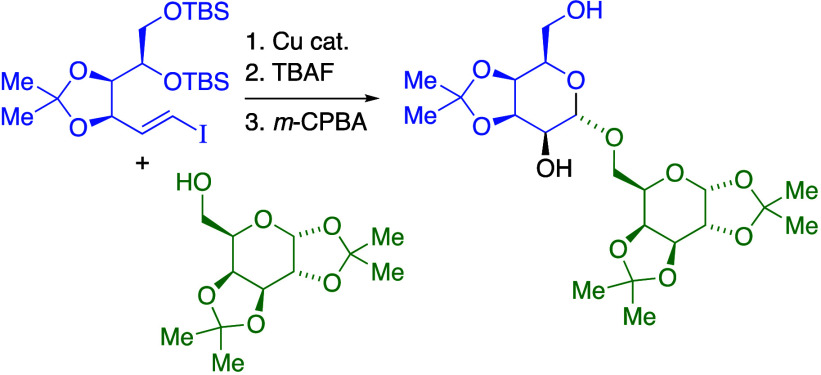

We describe complementary methods for synthesizing acyclic vinylic ethers from two carbohydrate-derived synthons. We compare a nonstereoselective olefination approach with a stereoselective catalytic C–O cross-coupling method, preparing 1,2-disubstituted vinylic ethers with complexity on both sides of the ether linkage. Upon epoxidation/*in situ* oxacyclization of acyclic vinylic ethers, we synthesized disaccharides with α-d-galacto-, α-d-talo-, β-d-allo-, and α-d-altropyranoside stereochemistry, from d-lyxose and d-ribose precursors. Stereoselective CuI/CyDMEDA-catalyzed C–O cross-couplings offer considerable potential for broadly implementing this nontraditional strategy for glycoside synthesis.

Glycoside synthesis traditionally involves carbon–oxygen bond formation by an intermolecular substitution mechanism linking a nucleophilic alcohol (glycosyl acceptor) with a carbohydrate electrophile (glycosyl donor). Glycoside stereoselectivity primarily arises from glycosyl donor stereochemistry, protective group effects, and reactants and conditions selected for electrophilic activation.^1^ Among several notable nontraditional glycoside synthesis strategies,^2^ Suzuki and Mukaiyama have pioneered an approach featuring *electrophile-promoted oxacyclizations of acyclic vinylic ether intermediates* (Scheme 1).^3−6^ Conceptually, the stereochemistry-defining C–O bond-forming step of glycoside synthesis becomes an *intramolecular reaction*, with stereoinduction arising from substituents on a conformationally flexible acyclic tether.^7^ However, this strategy is limited by the lack of stereoselective methods for preparing structurally complex (*E*)- or (*Z*)-1,2-disubstituted vinylic ethers. The few disaccharide syntheses reported by this nontraditional approach have prepared vinylic ether intermediates from olefination of alcohol-protected aldehyde or hemiacetal **1** with carbohydrate-derived phosphonate or phosphine oxide including **2**, giving mixtures of (*E*)- and (*Z*)-vinylic ethers **3** and **4**.^3−6^ In specific examples, *m*-CPBA epoxidation of isolated (*E*)-vinylic ether **3** followed by *in situ* oxacyclization exhibits good stereoselectivity, producing primarily disaccharide **5** (α-d-manno-:α-d-gluco, 85:15 dr). In contrast, epoxidation–cyclization from the major (*Z*)-vinylic ether **4** gives three disaccharide diastereomers.^6^

**Scheme 1:**
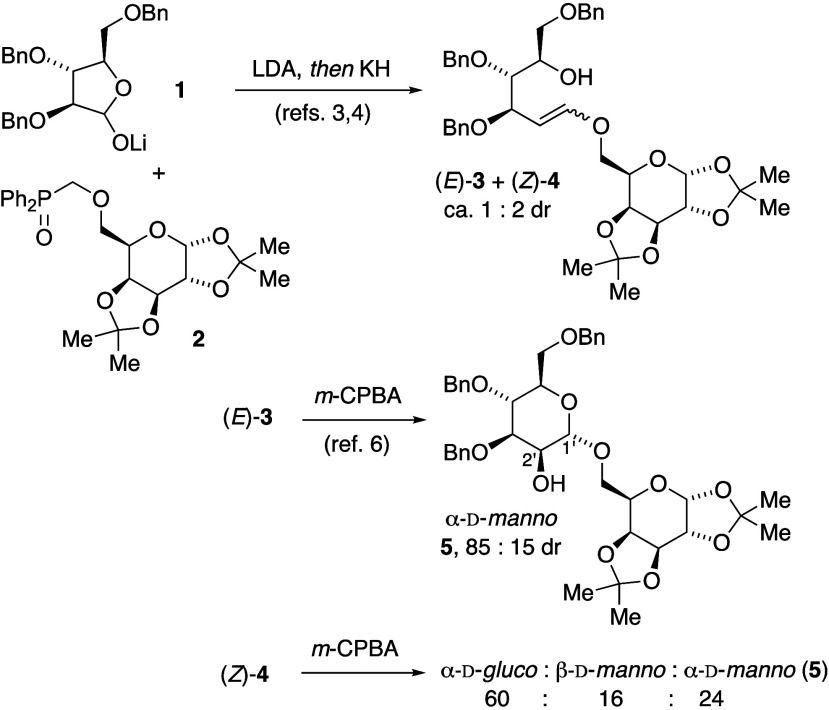
Nontraditional Disaccharide Synthesis from Oxacyclization of Vinylic Ether Intermediates

The poor stereoselectivity for vinylic ether synthesis has probably discouraged subsequent explorations of this nontraditional glycoside synthesis strategy. Therefore, we have pursued other approaches toward the (*E*)-selective synthesis of vinylic ethers corresponding to aldohexose precursors. Modified Julia olefinations of sulfone-stabilized anions offered potential for high (*E*)-stereoselectivity by varying reaction conditions,^8,9^ including a few examples applied to vinylic ether synthesis.^10^ Model experiments (see Supporting Information) indicated that benzothiazolylsulfones, including galactose-derived alkoxysulfone **7**, were viable nucleophilic synthons for the olefination step. We synthesized alkoxysulfone **7** in four steps from d-galactose bis-acetonide **6** (Scheme 2a). However, the final oxidation step from sulfide to produce sulfone **7** was unexpectedly challenging. Only sodium tungstate-catalyzed hydrogen peroxide oxidation succeeded, and required careful temperature control to avoid protonolysis of the sulfur–carbon bond to the heterocycle.^11^

**Scheme 2:**
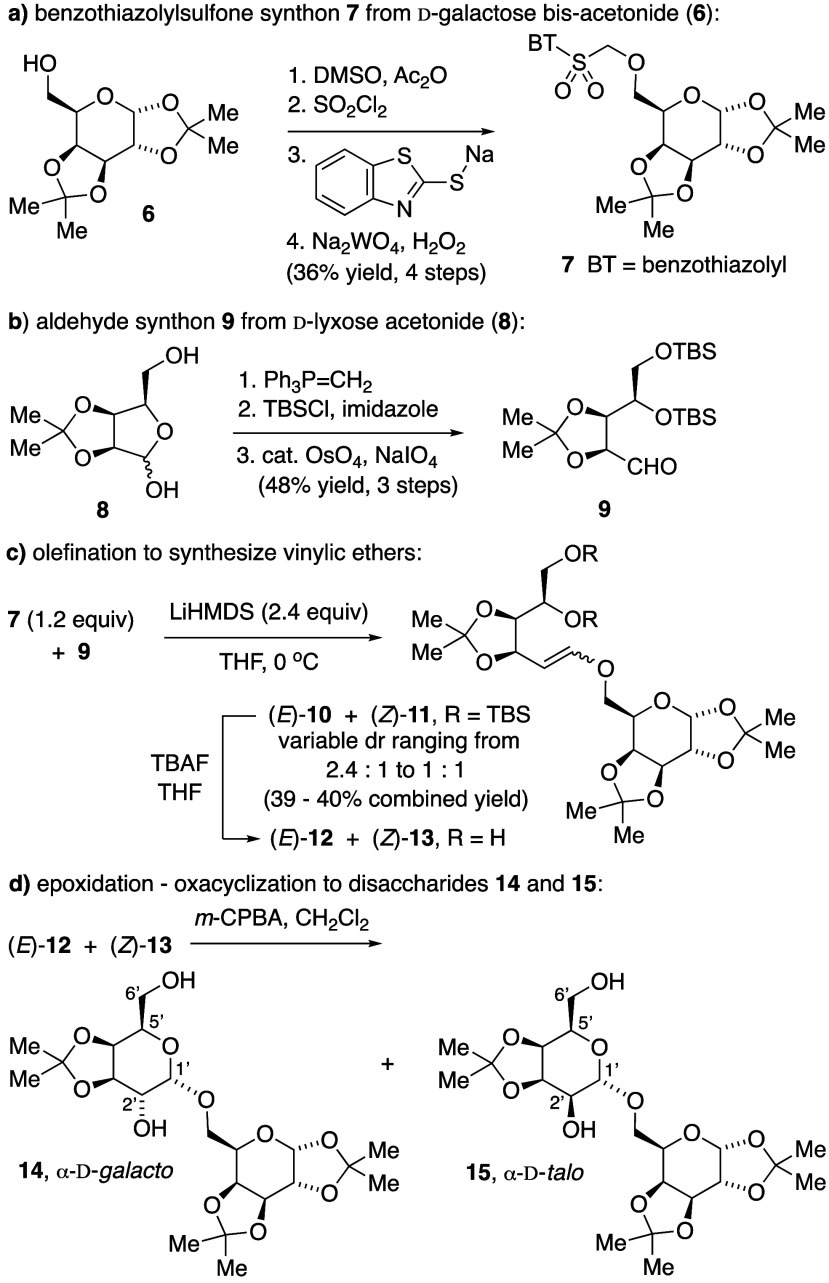
Modified Julia Olefination with Carbohydrate-Derived Synthons 7 and 9

Preliminary experiments revealed that cyclic hemiacetal reactants were ineffective electrophilic synthons for Julia olefination. Therefore, we adopted a protective group strategy that masked all alcohols as acetonide and silyl ethers, as the d-lyxose-derived aldehyde **9** (Scheme 2b).^12^ Although both KHMDS and LiHMDS promoted Julia olefination in model experiments with aldehyde **9**, only LiHMDS reproducibly gave vinylic ethers from galactose-derived sulfone **7** and aldehyde **9**, producing a mixture of (*E*)-**10** and (*Z*)-**11** (Scheme 2c). Benzothiazole-containing byproducts were difficult to separate from the vinylic ether products, and the isomers (*E*)-**10** and (*Z*)-**11** were nearly inseparable. Under seemingly identical conditions, *E*/*Z*-isomer ratios in crude product mixtures ranged from 2.4:1 to as low as 1:1.

After desilylation of an (*E*)-**10**/(*Z*)-**11** mixture to provide diol-vinylic ethers (*E*)-**12** and (*Z*)-**13**, also inseparable, *m*-CPBA epoxidation produced a mixture of disaccharide diastereomers, favoring α-galactopyranoside **14** and α-talopyranoside **15** (Scheme 2c). A trace of a third isomer coeluted with **15** (4.4:1 ratio) but was inseparable and thus not fully characterized. We determined stereochemical assignments for **14** and **15** primarily by comparing coupling constants for our synthetic disaccharides with data published for the corresponding methyl pyranosides **16** and **17** (Figure 1).^13,14^

**Figure 1:**
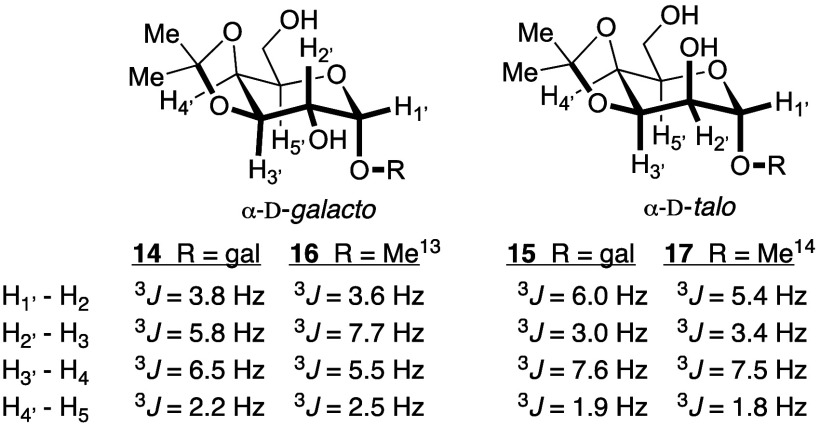
Stereochemical assignments from coupling constants for disaccharides **14** and **15**, compared with coupling constants for methyl glycosides **16** and **17**, respectively.

Even though we synthesized disaccharides **14** and **15** by the nontraditional strategy, the modified Julia olefination to generate the vinylic ether synthetic intermediate was not stereoselective. In addition, preparing alkoxysulfone **7** from carbohydrate alcohol **6** was challenging. Therefore, we pursued approaches in which a hydroxy group such as the alcohol in compound **6** was directly converted into vinylic ether. We initially considered intermolecular hydroalkoxylation of terminal alkynes with alcohols.^15^ However, a literature search of known rhodium catalysts, and model studies with ruthenium catalysts^16^ revealed that alkyne hydroalkoxylation was insufficiently developed for structurally complex substrates such as alcohol **6**, or with lyxose-derived alkyne **18** (Scheme 3). As an alternative to alkyne hydroalkoxylation, we attempted to apply literature methods for C–O cross-coupling to prepare vinylic ethers,^17^ but initially with little success. After we established that CuI with *trans*-*N,N′*-dimethylcyclohexanediamine (CyDMEDA) ligand effectively catalyzed stereospecific C–O cross-couplings, with several vinylic halides and a broad range of 1° and 2° alcohols,^18^ we tested this modified C–O cross-coupling method in the context of the nontraditional disaccharide synthetic strategy.

**Scheme 3:**
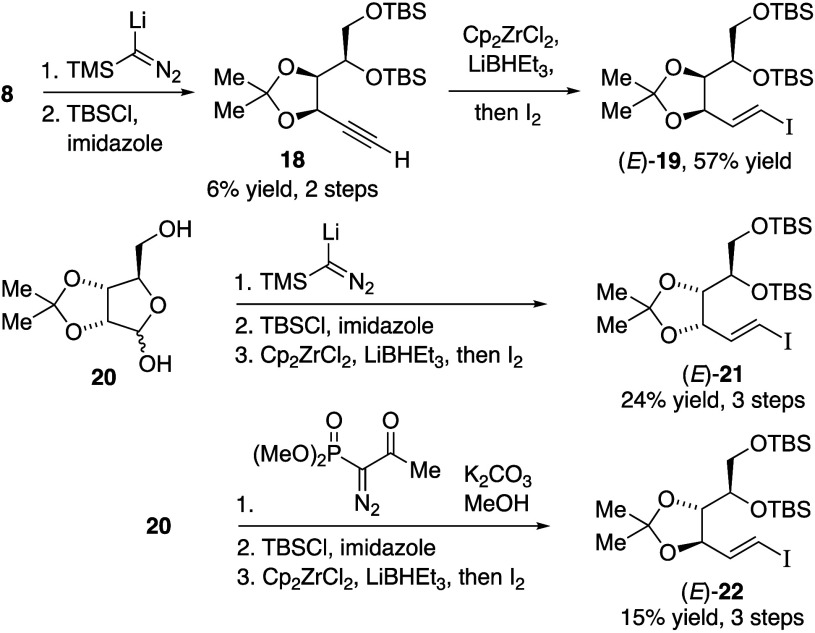
Synthesis of (*E*)-Vinylic Iodides from d-Lyxose and d-Ribose Acetonides

We prepared alkyne **18** from d-lyxose acetonide (**8**), using lithium trimethylsilyldiazomethane,^19^ followed by silyl ether protection of the diol (Scheme 3). Conversion was low, attributed to unfavorable ring-opening of cyclic hemiacetal to the open-chain aldehyde; however, we obtained terminal alkyne **18** without epimerization of the propargylic stereocenter. Hydrozirconation/iodination^20^ of **18** produced vinylic iodide (*E*)-**19** as a single stereoisomer. A similar approach from d-ribose acetonide (**20**) provided vinylic iodide (*E*)-**21**. However, Bestmann–Ohira alkynylation of **20** afforded an alkyne diastereomer from epimerization,^21,22^ which we also converted into vinylic iodide (*E*)-**22**.

Catalytic C–O cross-coupling of (*E*)-vinylic iodide **19** with 2 equiv of galactose bis-acetonide (**6**) stereoselectively gave the vinylic ether (*E*)-**10** (Scheme 4). Spectroscopic characteristics matched the (*E*)-vinylic ether component of the isomeric mixture of (*E*)-**10** + (*Z*)-**11** arising from nonstereoselective Julia olefination (Scheme 2c). Under identical cross-coupling conditions, we prepared vinylic ethers (*E*)-**23** and (*E*)-**24** from the respective vinylic iodides (*E*)-**21** and (*E*)-**22**. Product mixtures from C–O cross-coupling with vinylic iodide (*E*)-**21** showed an enyne in approximately 18% yield,^18^ which was chromatographically separable from the vinylic ether product (*E*)-**23**. In no case did we observe evidence of (*Z*)-isomeric products. Another significant advantage of C–O cross-coupling over olefination methods was directly using commercially available carbohydrate alcohol **6**, without requiring multistep syntheses of phosphine oxide **2** or sulfone **7**.

**Scheme 4:**
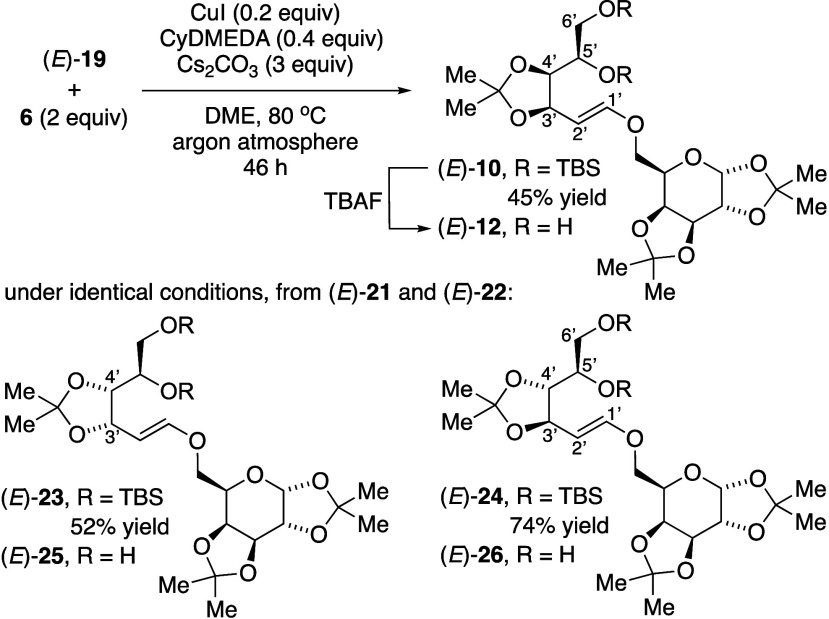
Synthesis of (*E*)-Vinylic Ethers from Catalytic C–O Cross-Coupling with Galactose Bis-Acetonide (6)

With an isomerically pure sample of diol-vinylic ether (*E*)-**12**, *m*-CPBA epoxidation with *in situ* oxacyclization produced a 5.7:1 mixture of disaccharide diastereomers, favoring α-talopyranoside **15** (Scheme 5). The major disaccharide **15** from pure (*E*)-**12** spectroscopically matched one of the major disaccharide products previously generated from the inseparable mixture of (*E*)-**12** and (*Z*)-**13**.^23^*O*-Acetylation of both alcohols, **15** → **27**, confirmed the 6′-primary alcohol and 2′-secondary alcohol, demonstrating that *in situ* oxacyclization of an epoxide reactive intermediate favored the pyranoside over other ring sizes, from regioselective oxacyclization of C5′-alcohol onto C1′. The stereochemical outcome is fully consistent with stereospecific epoxidation of the (*E*)-vinylic ether **12** followed by stereospecific anti-opening of an epoxide reactive intermediate.

**Scheme 5:**
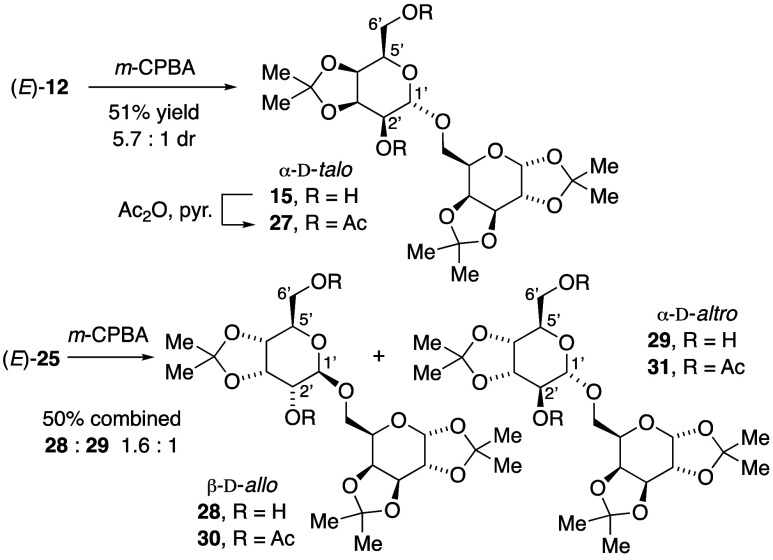
Epoxidations–Oxacyclizations of (*E*)-12 and (*E*)-25 to Novel Disaccharides

*m*-CPBA epoxidation–oxacyclization of the diol-vinylic ether (*E*)-**25** was less diastereoselective, giving a 1.6:1 mixture of β-allopyranoside **28** and α-altropyranoside **29**. Epoxidation with trifluoroperoxyacetic acid^24^ gave even lower diastereoselectivity (1.2:1 ratio). Vanadium-catalyzed^25^ hydroxy-directed epoxidation of (*E*)-**25** exhibited greater preference for diastereomer **28**, albeit with incomplete conversion. We assigned relative stereochemistry in diacetate derivative **30**, with larger H1′–H2′ coupling constant (*J* > 7 Hz) for diaxial vicinal pairs. The smaller H1′–H2′ coupling constant in the minor diastereomer **31** was consistent with a boat-like conformer (Figure 2). These results suggested that for *m*-CPBA epoxidation of diol-vinylic ether (*E*)-**25**, stereoinduction effects from the adjacent acetonide stereochemistry and possible hydroxy-directing effects from the C5′ alcohol were *mismatched*.

**Figure 2:**
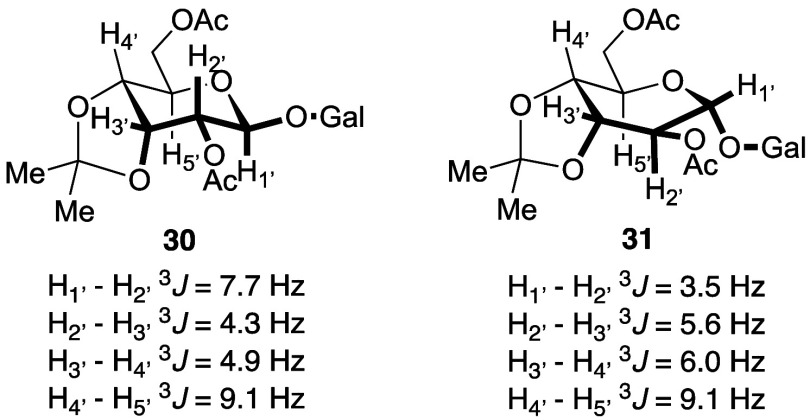
Stereochemical assignments for disaccharide diacetates **30** and **31**.

*m*-CPBA epoxidation of diol-vinylic ether (*E*)-**26** gave a mixture of compounds, which we could not definitively assign as disaccharide products. Although electrophilic oxacyclizations had proceeded smoothly for epoxide intermediates arising from *cis*-substituted acetonide substrates (*E*)-**12** and (*E*)-**25**, it appeared that *trans*-disubstituted acetonide disfavored oxacyclization of the diol-epoxide intermediate, revealing a significant drawback of the O3′–O4′ acetonide protective group.

Nonetheless, we have established that catalytic C–O cross-coupling effectively enables stereoselective syntheses of vinylic ethers *with structural and stereochemical complexity on both sides of the ether linkage*, removing a significant tactical barrier to applying the nontraditional glycoside synthetic strategy pioneered by Suzuki and Mukaiyama more than four decades ago.^3,4^ Our next research phase will apply this approach to synthesize oligosaccharides of deoxy- and amine-substituted carbohydrates.^26^ In forthcoming research, we will explore *de novo* preparations of alkyne and vinylic halide precursors with better yields,^27^ and will redesign the vicinal diol protective group strategy to circumvent restrictions encountered with *trans*-disubstituted acetonides.^28,29^

## Data Availability

The data underlying this study are available in the published article and its online Supporting Information.

## Supporting Information Available

Detailed experimental procedures, including preparations and characterizations (PDF)
Copies of ^1^H and ^13^C NMR spectra for compounds **7**, **9**, **10**, **12**, **14**, **15**, **18**, **19**, **21**–**25**, and **27**–**31** (PDF)
FAIR data, including the primary NMR FID files, for compounds **7**, **9**, **10**, **12**, **14**, **15**, **18**, **19**, **21**–**25**, and **27**–**31** (ZIP)
