# Supplementary material for: Nontraditional Synthesis of Disaccharides via Acyclic Vinylic Ether Intermediates: Catalytic C–O Cross-Coupling as the Enabling Link
Source: J Org Chem. 2024 Nov 27;89(24):18684–8. doi: 10.1021/acs.joc.4c02176 (PMC11667969; doi:10.1021/acs.joc.4c02176)
Supplement: Supplementary file 1 — jo4c02176_si_001.pdf [file jo4c02176_si_001.pdf]

Supporting Information

Non-traditional synthesis of disaccharides via acyclic vinylic ether intermediates: Catalytic C-O cross-coupling as the enabling link

Taehee Kim, Eric J. Meindl and Frank E. McDonald*
Department of Chemistry, Emory University, 1515 Dickey Drive NE, Atlanta GA 30322 USA
*Corresponding author: fmcdona@emory.edu

Part 1: Detailed experimental procedures, compound characterization

Table of contents

General Experimental	S-2
Synthesis of benzothiazole-sulfone **7** from D-galactose bis-acetonide (**6**)	S-3
Synthesis of aldehyde **9** from D-lyxose acetonide (**8**)	S-6
Model experiments for sulfone preparation, and for modified Julia olefination	S-8
Modified Julia olefination to synthesize vinylic ether mixtures (*E*)-**10** and (*Z*)-**11**	S-9
Deprotection of silyl ethers to give mixture of diol-vinylic ethers (*E*)-**12** and (*Z*)-**13**	S-10
Epoxidation-oxacyclization of (*E*)-**12** and (*Z*)-**13** mixture to disaccharides **14** and **15**	S-11
Vinylic iodide (*E*)-**19** from D-lyxose acetonide (**8**)	S-14
Vinylic iodide (*E*)-**21** from D-ribose acetonide (**20**)	S-16
Vinylic iodide (*E*)-**22** from D-ribose acetonide (**20**)	S-18
C-O cross-coupling to synthesize vinylic ether (*E*)-**10**	S-21
C-O cross-coupling to synthesize vinylic ether (*E*)-**23**	S-23
C-O cross-coupling to synthesize vinylic ether (*E*)-**24**	S-25
Deprotection of silyl ethers from (*E*)-**10** to diol-vinylic ether (*E*)-**12**	S-27
Deprotection of silyl ethers from (*E*)-**23** to diol-vinylic ether (*E*)-**25**	S-28
Deprotection of silyl ethers from (*E*)-**24** to diol-vinylic ether (*E*)-**26**	S-29
Epoxidation-oxacyclization of (*E*)-**12** to disaccharide **15** and diacetate derivative **27**	S-30
Epoxidation-oxacyclization of (*E*)-**25** to disaccharides **28** and **29**	S-34
References	S-39

General Experimental

^1H and ^{13}C NMR spectra were recorded with Varian AVIII 400, Bruker NEO 400, AVANCE 600 equipped with a cryogen probe, and ASCEND 800 spectrometers. Some structural assignments were made with additional information from gCOSY, gHSQC, and gHMBC experiments. NMR spectra were measured from solutions in deuterated chloroform ($CDCl_3$) (neutralized with anhydrous K_2CO_3 and kept dry with anhydrous $MgSO_4$), and deuterated benzene (C_6D_6) for potentially acid-sensitive compounds. The residual chloroform resonances (7.26 ppm for ^1H NMR and 77.23 ppm for ^{13}C NMR) and the residual benzene resonances (7.16 ppm for ^1H NMR and 128.06 ppm for ^{13}C NMR) were used as internal standards, and were reported in parts per million (ppm). Abbreviations for NMR signal coupling are as follows: s, singlet; d, doublet; t, triplet; q, quartet; dd, doublet of doublet; ddd, doublet of doublet of doublet; dt, doublet of triplet; m, multiplet; br, broad.

Mass spectra, variously conducted by high resolution ESI (electrospray ionization), NSI (nanospray desorption electrospray ionization) and atmospheric pressure chemical ionization (APCI) were recorded on a Scientific Exactive Plus Mass spectrometer, using the orbitrap mass analyzer, which is an electrostatic ion trap. Optical rotations were measured using a Perkin-Elmer 341 polarimeter (concentration in g/100mL). Thin layer chromatography (TLC) was performed on precoated glass backed plates purchased from Silicycle (silica gel 60F254; 0.25 mm thickness), or on precoated aluminum-backed plates purchased from Whatman (silica gel 60F254). Phosphomolybdic acid or *p*-anisaldehyde was used to visualize the compounds on TLC plates. Flash column chromatography was carried out with silica gel 60 (230-400 mesh ASTM) from Silicycle.

All reactions were conducted with anhydrous solvents in oven-dried glassware. Reactions under inert argon atmosphere were conducted by sealing the reaction vessel with a septum, and replacing the atmosphere with argon by three cycles of evacuating the atmosphere, then backfilling with argon after each evacuation. Reactants were used as received from commercial suppliers without prior purification, as were solvents used for extractions and chromatographic separations. All chemicals were purchased from Sigma Aldrich, Oakwood Chemical, TCI Chemicals, Ambeed, and Combi-blocks. Cs_2CO_3 (99.9% trace metal basis or 99% Reagent Plus) was purchased in 25 g quantities from Sigma Aldrich. CuI was purchased from Sigma Aldrich. Anhydrous THF was obtained from the Pure Process Purification solvent system. Methanol and acetone were used as received from commercial suppliers. Anhydrous DME (with 100 ppm of BHT inhibitor, extra dry (≥99.0%, ≤0.005% H_2O), containing 4Å molecular sieves) was purchased from Acros Organic. Reactions conducted above room temperature were heated in an external silicone oil bath within an evaporating dish placed on top of a dual stirrer / hot plate (Ceramag Midi), magnetically stirred, with a thermometer measuring the oil bath temperature.

Compounds that were susceptible to hydrolysis (e.g. vinylic ethers, TBS-protected diols) were purified using silica gel pre-treated with 1-2% triethylamine, due to the mildly acidic nature of silica gel used for flash column chromatography.

Synthesis of benzothiazole-sulfone 7 from D-galactose bis-acetonide (6):

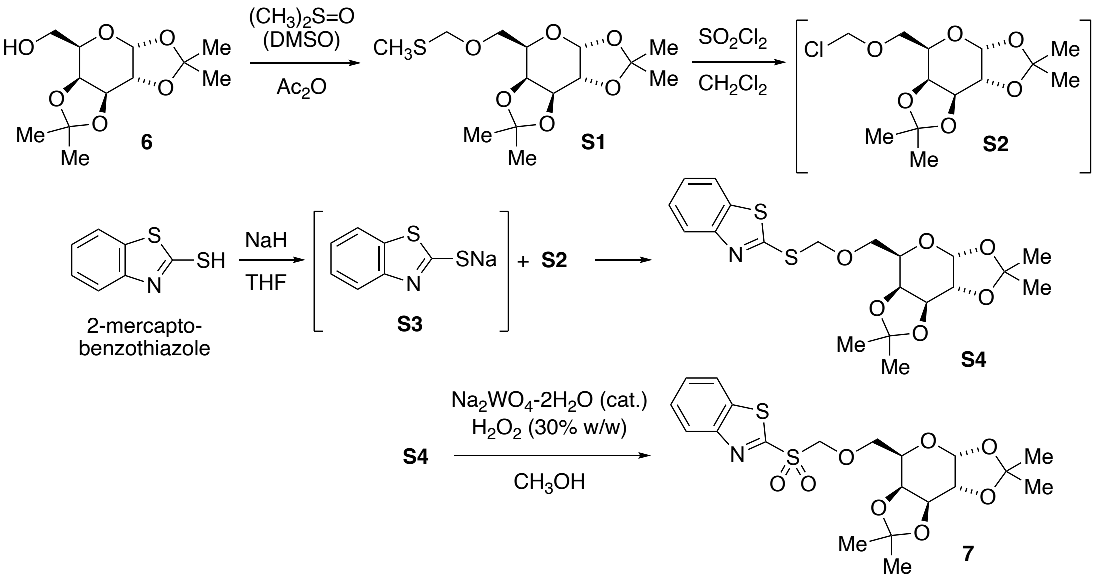

1,2:3,4-Di-*O*-isopropylidene-6-*O*-(methylthio)methyl-α-D-galactopyranose (**S1**):[1]

A 500 mL round bottom flask with magnetic stir bar was charged with D-galactose bis-acetonide (1,2:3,4-di-*O*-isopropylidene-α-D-galactopyranose, **6**, 21.870 g, 84.02 mmol), dissolved in acetic anhydride (58 mL, 1.46 M), and dimethylsulfoxide (DMSO, 87 mL, 0.97M) was added. The reaction mixture stirred at room temperature for 46 h. The reaction mixture was quenched by pouring into ice-cold saturated aqueous sodium bicarbonate solution, and diluted with EtOAc (100 mL), deionized water (100 mL), and brine (20 mL). The aqueous layer was extracted three times with EtOAc, the combined organic layers were dried over $MgSO_4$, filtered through a glass-fritted funnel, and the filtrate concentrated by rotary evaporation to give a clear light yellow oil (30 g). The crude oil was purified via column chromatography eluting with 90/10 Hexanes/EtOAc → 50/10 Hexanes/EtOAc to give **S1** as a clear light-yellow oil (17.100 g, 64% yield).

$[\alpha]_D^{20}$ = -246.3 ($CHCl_3$, *c* = 0.027)

^1H NMR (600 MHz; $CDCl_3$): δ 5.52 (d, *J* = 5.0 Hz, 1H), 4.72 (d, *J* = 11.4 Hz, 1H), 4.65 (d, *J* = 11.4 Hz, 1H), 4.59 (dd, *J* = 7.9, 2.4 Hz, 1H), 4.30 (dd, *J* = 5.1, 2.4 Hz, 1H), 4.24 (dd, *J* = 7.9, 1.9 Hz, 1H), 3.97 (ddd, *J* = 7.1, 5.3, 1.8 Hz, 1H), 3.76 (dd, *J* = 10.3, 5.4 Hz, 1H), 3.65 (dd, *J* = 10.2, 7.1 Hz, 1H), 2.13 (s, 3H), 1.52 (s, 3H), 1.43 (s, 3H), 1.32 (s, 3H), 1.31 (s, 3H).

$^{13}C\{^1H\}$ NMR (151 MHz; $CDCl_3$): δ 109.3, 108.5, 96.4, 75.6, 71.2, 70.7, 70.5, 66.8, 66.7, 26.04, 25.97, 24.9, 24.5, 13.8.

HRMS (ESI) calculated for $C_{14}H_{25}O_6S^+$ $[M + H]^+$ 321.1366, found 321.1368.

R_f = 0.41 (86/14 Hexanes/EtOAc)

1,2:3,4-Di-*O*-isopropylidene-6-*O*-(2-(benzothiazolyl)thio)methyl-α-D-galactopyranose (**S4**):

A 500 mL round bottom flask with magnetic stir bar was charged with **S1** (14.000 g, 43.70 mmol, 1.1 equiv), dissolved in CH_2Cl_2 (218 mL, 0.2 M), and externally cooled to 0 °C with an ice bath. Sulfuryl chloride (1.0 M in CH_2Cl_2, 47.7 mL, 47.66 mmol, 1.2 equiv) was added. After stirring at 0 °C for 15 minutes, the stir bar was removed and the volatiles were removed by rotary evaporation, providing the highly reactive and electrophilic chloromethyl ether intermediate **S2**.

> Note: The conversion of **S1** to **S2** was also successfully conducted with BCl_3 (1.0 M in hexanes, 1.1 equiv) in CH_2Cl_2 at 0 °C. However, the reaction with BCl_3 required several hours for complete conversion, whereas the reaction with SO_2Cl_2 required only 15 minutes for complete conversion of **S1**.

At the same time, a second 500 mL round bottom flask with magnetic stir bar was charged with 2-mercaptobenzothiazole (6.643 g, 39.72 mmol, 1.0 equiv), dissolved in THF (175 mL, 0.2 M), and externally cooled to 0 °C with an ice bath. 60% w/w sodium hydride (1.821 g, 43.70 mmol, 1.1 equiv) was slowly added. The resulting yellow bubbling mixture was stirred at 0 °C for 20 minutes, the ice bath was removed, and then stirred at room temperature for 40 minutes, producing a solution of the thiolate intermediate **S3**. The THF solution of **S3** was cooled to 0 °C with the external ice bath.

Crude **S2** was diluted in THF (25 mL), and transferred by syringe into the first 500 mL round bottom flask, cooled to 0 °C, containing the THF solution of thiolate intermediate **S3**. The resulting cloudy lime-colored mixture was stirred at room temperature for 16 hours, cooled to 0 °C with an external ice bath, and quenched with saturated aqueous NH_4Cl (20 mL). The mixture was diluted with deionized water (80 mL) and brine (20 mL). The aqueous layer was extracted with EtOAc (3 x 50 mL) and the combined organic layers were dried over $MgSO_4$, filtered through a glass-fritted funnel, and the filtrate concentrated by rotary evaporation to give **S4** as an orange oil (18.816 g, 42.81 mmol, 98% yield) that required no additional purification steps.

$[\alpha]_D^{20}$ = -204.9 ($CHCl_3$, *c* = 0.027)

^{1}H NMR (600 MHz; $CDCl_3$): δ 7.87 (dd, *J* = 8.1, 0.9 Hz, 1H), 7.73 (dd, *J* = 8.1, 0.9 Hz, 1H), 7.39 (ddd, *J* = 8.3, 7.2, 1.2 Hz, 1H), 7.28 (ddd, *J* = 8.2, 7.2, 1.3 Hz, 1H), 5.57 (d, *J* = 11.3 Hz, 1H), 5.51 (d, *J* = 5.1 Hz, 1H), 5.40 (d, *J* = 11.4 Hz, 1H), 4.56 (dd, *J* = 7.9, 2.4 Hz, 1H), 4.29 (dd, *J* = 5.1, 2.4 Hz, 1H), 4.18 (dd, *J* = 7.9, 1.9 Hz, 1H), 4.08 (m, 1H), 3.88 (dd, *J* = 10.6, 5.1 Hz, 1H), 3.80 (dd, *J* = 10.3, 7.0 Hz, 1H), 1.48 (s, 3H), 1.40 (s, 3H), 1.29 (s, 3H), 1.25 (s, 3H).

^{13}C{^{1}H} NMR (151 MHz; $CDCl_3$): δ 165.3, 153.0, 135.7, 126.1, 124.5, 121.9, 120.9, 109.4, 108.7, 96.4, 75.0, 71.1, 70.7, 70.5, 68.1, 66.6, 26.1, 26.0, 25.0, 24.4.

HRMS (ESI) calculated for $C_{20}H_{26}O_6NS_2^+$ $[M + H]^+$ 440.1196, found 440.1203.

R_f = 0.35 (80/20 Hexanes/EtOAc).

1,2:3,4-Di-*O*-isopropylidene-6-*O*-[2-(benzothiazolyl)sulfonyl]methyl-α-D-galactopyranose (**7**):

A 1-liter three-neck round bottom flask with magnetic stir bar and internal thermometer was charged with **S4** (7.242 g, 16.48 mmol) dissolved in methanol (290 mL, 0.056 M) and externally cooled with an ice bath. Sodium tungstate dihydrate (0.214 g, 0.65 mmol, 0.5 equiv) was added. The internal temperature remained at 1 °C. H_2O_2 (30% w/w in water, 3.35 mL, 33 mmol, 2 equiv) was added dropwise over 135 seconds: the internal temperature rose from 1 °C to 4 °C, as the reaction mixture acquired a lime-green color. With external ice bath cooling, the reaction mixture gradually turned cloudy as it stirred for 30 minutes, as the temperature cooled to 1 °C. A second portion of H_2O_2 (30% w/w in water, 3.35 mL, 33 mmol, 2 equiv) was added dropwise over 125 seconds. The resulting cloudy lime-yellow suspension stirred at 0 °C for 20 minutes, the external cooling bath was removed, and stirred for 24 hours at room temperature. The reaction mixture was cooled to 0 °C with an external ice bath, then quenched by dropwise addition of aqueous sodium metabisulfite solution (0.5 M, 50 mL) over 25 minutes. The internal temperature reached 12 °C in the first 15 minutes of the quench, and the mixture became cloudy white, but with external cooling the temperature dropped to 2 °C after sodium metabisulfite addition was complete. The reaction mixture was diluted with CH_2Cl_2 (300 mL), deionized water (150 mL), and brine (50 mL). The aqueous layer was extracted three times with CH_2Cl_2. The combined organic layers were dried over $MgSO_4$, filtered through a glass-fritted funnel, and the filtrate concentrated by rotary evaporation to give a clear oil, which was purified by silica gel column chromatography eluting with 75/25 Hexanes/EtOAc → 50/50 Hexanes/EtOAc to give **7** as a clear, viscous oil (4.427 g, 57% yield).

$[\alpha]_D^{20}$ = -160.7 ($CHCl_3$, *c* = 0.025)

^1H NMR (600 MHz; $CDCl_3$): δ 8.22 (ddd, *J* = 8.2, 1.3, 0.6 Hz, 1H), 7.99 (ddd, *J* = 8.0, 1.5, 0.6 Hz, 1H), 7.62 (ddd, *J* = 8.4, 7.2, 1.3 Hz, 1H), 7.58 (ddd, *J* = 8.2, 7.2, 1.3 Hz, 1H), 5.46 (d, *J* = 5.0 Hz, 1H), 5.14 (d, *J* = 12.9 Hz, 1H), 5.02 (d, *J* = 12.9 Hz, 1H), 4.55 (dd, *J* = 7.9, 2.5 Hz, 1H), 4.27 (dd, *J* = 5.0, 2.5 Hz, 1H), 4.14-4.11 (overlapping m, 2H), 3.99-3.92 (overlapping m, 2H), 1.42 (s, 3H), 1.39 (s, 3H), 1.274 (s, 3H), 1.267 (s, 3H).

$^{13}C\{^1H\}$ NMR (151 MHz; $CDCl_3$): δ 164.4, 153.0, 137.4, 128.2, 127.7, 125.8, 122.3, 109.6, 108.8, 96.3, 86.1, 72.7, 70.9, 70.7, 70.4, 67.1, 26.03, 26.02, 25.0, 24.5.

HRMS (ESI) calculated for $C_{20}H_{26}O_8NS_2^+$ $[M + H]^+$ 472.1094, found 472.1101.

R_f = 0.35 (67/33 Hexanes/EtOAc).

Attempts to perform the sulfide-to-sulfone oxidation with other oxidants or on larger scale were unsuccessful, likely due to heat buildup from exothermic reactions in the initial stage of the oxidation step, and in the quench. Side products corresponding to solvolysis of sulfide reactant, sulfoxide intermediate, and/or sulfone product included benzothiazole **S5** and the methoxymethyl ether **S6**. After we completed this stage of the project, we learned that others also reported difficulties synthesizing sulfones from *alpha*-alkoxybenzothiazolylsulfides.[2]

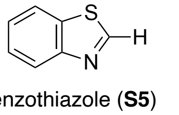

benzothiazole (**S5**)

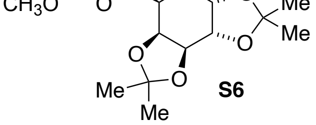

S6

Synthesis of aldehyde 9 from D-lyxose acetonide (8):

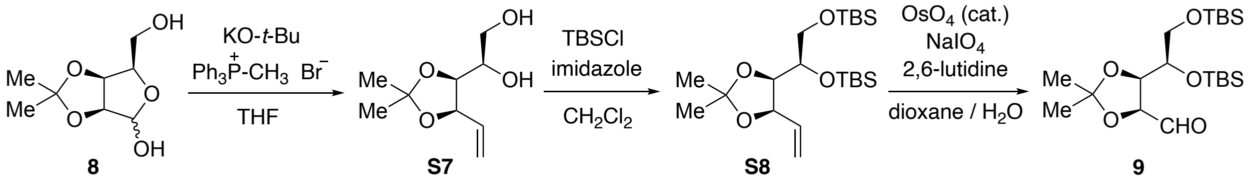

(*R*)-1-((4*S*,5*R*)-2,2-dimethyl-5-vinyl-1,3-dioxolan-4-yl)ethane-1,2-diol (**S7**):

An oven-dried 250 mL round bottom flask with magnetic stir bar was charged with methyltriphenylphosphonium bromide (19.720 g, 55.20 mmol, 3.5 equiv) in tetrahydrofuran (THF, 70 mL), and externally cooled to 0 °C with an ice bath. Potassium *tert*-butoxide (5.662 g, 50.46 mmol, 3.2 equiv) was added. The resulting yellow mixture was stirred at 0 °C for 20 minutes, the ice bath was removed, and the mixture stirred at room temperature for 1 hour. The mixture was re-cooled to 0 °C. A solution of D-lyxose acetonide **8**[3] (3.000 g, 15.77 mmol, 1.0 equiv) dissolved in THF (30 mL) was added by syringe transfer. The ice bath was removed, and the resulting opaque, tan-colored mixture stirred at room temperature for 12 hours. The reaction mixture was quenched with ice-cooled deionized water (50 mL), and diluted with ethyl acetate (EtOAc, 50 mL) and brine (20 mL). The aqueous layer was extracted three times with EtOAc. The combined organic layers were washed with deionized water and brine, and dried over $MgSO_4$. The solution was filtered through a glass-fritted funnel, and the filtrate concentrated with rotary evaporation to give a dark yellow oil (14.584 g). The crude oil was subjected to silica gel column chromatography, eluting with 50/50 EtOAc/Hexanes → 75/25 EtOAc/Hexanes to give **S7** mixed with some inseparable triphenylphosphine oxide byproduct as a yellow oil (3.070 g).

This step was scaled up three-fold (methyltriphenylphosphonium bromide, 59.159 g, 165.6 mmol; potassium *tert*-butoxide, 16.987 g, 151.4 mmol; D-lyxose acetonide **8**, 9.000 g, 47.31 mmol) to generate larger quantities of **S7** for subsequent steps. However, on this scale, the byproduct triphenylphosphine oxide was more difficult to remove (18.047 g total mass, containing estimated 10 g of **S7**). This material, although contaminated with triphenylphosphine oxide, was used in the next step without any complications.

(*R*)-5-((4*R*,5*R*)-2,2-dimethyl-5-vinyl-1,3-dioxolan-4-yl)-2,2,3,3,8,8,9,9-octamethyl-4,7-dioxa-3,8-disiladecane (**S8**):

A 500 mL round bottom flask with magnetic stir bar was charged with a mixture of **S7** contaminated with triphenylphosphine oxide (18 g total mass, containing estimated 10 g of **S7**, 53 mmol), arising from two batches of the larger scale preparation. This mixture was dissolved in CH_2Cl_2 (300 mL, est. 0.2 M), and externally cooled to 0 °C with an ice bath. Imidazole (25.319 g, 371.91 mmol, 7.0 equiv) and *t*-butyldimethylsilyl chloride (TBSCl, 40.039 g, 265.65 mmol, 5.0 equiv) were added. The ice bath was removed, and the resulting mixture stirred at room temperature for 12 hours. The reaction mixture was quenched with ice-cooled deionized water (150 mL) and brine (30 mL). The aqueous layer was extracted with CH_2Cl_2 (3 x 100 mL), and the combined organic layers were dried over $MgSO_4$. The solution was filtered through a glass-fritted funnel, and the filtrate concentrated with rotary evaporation to give a yellow oil. The crude oil was purified in two batches by silica gel column chromatography, eluting with 90/10 Hexanes/EtOAc to give **S8** (33.685 g) contaminated with an organosilicon impurity.

^{1}H NMR (600 MHz; $CDCl_3$): δ 5.91 (ddd, *J* = 17.2, 10.3, 8.0 Hz, 1H), 5.25 (ddd, *J* = 17.2, 1.7, 1.0 Hz, 1H), 5.18 (ddd, *J* = 10.3, 1.8, 0.9 Hz, 1H), 4.45 (dd, *J* = 8.1, 7.1 Hz, 1H), 4.12 (dd, *J* = 7.2, 6.2 Hz, 1H), 3.71 (ddd, *J* = 7.2, 5.3, 4.6 Hz, 1H), 3.57 (d, *J* = 5.4 Hz, 1H), 3.55 (d, *J* = 4.7 Hz, 1H), 1.45 (s, 3H), 1.31 (s, 3H), 0.87 (s, 9H), 0.86 (s, 9H), 0.06 (s, 3H), 0.05 (s, 3H), 0.02 (s, 3H), 0.01 (s, 3H).

$^{13}C\{^{1}H\}$ NMR (151 MHz; $CDCl_3$): δ 133.7, 118.2, 108.0, 79.0, 78.8, 72.6, 65.1, 27.8, 25.93, 25.91, 25.4, 18.4, 18.3, -4.3, -4.5, -5.41, -5.44.

HRMS (NSI) calculated for $C_{21}H_{44}O_4Si_2Na^+$ $[M + Na]^+$ 439.2676, found 439.2668.

(4*S*,5*R*)-2,2-dimethyl-5-((*R*)-2,2,3,3,8,8,9,9-octamethyl-4,7-dioxa-3,8-disiladecan-5-yl)-1,3-dioxolane-4-carbaldehyde (**9**):

A 500 mL round bottom flask with magnetic stir bar was charged with **S8** (33.685 g contaminated with organosilicon impurity, containing estimated 10 g of **S8**, 24 mmol), dissolved in dioxane / water mixture (3 : 1 ratio, 240 mL), at room temperature. 2,6-Lutidine (4.2 mL, 36 mmol, 1.5 equiv) was added, followed by osmium tetraoxide (20 drops of a 2% w/v solution in toluene), followed by sodium periodate (25.663 g, 120 mmol, 5.0 equiv). After 17 hours, alkene was still present by TLC analysis, so 2,6-lutidine (2.2 mL, 0.8 equiv), osmium tetraoxide (10 drops of 2% solution in toluene), and sodium periodate (10 g, 2 equiv) were added. After 3 days, the major TLC spot corresponded to aldehyde, but to fully consume alkene, 2,6-lutidine (2.2 mL, 0.8 equiv), osmium tetraoxide (10 drops of 2% solution in toluene), and sodium periodate (10 g, 2 equiv) were added. After 27 hours, the reaction mixture was diluted with deionized water (100 mL) and brine (75 mL). The reaction mixture was extracted three times with CH_2Cl_2, the combined organic layers were filtered through Celite, and concentrated with rotary evaporation to give a black-yellow oil (17.447 g). The crude oil was purified by silica gel column chromatography, eluting with 95/5 Hexanes/EtOAc → 91/9 Hexanes/EtOAc, to give aldehyde **9** (9.494 g, 48% combined yield over three steps beginning with 9.00 g of D-lyxose acetonide **8**).

$[\alpha]_D^{20}$ = +2.4 ($CHCl_3$, *c* = 0.025)

^{1}H NMR (600 MHz; $CDCl_3$): δ 9.75 (d, *J* = 2.3 Hz, 1H), 4.51 (dd, *J* = 7.7, 4.2 Hz, 1H), 4.40 (dd, *J* = 7.7, 2.3 Hz, 1H), 3.86 (app q, *J* = 4.0 Hz, 1H), 3.66 (app d, *J* = 6.3 Hz, 2H), 1.57 (s, 3H), 1.38 (s, 3H), 0.90 (s, 9H), 0.88 (s, 9H), 0.07 (s, 6H), 0.06 (s, 6H).

$^{13}C\{^{1}H\}$ NMR (151 MHz; $CDCl_3$): δ 201.3, 110.1, 80.6, 79.7, 70.9, 64.2, 26.8, 25.91 (2C), 25.1, 18.3, 18.2, -3.8, -4.5, -5.45, -5.49.

HRMS (ESI) calculated for $C_{20}H_{42}O_5Si_2^+$ $[M + H]^+$ 419.2644, found 419.2641.

Model experiments for sulfone preparation:

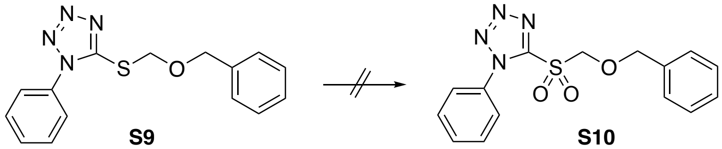
S9 S10

Oxidizing heterocyclic alkoxysulfides to corresponding sulfones was unexpectedly challenging. For example, *N*-phenyltetrazolylsulfide **S9** did not produce sulfone **S10** under any conditions attempted, including $(NH_4)_2Mo_7O_{24}$-$4H_2O$ / 30% H_2O_2, Na_2WO_4-$2H_2O$ / 30% H_2O_2, *m*-CPBA, or urea-hydrogen peroxide (UHP), releasing only protonolysis product *N*-phenyltetrazole.

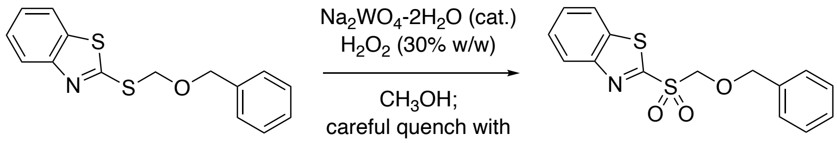

S11 S12

Oxidizing benzothiazolylsulfide **S11** to sulfone **S12** similarly failed with $(NH_4)_2Mo_7O_{24}$-$4H_2O$ / 30% H_2O_2, and initially with Na_2WO_4-$2H_2O$ / 30% H_2O_2, until we observed that reductive quench was exothermic. Slow addition of 0.5 M aqueous sodium metabisulfite to an ice-cold reaction mixture, with efficient cooling, reproducibly produced **S12**.

Model experiments for modified Julia olefination:

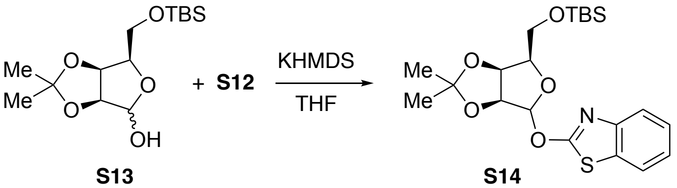

S13 S14

Attempted olefination of cyclic hemiacetal **S13** with alkoxysulfone **12** gave a mixture of several substances, none corresponding to vinylic ethers, although a possible side-product **S14** was isolated, with formula confirmed by high resolution mass spectrometry.

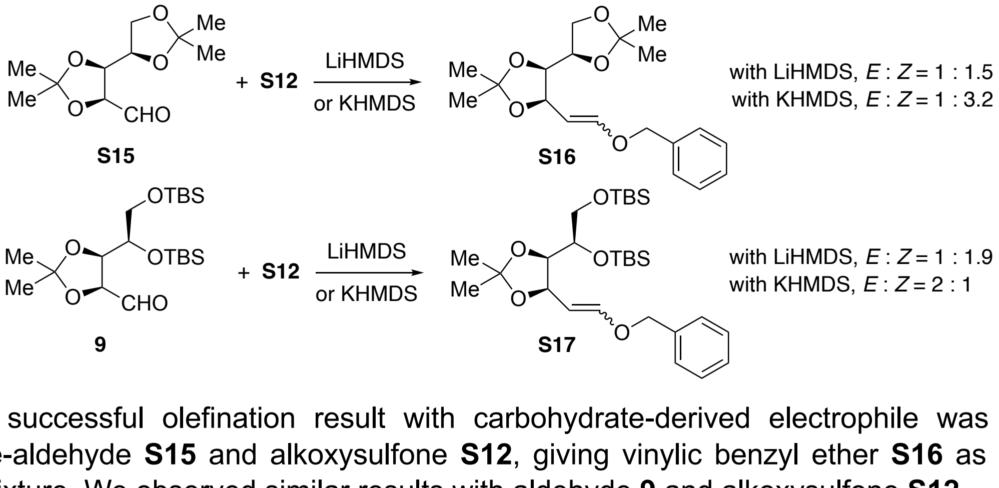

Our first successful olefination result with carbohydrate-derived electrophile was with bis-acetonide-aldehyde **S15** and alkoxysulfone **S12**, giving vinylic benzyl ether **S16** as an *E* / *Z*-isomer mixture. We observed similar results with aldehyde **9** and alkoxysulfone **S12**.

Modified Julia olefination to synthesize vinylic ether mixture (*E*)-10 and (*Z*)-11:

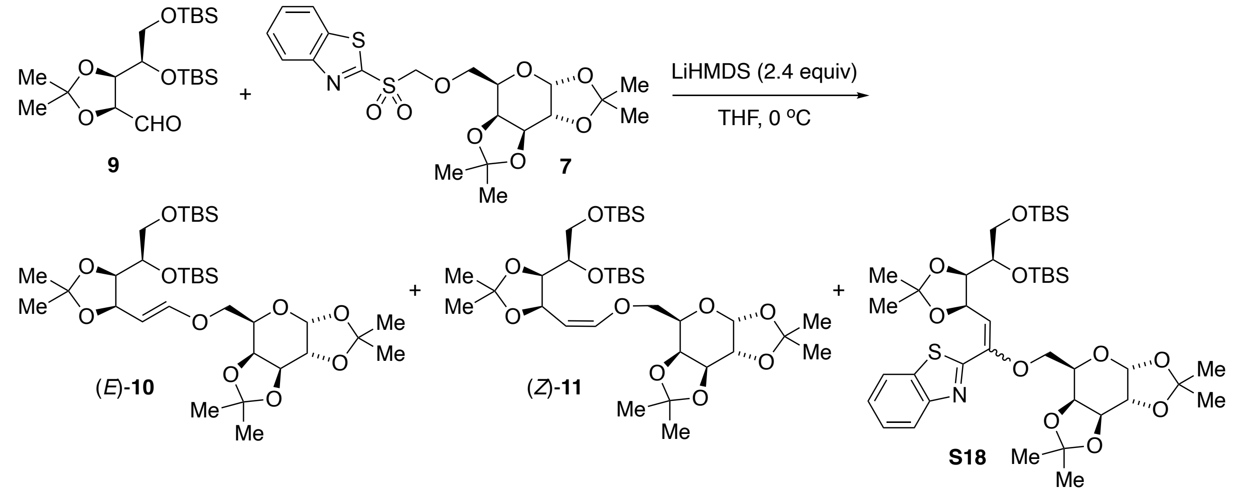

An oven-dried 250 mL round bottom flask with magnetic stir bar was charged with sulfone **7** (4.427 g, 9.39 mmol, 1.2 equiv) and aldehyde **9** (3.278 g, 7.83 mmol, 1.0 equiv), dissolved in THF (92 mL, 0.085 M), and externally cooled with an ice bath. LiHMDS (1.0 M in THF) (18.8 mL, 18.8 mmol, 2.4 equiv) was added slowly over a period of 16 minutes. The resulting dark orange solution was stirred at 0 °C for 90 minutes, quenched with slow addition of a solution of saturated aqueous NH_4Cl (25 mL) while maintaining external cooling at 0 °C, and diluted with deionized water (50 mL) and brine (20 mL). The aqueous layer was extracted with EtOAc (3 x 50 mL). The combined organic layers were dried over $MgSO_4$, filtered through a glass-fritted funnel, and the filtrate concentrated by rotary evaporation to give a dark orange-red oil (7.040 g). The crude oil was subjected to silica gel column chromatography eluting with 95/5 Hexanes/EtOAc → 88/12 Hexanes/EtOAc, to a mixture of (*E*)-**10** and (*Z*)-**11** as a foamy, viscous oil (2.128 g, 3.15 mmol, 40% yield, *E*/*Z* = 51/49).

The product mixture had similar R_f with a benzothiazole-containing side product, tentatively assigned as **S18**. This side product may have arisen from deprotonation of the vinylic ether product as it initially formed. Similar side products were observed in model experiments with sulfone **S12** with both aldehydes **S15** and **9**.

A small ~100 mg sample of the vinylic ether product mixture was partially purified by silica gel column chromatography eluting with 90/10 Hexanes/EtOAc, to provide a few fractions containing mostly (*Z*)-**11** (R_f 0.35), and (*E*)-**10** (R_f 0.31).

(*E*)-**10**:

^{1}H NMR (600 MHz; C_6D_6): δ 6.41 (d, *J* = 12.6 Hz, 1H), 5.47 (d, *J* = 5.0 Hz, 1H), 5.20 (dd, *J* = 12.6, 9.6 Hz, 1H), 4.46 (dd, *J* = 7.9, 2.4 Hz, 1H), 4.36 (dd, *J* = 9.6, 6.1 Hz, 1H), 4.21 (m, 2H), 4.14 (dd, *J* = 5.0, 2.4 Hz, 1H), 4.04 (dd, *J* = 10.1, 5.6 Hz, 1H), 4.00 (m, 2H), 3.91 (m, 1H), 3.69 (m, 2H), 1.52 (s, 3H), 1.44 (s, 3H), 1.42 (s, 3H), 1.33 (s, 3H), 1.13 (s, 3H), 1.11 (s, 9H), 1.02 (s, 3H), 0.98 (s, 9H), 0.29 (s, 3H), 0.24 (s, 3H), 0.12 (s, 3H), 0.09 (s, 3H).

(*Z*)-**11**:

^{1}H NMR (600 MHz; C_6D_6): δ 5.94 (dd, *J* = 6.3, 1.0 Hz, 1H), 5.43 (d, *J* = 5.0 Hz, 1H), 5.32 (ddd, *J* = 9.7, 5.9, 1.0 Hz, 1H), 4.67 (dd, *J* = 9.8, 6.3 Hz, 1H), 4.43 (dd, *J* = 7.9, 2.4 Hz, 1H), 4.25 (dd, *J* = 7.3, 5.9 Hz, 1H), 4.12 (dd, *J* = 5.0, 2.4 Hz, 1H), 4.06 (ddd, *J* = 8.1, 6.3, 1.8 Hz, 1H), 3.99 (dd, *J* =

10.6, 6.3 Hz, 1H), 3.96 (dd, J = 8.0, 1.9 Hz, 1H), 3.93 (ddd, J = 9.2, 5.9, 3.2 Hz, 1H), 3.87 (dd, J = 10.6, 6.3 Hz, 1H), 3.75 (dd, J = 10.3, 3.2 Hz, 1H), 3.68 (dd, J = 10.3, 6.0 Hz, 1H), 1.52 (s, 3H), 1.45 (s, 3H), 1.42 (s, 3H), 1.32 (s, 3H), 1.11 (s, 9H), 1.10 (s, 3H), 1.03 (s, 3H), 1.00 (s, 9H), 0.27 (s, 3H), 0.25 (s, 3H), 0.11 (s, 3H), 0.10 (s, 3H)

Deprotection of silyl ethers to give mixture of diol vinylic ethers (*E*)-12 and (*Z*)-13:

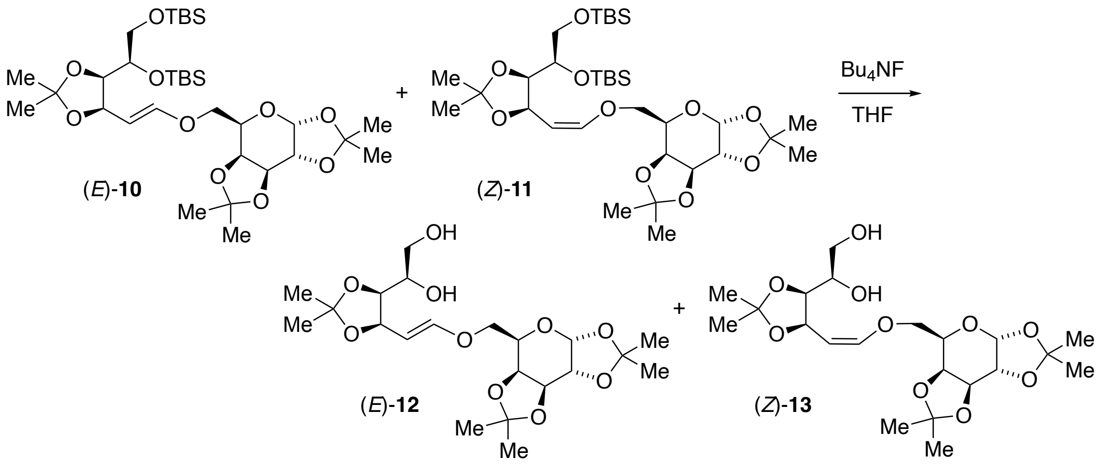

A 100 mL round bottom flask with magnetic stir bar was charged with a mixture of (*E*)-**12** and (*Z*)-**13** (1.690 g, 2.50 mmol, *E* : *Z* = 64 : 36, 1.0 equiv) dissolved in THF (50 mL), and externally cooled with an ice bath. Tetrabutylammonium fluoride (1.0 M in THF, 8.8 mL, 8.8 mmol, 3.5 equiv) was added slowly, causing the color to become dark orange but remaining clear. The cooling bath was removed, allowing the reaction mixture to warm to room temperature over a period of 5 hours. The reaction mixture was cooled with an external ice bath, and quenched by slowly adding a solution of saturated aqueous NH_4Cl (10 mL), and diluting with deionized water (30 mL) and brine (10 mL). The aqueous layer was extracted with EtOAc (3 x 30 mL). The combined organic layers were dried over $MgSO_4$, filtered through a glass fritted funnel, and the filtrate concentrated by rotary evaporation to give an orange oil (1.974 g). The crude oil was subjected to silica gel column chromatography eluting with 25/75 hexanes/EtOAc, to a mixture of (*E*)-**12** and (*Z*)-**13** as a clear yellow oil (0.508 g, 46% yield, *E* : *Z* = 58 : 42). These diastereomers were inseparable by column chromatography (R_f 0.31).

The following ^{1}H NMR data are from the mixture of (*E*)-**12** and (*Z*)-**13**, assigned to each diastereomer by relative integration values (1.00 / 0.70).

(*E*)-**12**:

^{1}H NMR (600 MHz; C_6D_6): δ 6.41 (d, J = 12.6 Hz, 1H), 5.49 (d, J = 5.1 Hz, 1H), 5.26 (dd, J = 12.7, 9.4 Hz, 1H), 4.47 (dd, J = 7.9, 2.4 Hz, 1H), 4.32 (dd, J = 9.4, 6.9 Hz, 1H), 4.19 (ddd, J = 7.3, 5.9, 1.9 Hz, 1H), 4.16 (dd, J = 5.0, 2.3 Hz, 1H), 4.06 (dd, J = 10.3, 6.7 Hz, 1H), 4.02 (dd, J = 10.3, 5.6 Hz, 1H), 4.00 (dd, J = 7.9, 1.9 Hz, 1H), 3.91 - 3.79 (m, 2H), 3.58 (m, 2H), 1.44 (s, 3H), 1.43 (s, 3H), 1.42 (s, 3H), 1.24 (s, 3H), 1.15 (s, 3H), 1.05 (s, 3H).

(*Z*)-**13**:

^{1}H NMR (600 MHz; C_6D_6): δ 5.94 (dd, J = 6.3, 1.2 Hz, 1H), 5.44 (ddd, J = 8.3, 6.7, 1.2 Hz, 1H), 5.41 (d, J = 5.0 Hz, 1H), 4.82 (dd, J = 8.7, 6.3 Hz, 1H), 4.41 (dd, J = 7.9, 2.4 Hz, 1H), 4.24 (dd, J = 6.8, 4.7 Hz, 1H), 4.11 (dd, J = 5.0, 2.4 Hz, 1H), 3.96 (ddd, J = 6.8, 4.5, 1.8 Hz, 1H), 3.91 - 3.79 (m, 2H), 3.77 (dd, J = 11.2, 4.2 Hz, 1H), 3.73 (dd, J = 11.2, 6.1 Hz, 1H), 3.58 (m, 2H), 1.46 (s, 3H), 1.45 (s, 3H), 1.41 (s, 3H), 1.28 (s, 3H), 1.11 (s, 3H), 1.04 (s, 3H).

Epoxidation-oxacyclization of (*E*)-12 and (*Z*)-13 mixture to disaccharides 14 and 15:

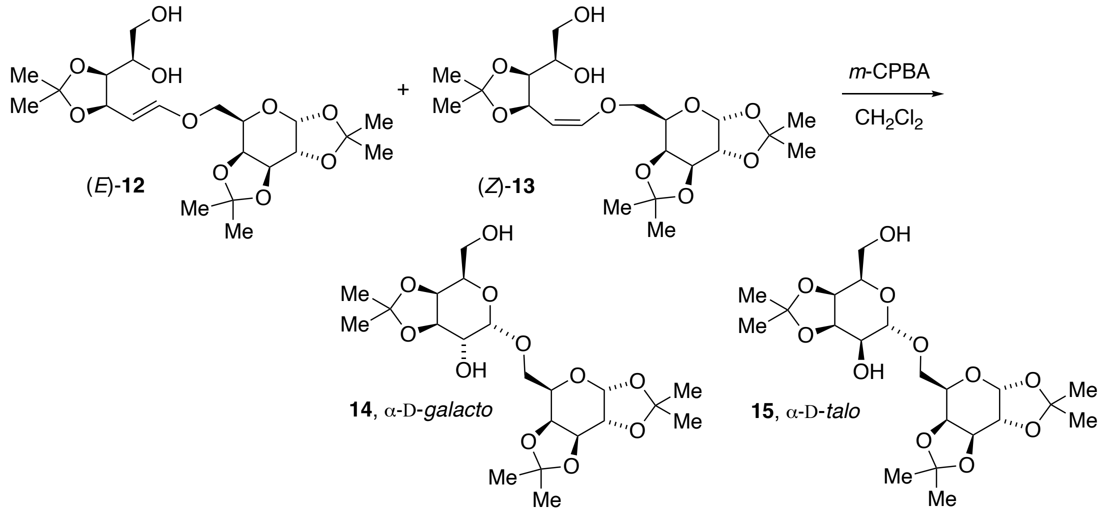

1,2:3,4-Di-*O*-(isopropylidene)-6-*O*-[3,4-*O*-(isopropylidene)-α-D-galactopyranosyl]-α-D-galactopyranose (**14**) + 1,2:3,4-Di-*O*-(isopropylidene)-6-*O*-[3,4-*O*-(isopropylidene)-α-D-talopyranosyl]-α-D-galactopyranose (**15**)

A 50 mL round-bottom flask with stir bar was charged with a mixture of diol-vinylic ethers *E*)-**12** and (*Z*)-**13** (0.405 g, 0.91 mmol, *E* : *Z* = 58 : 42, 1.0 equiv), and cooled to 0 °C with an external cooling bath. *m*-CPBA (77% w/w) (0.224 g, 1.00 mmol, 1.1 equiv) was slowly added to the stirring solution at 0 °C. The resulting suspension was stirred for 15 minutes at 0 °C, the external cooling bath was removed, and the reaction mixture stirred for 5 h at room temperature. The reaction mixture was stored in a 4 °C refrigerator overnight, during which time a white precipitate was observed on the walls of the flask, presumably *m*-chlorobenzoic acid. The heterogenous reaction mixture was diluted with cold CH_2Cl_2 and filtered through a glass fritted funnel (washing 3 x 20 mL of CH_2Cl_2) to remove the precipitate. To the filtrate, a saturated aqueous solution of $NaHCO_3$ (5 mL), deionized water (50 mL), and brine (10 mL) was added. The aqueous layer was extracted with CH_2Cl_2 (3 x 30 mL), and the combined organic layers were dried over $MgSO_4$, filtered through a glass fritted funnel, and the filtrate was concentrated by rotary evaporation to give a viscous yellow oil. The crude product was purified by silica gel flash chromatography (eluting with hexanes/EtOAc 1 : 1 → 1 : 5 gradient). Compound **14** eluted first (R_f 0.31, 45 mg), followed by fractions containing a mixture of **14** and **15** (63 mg), followed by fractions containing primarily compound **15** inseparable from a minor isomer (4.4 : 1 ratio, R_f 0.21, 86 mg).

1,2:3,4-Di-*O*-(isopropylidene)-6-*O*-[3,4-*O*-(isopropylidene)-α-D-galactopyranosyl]-α-D-galactopyranose (**14**):

^{1}H NMR (600 MHz; C_6D_6) δ 5.49 (d, *J* = 5.0 Hz, 1H), 4.95 (d, *J* = 3.8 Hz, 1H), 4.53 (dd, *J* = 7.9, 2.4 Hz, 1H), 4.31 (t, *J* = 6.1 Hz, 1H), 4.24 (ddd, *J* = 6.9, 4.2, 2.2 Hz, 1H), 4.22 (ddd, *J* = 7.4, 6.5, 1.9 Hz, 1H), 4.16 (dd, *J* = 5.0, 2.4 Hz, 1H), 4.11 (dd, *J* = 7.9, 1.9 Hz, 1H), 4.03 (dd, *J* = 10.8, 6.9 Hz, 1H), 3.99 (dd, *J* = 11.6, 7.4 Hz, 1H), 3.97 (dd, *J* = 5.8, 3.7 Hz, 1H), 3.93 (dd, *J* = 6.4, 2.2 Hz, 1H), 3.86 (dd, *J* = 10.9, 6.9 Hz, 1H), 3.83 (broad dd, *J* = 11.7, 4.2 Hz, 1H), 2.94 (s, 1H), 2.38 (s, 1H), 1.47 (s, 3H), 1.46 (s, 3H), 1.42 (s, 3H), 1.17 (s, 3H), 1.16 (s, 3H), 1.03 (s, 3H).

$^{13}C\{^1H\}$ NMR (151 MHz; C_6D_6): δ 109.50, 109.49, 108.7, 98.5, 96.9, 76.2, 73.7, 71.5, 71.2, 71.0, 70.2, 69.2, 67.9, 66.8, 62.9, 27.6, 26.24, 26.18, 25.8, 24.8, 24.5.

HRMS (NSI) calculated for $C_{21}H_{34}O_{11}Na_1^+$ [M + Na]$^+$ 485.1993, found 485.1997.

1,2:3,4-Di-*O*-(isopropylidene)-6-*O*-[3,4-*O*-(isopropylidene)-α-D-talopyranosyl]-α-D-galactopyranose (**15**):

^{1}H NMR (600 MHz; C_6D_6) δ 5.52 (d, *J* = 5.0 Hz, 1H), 4.99 (d, *J* = 6.0 Hz, 1H), 4.54 (dd, *J* = 7.9, 2.4 Hz, 1H), 4.30 (ddd, *J* = 8.2, 6.7, 1.7 Hz, 1H), 4.24 (dd, *J* = 7.6, 3.0 Hz, 1H), 4.20 (dd, *J* = 7.9, 1.8 Hz, 1H), 4.17 (dd, *J* = 5.0, 2.4 Hz, 1H), 4.09 (dd, *J* = 11.0, 6.9 Hz, 1H), 4.00 (dd, *J* = 11.0, 6.5 Hz, 1H), 3.94 (dd, *J* = 11.6, 7.5 Hz, 1H), 3.76 (broad dd, *J* = 11.7, 4.1 Hz, 1H), 3.72 (dd, *J* = 7.6, 1.9 Hz, 1H), 3.65 - 3.62 (m, 2H), 3.02 (s, 1H), 2.65 (s, 1H), 1.50 (s, 3H), 1.47 (s, 3H), 1.45 (s, 3H), 1.19 (s, 3H), 1.17 (s, 3H), 1.03 (s, 3H).

^{13}C{^{1}H} NMR (151 MHz; C_6D_6): δ 110.2, 109.5, 108.7, 102.4, 96.9, 74.9, 74.7, 71.5, 71.3, 71.2, 71.1, 69.5, 68.0, 66.9, 62.4, 26.33, 26.26, 26.19, 25.3, 24.8, 24.6.

HRMS (NSI) calculated for $C_{21}H_{35}O_{11}^{+}$ $[M + H]^{+}$ 463.2174, found 463.2177.

We assigned stereochemistry for disaccharides **14** and **15** by comparing ^{1}H-^{1}H coupling constants with literature reports for methyl β- and α-D-galactopyranosides,[4,5] and methyl α- and β-D-talopyranosides.[6] Data shown below are *J* values, in Hz.

Coupling constants for disaccharide **14** were measured at 600 MHz.

Coupling constants for disaccharide **15** were measured on a sample prepared by C-O cross-coupling (*vide infra*, page S-32), and were measured at 800 MHz. The 600 MHz spectrum of compound **15** arising from the modified Julia olefination exhibited two overlapping multiplets from 3.65 - 3.62, for which we could not accurately measure coupling constants.

For disaccharides **14** and **15**, a range of coupling constants indicates differences observed in couplings measured for specific ^{1}H resonances within a spectrum of the same sample.

	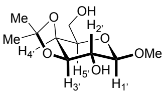	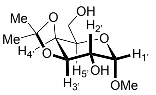	disaccharides		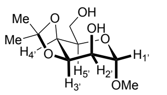	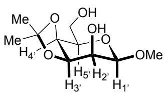
	methyl β-galacto-pyranoside	methyl α-galacto-pyranoside (**16**)	α-galacto-pyranoside (**14**)	α-talo-pyranoside (**15**)	methyl α-talo-pyranoside (**17**)	methyl β-talo-pyranoside
	$CDCl_3+D_2O$	CD_3OD	C_6D_6	C_6D_6	$CDCl_3$	$CDCl_3$
^{1}H-^{1}H	(ref. 4)	(ref. 5)	this research		(ref. 6)	(ref. 6)
1,2	8.3	3.6	3.7 - 3.8	5.9	5.4	1.9
2,3	7.2	7.7	5.8 - 6.1	3.1 - 3.4	3.4	4.7
3,4	5.6	5.5	6.1 - 6.4	7.6 - 8.0	7.5	6.6
4,5	2.0	2.5	2.2	1.9 - 2.0	1.8	2.3
5,6a	3.8	nr	6.9 - 7.4	7.6	7.5	8.2
5,6b	3.8	nr	4.2	3.9	nr	nr
6a,6b	8.8	nr	11.6 - 11.7	11.7	12.0	12.5

Assignments of diagnostic ^{13}C and ^{1}H NMR resonances for disaccharides **14** and **15** prepared by the modified Julia olefination route, determined by HSQC, COSY, and HMBC spectroscopy:

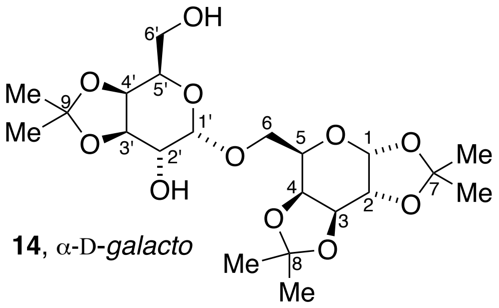

14, α-D-*galacto*

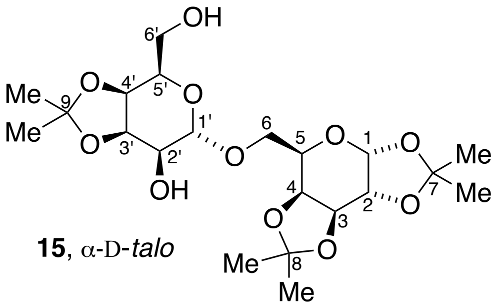

15, α-D-*talo*

carbon position	δ_C[a]	δ_H[b,c] (*J*, Hz)	δ_C[a]	δ_H[b,c] (*J*, Hz)
1'	98.5	4.95, d (3.8)	102.4	4.99, d (6.0)
2'	69.2	3.97, dd (5.8, 3.7)	69.5	3.62, m
3'	76.2	4.31, t (6.1)	74.7	4.24, dd (7.6, 3.0)
4'	73.7	3.93, dd (6.4, 2.2)	74.9	3.72, dd (7.6, 1.9)
5'	70.2	4.24, ddd (6.9, 4.2, 2.2)	71.2	3.65, m
6a'	62.9	3.99, dd (11.6, 7.4)	62.4	3.94, dd (11.6, 7.5)
6b'		3.83, dd (11.7, 4.2)		3.76, dd (11.7, 4.1)
1	96.6	5.49, d (5.0)	96.9	5.52, d (5.0)
2	71.0	4.16, dd (5.0, 2.4)	71.1	4.17, dd (5.0, 2.4)
3	71.2	4.52, dd (7.9, 2.4)	71.3	4.54, dd (7.9, 2.4)
4	71.5	4.11, dd (7.9, 1.8)	71.5	4.20, dd (7.9, 1.8)
5	66.8	4.22, ddd (7.4, 6.5, 1.9)	66.9	4.30, ddd (8.2, 6.7, 1.7)
6a	67.9	4.03, dd (10.8, 6.9)	68.0	4.09, dd (11.0, 6.9)
6b		3.86, dd (10.9, 6.9)	--	4.00, dd (11.0, 6.5)

[a] Recorded at 151 MHz in C_6D_6
[b] Recorded at 600 MHz in C_6D_6
[c] Denotes ^{1}H resonance(s) correlating to each ^{13}C for the carbon position, determined by HSQC.
[d] Due to overlapping ^{13}C peaks, these assignments are tentative.

Vinylic iodide (*E*)-19 from D-lyxose acetonide (8):

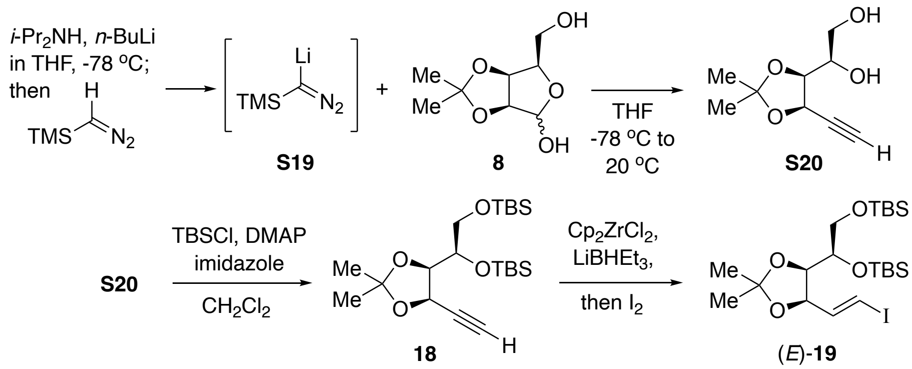

1,2-Dideoxy-3,4-*O*-(isopropylidene)-D-*lyxo*-hex-1-ynitol (**S20**):

An oven-dried 100-mL round-bottom flask with magnetic stir bar was charged with *i*-Pr_2NH (3.5 mL, 25 mmol), dissolved in THF (18 mL) and cooled to -78 °C with an external cooling bath of dry ice-acetone. *n*-BuLi (2.5 M in hexanes, 10 mL, 25 mmol) was slowly added, and the resulting mixture was stirred for 20 min at -78 °C. A solution of $TMSCHN_2$ (2.0 M in hexanes, 12.5 mL, 25 mmol) was then added dropwise at -78 °C, and this mixture stirred for 20 minutes to generate a THF solution of lithium trimethylsilyldiazomethane (**S19**).[7]

(2,3-*O*-isopropylidene)-D-lyxofuranose **8**[3] (0.95 g, 5.0 mmol) was dissolved in THF (6 mL), and this solution was slowly added to the -78 °C THF solution of **S19**. The resulting mixture was allowed to warm to room temperature overnight. The reaction mixture was quenched with saturated aqueous ammonium chloride (50 mL), and brine (20 mL) was added. The mixture was extracted with EtOAc (50 mL × 3). The organic layer was dried over anhydrous $MgSO_4$, filtered, and the filtrate was concentrated by rotary evaporation. Purification of the crude product by silica gel flash chromatography (CH_2Cl_2/EtOAc 1:1, gradient elution to 1:2) afforded the corresponding alkynyl diol **S20** (106 mg, 11% yield from D-lyxose acetonide **8**). Approximately 10% of compound **8** was recovered.

^{1}H NMR (400 MHz, C_6D_6) δ 4.50 (dd, *J* = 5.8, 2.3 Hz, 1H), 4.07 (ddd, *J* = 7.4, 5.5, 3.6 Hz, 1H), 3.96 (dd, *J* = 7.4, 5.8 Hz, 1H), 3.68 (dd, *J* = 11.6, 3.6 Hz, 1H), 3.59 (dd, *J* = 11.6, 5.4 Hz, 1H), 3.00 (br s, 2H), 2.03 (d, *J* = 2.2 Hz, 1H), 1.53 (s, 3H), 1.19 (d, *J* = 0.8 Hz, 3H).

^{13}C{^{1}H} NMR (101 MHz, C_6D_6) δ 110.4, 80.2, 78.6, 76.3, 72.4, 67.1, 63.5, 27.7, 26.0.

[5,6-Di-*O*-(*tert*-butyldimethylsilyl)]-1,2-dideoxy-3,4-*O*-(isopropylidene)-D-*lyxo*-hex-1-ynitol (**18**):

Imidazole (175 mg, 2.6 mmol, 6 equiv), *t*-butyldimethylsilyl chloride (TBSCl, 194 mg, 1.3 mmol, 3 equiv), and 4-(dimethylamino)pyridine (DMAP, 26 mg, 0.21 mmol, 0.5 equiv) were added to a round bottom flask containing a stirring solution of **S20** (80 mg, 0.43 mmol) at 0 °C in CH_2Cl_2. The reaction was allowed to warm up to room temperature and stirred overnight before quenching with saturated aqueous solution of NH_4Cl and water. The aqueous layer was extracted with CH_2Cl_2. The combined organic layer was washed with brine, dried over $MgSO_4$, filtered off the solids, and the filtrates were concentrated by rotary evaporation. Purification of the crude product by silica gel flash chromatography (pretreated with 2% triethylamine and hexanes/EtOAc 70:1) afforded the corresponding TBS-protected alkynyldiol **18** (122 mg, 68% yield from **S20**) as a clear colorless oil.

$[\alpha]^{22}_{D}$ 14.0 (c = 0.1, $CHCl_3$)

^1H NMR (400 MHz, C_6D_6) δ 4.68 (dd, *J* = 5.1, 2.2 Hz, 1H), 4.28 (ddd, *J* = 8.3, 5.0, 4.2 Hz, 1H), 4.07 (dd, *J* = 8.3, 5.1 Hz, 1H), 3.82 (dd, *J* = 10.5, 5.0 Hz, 1H), 3.64 (dd, *J* = 10.5, 4.2 Hz, 1H), 2.01 (d, *J* = 2.1 Hz, 1H), 1.62 (s, 3H), 1.27 (d, *J* = 0.7 Hz, 3H), 1.10 (s, 9H), 0.95 (s, 9H), 0.32 (s, 3H), 0.27 (s, 3H), 0.08 (s, 3H), 0.07 (s, 3H).

$^{13}C\{^1H\}$ NMR (101 MHz, C_6D_6) δ 109.9, 81.6, 80.2, 75.5, 74.2, 67.6, 66.3, 28.0, 26.4, 26.3 (3C), 26.2 (3C), 18.7, 18.6, -3.9, -4.3, -5.26, -5.30.

HRMS (ESI): *m/z* calcd for $C_{18}H_{39}O_4Si_2^+$ $[M – C_3H_6]^+$ 375.2381; found 375.2380.

[5,6-Di-*O*-(*tert*-butyldimethylsilyl)]-1,2-dideoxy-3,4-*O*-(isopropylidene)-(*E*)-1-iodo-D-*lyxo*-hex-1-enitol (**19**):

An oven-dried 25 mL flask with stir bar, cooled under argon, was charged with Cp_2ZrCl_2 (238 mg, 0.8 mmol, 2 equiv). The flask was vacuum pulled and re-filled with argon, and the cycle was repeated three times. After THF (1.3 mL, 5.5 mL / g of Cp_2ZrCl_2) was added, 1.0 M THF solution of $LiHBEt_3$ (a.k.a Super-hydride, 0.8 mL, 0.8 mmol, 2 equiv) was added dropwise into the stirring solution. The resulting white mixture was wrapped in aluminum foil and stirred at ambient temperature for 1 hr via syringe pump at 5 mL/hr. Alkyne **18** (167 mg, 0.4 mmol) in THF (0.4 mL, 1 M of alkyne) was added dropwise at 0 °C via syringe pump at 5 mL/hr. After 0.5 hr of stirring at 0 °C, the ice bath was removed, and the reaction mixture was stirred at ambient temperature for an additional 0.5 hr. Approximately 1 M solution of iodine (143 mg, 0.56 mmol, 1.4 equiv) in THF (0.56 mL) was added dropwise until the brown color persisted. After additional stirring for 0.5 hr, the reaction mixture was quenched with a solution of saturated aqueous solution of $NaHCO_3$ (50 mL) and extracted with diethyl ether (50 mL × 3). The combined organic layer was washed successively with saturated solution of $Na_2S_2O_3$ (50 mL), saturated brine, dried over $MgSO_4$, filtered, and the filtrate was concentrated by rotary evaporation. Purification of the crude product by silica gel flash chromatography (pre-treated with 2% triethylamine, hexanes/EtOAc 100:1 eluant) provided vinylic iodide (*E*)-**19** contaminated with terminal alkene byproduct, which was removed with a second column chromatography with silica gel pre-treated with 1% triethylamine, hexanes/CH_2Cl_2 7:1 eluant, to afford vinylic iodide (*E*)-**19** (124 mg, 57% yield).

$[\alpha]^{22}_{D}$ 10.6 (c = 0.1, $CHCl_3$)

^1H NMR (400 MHz, C_6D_6) δ 6.84 (dd, *J* = 14.5, 7.3 Hz, 1H), 6.16 (dd, *J* = 14.5, 1.0 Hz, 1H), 4.29 (ddd, *J* = 7.3, 6.2, 1.1 Hz, 1H), 4.17 (t, *J* = 6.5 Hz, 1H), 3.88 (dt, *J* = 6.8, 5.0 Hz, 1H), 3.68 (dd, *J* = 10.3, 5.3 Hz, 1H), 3.62 (dd, *J* = 10.4, 4.8 Hz, 1H), 1.41 (s, 3H), 1.24 (s, 3H), 1.04 (s, 9H), 0.96 (s, 9H), 0.21 (s, 3H), 0.18 (s, 3H), 0.08 (s, 3H), 0.07 (s, 3H).

$^{13}C\{^1H\}$ NMR (101 MHz, C_6D_6) δ 143.5, 108.6, 79.9, 79.7, 79.5, 73.2, 65.6, 27.8, 26.3 (3C), 26.2 (3C), 25.4, 18.6 (2C), -4.0, -4.1, -5.19, -5.22.

HRMS (ESI): *m/z* calcd for $C_{21}H_{43}IO_4Si_2{}^{35}Cl^-$ $[M + Cl]^-$ 577.1439; found 577.1452.

Vinylic iodide (*E*)-21 from D-ribose acetonide (20):

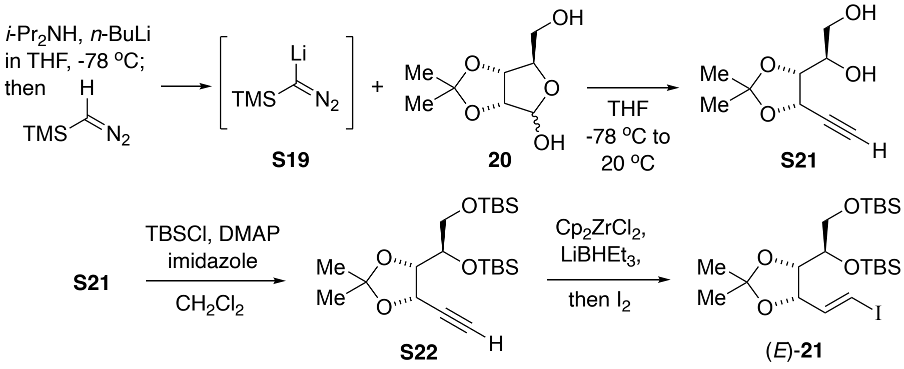

1,2-Dideoxy-3,4-*O*-(isopropylidene)-D-*ribo*-hex-1-ynitol (**S21**):

As described earlier, a solution of lithium trimethylsilyldiazomethane (**S19**) was prepared from *i*-Pr_2NH (3.5 mL, 25 mmol) in THF (18 mL), *n*-BuLi (2.5 M in hexanes, 10 mL, 25 mmol), and $TMSCHN_2$ (2.0 M in hexanes, 7.5 mL, 15 mmol).

(2,3-*O*-isopropylidene)-D-ribofuranose **20**[3,8] (0.95 g, 5.0 mmol) was dissolved in THF (6 mL), and this solution was slowly added to the -78 °C THF solution of **S19**. The resulting mixture was allowed to warm to room temperature overnight. The reaction mixture was quenched with saturated aqueous ammonium chloride (50 mL), and brine (20 mL) was added. The mixture was extracted with EtOAc (50 mL × 3). The organic layer was dried over anhydrous $MgSO_4$, filtered, and the filtrate was concentrated by rotary evaporation. The crude product **S21** was used in the next step without further purification.

For characterization of **S21**, a small portion of the crude product was purified by silica gel column chromatography, using a CH_2Cl_2/EtOAc (1:1) eluant.

^{1}H NMR (400 MHz, C_6D_6) δ 4.74 (dt, *J* = 5.6, 1.9 Hz, 1H, 4.01 (ddd, *J* = 8.7, 5.4, 3.2 Hz, 1H), 3.89 (dd, *J* = 8.7, 5.8 Hz, 1H), 3.72 (dd, *J* = 11.2, 3.4 Hz, 1H), 3.62 (dd, *J* = 11.1, 5.4 Hz, 1H), 2.15 (br s, 1H), 2.01 (s, 1H), 1.48 (s, 3H), 1.15 (s, 3H), 0.54 (br s, 1H).

^{13}C{^{1}H} NMR (101 MHz, C_6D_6) δ 110.7, 80.5, 77.7, 75.6, 71.3, 68.5, 64.1, 27.4, 25.8.

[5,6-Di-*O*-(*tert*-butyldimethylsilyl)]-1,2-dideoxy-3,4-*O*-(isopropylidene)-D-*ribo*-hex-1-ynitol (**S22**):

Imidazole (2.1 g, 31.1 mmol, 6 equiv), *t*-butyldimethylsilyl chloride (TBSCl, 2.4 g, 17 mmol, 3 equiv), and 4-(dimethylamino)pyridine (DMAP, 0.3 g, 2.5 mmol, 0.5 equiv) were added to a round bottom flask containing a stirring solution of **S21** (0.95 g, 5.0 mmol) at 0 °C in CH_2Cl_2 (25 mL, 0.2 M). The reaction was allowed to warm up to room temperature and stirred overnight before quenching with saturated aqueous solution of NH_4Cl and water. The aqueous layer was extracted with CH_2Cl_2. The combined organic layer was washed with brine, dried over $MgSO_4$, filtered off the solids, and the filtrates were concentrated by rotary evaporation. Purification of the crude product by silica gel flash chromatography (pretreated with 2% triethylamine and hexanes/EtOAc 70:1), followed by a second column chromatography with hexanes/CH_2Cl_2 (4:1) to remove a trace impurity afforded the corresponding TBS-protected alkynyldiol **S22** (798 mg, 39% yield from D-ribose acetonide **10**, 2 steps) as a clear colorless oil.

$[\alpha]^{21}{}_D$ -38.3 (c = 0.1, $CHCl_3$)

^1H NMR (400 MHz, C_6D_6) δ 4.86 (dd, *J* = 5.0, 2.1 Hz, 1H), 4.24 (ddd, *J* = 8.9, 4.4, 2.0 Hz, 1H), 4.14 (dd, *J* = 8.9, 5.0 Hz, 1H), 3.91 (dd, *J* = 10.8, 2.0 Hz, 1H), 3.78 (dd, *J* = 10.8, 4.4 Hz, 1H), 2.01 (d, *J* = 2.1 Hz, 1H), 1.60 (s, 3H), 1.28 (d, *J* = 0.7 Hz, 3H), 1.02 (s, 9H), 0.99 (s, 9H), 0.24 (s, 3H), 0.22 (s, 3H), 0.08 (s, 6H).

$^{13}C\{^1H\}$ NMR (101 MHz, C_6D_6) δ 110.9, 81.9, 77.9, 75.9, 74.3, 69.6, 65.8, 28.0, 26.8, 26.25 (3C), 26.21 (3C), 18.7, 18.5, -3.5, -4.4, -5.2, -5.3.

HRMS (ESI): *m/z* calcd for $C_{21}H_{42}O_4Si_2Na^+$ $[M + Na]^+$ 437.2514; found 437.2530.

[5,6-Di-*O*-(*tert*-butyldimethylsilyl)]-1,2-dideoxy-3,4-*O*-(isopropylidene)-(*E*)-1-iodo-D-*ribo*-hex-1-enitol ((*E*)-**21**):

An oven-dried 25 mL flask with stir bar, cooled under argon, was charged with Cp_2ZrCl_2 (585 mg, 2 mmol, 2 equiv). The flask was vacuum pulled and re-filled with argon, and the cycle was repeated three times. After THF (3.2 mL, 5.5 mL / g of Cp_2ZrCl_2) was added, 1.0 M THF solution of $LiHBEt_3$ (a.k.a Super-hydride, 2 mL, 2 mmol, 2 equiv) was added dropwise into the stirring solution. The resulting white mixture was wrapped in aluminum foil and stirred at ambient temperature for 1 hr. Alkyne **S22** (415 mg, 1 mmol) in THF (1 mL, 1M of alkyne) was added dropwise at 0 °C. After 0.5 hr of stirring at 0 °C, the ice bath was removed, and the reaction mixture was stirred at ambient temperature for an additional 0.5 hr. Approximately 1 M solution of iodine (356 mg, 1.4 mmol, 1.4 equiv) in THF (1.4 mL) was added dropwise until the brown color persisted. After additional stirring for 0.5 hr, the reaction mixture was quenched with a solution of saturated aqueous solution of $NaHCO_3$ (50 mL) and extracted with diethyl ether (50 mL × 3). The combined organic layer was washed successively with saturated solution of $Na_2S_2O_3$ (50 mL), saturated brine, dried over $MgSO_4$, filtered, and the filtrate was concentrated by rotary evaporation. Purification of the crude product by silica gel flash chromatography (pre-treated with 2% triethylamine, hexanes/EtOAc 100:1 eluant) provided vinylic iodide (*E*)-**21** (357 mg, containing 10% of the corresponding terminal alkene, 61% yield).

$[\alpha]^{21}{}_D$ -48.5 (c = 0.1, $CHCl_3$)

^1H NMR (400 MHz, C_6D_6) δ 6.87 (dd, *J* = 14.4, 6.5 Hz, 1H), 6.29 (dd, *J* = 14.4, 1.2 Hz, 1H), 4.44 (td, *J* = 6.3, 1.2 Hz, 1H), 4.25 (dd, *J* = 7.7, 6.1 Hz, 1H), 3.80 (dt, *J* = 7.7, 3.6 Hz, 1H), 3.71 (AB dd, *J* = 10.9, 3.1 Hz, 1H), 3.68 (AB dd, *J* = 10.9, 3.7 Hz, 1H), 1.35 (s, 3H), 1.24 (s, 3H), 0.99 (s, 9H), 0.97 (s, 9H), 0.19 (s, 3H), 0.18 (s, 3H), 0.06 (s, 6H).

$^{13}C\{^1H\}$ NMR (101 MHz, C_6D_6) δ 143.9, 108.7, 80.0, 79.3, 77.4, 72.8, 65.0, 28.0, 26.2 (3C), 26.1 (3C), 25.5, 18.6, 18.5, -3.5, -4.3, -5.2, -5.4.

HRMS (ESI): *m/z* calcd for $C_{21}H_{43}IO_4Si_2Na^+$ $[M + Na]^+$ 565.1637; found 565.1647.

Vinylic iodide (*E*)-22 from D-ribose acetonide (20):

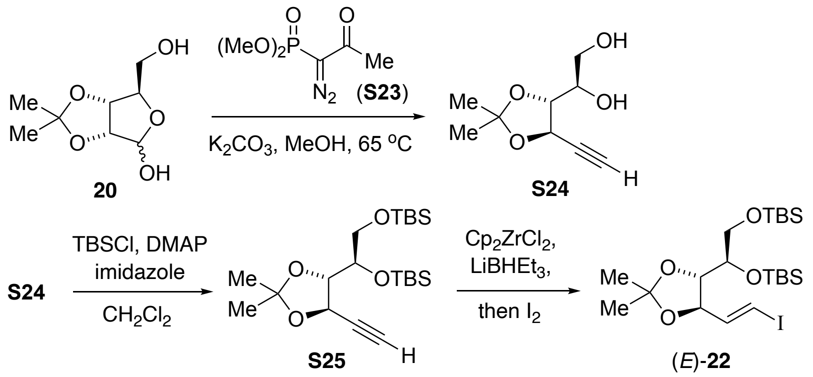

1,2-Dideoxy-3,4-*O*-(isopropylidene)-D-*arabino*-hex-1-ynitol (**S24**):

A two-neck round bottom flask with stir bar and reflux condenser was charged with a solution of **20** (1.5 g, 7.9 mmol) and K_2CO_3 (2.2 g, 16 mmol) in methanol (39 mL) under argon and heated to reflux in oil bath. A solution of Bestmann-Ohira reagent[9] (**S23**, 3.0 g, 16 mmol) dissolved in methanol (20 mL) was added dropwise via syringe pump for over 6 h. After stirring for additional 13 h, the reaction mixture was cooled to room temperature and neutralized with 1M HCl, and the precipitate formed was filtered on filter paper. The filtrate was extracted with EtOAc (× 3). The combined organic layers were dried with anhydrous $MgSO_4$, filtered, and the filtrate was concentrated by rotary evaporation. The crude product **S24** was used for the next step without further purification.

For characterization of **S24**, a small portion of the crude product was purified by silica gel column chromatography, using a CH_2Cl_2/EtOAc (1:1) eluant. The ^{1}H NMR spectrum of crude **S24** matches the previously reported data.[10]

^{1}H NMR (400 MHz, C_6D_6) δ 4.77 (dd, *J* = 6.3, 2.1 Hz, 1H), 4.11 (t, *J* = 6.1 Hz, 1H), 3.57 – 3.48 (m, 1H), 3.48 – 3.35 (m, 2H), 2.03 (d, *J* = 2.1 Hz, 1H), 1.42 (s, 3H), 1.26 (s, 3H).

^{13}C{^{1}H} NMR (101 MHz, C_6D_6) δ 110.9, 82.7, 82.5, 74.4, 72.2, 67.5, 63.4, 27.1, 26.1.

Comparing ^{1}H NMR assignments for acetonide-protected alkynyldiol isomers:

Chemical shifts for alkynyldiols **S20**, **S21**, and **S24** were measured as solutions in $CDCl_3$ (ppm). As previously reported for the corresponding benzylidene-protected compounds,[7] H5 chemical shifts for *lyxo*- and *ribo*-diastereomers **S20** and **S21** were deshielded relative to the *arabino*-diastereomer **S24**. In addition, acetonide methyl chemical shifts exhibited greater separation for *cis*-disubstituted acetonides **S20** and **S21**, relative to *trans*-disubstituted acetonide **S24**.

	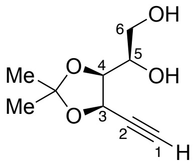 **S20**, *lyxo*		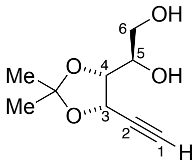 **S21**, *ribo*		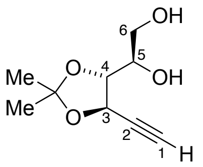 **S24**, *arabino*	
1	2.60 (d, *J* = 2.2 Hz)		2.65 (d, *J* = 2.2 Hz)		2.56 (d, *J* = 2.1 Hz)	
3	4.83 (dd, *J* = 6.0, 2.2 Hz)		4.93 (dd, *J* = 5.7, 2.2 Hz)		4.69 (d, *J* = 6.9, 2.3 Hz)	
4	4.19 (dd, *J* = 6.8, 6.0 Hz)		4.11 (dd, *J* = 8.7, 5.7 Hz)		4.15 (dd, *J* = 6.9, 5.0 Hz)	
5	4.06 (ddd, *J* = 6.8, 5.2, 3.6 Hz)		4.05 (ddd, *J* = 8.7, 5.3, 3.3 Hz)		3.92 (dt, *J* = 6.0, 4.5 Hz)	
6a	3.80 (dd, *J* = 11.6, 3.7 Hz)		3.90 (dd, *J* = 11.4, 3.3 Hz)		3.79 (m)	
6b	3.70 (dd, *J* = 11.6, 5.2 Hz)		3.78 (dd, *J* = 11.4, 5.3 Hz)		3.72 (dd, *J* = 11.5, 6.2 Hz)	
Me	1.57	Δ 0.19 ppm	1.52	Δ 0.17 ppm	1.51	Δ 0.07 ppm
Me	1.38		1.35		1.44	

[5,6-Di-*O*-(*tert*-butyldimethylsilyl)]-1,2-dideoxy-3,4-*O*-(isopropylidene)-D-*arabino*-hex-1-ynitol (**S25**):

Imidazole (1.09 g, 16 mmol, 6 equiv), *t*-butyldimethylsilyl chloride (TBSCl, 1.21 g, 8.1 mmol, 3 equiv), and 4-(dimethylamino)pyridine (DMAP, 0.16 g, 1.3 mmol, 0.5 equiv) were added to a round bottom flask containing a stirring solution of **S24** (0.50 g, 2.7 mmol) at 0 °C in CH_2Cl_2. The reaction was allowed to warm up to room temperature and stirred overnight before quenching with saturated aqueous solution of NH_4Cl and water. The aqueous layer was extracted with CH_2Cl_2. The combined organic layer was washed with brine, dried over $MgSO_4$, filtered off the solids, and the filtrates were concentrated by rotary evaporation. Purification of the crude product by silica gel flash chromatography (pretreated with 2% triethylamine and hexanes/EtOAc 70:1), followed by a second column chromatography with hexanes/EtOAc (100:1, gradient elution to 20:1) to remove a trace impurity afforded the corresponding TBS-protected alkynyldiol **S25** (338 mg, 23% yield from D-ribose acetonide **20**, 2 steps) as a clear colorless oil.

$[\alpha]^{22}_{D}$ -12.9 (c = 0.1, $CHCl_3$)

^{1}H NMR (400 MHz, C_6D_6) δ 4.98 (dd, *J* = 6.7, 2.1 Hz, 1H), 4.48 (dd, *J* = 6.7, 4.0 Hz, 1H), 4.01 (td, *J* = 5.5, 4.0 Hz, 1H), 3.77 (dd, *J* = 10.4, 5.5 Hz, 1H), 3.67 (dd, *J* = 10.4, 5.4 Hz, 1H), 2.07 (d, *J* = 2.1 Hz, 1H), 1.48 (s, 3H), 1.41 (s, 3H), 0.99 (s, 9H), 0.97 (s, 9H), 0.15 (s, 3H), 0.14 (s, 3H), 0.07 (s, 6H).

^{13}C{^{1}H} NMR (101 MHz, C_6D_6) δ 110.4, 83.7, 82.6, 74.2, 73.6, 66.4, 65.5, 27.0, 26.2 (3C), 26.1 (3C), 25.8, 18.6, 18.4, -4.1, -4.5, -5.3 (2C).

HRMS (ESI): *m/z* calcd for $C_{18}H_{39}O_4Si_2^+$ $[M – C_3H_6]^+$ 375.2381; found 375.2379.

[5,6-Di-*O*-(*tert*-butyldimethylsilyl)]-1,2-dideoxy-3,4-*O*-(isopropylidene)-(*E*)-1-iodo-D-*arabino*-hex-1-enitol ((*E*)-**22**):

An oven-dried 25 mL flask with stir bar, cooled under argon, was charged with Cp_2ZrCl_2 (423 mg, 1.4 mmol, 2 equiv). The flask was vacuum pulled and re-filled with argon, and the cycle was repeated three times. After THF (2.3 mL, 5.5 mL / g of Cp_2ZrCl_2) was added, 1.0 M THF solution of $LiHBEt_3$ (a.k.a Super-hydride, 1.4 mL, 1.4 mmol, 2 equiv) was added dropwise into the stirring solution. The resulting white mixture was wrapped in aluminum foil and stirred at ambient temperature for 1 hr. Alkyne **S25** (298 mg, 0.7 mmol) in THF (0.7 mL, 1 M of alkyne) was added dropwise at 0 °C. After 0.5 hr of stirring at 0 °C, the ice bath was removed, and the reaction mixture was stirred at ambient temperature for an additional 0.5 hr. Approximately 1 M solution of iodine (257 mg, 1 mmol, 1.4 equiv) in THF (1 mL) was added dropwise until the brown color persisted. After additional stirring for 0.5 hr, the reaction mixture was quenched with a solution of saturated aqueous solution of $NaHCO_3$ (50 mL) and extracted with diethyl ether (50 mL × 3). The combined organic layer was washed successively with saturated solution of $Na_2S_2O_3$ (50 mL), saturated brine, dried over $MgSO_4$, filtered, and the filtrate was concentrated by rotary evaporation. Purification of the crude product by silica gel flash chromatography (pre-treated with 2% triethylamine, hexanes/EtOAc 100:1 eluant) provided vinylic iodide (*E*)-**22** (296 mg, containing 16% of the corresponding terminal alkene, 67% yield). A portion of this material was subjected to second column chromatography to provide a sample for analysis.

$[\alpha]^{22}_{D}$ -4.8 (c = 0.1, $CHCl_3$)

^1H NMR (400 MHz, C_6D_6) δ 6.76 (dd, *J* = 14.5, 5.8 Hz, 1H), 6.38 (dd, *J* = 14.5, 1.3 Hz, 1H), 4.53 (ddd, *J* = 7.2, 5.9, 1.3 Hz, 1H), 4.04 – 3.95 (m, 2H), 3.64 – 3.59 (m, 1H), 3.59 – 3.54 (m, 1H), 1.41 (s, 3H), 1.25 (s, 3H), 0.99 (s, 9H), 0.95 (s, 9H), 0.13 (s, 3H), 0.12 (s, 3H), 0.06 (s, 3H), 0.05 (s, 3H).

$^{13}C\{^1H\}$ NMR (101 MHz, C_6D_6) δ 145.4, 109.1, 80.3, 79.2, 79.1, 73.5, 65.3, 27.2, 26.7, 26.2 (3C), 26.1 (3C), 18.6, 18.4, -4.2, -4.3, -5.2, -5.3.

HRMS (APCI): *m/z* calcd for $C_{21}H_{43}IO_4Si_2{}^{35}Cl^-$ $[M + Cl]^-$ 577.1439; found 577.1445.

.

C-O cross-coupling to synthesize vinylic ether (*E*)-10:

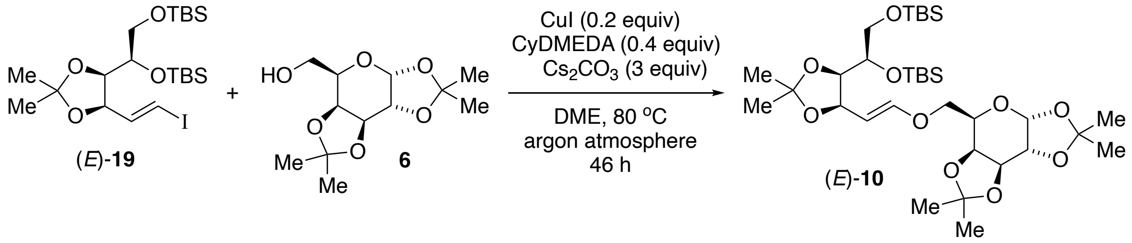

1,2:3,4-Di-*O*-isopropylidene-6-*O*-(*E*)-[5,6-di-*O*-(*tert*-butyldimethylsilyl)]-1,2-dideoxy-3,4-*O*-(isopropylidene)-D-*lyxo*-hex-1-enitol]-α-D-galactopyranose ((*E*)-**10**):

An oven-dried 4 mL vial with a stir bar was charged with 1,2:3,4-di-O-isopropylidene-α-D-galactopyranose (**6**, 156 mg, 0.6 mmol, 2 equiv), Cs_2CO_3 (294 mg, 0.9 mmol, 3 equiv), *trans-N,N'*-dimethylcyclohexane-1,2-diamine (CyDMEDA, 18.9 µL, 0.12 mmol, 0.4 equiv), and vinylic iodide (*E*)-**19** (163 mg, 0.3 mmol, 1 equiv). The reaction vial was purged continuously with argon for 5 min before CuI (12 mg, 0.06 mmol, 0.2 equiv) was added. Anhydrous 1,2-dimethoxyethane (DME, 0.43 mL, 0.7 M based on vinylic iodide) was added, and the reaction mixture was bubbled with argon for 5 minutes. The reaction vial was quickly closed with a solid cap, sealed with Teflon tape and electrical tape, and heated at an internal temperature at 75 °C in an oil bath. The reaction mixture was stirred for 42 hours, cooled to room temperature, and diluted with EtOAc. The mixture was filtered through a Celite® pad and rinsed with EtOAc (100 mL), and the filtrate was concentrated by rotary evaporation. Purification of the crude product by silica gel flash chromatography (pre-treated with 2% triethylamine, hexanes / EtOAc 12:1 eluent) afforded the vinylic ether (*E*)-**10** (92 mg, 45% yield from compound (*E*)-**19**).

$[\alpha]^{22}_{D}$ -7.60 (c = 0.1, $CHCl_3$)

^{1}H NMR (800 MHz, C_6D_6) δ 6.41 (d, *J* = 12.6 Hz, 1H), 5.47 (d, *J* = 5.0 Hz, 1H), 5.19 (dd, *J* = 12.6, 9.6 Hz, 1H), 4.45 (dd, *J* = 7.9, 2.4 Hz, 1H), 4.37 (dd, *J* = 9.6, 6.1 Hz, 1H), 4.21 (apparent t, *J* = 6.4 Hz, 1H), 4.19 (td, *J* = 6.2, 2.0 Hz, 1H), 4.14 (dd, *J* = 5.0, 2.4 Hz, 1H), 4.04 (dd, *J* = 10.1, 5.6 Hz, 1H), 4.01 (dd, *J* = 7.5, 1.7 Hz, 1H), 4.00 – 3.98 (m, 1H), 3.91 (ddd, *J* = 6.8, 5.3, 4.0 Hz, 1H), 3.70 (dd, *J* = 10.3, 5.4 Hz, 1H), 3.68 (dd, *J* = 10.3, 4.0 Hz, 1H), 1.52 (s, 3H), 1.44 (s, 3H), 1.42 (s, 3H), 1.33 (s, 3H), 1.13 (s, 3H), 1.10 (s, 9H), 1.02 (d, *J* = 1.0 Hz, 3H), 0.98 (s, 9H), 0.28 (s, 3H), 0.24 (s, 3H), 0.12 (s, 3H), 0.09 (s, 3H).

$^{13}C\{^1H\}$ NMR (101 MHz, C_6D_6) δ 150.8, 109.4, 108.5, 107.7, 101.8, 96.8, 79.0, 76.4, 74.0, 71.4, 71.2, 70.9, 68.9, 66.9, 65.6, 28.3, 26.4 (3C), 26.3 (3C), 26.2 (2C), 25.8, 24.8, 24.4, 18.8, 18.7, -3.7, -4.0, -5.1, -5.2.

HRMS (APCI): *m/z* calcd for $C_{33}H_{63}O_{10}Si_2^+$ $[M + H]^+$ 675.3954, found 675.3966.

Subsequent analysis of the crude ^{1}H NMR spectrum indicated approximately 2% of an (*E*)-enyne **S26** was formed, based on resonances observed at 6.45 ppm and 5.80 ppm.

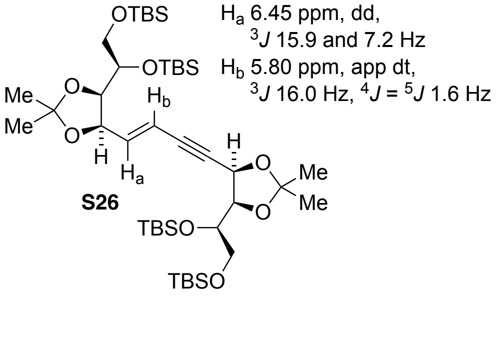

Assignments of diagnostic ^{13}C and ^{1}H NMR resonances for vinylic ether (*E*)-**10**, determined by HSQC, COSY, and HMBC spectroscopy:

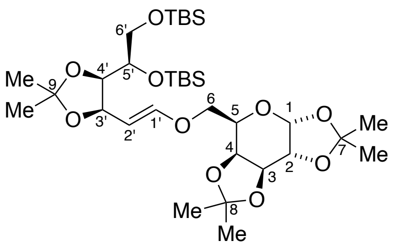

carbon position	δ_C[a]	δ_H[b,c] (*J*, Hz)
1'	150.8	6.41, d (12.6)
2'	101.8	5.19, dd (12.6, 9.6)
3'	76.4	4.37, dd (9.6, 6.1)
4'	79.0	4.21, t (6.4)
5'	74.0	3.91, ddd (6.8, 5.3, 4.0)
6a'	65.6	3.70, dd (10.3, 5.4)
6b'		3.68, dd (10.3, 4.0)
1	96.8	5.47, d (5.0)
2	70.9	4.14, dd (5.0, 2.4)
3	71.2	4.45, dd (7.9, 2.4)
4	71.4	4.00, overlapping m[d]
5	66.9	4.19, td (6.2, 2.0)
6a	68.9	4.04, dd (10.1, 5.6)
6b		3.99, dd (ca. 10, 7)[d]
7	108.5	--
8	109.4	--
9	107.7	--

[a] Recorded at 101 MHz in C_6D_6
[b] Recorded at 800 MHz in C_6D_6
[c] Denotes ^{1}H resonance(s) correlating to each ^{13}C for the carbon position
[d] Due to overlapping ^{1}H peaks, these assignments are tentative.

C-O cross-coupling to synthesize vinylic ether (*E*)-23:

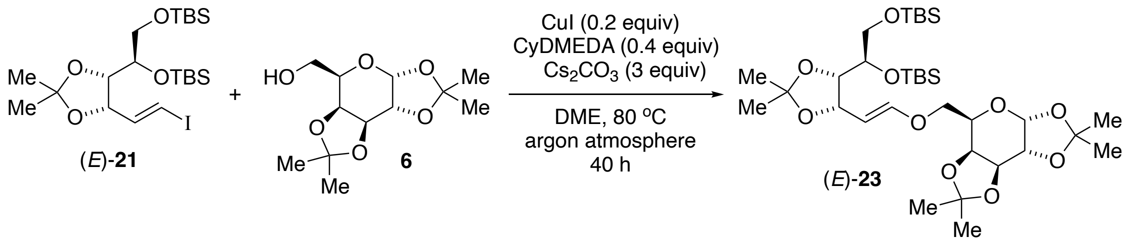

1,2:3,4-Di-*O*-isopropylidene-6-*O*-(*E*)-[5,6-di-*O*-(*tert*-butyldimethylsilyl)]-1,2-dideoxy-3,4-*O*-(isopropylidene)-D-*ribo*-hex-1-enitol]-α-D-galactopyranose ((*E*)-**23**):

An oven-dried 4 mL vial with a stir bar was charged with 1,2:3,4-di-O-isopropylidene-α-D-galactopyranose (**6**, 261 mg, 1 mmol, 2 equiv), Cs_2CO_3 (490 mg, 1.5 mmol, 3 equiv), *trans-N,N'*-dimethylcyclohexane-1,2-diamine (CyDMEDA, 31.6 µL, 0.2 mmol, 0.4 equiv), and vinylic iodide (*E*)-**21** (272 mg, containing 9% terminal alkene, 0.46 mmol, 1 equiv). The reaction vial was purged continuously with argon for 5 min before CuI (19 mg, 0.1 mmol, 0.2 equiv) was added. Anhydrous 1,2-dimethoxyethane (DME, 0.7 mL, 0.7 M based on vinylic iodide) was added, and the reaction mixture was bubbled with argon for 5 minutes. The reaction vial was quickly closed with a solid cap, sealed with Teflon tape and electrical tape, and heated at an internal temperature at 75 °C in an oil bath. The reaction mixture was stirred for 46 hours, cooled to room temperature, and diluted with EtOAc. The mixture was filtered through a Celite® pad and rinsed with EtOAc (100 mL), and the filtrate was concentrated by rotary evaporation. Purification of the crude product by silica gel flash chromatography (pre-treated with 2% triethylamine, hexanes / EtOAc 12:1 eluent) afforded the vinylic ether (*E*)-**23** (162 mg, 52% yield).

A mixture of terminal alkene impurity **S27** and enyne **S28** were obtained from less polar fractions (67 mg, **S27** : **S28** ratio = 1.5 : 1).

Data for (*E*)-**23**:

$[\alpha]^{22}_D$ -72.1 (c = 0.1, $CHCl_3$)

^{1}H NMR (800 MHz, C_6D_6) δ 6.53 (d, *J* = 12.7 Hz, 1H), 5.48 (d, *J* = 5.0 Hz, 1H), 5.21 (dd, *J* = 12.6, 9.0 Hz, 1H), 4.61 (dd, *J* = 9.0, 6.0 Hz, 1H), 4.47 (dd, *J* = 7.9, 2.4 Hz, 1H), 4.33 (dd, *J* = 7.5, 6.0 Hz, 1H), 4.23 (td, *J* = 6.4, 1.9 Hz, 1H), 4.15 (dd, *J* = 5.0, 2.4 Hz, 1H), 4.09 (dd, *J* = 8.0, 1.8 Hz, 1H), 4.07 (dd, *J* = 9.8, 6.7 Hz, 1H), 4.02 (dd, *J* = 9.8, 6.0 Hz, 1H), 3.93 (ddd, *J* = 7.3, 4.2, 2.9 Hz, 1H), 3.88 (dd, *J* = 10.8, 2.9 Hz, 1H), 3.82 (dd, *J* = 10.8, 4.2 Hz, 1H), 1.463 (s, 3H), 1.460 (s, 3H), 1.43 (s, 3H), 1.33 (s, 3H), 1.15 (s, 3H), 1.04 (s, 3H), 1.03 (s, 9H), 1.00 (s, 9H), 0.23 (s, 3H), 0.20 (s, 3H), 0.12 (s, 3H), 0.11 (s, 3H).

$^{13}C\{^1H\}$ NMR (101 MHz, C_6D_6) δ δ 150.5, 109.2, 108.5, 107.9, 101.9, 96.8, 77.7, 77.0, 73.3, 71.2, 71.13, 71.05, 68.4, 66.7, 65.5, 28.4, 26.32 (3C), 26.28, 26.27, 26.23 (3C), 25.9, 24.9, 24.4, 18.7, 18.5, -3.4, -4.3, -5.15, -5.25.

HRMS (ESI): *m/z* calcd for $C_{33}H_{62}O_{10}Si_2Na^+$ $[M + Na]^+$ 697.3774, found 697.3806.

Assignments of diagnostic ^{13}C and ^{1}H NMR resonances for vinylic ether (*E*)-**23**, determined by HSQC, COSY, and HMBC spectroscopy:

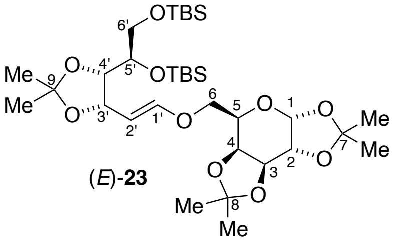

(*E*)-**23**

carbon position	δ_C[a]	δ_H[b,c] (*J*, Hz)
1'	150.5	6.53, d (12.7)
2'	101.9	5.21, dd (12.6, 9.0)
3'	77.0	4.61, dd (9.0, 6.0)
4'	77.7	4.33, dd (7.5, 6.0)
5'	73.3	3.93, ddd (7.3, 4.2, 2.9)
6a'	65.5	3.88, dd (10.8, 2.9)
6b'		3.82, dd (10.8, 4.2)
1	96.8	5.48, d (5.0)
2	71.05	4.15, dd (5.0, 2.4)
3	71.13[d]	4.47, dd (7.9, 2.4)
4	71.2[d]	4.09, dd (8.0, 1.8)
5	66.7	4.23, td (6.4, 1.9)
6a	68.4	4.07, dd (9.8, 6.7)
6b		4.02, dd (9.8, 6.0)
7	108.5	--
8	109.2	--
9	107.9	--

[a] Recorded at 101 MHz in C_6D_6
[b] Recorded at 800 MHz in C_6D_6
[c] Denotes ^{1}H resonance(s) correlating to each ^{13}C for the carbon position
[d] Due to overlapping ^{13}C peaks, these assignments are tentative.

Diagnostic resonances for terminal alkene **S27** and enyne **S28**:

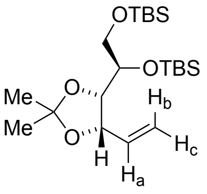

S27

H_a 6.12 ppm, ddd, ^{3}J 17.1, 10.4, 6.5 Hz
H_b 5.42 ppm, ddd, ^{3}J 17.2 Hz, ^{2}J and ^{4}J 2.1, 1.4 Hz
H_c 5.16 ppm, ddd, ^{3}J 10.4 Hz, ^{2}J and ^{4}J 2.0, 1.2 Hz

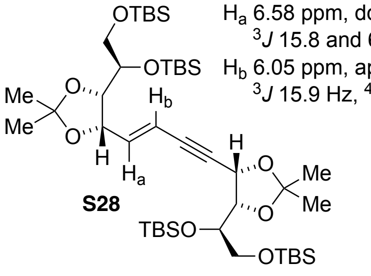

S28

H_a 6.58 ppm, dd, ^{3}J 15.8 and 6.1 Hz
H_b 6.05 ppm, app dt, ^{3}J 15.9 Hz, ^{4}J = ^{5}J 1.7 Hz

C-O cross-coupling to synthesize vinylic ether (*E*)-24:

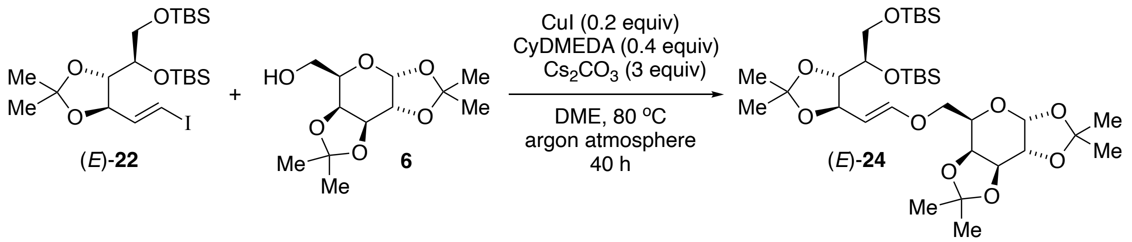

1,2:3,4-Di-*O*-isopropylidene-6-*O*-(*E*)-[5,6-di-*O*-(*tert*-butyldimethylsilyl)]-1,2-dideoxy-3,4-*O*-(isopropylidene)-D-*arabino*-hex-1-enitol]-α-D-galactopyranose ((*E*)-**24**):

An oven-dried 4 mL vial with a stir bar was charged with 1,2:3,4-di-O-isopropylidene-α-D-galactopyranose (**6**, 182 mg, 0.7 mmol, 2 equiv), Cs_2CO_3 (342 mg, 1.1 mmol, 3 equiv), *trans-N,N'*-dimethylcyclohexane-1,2-diamine (CyDMEDA, 22.1 µL, 0.14 mmol, 0.4 equiv), and vinylic iodide (*E*)-**22** (190 mg, containing 16% of the terminal alkene, 0.31 mmol, 1 equiv). The reaction vial was purged continuously with argon for 5 min before CuI (13 mg, 0.07 mmol, 0.2 equiv) was added. Anhydrous 1,2-dimethoxyethane (DME, 0.5 mL, 0.7 M based on vinylic iodide) was added, and the reaction mixture was bubbled with argon for 5 minutes. The reaction vial was quickly closed with a solid cap, sealed with Teflon tape and electrical tape, and heated at an internal temperature at 75 °C in an oil bath. The reaction mixture was stirred for 42 hours, cooled to room temperature, and diluted with EtOAc. The mixture was filtered through a Celite® pad and rinsed with EtOAc (100 mL), and the filtrate was concentrated by rotary evaporation. Purification of the crude product by silica gel flash chromatography (pre-treated with 2% triethylamine, hexanes / EtOAc 12:1 eluent) afforded the vinylic ether (*E*)-**24** (155 mg, 74% yield).

$[\alpha]^{22}_{D}$ -18.1 (c = 0.1, $CHCl_3$)

^{1}H NMR (800 MHz, C_6D_6) δ 6.64 (d, *J* = 12.6 Hz, 1H), 5.48 (d, *J* = 5.0 Hz, 1H), 5.05 (dd, *J* = 12.6, 8.4 Hz, 1H), 4.68 (t, *J* = 8.2 Hz, 1H), 4.47 (dd, *J* = 7.9, 2.4 Hz, 1H), 4.20 (td, *J* = 6.1, 1.9 Hz, 1H), 4.16 – 4.15 (m, 1H), 4.15 – 4.13 (m, 1H), 4.06 (dd, *J* = 8.1, 3.4 Hz, 1H), 4.03 – 4.01 (m, 1H), 4.01 – 3.98 (m, 2H), 3.78 (dd, *J* = 10.2, 6.2 Hz, 1H), 3.71 (dd, *J* = 10.2, 5.8 Hz, 1H), 1.48 (s, 3H), 1.45 (s, 3H), 1.43 (s, 3H), 1.41 (s, 3H), 1.15 (s, 3H), 1.03 (s, 3H), 1.02 (s, 9H), 0.97 (s, 9H), 0.20 (s, 3H), 0.17 (s, 3H), 0.09 (s, 3H), 0.08 (s, 3H).

$^{13}C\{^1H\}$ NMR (101 MHz, C_6D_6) δ 150.6, 109.3, 108.5, 108.0, 103.5, 96.8, 81.6, 75.6, 73.4, 71.4, 71.2, 71.0, 68.6, 66.8, 65.4, 27.6, 27.4, 26.3, 26.2 (7C), 24.9, 24.4, 18.6, 18.5, -4.16, -4.18, -5.2 (2C).

HRMS (ESI): *m/z* calcd for $C_{33}H_{62}O_{10}Si_2{}^{35}Cl^-$ $[M + Cl]^-$ 709.3576, found 709.3561.

Assignments of diagnostic ^{13}C and ^{1}H NMR resonances for vinylic ether (*E*)-**24**, determined by HSQC, COSY, and HMBC spectroscopy:

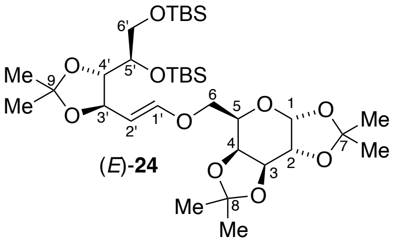

(*E*)-**24**

carbon position	δ_C[a]	δ_H[b,c] (*J*, Hz)
1'	150.6	6.64, d (12.6)
2'	103.5	5.05, dd (12.6, 8.4)
3'	75.6	4.68, t (8.2)
4'	81.6	4.06, dd (8.1, 3.4)
5'	73.4	4.15 – 4.13,[d] m
6a'	65.4	3.78, dd (10.2, 6.2)
6b'		3.71, dd (10.2, 5.8)
1	96.8	5.48, d (5.0)
2	71.0	4.16 – 4.15,[d] m
3	71.2	4.47, dd (7.9, 2.4)
4	71.4	4.03 – 4.01,[d] m
5	66.8	4.20, td (6.1, 1.9)
6a	68.6	4.01 – 3.98,[d] m (2H)
6b		
7	108.5	--
8	109.3	--
9	108.0	--

[a] Recorded at 101 MHz in C_6D_6
[b] Recorded at 800 MHz in C_6D_6
[c] Denotes ^{1}H resonance(s) correlating to each ^{13}C for the carbon position
[d] Due to overlapping ^{1}H peaks, these assignments are tentative.

Deprotection of silyl ethers from (*E*)-10 to diol-vinylic ether (*E*)-12:

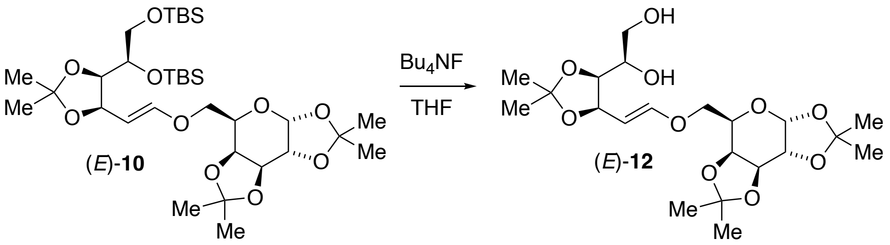

1,2:3,4-Di-*O*-isopropylidene-6-*O*-(*E*)-[1,2-dideoxy-3,4-*O*-(isopropylidene)-D-*lyxo*-hex-1-enitol]-α-D-galactopyranose ((*E*)-**12**):

An oven-dried 50 mL round-bottom flask with stir bar was charged with vinylic ether (*E*)-**10** (150 mg, 0.22 mmol), dissolved in anhydrous THF (21 mL, 0.01 M), and cooled to 0 °C with an ice bath. A solution of tetrabutylammonium fluoride (TBAF, 1.0 M in THF, 0.75 mL, 0.75 mmol, 3.5 equiv) was added slowly. The ice bath was removed, and the reaction mixture stirred for 5 h at room temperature. The reaction mixture was cooled to 0 °C and quenched by slow addition of saturated aqueous NH_4Cl solution at room temperature, and then diluting with deionized water and brine. The aqueous layer was extracted with EtOAc (x 3) and the combined organic layers were dried over $MgSO_4$, filtered, and the filtrate was concentrated by rotary evaporation. The crude product was purified by silica gel flash chromatography (hexanes/EtOAc 3:7 → 1:5 eluant) to give the diol-vinylic ether (*E*)-**12** (83 mg, 55% yield).

$[\alpha]^{23}_{D}$ -49.1 (c = 0.1, $CHCl_3$)

^{1}H NMR (800 MHz, C_6D_6) δ 6.38 (d, *J* = 12.7 Hz, 1H), 5.49 (d, *J* = 5.0 Hz, 1H), 5.25 (dd, *J* = 12.7, 9.4 Hz, 1H), 4.45 (dd, *J* = 7.9, 2.4 Hz, 1H), 4.28 (dd, *J* = 9.4, 7.0 Hz, 1H), 4.21 (ddd, *J* = 7.0, 5.4, 1.9 Hz, 1H), 4.15 (dd, *J* = 5.0, 2.4 Hz, 1H), 4.07 (dd, *J* = 10.3, 6.8 Hz, 1H), 4.02 (dd, *J* = 10.3, 5.5 Hz, 1H), 3.97 (dd, *J* = 7.9, 1.9 Hz, 1H), 3.79 (dd, *J* = 7.0, 3.4 Hz, 1H), 3.51 (d, *J* = 4.2 Hz, 2H), 3.50 – 3.48 (m, 1H), 2.32 (br, 1H), 1.87 (br, 1H), 1.43 (s, 3H), 1.42 (s, 3H), 1.38 (s, 3H), 1.20 (s, 3H), 1.13 (s, 3H), 1.03 (s, 3H).

^{13}C{^{1}H} NMR (201 MHz, C_6D_6) δ 151.2, 109.4, 108.6, 107.9, 100.5, 96.8, 78.0, 76.8, 71.3, 71.2, 70.9, 70.3, 68.8, 66.9, 64.7, 27.3, 26.23, 26.19, 24.9, 24.8, 24.3.

HRMS (ESI negative): *m/z* calcd for $C_{21}H_{34}O_{10}\,^{35}Cl^-$ $[M + Cl]^-$ 481.1846, found 481.1853.

Deprotection of silyl ethers from (*E*)-23 to diol-vinylic ether (*E*)-25:

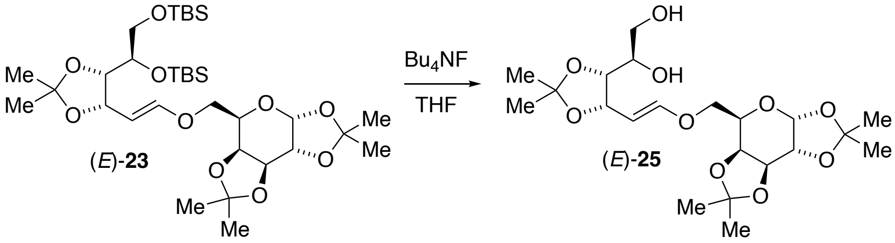

1,2:3,4-Di-*O*-isopropylidene-6-*O*-(*E*)-[1,2-dideoxy-3,4-*O*-(isopropylidene)-D-*ribo*-hex-1-enitol]-α-D-galactopyranose ((*E*)-**25**):

A 50 mL round-bottom flask with stir bar was charged with vinylic ether (*E*)-**23** (140 mg, 0.2 mmol), dissolved in anhydrous THF (19 mL, 0.01 M), and cooled to 0 °C with an external cooling bath. A solution of tetrabutylammonium fluoride (TBAF, 1.0 M in THF, 0.7 mL, 0.7 mmol, 3.5 equiv) was added slowly. The external cooling bath was removed, and the reaction mixture stirred for 5 h at room temperature. The reaction mixture was cooled to 0 °C, and quenched by slow addition of a saturated aqueous NH_4Cl solution at room temperature, and then diluting with deionized water and brine. The aqueous layer was extracted with EtOAc (x 3) and the combined organic layers were dried over $MgSO_4$, filtered, and the filtrate was concentrated by rotary evaporation. The crude product was purified by silica gel flash chromatography (pre-treated with 2% triethylamine, hexanes/EtOAc 1:3 → 1:5 eluant) to give the diol-vinylic ether (*E*)-**25** (61 mg, 65% yield).

$[\alpha]^{22}_{D}$ -30.6 (c = 0.1, $CHCl_3$)

^{1}H NMR (800 MHz, C_6D_6) δ 6.46 (d, *J* = 12.4 Hz, 1H), 5.44 (d, *J* = 5.0 Hz, 1H), 5.20 (dd, *J* = 12.5, 9.0 Hz, 1H), 4.59 (dd, *J* = 8.9, 5.9 Hz, 1H), 4.41 (dd, *J* = 8.0, 2.4 Hz, 1H), 4.13 - 4.10 (m, 2H), 4.07 (dd, *J* = 8.7, 6.0 Hz, 1H), 3.98 (d, *J* = 5.9 Hz, 2H), 3.92 (dt, *J* = 7.5, 1.9 Hz, 1H), 3.88 (dd, *J* = 7.9, 1.9 Hz, 1H), 3.84 – 3.75 (m, 2H), 2.9 (br, 1H), 2.4 (br, 1H), 1.45 (s, 3H), 1.43 (s, 3H), 1.39 (s, 3H), 1.26 (s, 3H), 1.12 (s, 3H), 1.04 (s, 3H).

^{13}C{^{1}H} NMR (101 MHz, C_6D_6) δ 150.8, 109.5, 108.8, 108.6, 101.6, 96.7, 78.6, 77.0, 71.4, 71.1, 70.9, 70.3, 69.6, 67.7, 65.2, 28.3, 26.20, 26.17, 25.7, 24.8, 24.3.

HRMS (ESI negative): *m/z* calcd for $C_{21}H_{34}O_{10}{}^{35}Cl$ $[M + Cl]^-$ 481.1846, found 481.1858.

Deprotection of silyl ethers from (*E*)-24 to diol-vinylic ether (*E*)-26:

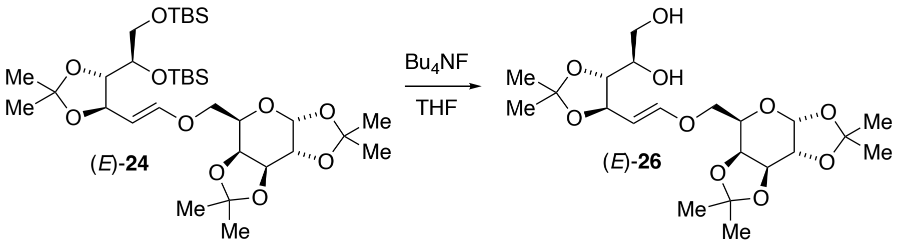

1,2:3,4-Di-*O*-isopropylidene-6-*O*-(*E*)-[1,2-dideoxy-3,4-*O*-(isopropylidene)-D-*arabino*-hex-1-enitol]-α-D-galactopyranose ((*E*)-**26**):

An oven-dried 50 mL round-bottom flask with stir bar was charged with vinylic ether (*E*)-**24** (50 mg, 0.074 mmol), dissolved in anhydrous THF (7 mL, 0.01 M), and cooled to 0 °C with an ice bath. A solution of tetrabutylammonium fluoride (TBAF, 1.0 M in THF, 0.25 mL, 0.25 mmol, 3.4 equiv) was added slowly. The ice bath was removed, and the reaction mixture stirred for 5 h at room temperature. The reaction mixture was cooled to 0 °C and quenched by slow addition of saturated aqueous NH_4Cl solution at room temperature, and then diluting with deionized water and brine. The aqueous layer was extracted with EtOAc (x 3) and the combined organic layers were dried over $MgSO_4$, filtered, and the filtrate was concentrated by rotary evaporation. The crude product was purified by silica gel flash chromatography (hexanes/EtOAc 1:3 eluant) to give the diol-vinylic ether (*E*)-**26** (23 mg, 71% yield).

$[\alpha]^{23}_{D}$ -49.1 (c = 0.1, $CHCl_3$)

^{1}H NMR (400 MHz, C_6D_6) δ 6.54 (d, *J* = 12.6 Hz, 1H), 5.48 (d, *J* = 5.0 Hz, 1H), 4.89 (dd, *J* = 12.6, 8.5 Hz, 1H), 4.46 (dd, *J* = 7.9, 2.4 Hz, 1H), 4.34 (t, *J* = 8.1 Hz, 1H), 4.20 (td, *J* = 6.1, 1.9 Hz, 1H), 4.15 (dd, *J* = 5.0, 2.4 Hz, 1H), 4.01 (dd, *J* = 10.3, 6.5 Hz, 1H), 3.99 – 3.94 (m, 2H), 3.68 (app q, *J* = 5.1 Hz, 1H), 3.62 (dd, *J* = 7.9, 5.3 Hz, 1H), 3.54 (app d, *J* = 5.0 Hz, 2H), 2.4 (br, 1H), 2.1 (br, 1H), 1.43 (app s, 6H), 1.34 (s, 3H), 1.32 (s, 3H), 1.13 (s, 3H), 1.02 (m, 3H).

^{13}C{^{1}H} NMR (101 MHz, C_6D_6) δ 150.5, 109.0, 108.24, 108.16, 102.4, 96.4, 81.5, 77.0, 72.2, 71.0, 70.8, 70.6, 68.3, 66.5, 63.5, 27.0, 26.8, 25.88, 25.85, 24.5, 24.0.

HRMS (ESI negative): *m/z* calcd for $C_{21}H_{34}O_{10}$ $^{35}Cl^-$ $[M + Cl]^-$ 481.1846, found 481.1853.

Epoxidation-oxacyclization of (*E*)-12 to disaccharide 15 and diacetate derivative 27:

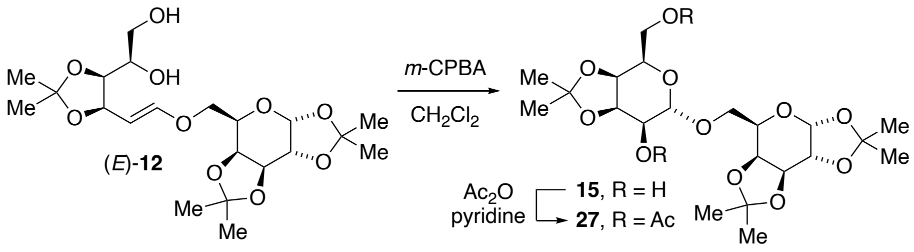

1,2:3,4-Di-*O*-(isopropylidene)-6-*O*-[3,4-*O*-(isopropylidene)-α-D-talopyranosyl]-α-D-galactopyranose (**15**):

A 10 mL round-bottom flask with stir bar was charged with diol-vinylic ether (*E*)-**12** (71 mg, 0.16 mmol) and CH_2Cl_2 (2.5 mL, 0.06 M), and cooled to 0 °C with an external cooling bath. *m*-CPBA (77% w/w) (40 mg, 0.23 mmol, 1.1 equiv) was slowly added to the stirring solution at 0 °C. The resulting suspension was stirred for 15 minutes at 0 °C, the external cooling bath was removed, and the reaction mixture stirred for 5 h at room temperature. The reaction was quenched by adding a saturated aqueous solution of $NaHCO_3$ (10 mL). The aqueous layer was extracted with CH_2Cl_2 (3 x 30 mL) and the combined organic layers were dried over $MgSO_4$, filtered with a filter paper, and the filtrate was concentrated by rotary evaporation. The crude product was purified by silica gel flash chromatography (hexanes/EtOAc 4:6 → 1:4 eluant) to give the disaccharide **15** (33 mg, 51% yield).

Compound **15** was inseparable from an isomeric substance (5.7 : 1 ratio) that we could not adequately characterize.

$[\alpha]^{22}_{D}$ -4.60 (c = 0.1, $CHCl_3$)

^{1}H NMR (800 MHz, C_6D_6) δ 5.52 (d, *J* = 5.0 Hz, 1H), 4.95 (d, *J* = 5.9 Hz, 1H), 4.53 (dd, *J* = 7.9, 2.4 Hz, 1H), 4.30 (td, *J* = 6.7, 1.9 Hz, 1H), 4.19 (dd, *J* = 7.9, 1.9 Hz, 1H), 4.17 (dd, *J* = 8.0, 3.4 Hz, 1H), 4.16 (dd, *J* = 5.1, 2.5 Hz, 1H), 4.08 (dd, *J* = 10.9, 6.9 Hz, 1H), 3.98 (dd, *J* = 10.9, 6.4 Hz, 1H), 3.93 (dd, *J* = 11.7, 7.6 Hz, 1H), 3.73 (dd, *J* = 11.7, 3.9 Hz, 1H), 3.63 (dd, *J* = 7.6, 2.0 Hz, 1H), 3.59 (ddd, *J* = 7.6, 3.9, 1.9 Hz, 1H), 3.56 (dd, *J* = 5.9, 3.1 Hz, 1H), 2.57 (br, 1H), 2.40 (br, 1H), 1.49 (s, 3H), 1.45 (s, 6H), 1.18 (s, 3H), 1.14 (s, 3H), 1.02 (s, 3H).

^{13}C{^{1}H} NMR (101 MHz, C_6D_6) δ 110.1, 109.5, 108.6, 102.5, 96.9, 74.8, 74.6, 71.5, 71.3, 71.2, 71.1, 69.5, 68.0, 66.9, 62.5, 26.31, 26.25, 26.18, 25.2, 24.8, 24.6.

HRMS (ESI): *m/z* calcd for $C_{21}H_{34}O_{11}{}^{35}Cl^-$ $[M + Cl]^-$ 497.1795, found 497.1809.

Disaccharide **15** prepared by this route exhibited an ^{1}H NMR spectrum with slightly different chemical shifts from disaccharide **15** prepared by the modified Julia olefination synthesis, but identical coupling patterns and coupling constants. Moreover the ^{13}C NMR spectra for both samples of **15** were virtually identical.

We determined that there was a substantial concentration effect on ^{1}H chemical shifts in the talopyranosyl region of disaccharide **15**. This was carefully measured by dissolving 18 mg of compound **15** in 0.7 mL of C_6D_6, for a 56 mM concentration sample. This sample was then divided in portions, one diluted to 32 mM and the other to 16 mM. This effect was more significant in concentrated samples, i.e. comparing chemical shifts in the 56 mM sample with the 32 mM sample (**Figures S1, S2**).

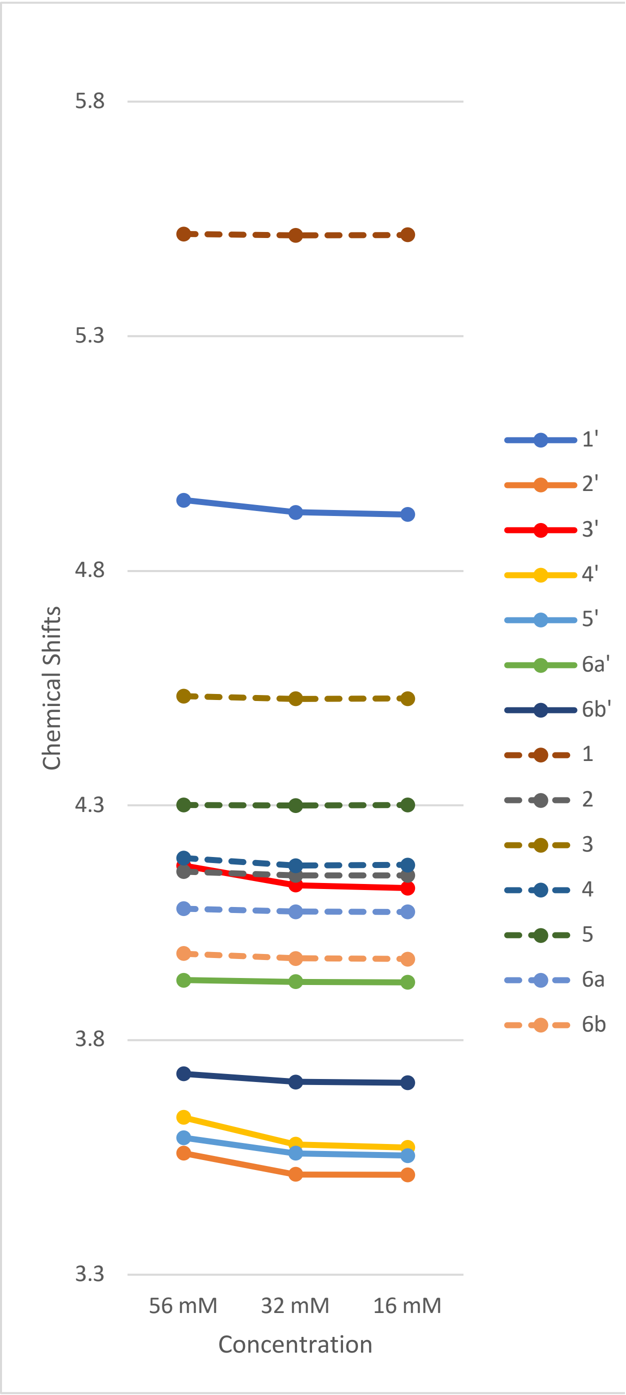

Figure S1. Concentration effects on ^{1}H NMR chemical shifts in disaccharide **15**

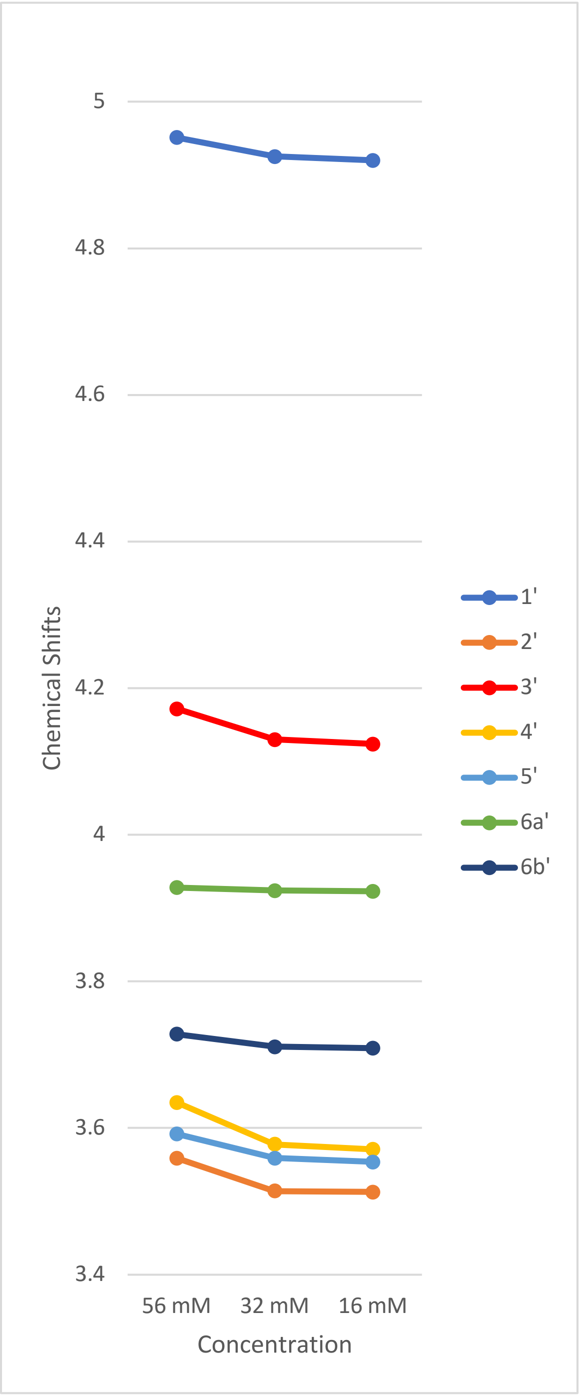

Figure S2. Concentration effects on ^{1}H NMR chemical shifts for only α-talopyranosyl protons in disaccharide **15**

Although we had not previously recorded the concentration of our NMR samples, we now suspect that the differences in ^{1}H NMR chemical shifts between this samples of disaccharide **15** are simply due to a relatively high concentration of disaccharide **15** in the C_6D_6 solution prepared for NMR analysis, providing the data listed on pp. S12-S13.

1,2:3,4-Di-*O*-(isopropylidene)-6-*O*-[3,4-*O*-(isopropylidene)-α-D-talopyranosyl]-α-D-galactopyranose, 2',6'-bis-*O*-acetyl ester (**27**):

A 100 mL round bottom flask with stir bar was charged with disaccharide diol **15** (17 mg, 0.037 mmol), acetic anhydride (10 mL) was added, followed by pyridine (20 mL). The reaction mixture was stirred at room temperature for 16 h. The mixture was then concentrated by rotary evaporation. The crude reaction mixture was quenched with water (20 mL) and brine (10 mL), and the aqueous layer was extracted with EtOAc (3 x 20 mL). The combined organic layers were dried over $MgSO_4$, filtered, and the filtrate was concentrated by rotary evaporation. The crude product was purified by silica gel flash chromatography (petroleum ether : EtOAc 2:1 to 1:1) to afford the corresponding diacetate (**27**, 11 mg, 56 % yield).

^{1}H NMR (800 MHz, C_6D_6) δ 5.49 (d, *J* = 5.0 Hz, 1H), 5.20 (d, *J* = 6.1 Hz, 1H), 5.13 (dd, *J* = 6.2, 2.9 Hz, 1H), 4.56 (dd, *J* = 7.9, 2.4 Hz, 1H), 4.45 (dd, *J* = 7.6, 2.9 Hz, 1H), 4.43 (dd, *J* = 11.4, 7.5 Hz, 1H), 4.31 (dd, *J* = 11.4, 4.8 Hz, 1H), 4.29 (td, *J* = 7.0, 1.9 Hz, 1H), 4.21 (dd, *J* = 7.9, 1.9 Hz, 1H), 4.17 (dd, *J* = 5.0, 2.4 Hz, 1H), 4.13 (dd, *J* = 10.2, 6.3 Hz, 1H), 3.97 (dd, *J* = 10.2, 7.2 Hz, 1H), 3.72 (ddd, *J* = 7.1, 4.8, 1.9 Hz, 1H), 3.60 (dd, *J* = 7.6, 1.9 Hz, 1H), 1.78 (s, 3H), 1.71 (s, 3H), 1.44 (s, 3H), 1.43 (s, 3H), 1.42 (s, 3H), 1.17 (s, 3H), 1.10 (s, 3H), 1.02 (s, 3H).

^{13}C{^{1}H} NMR (101 MHz, C_6D_6) δ 170.1, 169.8, 110.8, 109.2, 108.5, 99.0, 96.9, 74.3, 72.8, 71.5, 71.2, 71.1, 71.0, 68.5, 67.4, 66.6, 63.1, 26.27, 26.25, 26.17, 25.3, 24.9, 24.6, 20.56, 20.55.

HRMS (ESI): *m/z* calcd for $C_{25}H_{38}O_{13}Na^+$ [M + Na]$^+$ 569.2205, found 569.2215.

Assignments of diagnostic ^{13}C and ^{1}H NMR resonances for disaccharide **15** and diacetate derivative **27**, determined by HSQC, COSY, and HMBC spectroscopy:

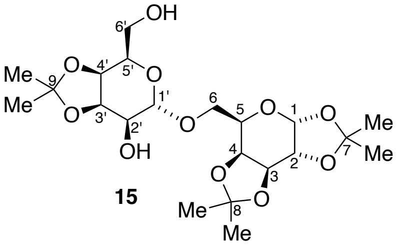

15

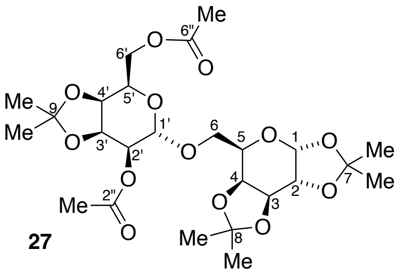

27

carbon position	δ_C [a]	δ_H [b,c] (*J*, Hz)	δ_C [a]	δ_H [b,c] (*J*, Hz)
1'	102.5	4.95, d (5.9)	99.0	5.20, d (6.1)
2'	69.5	3.56, dd (5.9, 3.1)	71.0	5.13, dd (6.2, 2.9)
3'	74.6	4.17, dd (8.0, 3.4)	72.8	4.45, dd (7.6, 2.9)
4'	74.8	3.63, dd (7.6, 2.0)	74.3	3.60, dd (7.6, 1.9)
5'	71.3	3.59, ddd (7.6, 3.9, 1.9)	68.5	3.72, ddd (7.1, 4.8, 1.9)
6a'	62.5	3.93, dd (11.7, 7.6)	63.1	4.43, dd (11.4, 7.5)
6b'		3.73, dd (11.7, 3.9)		4.31, dd (11.4, 4.8)
1	96.9	5.52, d (5.0)	96.9	5.49, d (5.0)
2	71.1	4.16, dd (5.1, 2.5)	71.1	4.17, dd (5.0, 2.4)
3	71.2	4.53, dd (7.9, 2.4)	71.2	4.56, dd (7.9, 2.4)
4	71.5	4.19, dd (7.9, 1.9)	71.5	4.21, dd (7.9, 1.9)
5	66.9	4.30, td (6.7, 1.9)	66.6	4.29, td (7.0, 1.9)
6a	68.0	4.08, dd (10.9, 6.9)	67.4	4.13, dd (10.2, 6.3)
6b		3.98, dd (10.9, 6.4)		3.97, dd (10.2, 7.2)
7	108.5	--	108.5	--
8	109.4	--	109.2	--
9	107.7	--	110.8	--
2"	--	--	169.8	--
6"	--	--	170.0	--

[a] Recorded at 101 MHz in C_6D_6
[b] Recorded at 800 MHz in C_6D_6
[c] Denotes ^{1}H resonance(s) correlating to each ^{13}C for the carbon position

Epoxidation-oxacyclization of (*E*)-25 to disaccharides 28 and 29:

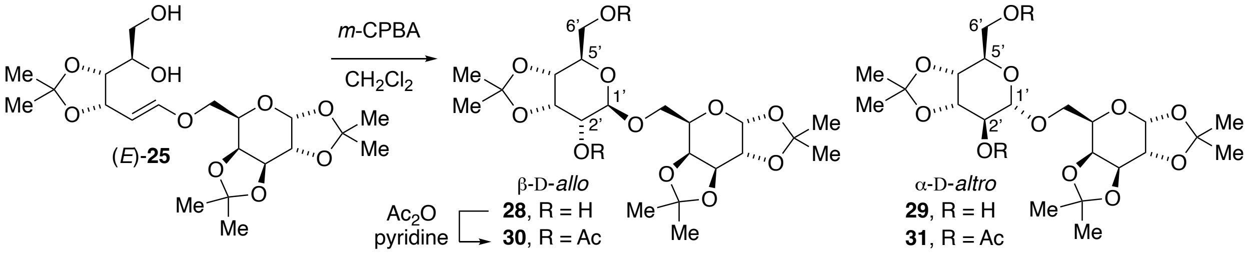

1) with *m*-CPBA: A 5 mL round-bottom flask with stir bar was charged with diol-vinylic ether (*E*)-**25** (46 mg, 0.1 mmol) and CH_2Cl_2 (1.7 mL, 0.1 M), and cooled to 0 °C with an external cooling bath. *m*-CPBA (77% w/w) (36 mg, 0.2 mmol, 1.1 equiv) was slowly added to the stirring solution at 0 °C. The resulting suspension was stirred for 15 minutes at 0 °C, the external cooling bath was removed, and the reaction mixture stirred for 5 h at room temperature. The reaction was quenched by adding a saturated aqueous solution of $NaHCO_3$ (10 mL). The aqueous layer was extracted with CH_2Cl_2 (3 x 30 mL) and the combined organic layers were dried over $MgSO_4$, filtered with a filter paper, and the filtrate was concentrated by rotary evaporation. 1H NMR of the crude mixture indicated a 1.6 : 1 ratio of disaccharide diastereomers **28** and **29**. The crude product was purified by silica gel flash chromatography (hexanes/EtOAc 1:3 → 1:5 eluant) to give partial separation of diastereomers **28** and **29**, with the minor diastereomer **29** eluting first (46 mg, 50% combined yield).

2) with TFAA-UHP: A 3 mL conical vial with stir bar was charged with diol-vinylic ether (*E*)-**25** (12 mg, 0.027 mmol) and CH_2Cl_2 (1.2 mL, 0.2 M), and cooled to 0 °C with an external cooling bath. Na_2HPO_4 (34 mg, 0.24 mmol, 10 equiv), urea-hydrogen peroxide (UHP, 25 mg, 0.27 mmol, 10 equiv), and then trifluoroacetic anhydride (TFAA, 9.5 µL, 0.068 mmol, 3 equiv) were added at 0 °C. The resulting suspension was stirred for 15 minutes at 0 °C, the external cooling bath was removed, and the reaction mixture stirred for 5 h at room temperature. The reaction was quenched by adding a saturated aqueous solution of $NaHCO_3$ (10 mL). The aqueous layer was extracted with CH_2Cl_2 (3 x 30 mL) and the combined organic layers were dried over $MgSO_4$, filtered with a filter paper, and the filtrate was concentrated by rotary evaporation. ^{1}H NMR of the crude mixture indicated a 1.2 : 1 ratio of disaccharide diastereomers **28** and **29**.

3) with cat. $VO(acac)_2$ / TBHP: A 3 mL conical vial with stir bar was charged with diol-vinylic ether (*E*)-**25** (12 mg, 0.027 mmol), $VO(acac)_2$ (17 mg, 0.063 mmol, 2.3 equiv), and CH_2Cl_2 (0.08 mL, 0.3 M), and cooled to 0 °C with an external cooling bath. *Tert*-butyl hydrogen peroxide (TBHP, 5.5 M solution in decane, 0.01 mL, 0.054 mmol, 2 equiv.) was slowly added to the stirring solution at 0 °C. The solution turned from blue green to red. The resulting suspension was stirred for 15 minutes at 0 °C, the external cooling bath was removed, and the reaction mixture stirred for 5 h at room temperature. The reaction was quenched by adding a saturated aqueous solution of $NaHCO_3$ (10 mL). The aqueous layer was extracted with CH_2Cl_2 (3 x 30 mL) and the combined organic layers were dried over $MgSO_4$, filtered with a filter paper, and the filtrate was concentrated by rotary evaporation. 1H NMR of the crude mixture indicated a 4 : 1 ratio of disaccharide diastereomers **28** and **29** along with ~50% of unreacted diol-vinylic ether (*E*)-**25.**

Note: An attempt at higher conversion over 18 hrs resulted only in hydrolyzed compounds.

1,2:3,4-Di-*O*-(isopropylidene)-6-*O*-[3,4-*O*-(isopropylidene)-β-D-allopyranosyl]-α-D-galactopyranose (**28**):

$[\alpha]^{21}_{D}$ -42.9 (c = 0.1, $CHCl_3$)

^{1}H NMR (800 MHz, C_6D_6) δ 5.47 (d, *J* = 5.0 Hz, 1H), 4.87 (d, *J* = 6.7 Hz, 1H), 4.46 (dd, *J* = 8.0, 2.4 Hz, 1H), 4.23 (dd, *J* = 5.5, 4.4 Hz, 1H), 4.20 (dt, *J* = 6.3, 1.8 Hz, 1H), 4.18 (dd, *J* = 9.0, 6.3 Hz, 1H), 4.13 (dd, *J* = 5.1, 2.4 Hz, 1H), 4.08 (dd, *J* = 8.0, 1.6 Hz, 1H), 3.94 (dd, *J* = 8.9, 4.9 Hz, 1H), 3.92 (dd, *J* = 9.1, 5.5 Hz, 1H), 3.82 (ddd, *J* = 11.8, 6.0, 2.7 Hz, 1H), 3.77 (dt, *J* = 6.6, 4.4 Hz, 1H), 3.66 (ddd, *J* = 9.1, 5.5, 2.7 Hz, 1H), 3.61 (dt, *J* = 11.8, 5.9 Hz, 1H), 2.60 (d, *J* = 5.4 Hz, 1H), 2.36 (t, *J* = 6.7 Hz, 1H), 1.47 (s, 3H), 1.44 (s, 3H), 1.29 (s, 3H), 1.20 (s, 3H), 1.14 (s, 3H), 1.01 (s, 3H).

$^{13}C\{^1H\}$ NMR (101 MHz, C_6D_6) δ 109.9, 109.5, 108.6, 101.4, 96.9, 76.4, 75.0, 72.5, 71.3, 71.2, 71.0, 69.1, 68.3, 67.7, 62.8, 27.9, 26.2 (2C), 25.8, 24.8, 24.2.

HRMS (ESI): *m/z* calcd for $C_{21}H_{34}O_{11}Na^+$ $[M + Na]^+$ 485.1993, found 485.2006.

1,2:3,4-Di-*O*-(isopropylidene)-6-*O*-[3,4-*O*-(isopropylidene)-α-D-altropyranosyl]-α-D-galactopyranose (**29**):

$[\alpha]^{21}_{D}$ -4.5 (c = 0.1, $CHCl_3$)

^{1}H NMR (800 MHz, C_6D_6) δ 5.51 (d, *J* = 5.1 Hz, 1H), 4.67 (d, *J* = 5.9 Hz, 1H), 4.52 (dd, *J* = 7.9, 2.4 Hz, 1H), 4.28 (td, *J* = 6.7, 1.9 Hz, 1H), 4.20 (dd, *J* = 8.0, 1.9 Hz, 1H), 4.16 (dd, *J* = 5.1, 2.4 Hz, 1H), 4.07 (dd, *J* = 10.6, 6.6 Hz, 1H), 4.01 - 3.95 (overlapping m, 4H), 3.91 (t, *J* = 6.6 Hz, 1H), 3.82 (dq, *J* = 12.2, 2.5 Hz, 1H), 3.61 (dt, *J* = 11.4, 5.2 Hz, 1H), 2.37 (br s, 1H), 2.21 (br t, *J* = 6.6 Hz, 1H), 1.46 (s, 4H), 1.46 (s, 3H), 1.38 (s, 3H), 1.16 (s, 3H), 1.15 (s, 3H), 1.02 (s, 3H).

$^{13}C\{^1H\}$ NMR (101 MHz, C_6D_6) δ 110.5, 109.4, 108.6, 102.7, 96.9, 77.4, 73.7, 72.8, 72.6, 71.4, 71.2, 71.1, 68.2, 67.0, 63.8, 27.5, 26.3, 26.2, 25.2, 24.8, 24.5.

HRMS (ESI): *m/z* calcd for $C_{21}H_{34}O_{11}Na^+$ $[M + Na]^+$ 485.1993, found 485.1990.

Assignments of diagnostic ^{13}C and ^{1}H NMR resonances for β-D-*allo*-disaccharide **28** and α-D-*altro*-disaccharide **29**, determined by HSQC, COSY, and HMBC spectroscopy:

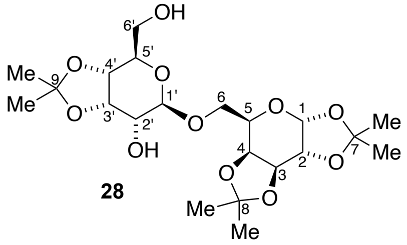

28

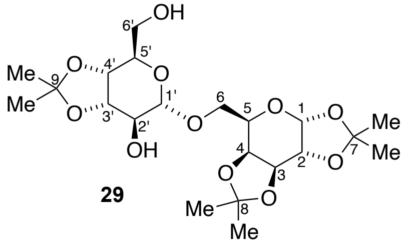

29

carbon position	δ_C[a]	δ_H[b,c] (J, Hz)	δ_C[a]	δ_H[b,c] (J, Hz)
1'	101.4	4.87, d (6.7)	102.7	4.67, d (5.9)
2'	69.1	3.77, dt (6.6, 4.4)	73.7	3.91, t (6.6)
3'	75.0	4.23, dd (5.5, 4.4)	77.4[d]	3.98,[d] m
4'	72.5	3.92, dd (9.1, 5.6)	72.6[d]	3.99,[d] m
5'	76.4	3.66, ddd (9.1, 5.5, 2.7)	72.8	3.96,[d] m
6a'	62.8	3.82, ddd (11.8, 6.0, 2.7)	63.8	3.81, ddd (11.8, 6.8, 2.3)
6b'		3.61, dt (11.8, 5.9)		3.60, dt (11.8, 5.9)
1	96.9	5.47, d (5.0)	96.9	5.50, d (5.0)
2	71.0	4.13, dd (5.1, 2.4)	71.1	4.16, dd (5.1, 2.4)
3	71.2	4.46, dd (8.0, 2.4)	71.2	4.52, dd (7.9, 2.4)
4	71.3	4.08, dd (8.0, 1.6)	71.4	4.18, dd (7.9, 1.9)
5	67.7	4.20, td (6.3, 1.8)	67.0	4.28, td (6.7, 1.9)
6a	68.3	4.18, dd (9.0, 6.3)	68.3	4.07, dd (10.6, 6.7)
6b		3.94, dd (8.9, 4.9)		4.00,[d] m
7	108.6	--	108.6	--
8	109.5	--	109.4	--
9	109.9	--	110.5	--

[a] Recorded at 101 MHz in C_6D_6

[b] Recorded at 800 MHz in C_6D_6

[c] Denotes ^{1}H resonance(s) correlating to each ^{13}C for the carbon position

[d] Due to overlapping ^{1}H peaks, including in HMBC, these assignments may be swapped.

1,2:3,4-Di-*O*-(isopropylidene)-6-*O*-[3,4-*O*-(isopropylidene)-β-D-allopyranosyl]-α-D-galactopyranose, 2',6'-bis-*O*-acetyl ester (**30**):

A 100 mL round bottom flask with stir bar was charged with disaccharide diol **28** (11 mg, 0.024 mmol), acetic anhydride (5 mL) was added, followed by pyridine (10 mL). The reaction mixture was stirred at room temperature for 16 h. The mixture was then concentrated by rotary evaporation. The crude reaction mixture was quenched with water (20 mL) and brine (10 mL), and the aqueous layer was extracted with EtOAc (3 x 20 mL). The combined organic layers were dried over $MgSO_4$, filtered, and the filtrate was concentrated by rotary evaporation to afford the corresponding diacetate **30** (13 mg, quant.).

1,2:3,4-Di-*O*-(isopropylidene)-6-*O*-[3,4-*O*-(isopropylidene)-α-D-altropyranosyl]-α-D-galactopyranose, 2',6'-bis-*O*-acetyl ester (**31**):

A 100 mL round bottom flask with stir bar was charged with disaccharide diol **29** (8 mg, 0.017 mmol), acetic anhydride (5 mL) was added, followed by pyridine (10 mL). The reaction mixture was stirred at room temperature for 16 h. The mixture was then concentrated by rotary evaporation. The crude reaction mixture was quenched with water (20 mL) and brine (10 mL), and the aqueous layer was extracted with EtOAc (3 x 20 mL). The combined organic layers were dried over $MgSO_4$, filtered, and the filtrate was concentrated by rotary evaporation to afford the corresponding diacetate **31** (10 mg, quant.).

Assignments of diagnostic ^{13}C and ^{1}H NMR resonances for disaccharide diacetates **30** and **31**, determined by HSQC, COSY, and HMBC spectroscopy:

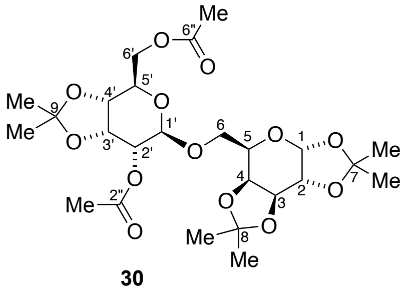

30

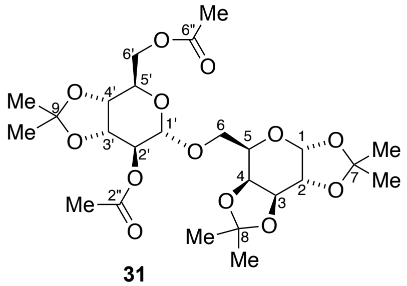

31

carbon position	δ_{C}[a]	δ_{H}[b,c] (*J*, Hz)	δ_{C}[a]	δ_{H}[b,c] (*J*, Hz)
1'	100.0	5.01, d (7.7)	99.3	4.83, d (3.5)
2'	69.4	5.32, dd (7.7, 4.3)	71.7[d]	5.51, dd (5.6, 3.6)
3'	74.2	4.43, t (4.7)	75.2	4.10, t (6.0)
4'	73.0	3.72, dd (9.3, 4.9)	68.4	4.23, m
5'	73.9	3.68, ddd (9.1, 6.0, 2.7)	71.9[d]	3.99, dd (9.1, 6.5)
6a'	63.8	4.35, dd (11.9, 2.7)	64.2	4.44, d (9.4 Hz)
6b'		4.20, dd (11.9, 6.0)		4.25, m
1	96.8	5.48, d (4.9)	96.8	5.46, d (5.0)
2	71.0	4.13, dd (4.9, 2.4)	71.1[d]	4.16, dd (5.0, 2.3)
3	71.2	4.41, dd (8.0, 2.4)	71.2[d]	4.50, dd (8.0, 2.3)
4	71.6	3.87, dd (8.0, 1.8)	71.3[d]	4.19, dd (7.9, 1.9)
5	68.2	4.12, m	66.8	4.22, m
6a	69.4	4.22, dd (10.9, 6.0)	67.4	4.11, dd (9.8, 6.1)
6b		3.94, dd (10.9, 7.2)		3.87, dd (9.8, 7.1)
7	108.5	--	108.5	--
8	109.3	--	109.2	--
9	110.5	--	110.8	--
2"	169.7	--	168.9	--
6"	170.1	--	170.1	--

[a] Recorded at 101 MHz in C_6D_6
[b] Recorded at 800 MHz in C_6D_6
[c] Denotes ^{1}H resonance(s) correlating to each ^{13}C for the carbon position
[d] Due to overlapping ^{13}C peaks in HMBC, these assignments are tentative.

References

(1) Godman, J. L.; Horton, D. Reaction of methyl sulfoxide-acetic anhydride with 1,2:3,4-di-*O*-isopropylidene-α-D-galactopyranose. *Carbohyd. Res.* **1968**, *6*, 229-232.

(2) Oka, N.; Mori, A.; Suzuki, K.; Ando, K. Stereoselective Synthesis of Ribofuranoid *exo*-Glycals by One-Pot Julia Olefination Using Ribofuranosyl Sulfones. *J. Org. Chem.* **2021**, *86*, 657-673.

(3) Mahankali, B.; Srihari, P. A Carbohydrate Approach for the First Total Synthesis of Cochliomycin C: Stereoselective Total Synthesis of Paecilomycin E, Paecilomycin F and 6-*epi*-Cochliomycin C. *Eur. J. Org. Chem.* **2015**, 3983-3993.

(4) Betaneli, V. I.; Ott, A. Y.; Brukhanova, O. V.; Kochetkov, N. K. Synthesis of 1,2-*O*-Cyanoethylidene Derivatives of Alkyl Glycopyranuronates by Oxidation of the 6-Trityl Ethers of Their Hexose Analogues. *Carbohydr. Res.* **1988**, *179*, 37-50.

(5) Matwiejuk, M.; Thiem, J. Hydroxy Group Acidities of Partially Protected Glycopyranosides. *Eur. J. Org. Chem.* **2012**, 2180-2187.

(6) Attolino, E.; Catelani, G.; D'Andrea, F. Regiospecific Synthesis of 4-Deoxy-D-*threo*-hex-3-enopyranosides by Simultaneous Activation-Elimination of the Talopyranoside Axial 4-OH with the NaH/Im_2SO_2 System: Manifestation of the Stereoelectronic Effect. *Eur. J. Org. Chem.* **2006**, 5279-5292.

(7) McDonald, F. E.; Ding, D.; Ephron, A. J.; Bacsa, J. Alkynylation of Pentose Derivatives with Stereochemical Fidelity: Implications for the Regioselectivity of Alkynyl Diol Cycloisomerizations to Cyclic Enol Ethers. *Org. Lett.* **2019**, *21*, 3295–3298.

(8) Song, W.-S.; Liu, S.-X.; Chang, C.-C. Synthesis of L-Deoxyribonucleosides from D-Ribose. *J. Org. Chem.* **2018**, *83*, 14923-14932.

(9) Pietruszka, J.; Witt, A. Synthesis of the Bestmann-Ohira Reagent. *Synthesis* **2006**, 4266–4268.

(10) Boone, M. A.; McDonald, F. E.; Lichter, J.; Lutz, S.; Cao, R.; Hardcastle, K. I. 1,5-α-D-Mannoseptanosides, Ring-Size Isomers That Are Impervious to α-Mannosidase-Catalyzed Hydrolysis. *Org. Lett.* **2009**, *11*, 851–854.
