# Supplementary material for: Nontraditional Synthesis of Disaccharides via Acyclic Vinylic Ether Intermediates: Catalytic C–O Cross-Coupling as the Enabling Link
Source: J Org Chem. 2024 Nov 27;89(24):18684–8. doi: 10.1021/acs.joc.4c02176 (PMC11667969; doi:10.1021/acs.joc.4c02176)

Supporting Information

Non-traditional synthesis of disaccharides via acyclic vinylic ether intermediates: Catalytic C-O cross-coupling as the enabling link

Taehee Kim, Eric J. Meindl and Frank E. McDonald*

Department of Chemistry, Emory University, 1515 Dickey Drive NE, Atlanta GA 30322 USA

*Corresponding author: fmcdona@emory.edu

Part 2: ^{1}H and ^{13}C NMR spectra for new compounds

^{1}H NMR Spectrum of **7** (600 MHz, $CDCl_3$)

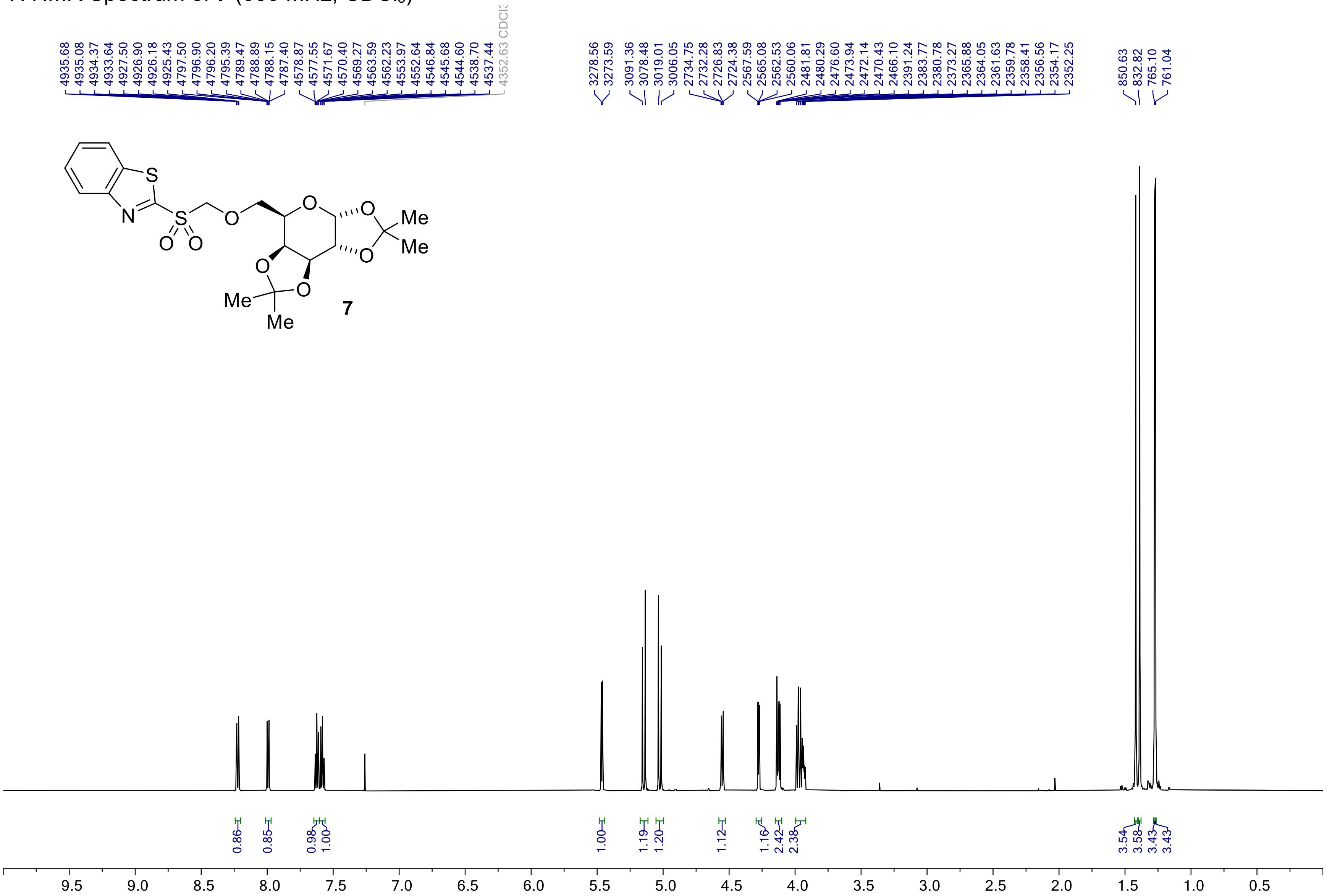

$^{13}C\{^{1}H\}$ NMR Spectrum of **7** (151 MHz, $CDCl_3$)

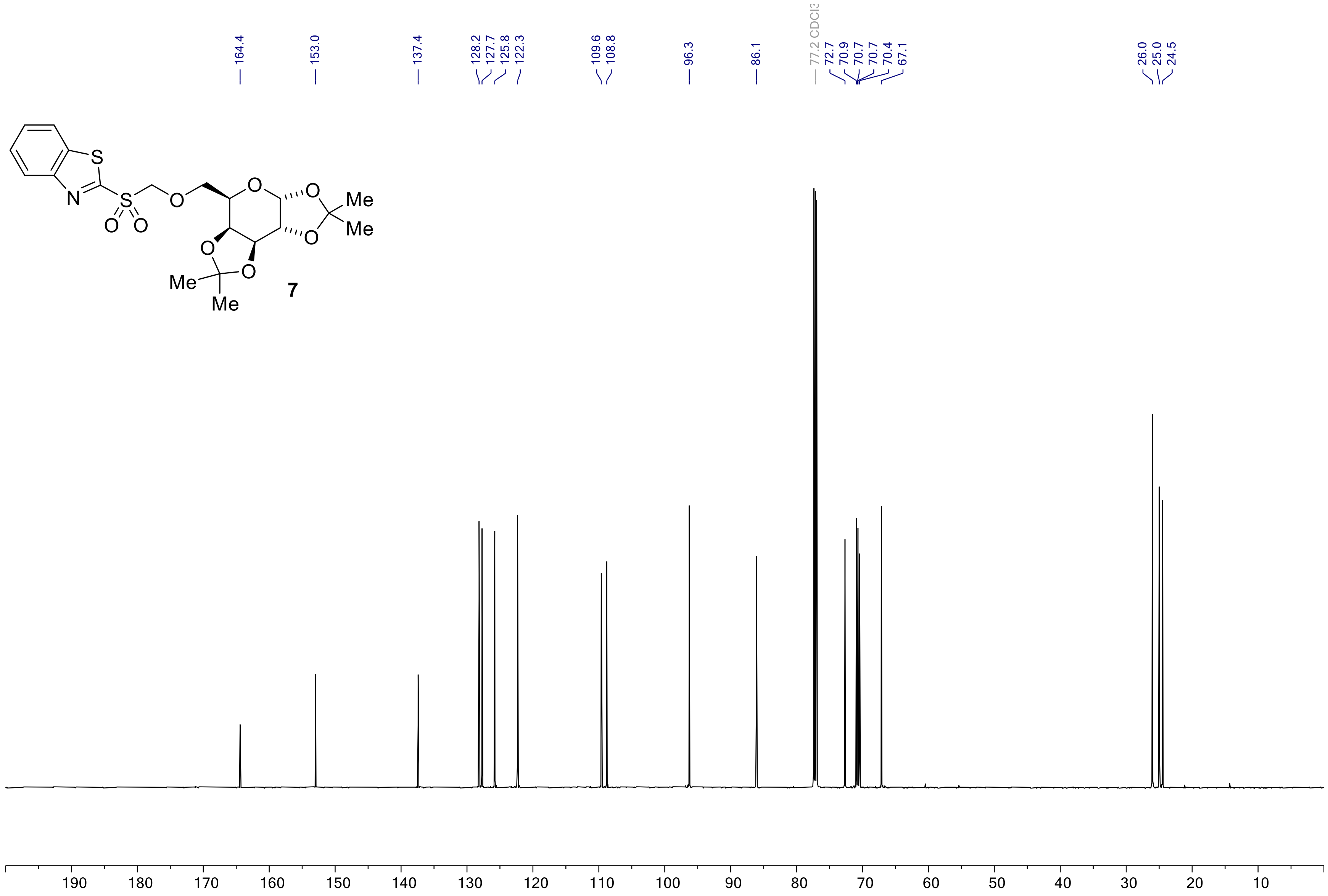

7

^{1}H NMR Spectrum of **9** (600 MHz, $CDCl_3$)

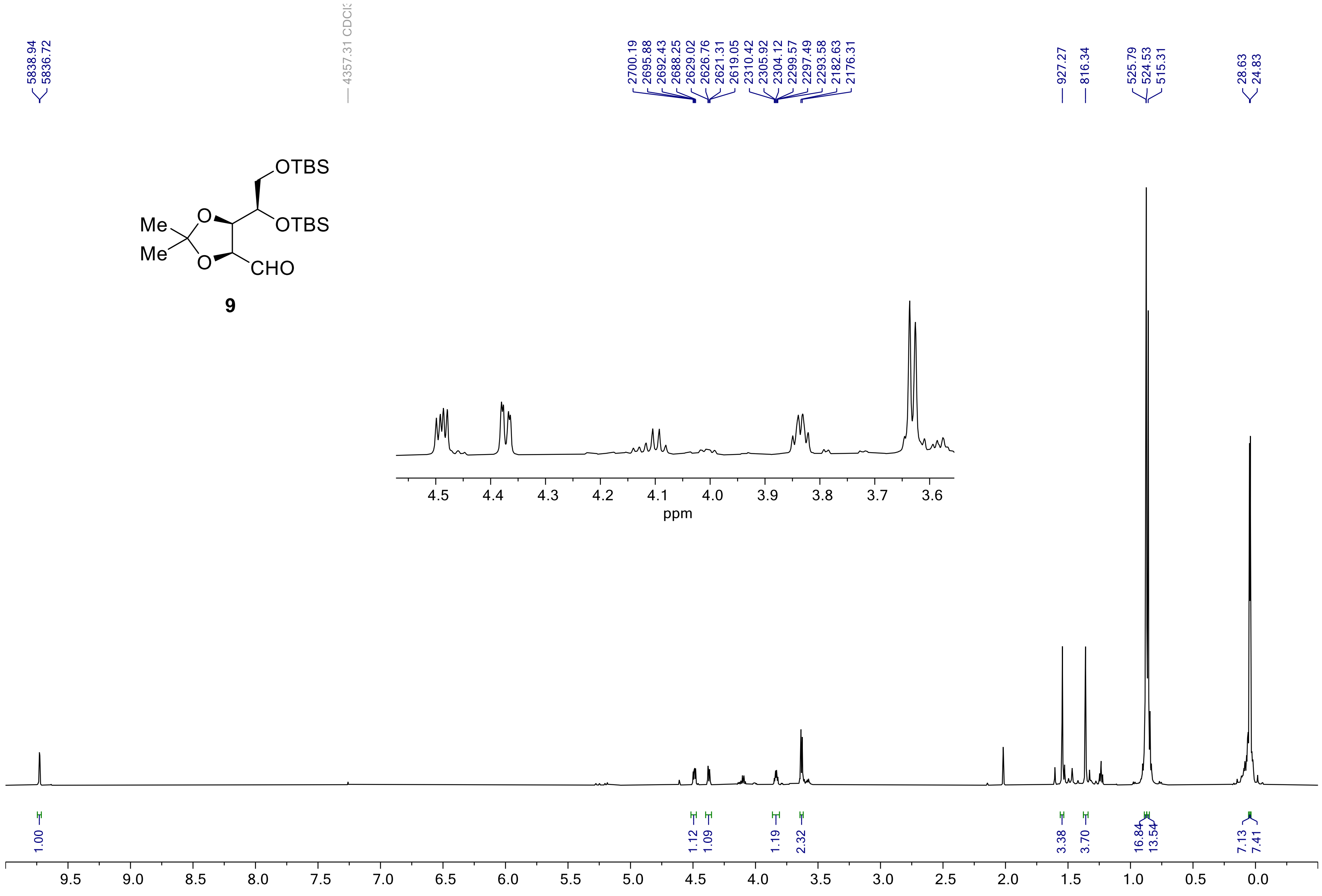

^{1}H NMR Spectrum of **14** (600 MHz, C_6D_6) – full spectrum, with ethyl acetate impurity [δ 3.89 (q), 1.65 (s), 0.92 (t)]

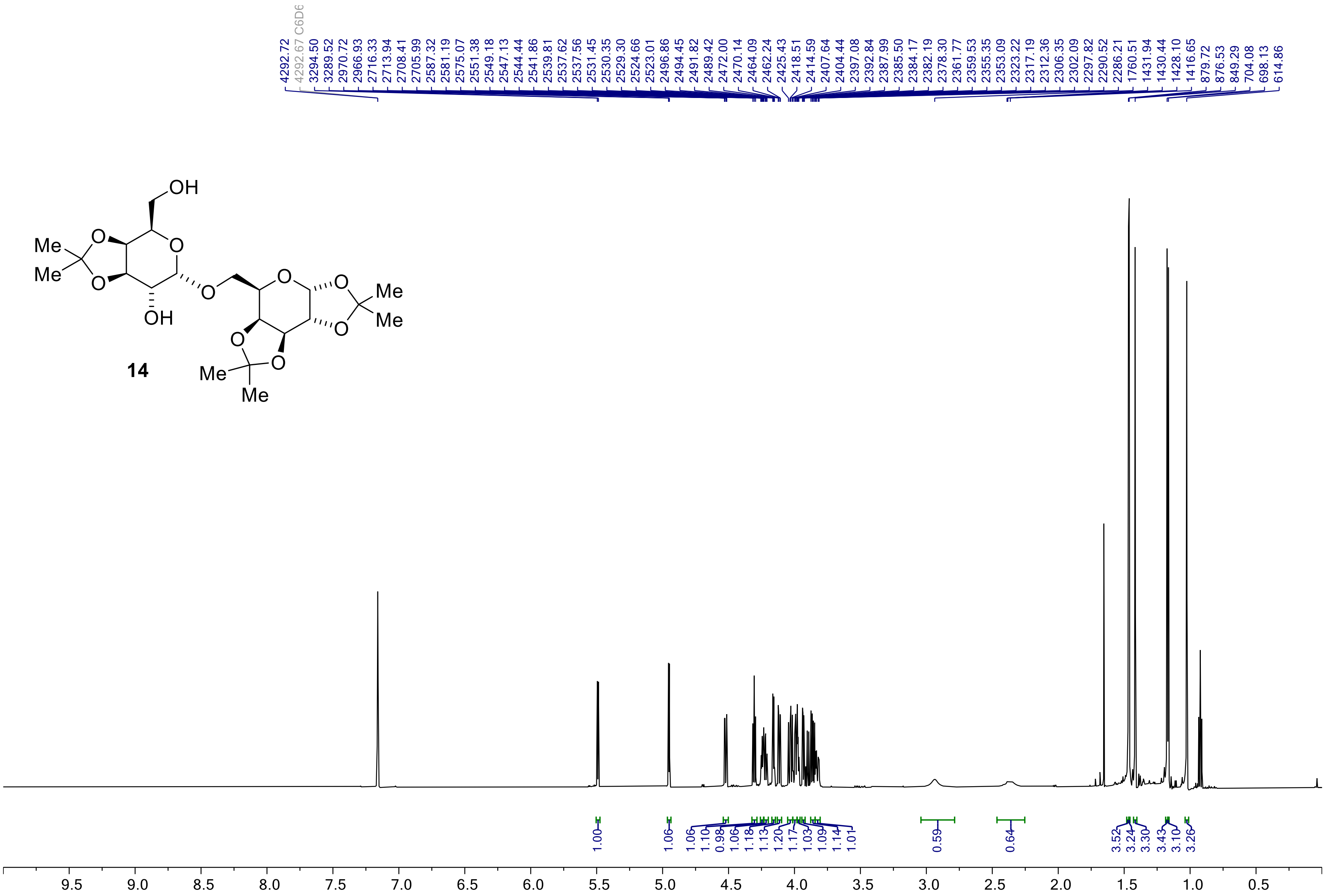

14

^{1}H NMR Spectrum of **14** (600 MHz, C_6D_6) – expansion, with ethyl acetate impurity [δ 3.89 (q)]

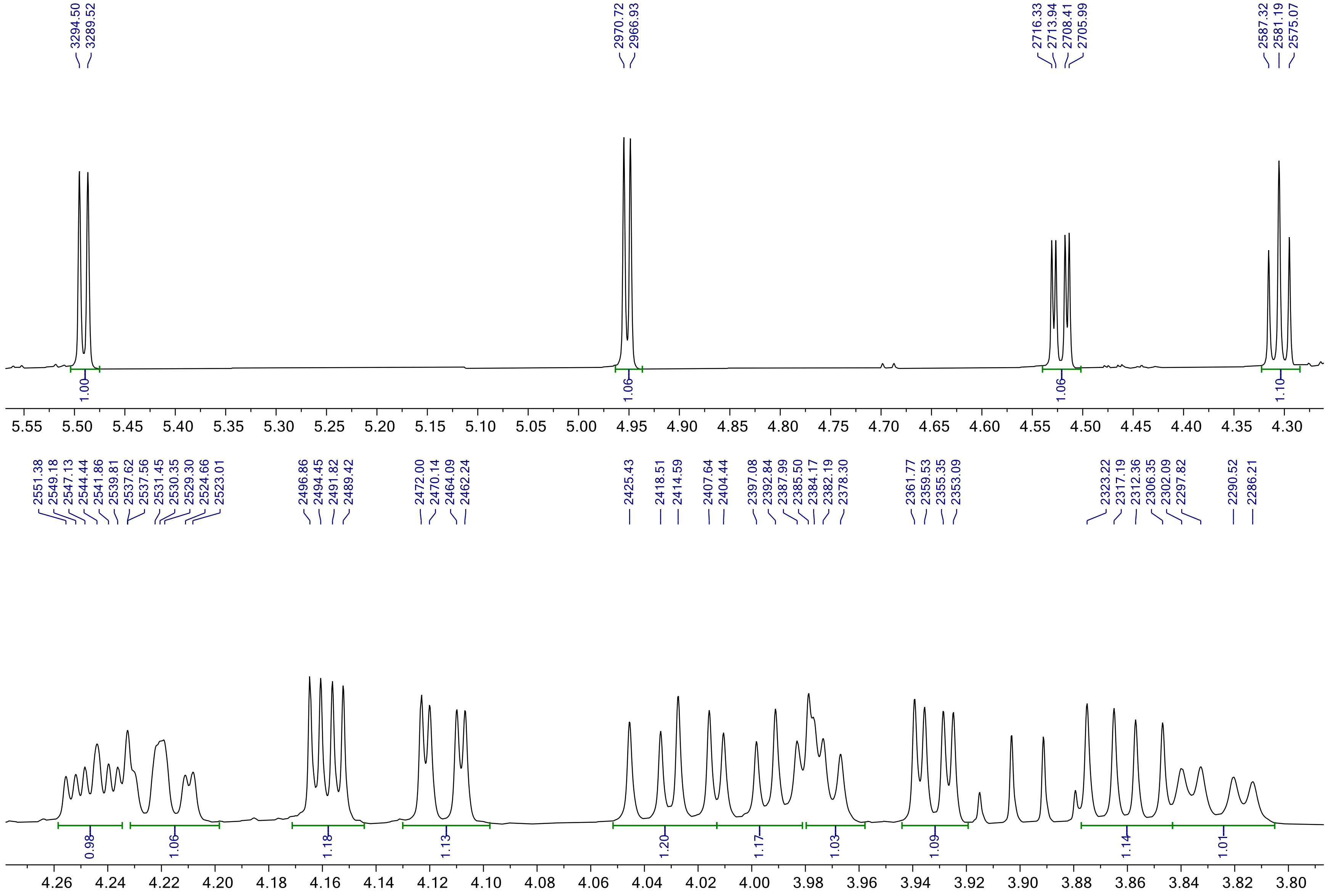

$^{13}C\{^{1}H\}$ NMR Spectrum of **14** (151 MHz, C_6D_6), with ethyl acetate impurity [δ 170.4, 60.2, 20.6, 14.2]

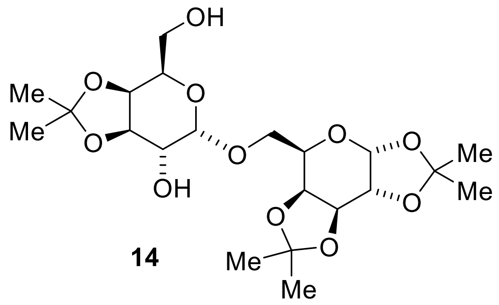

14

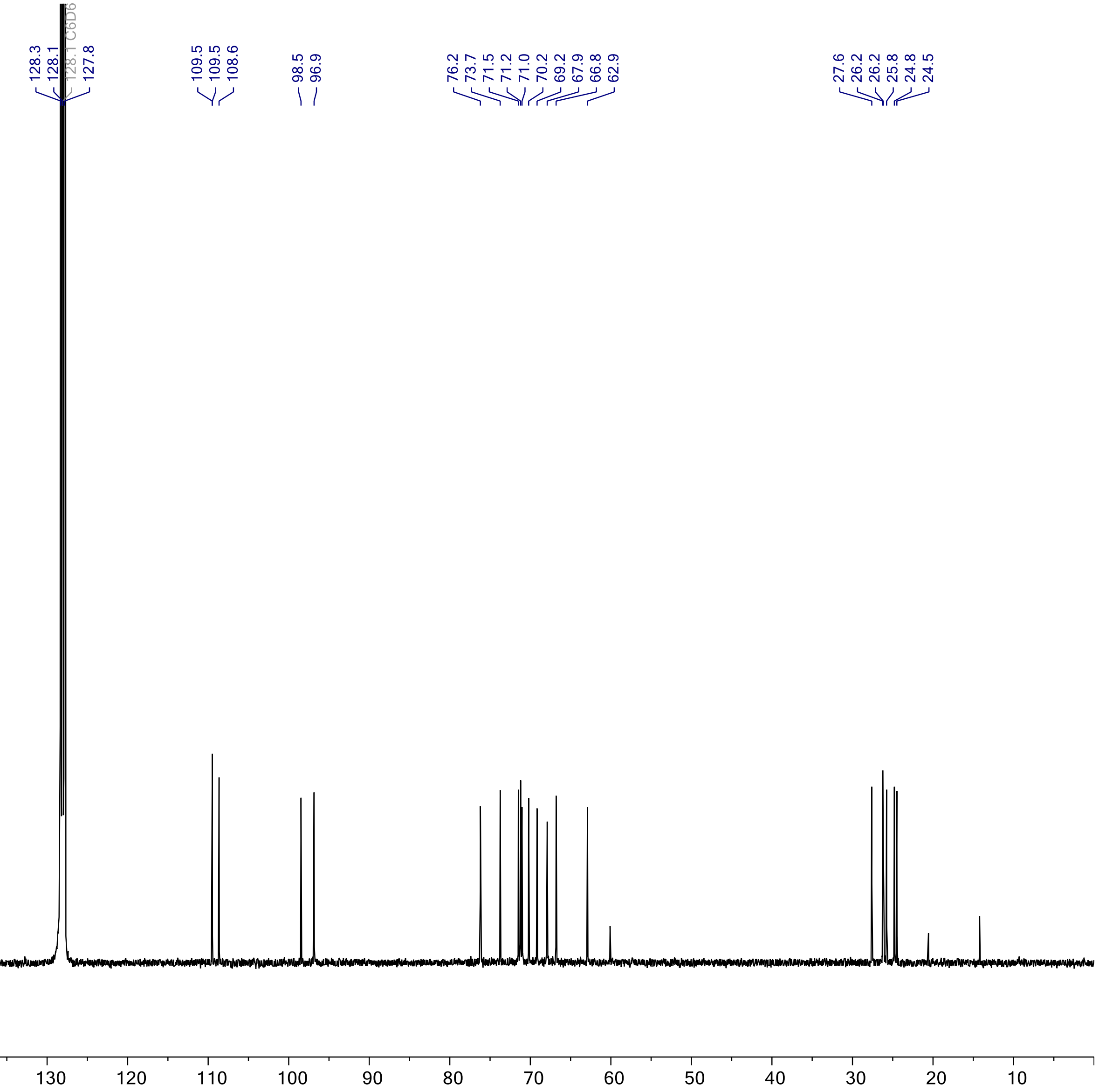

COSY spectrum of **14** (400 MHz, C_6D_6) – full spectrum

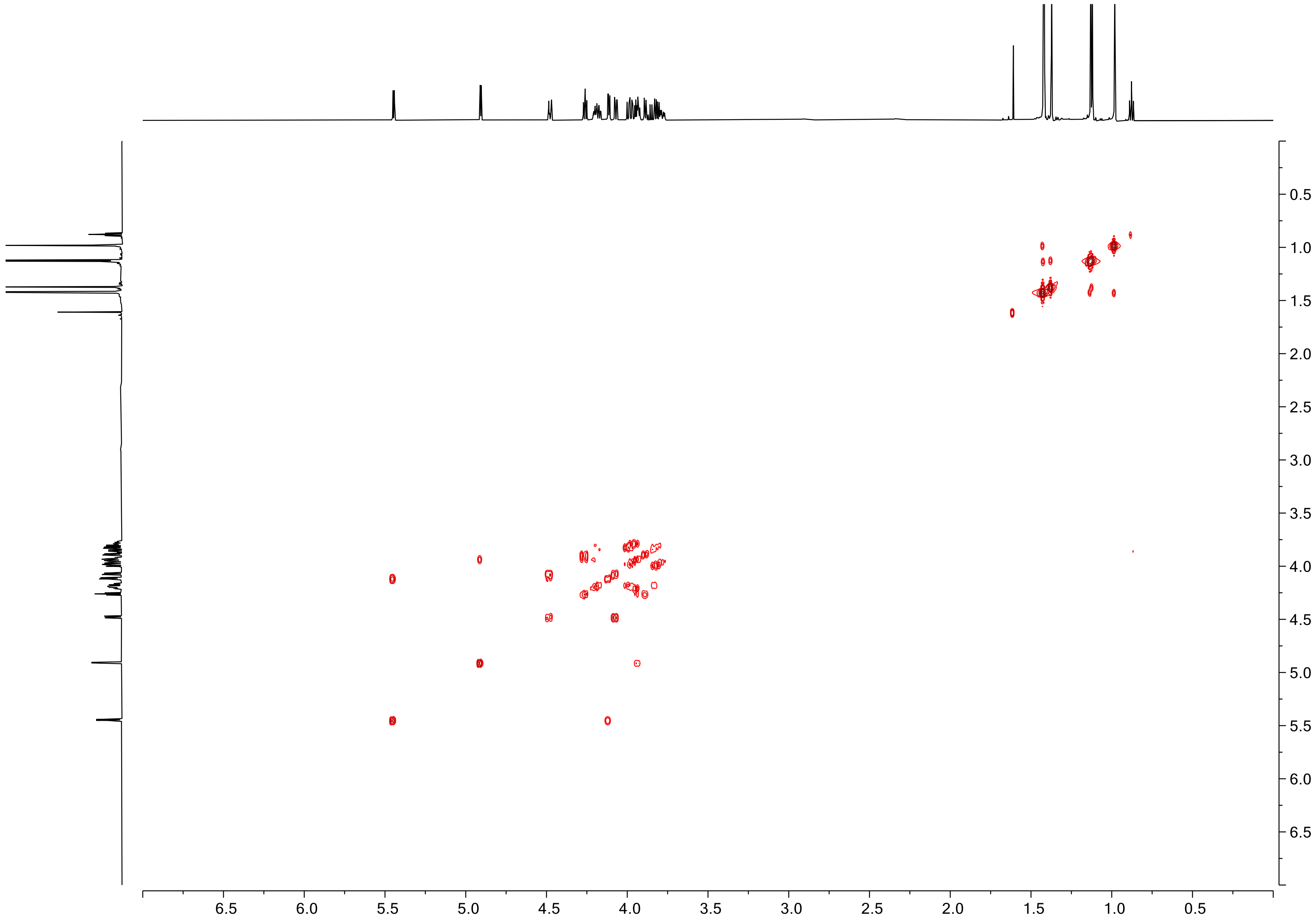

COSY spectrum of **14** (400 MHz, C_6D_6) – expansion

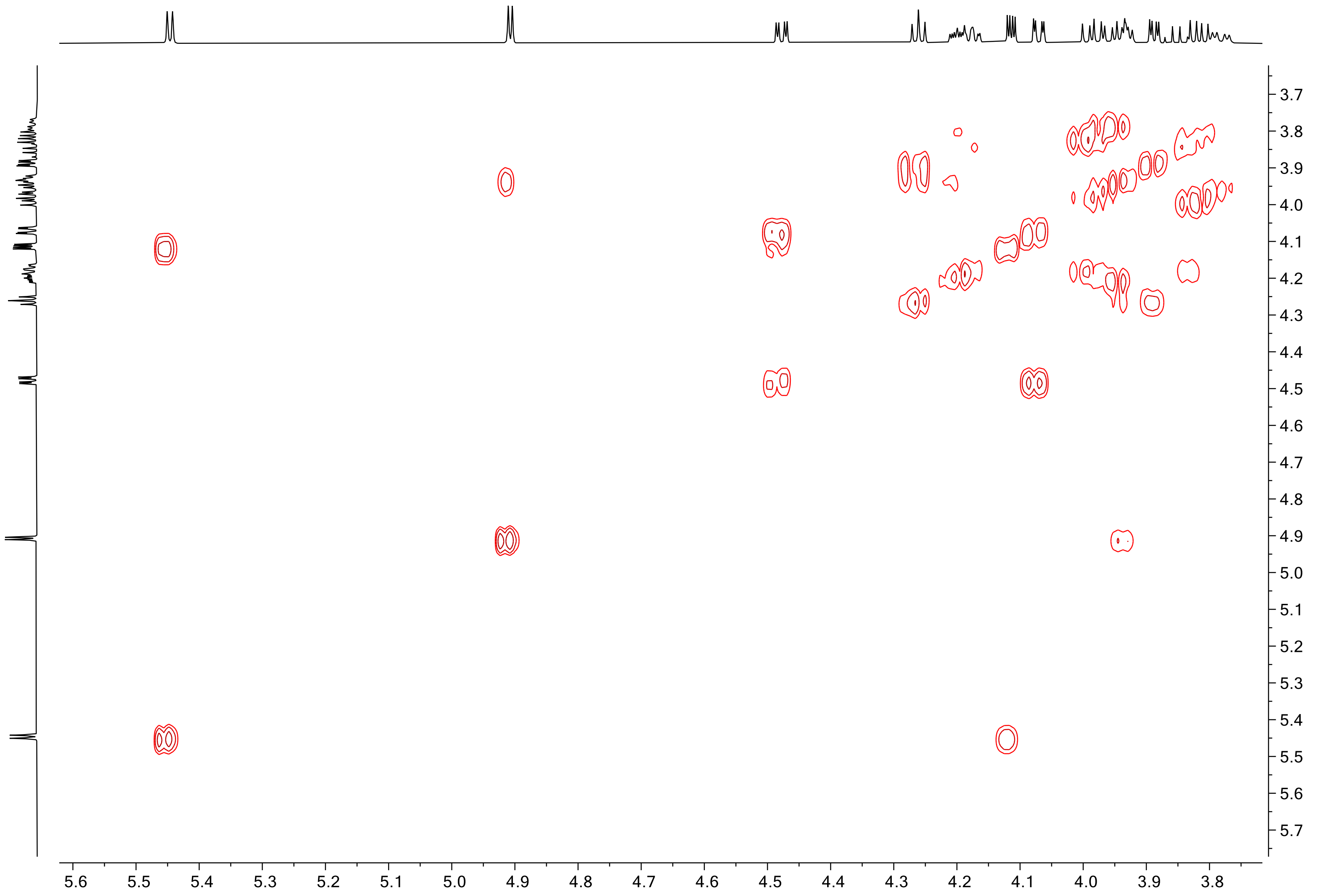

HSQC spectrum of **14** (400 MHz, C_6D_6) – full spectrum

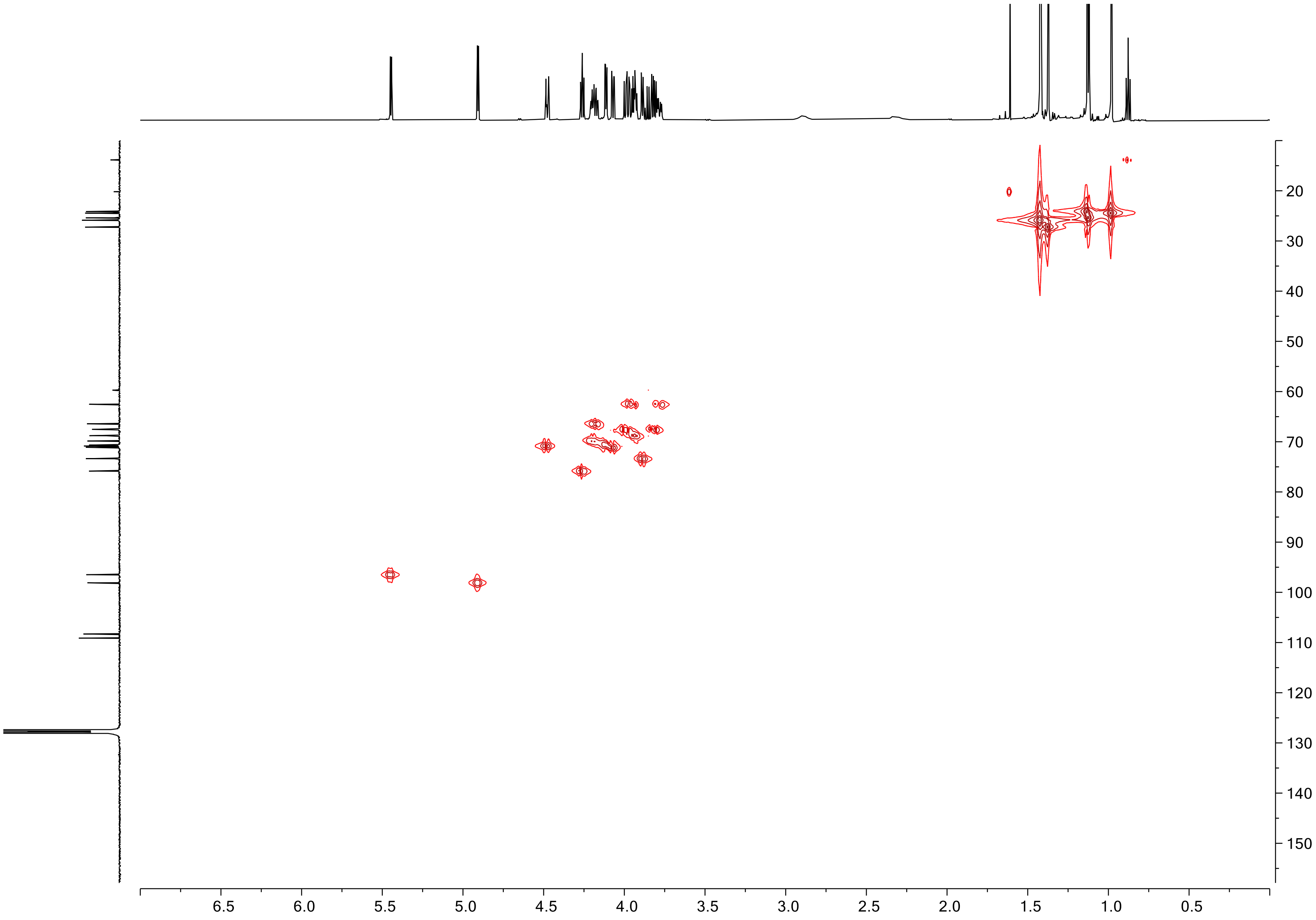

HSQC spectrum of **14** (400 MHz, C_6D_6) – expansion

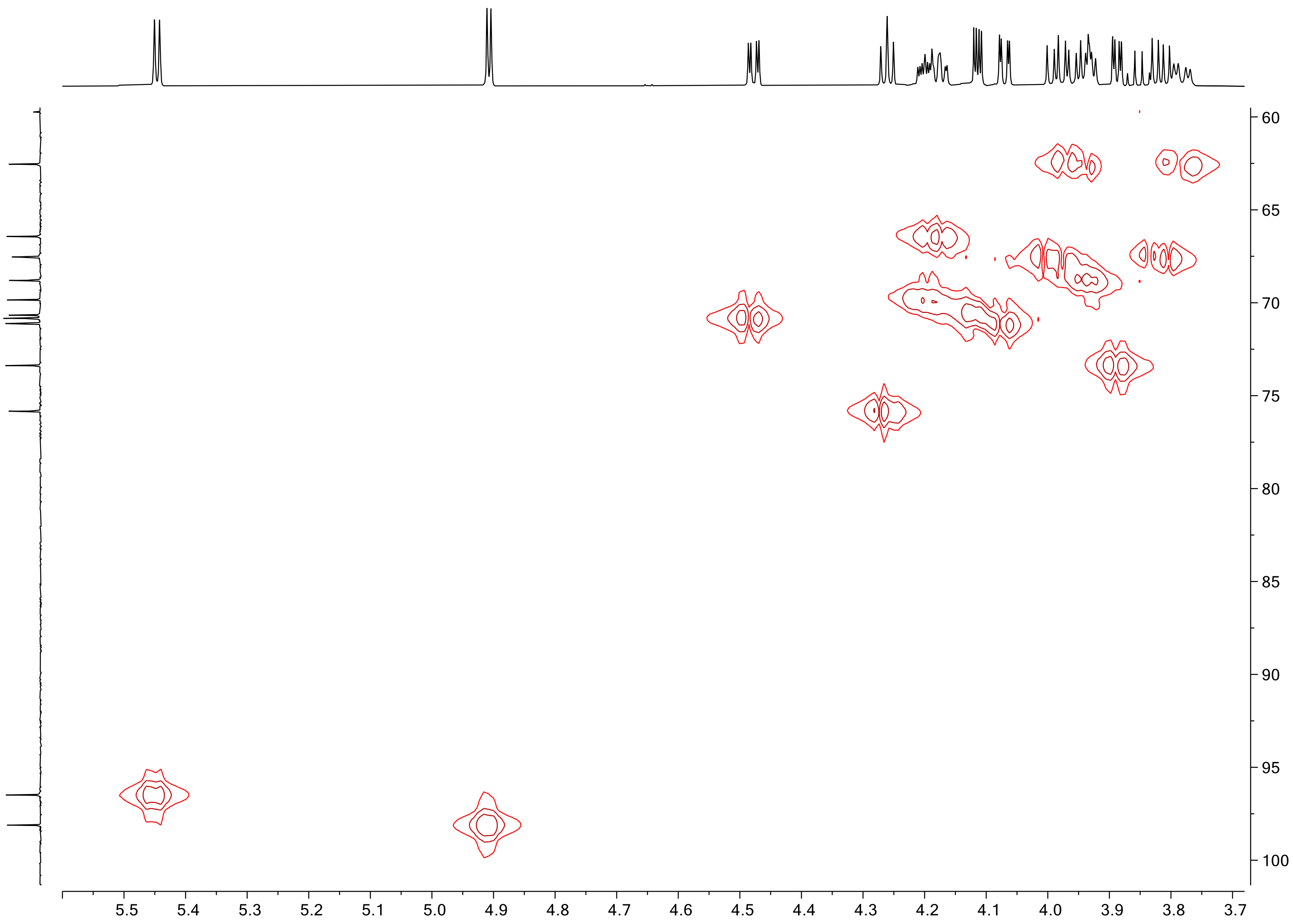

^{1}H NMR spectrum of **15** from mixture of E/Z vinylic ethers (600 MHz, C_6D_6) – full spectrum, with ethyl acetate impurity [δ 3.89 (q), 1.65 (s), 0.92 (t)]

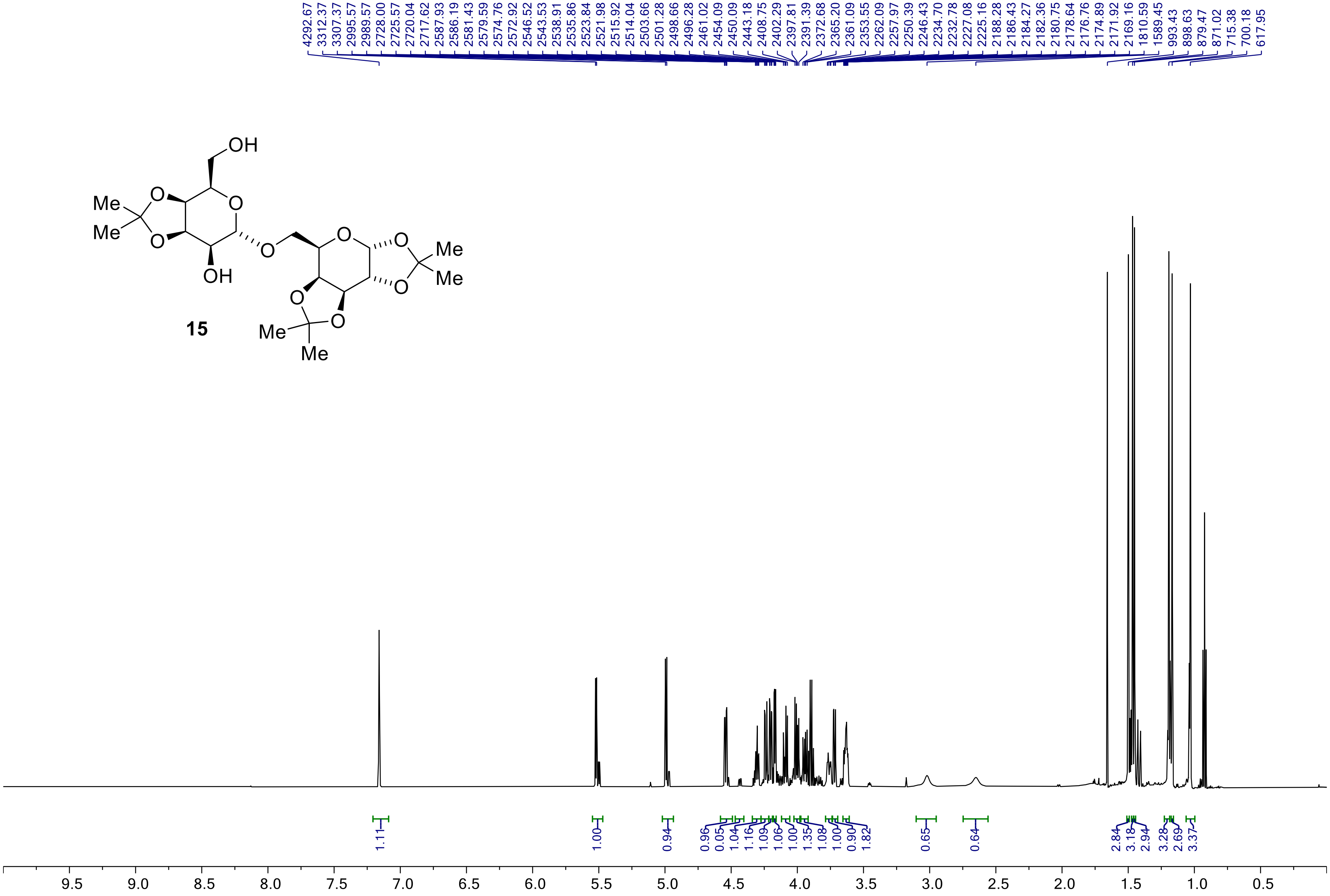

^{1}H NMR Spectrum of **15** from a mixture of E/Z vinylic ethers (600 MHz, C_6D_6) – expansion, with a minor inseparable isomer, and ethyl acetate impurity [δ 3.89 (q)]

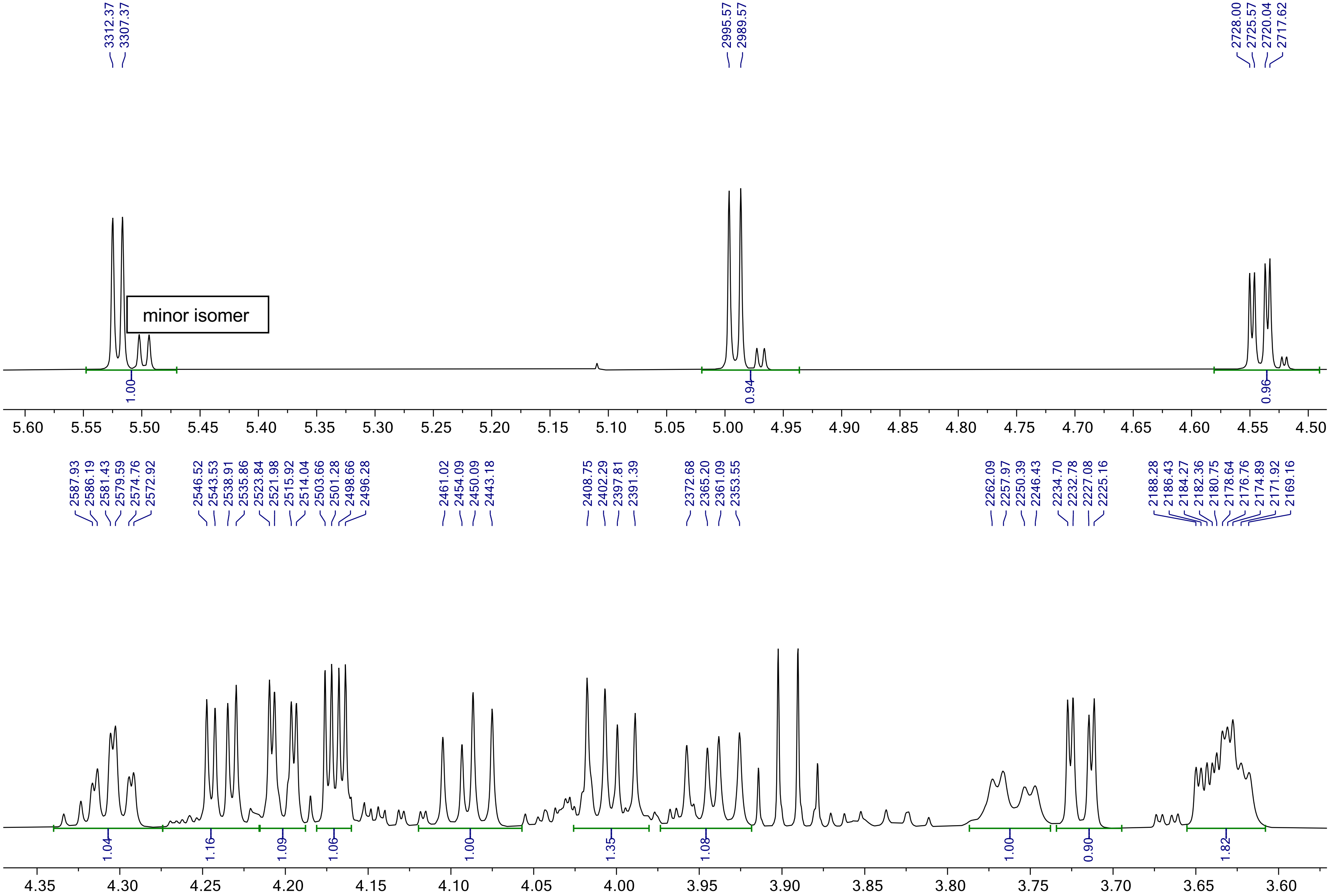

$^{13}C\{^{1}H\}$ NMR Spectrum of **15** from a mixture of E/Z vinylic ethers (151 MHz, C_6D_6), with a minor inseparable isomer, and ethyl acetate impurity [δ 170.4, 60.2, 20.6, 14.2]

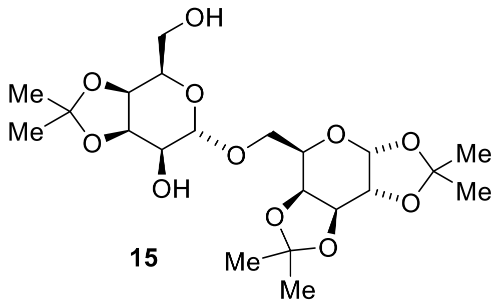

15

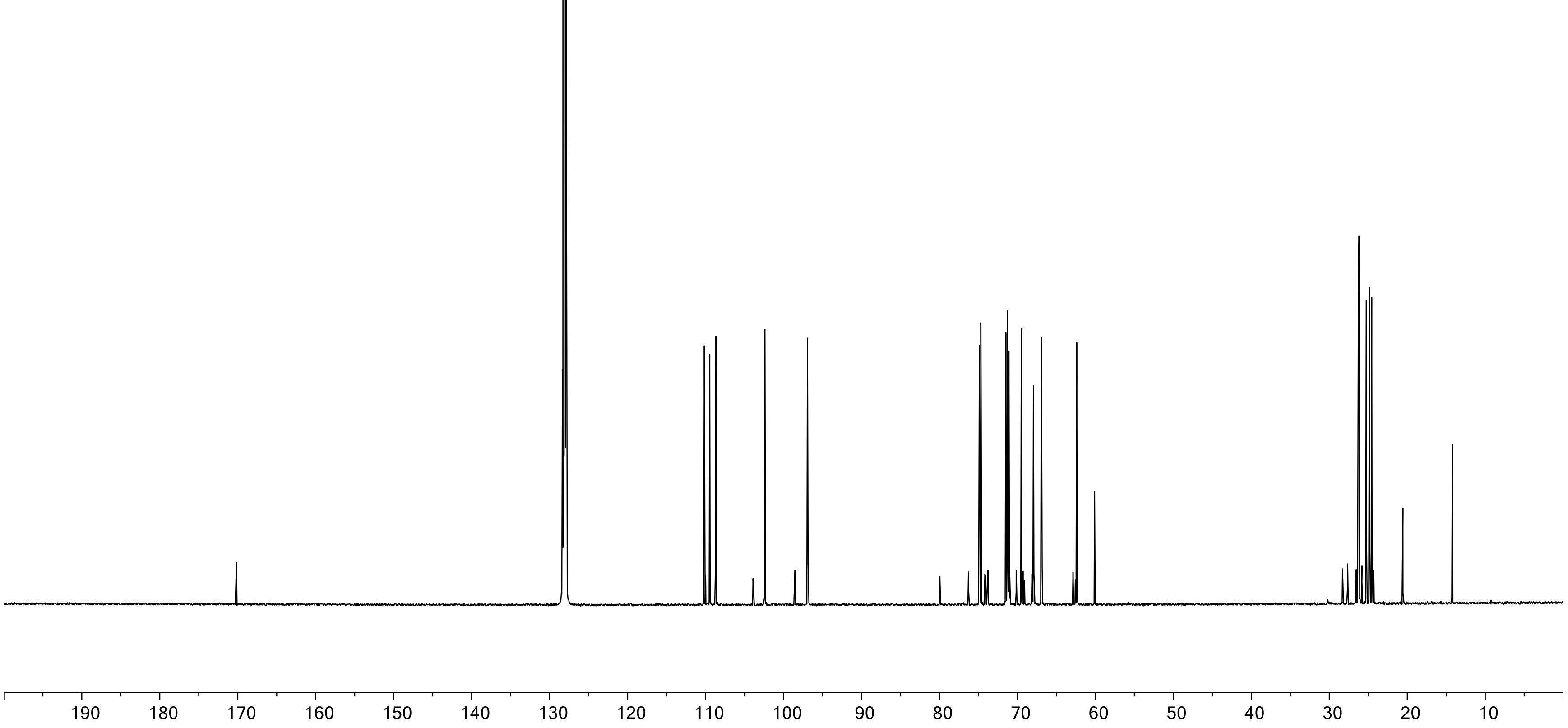

COSY spectrum of **15** from mixture of E/Z vinylic ethers (600 MHz, C_6D_6) – full spectrum

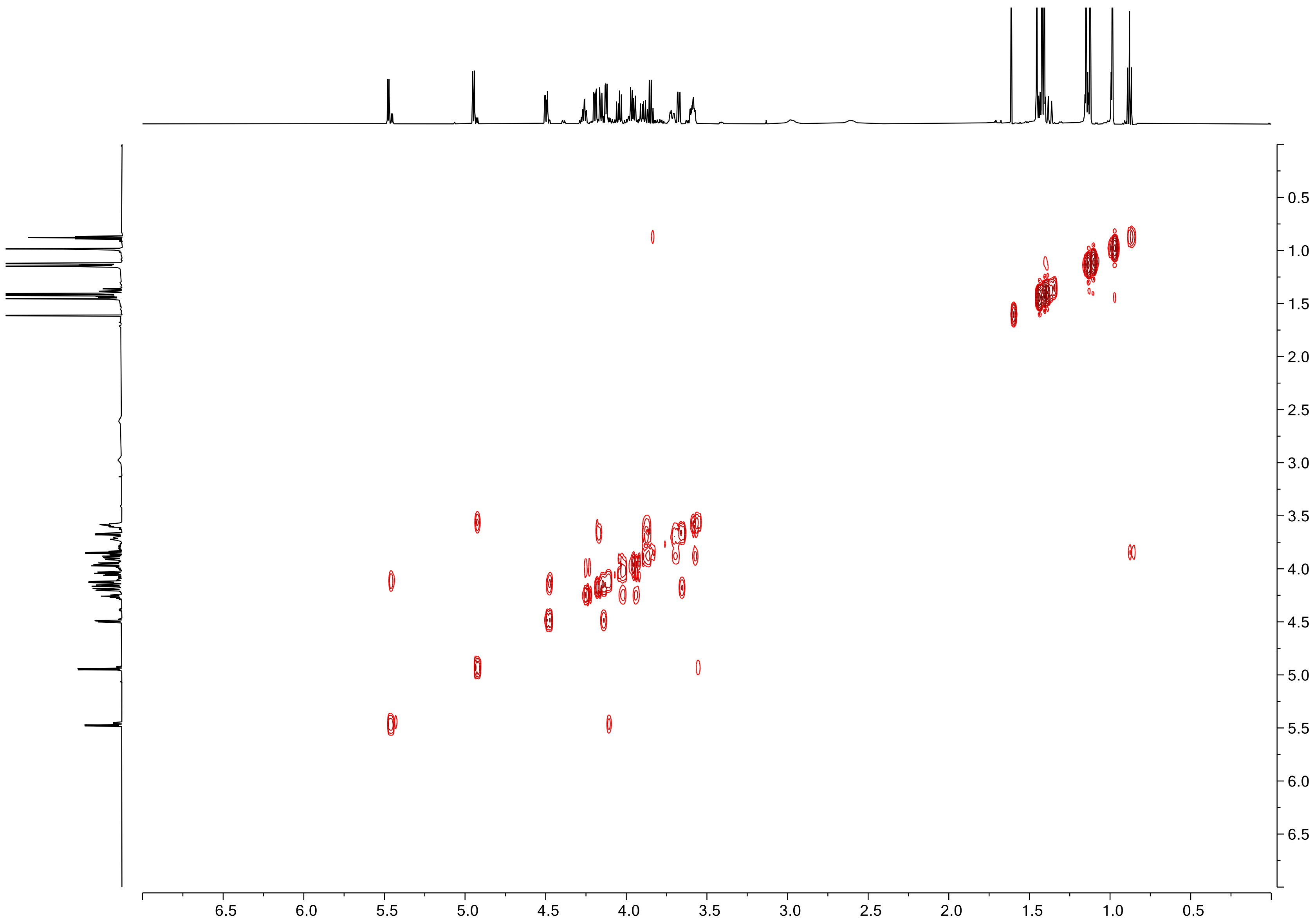

COSY spectrum of **15** from a mixture of E/Z vinylic ethers (600 MHz, C_6D_6) – expansion

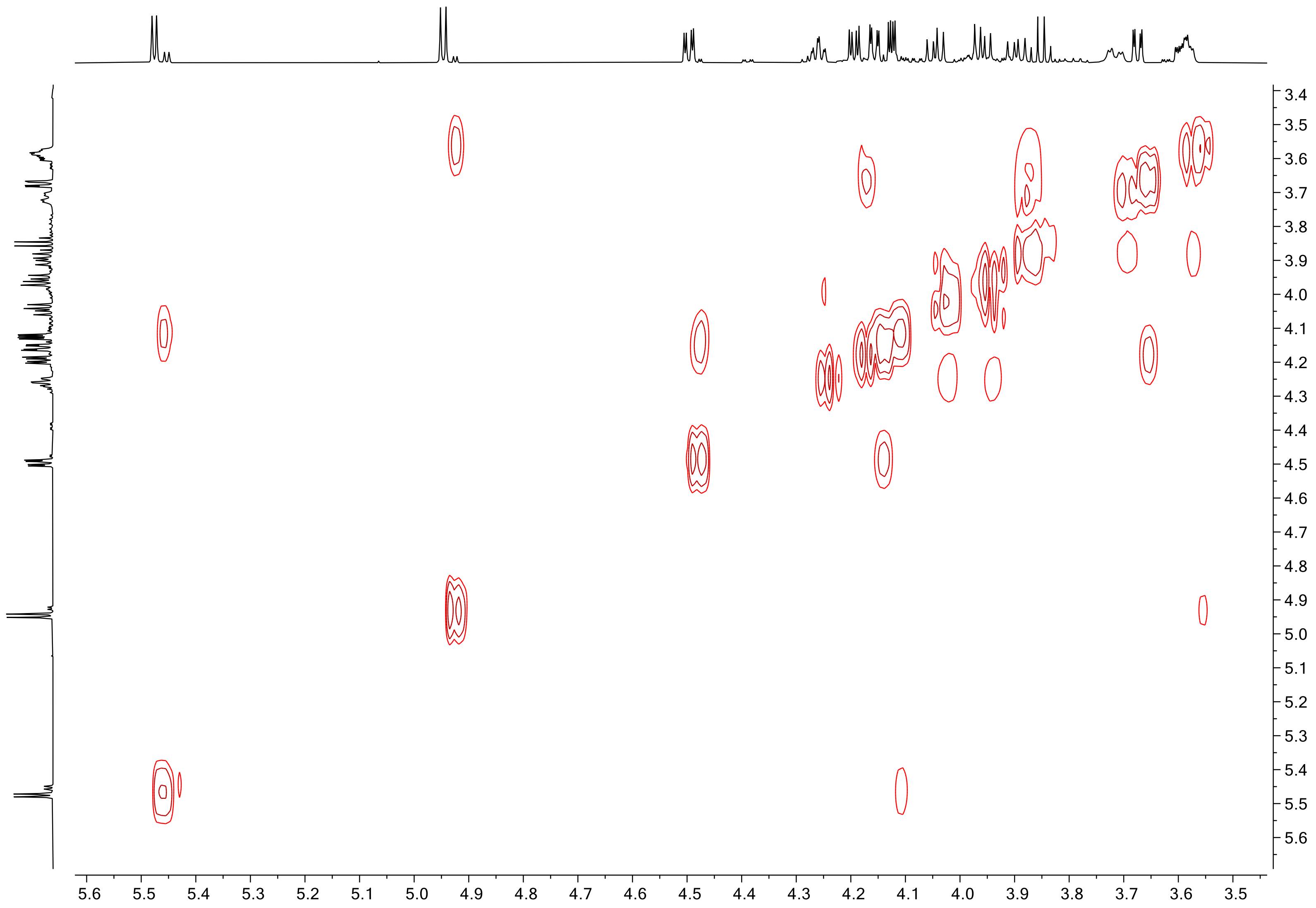

HSQC spectrum of **15** from a mixture of E/Z vinylic ethers (600 MHz, C_6D_6) – full spectrum

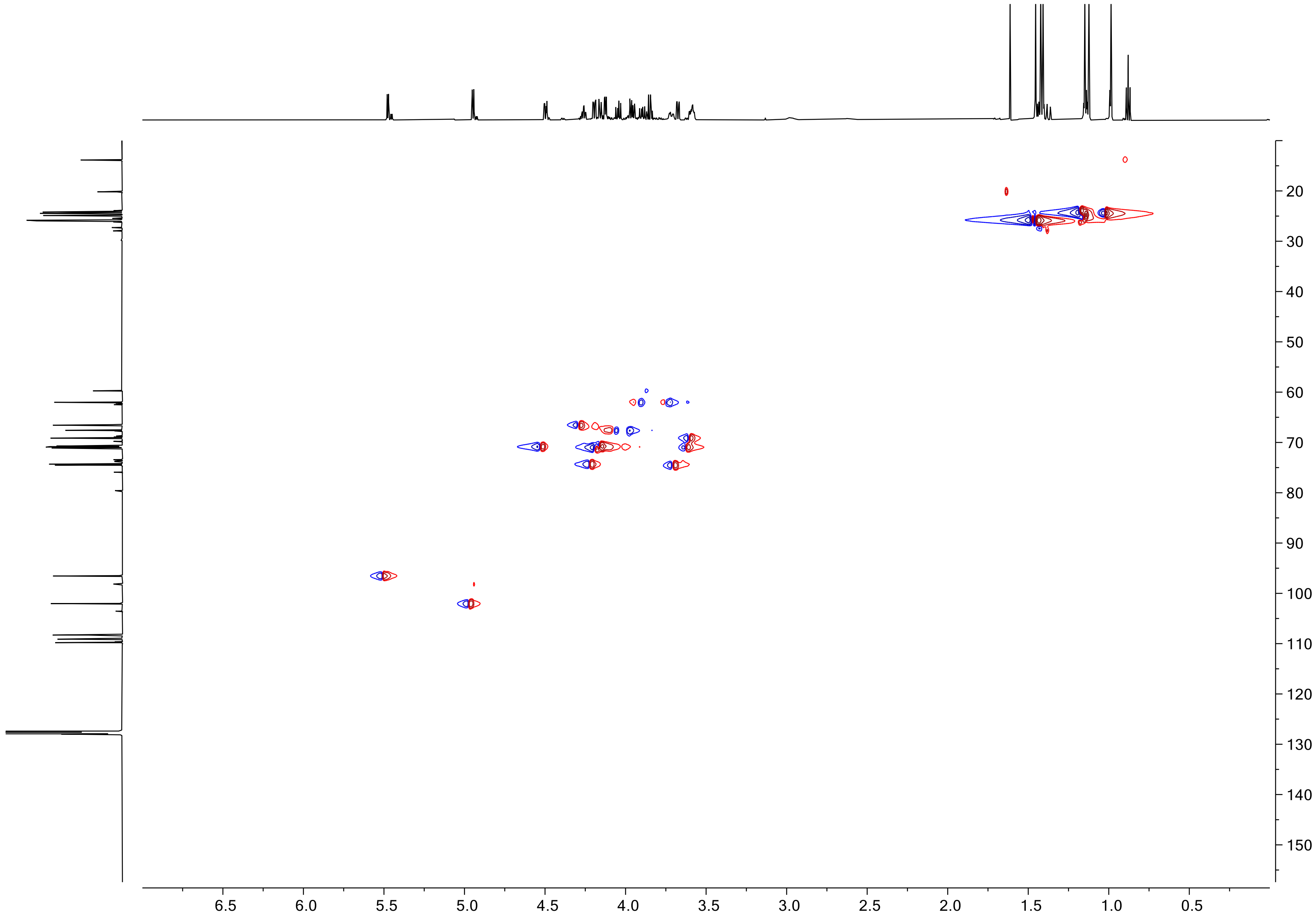

HSQC spectrum of **15** from a mixture of E/Z vinylic ethers (600 MHz, C_6D_6) – expansion

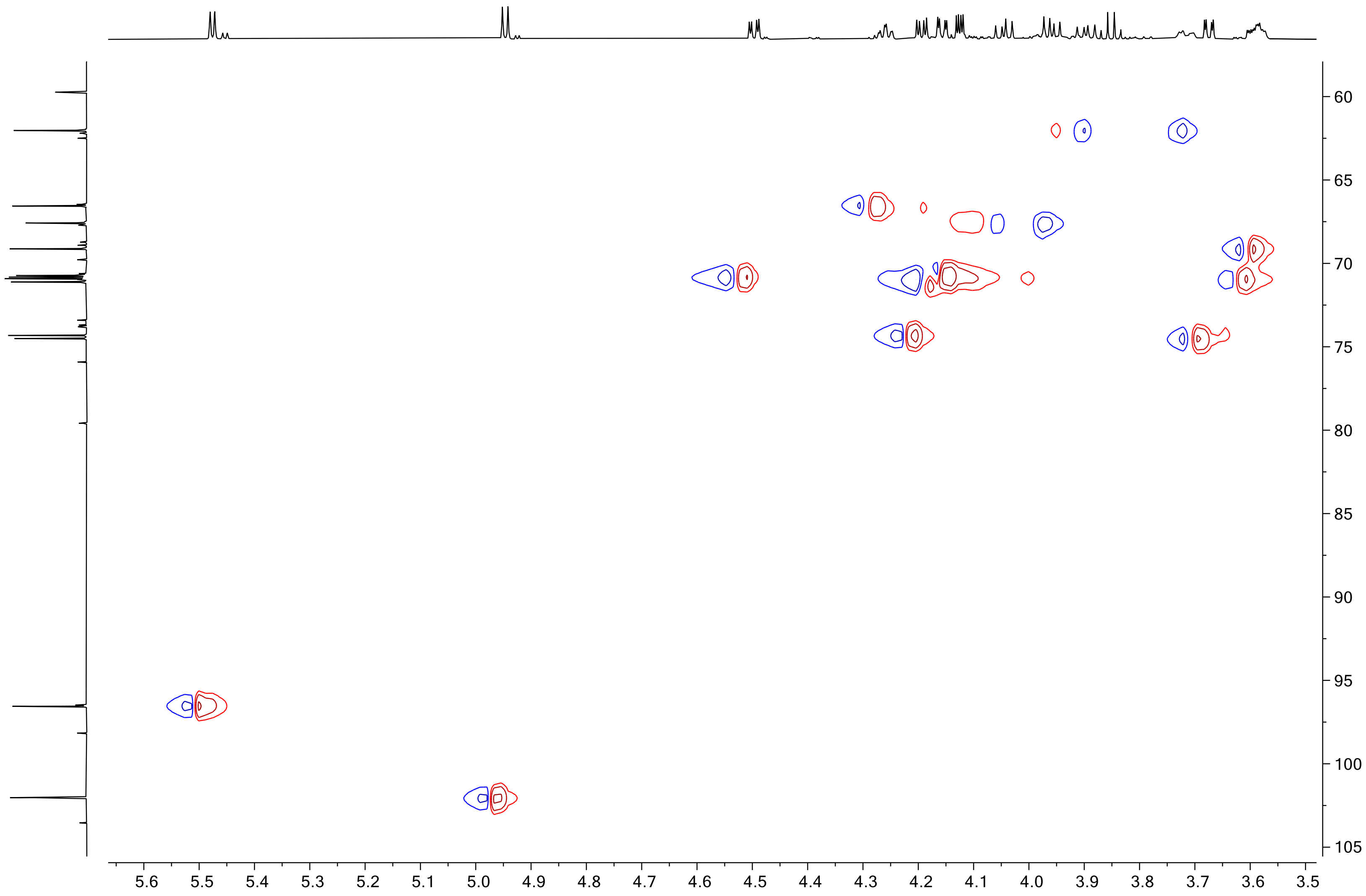

^{1}H NMR Spectrum of **18** (400 MHz, C_6D_6)

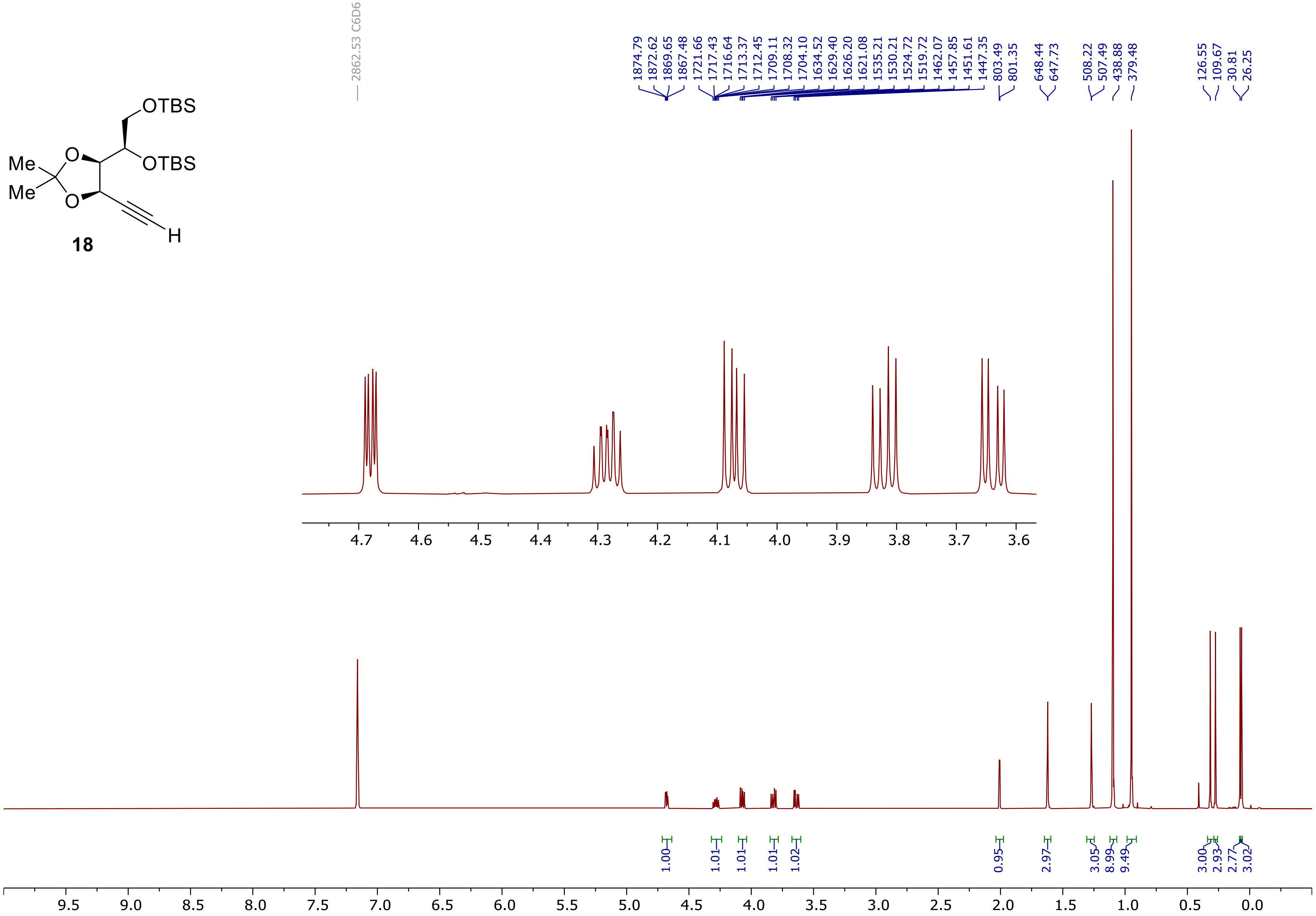

$^{13}C\{^{1}H\}$ NMR Spectrum of **18** (101 MHz, C_6D_6)

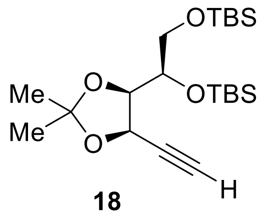

18

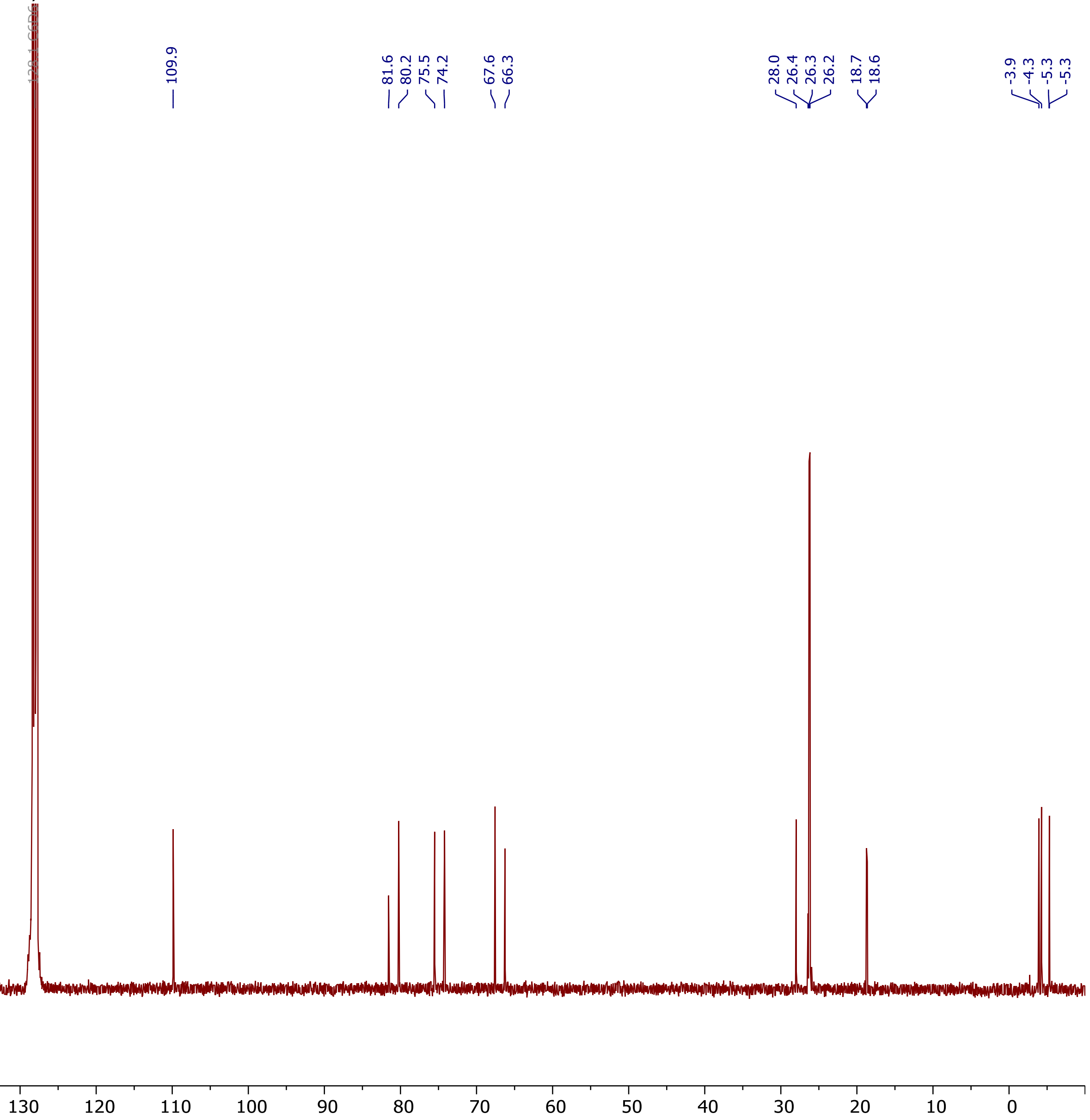

^{1}H NMR Spectrum of (*E*)-**19** (400 MHz, C_6D_6)

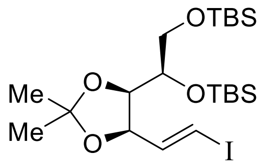

(*E*)-**19**

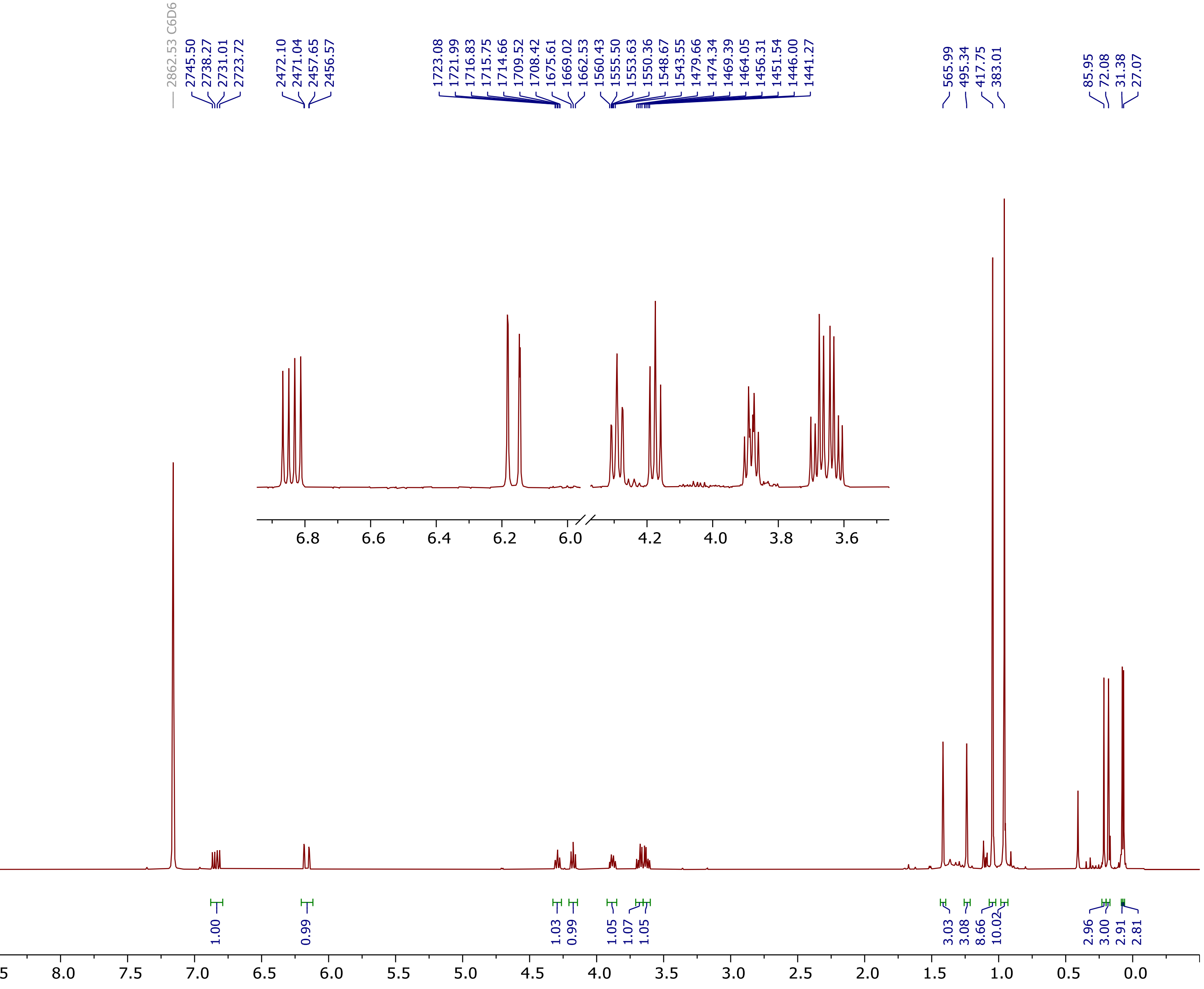

$^{13}C\{^{1}H\}$ NMR Spectrum of (*E*)-**19** (101 MHz, C_6D_6)

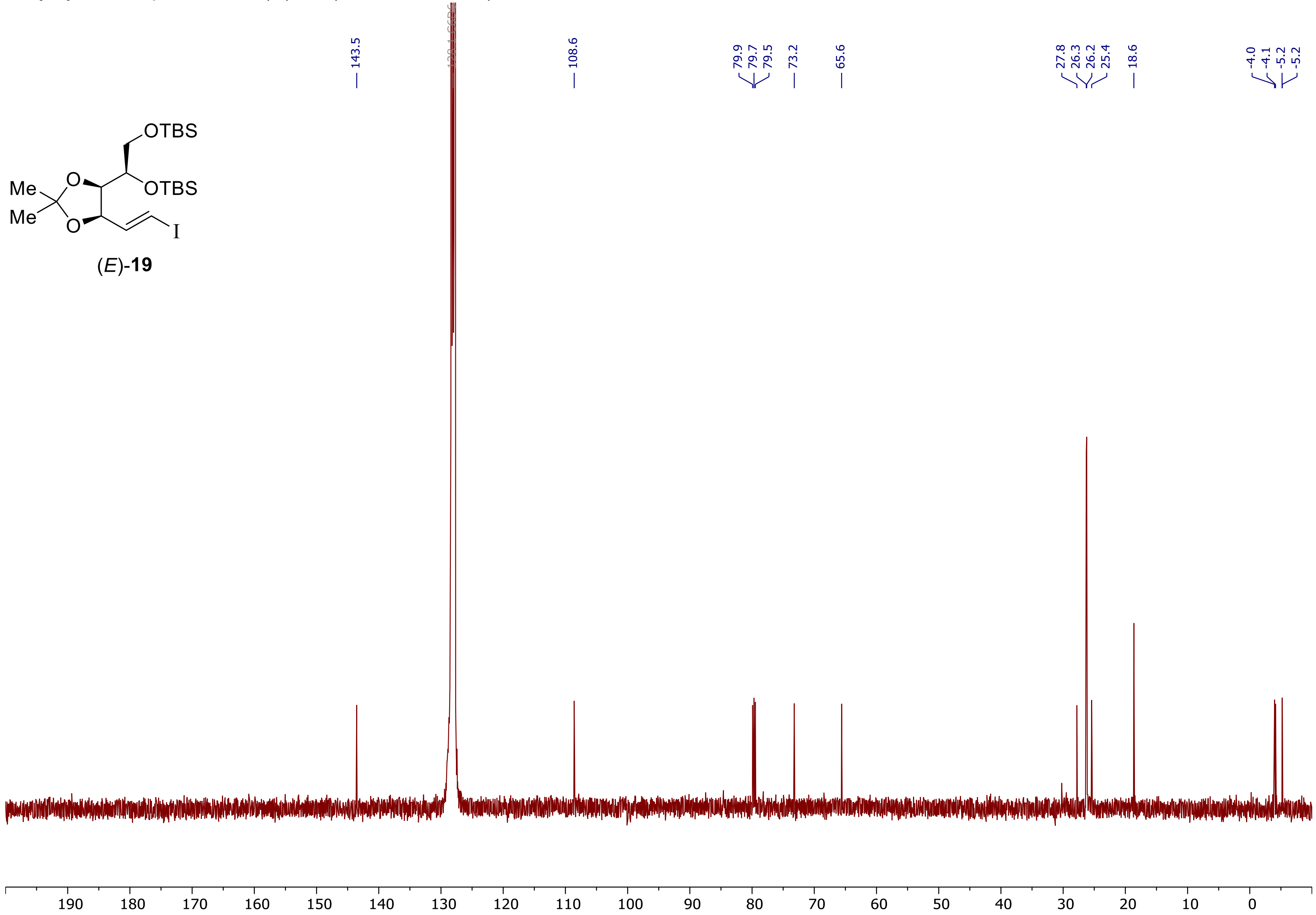

^{1}H NMR Spectrum of **S22** (400 MHz, C_6D_6)

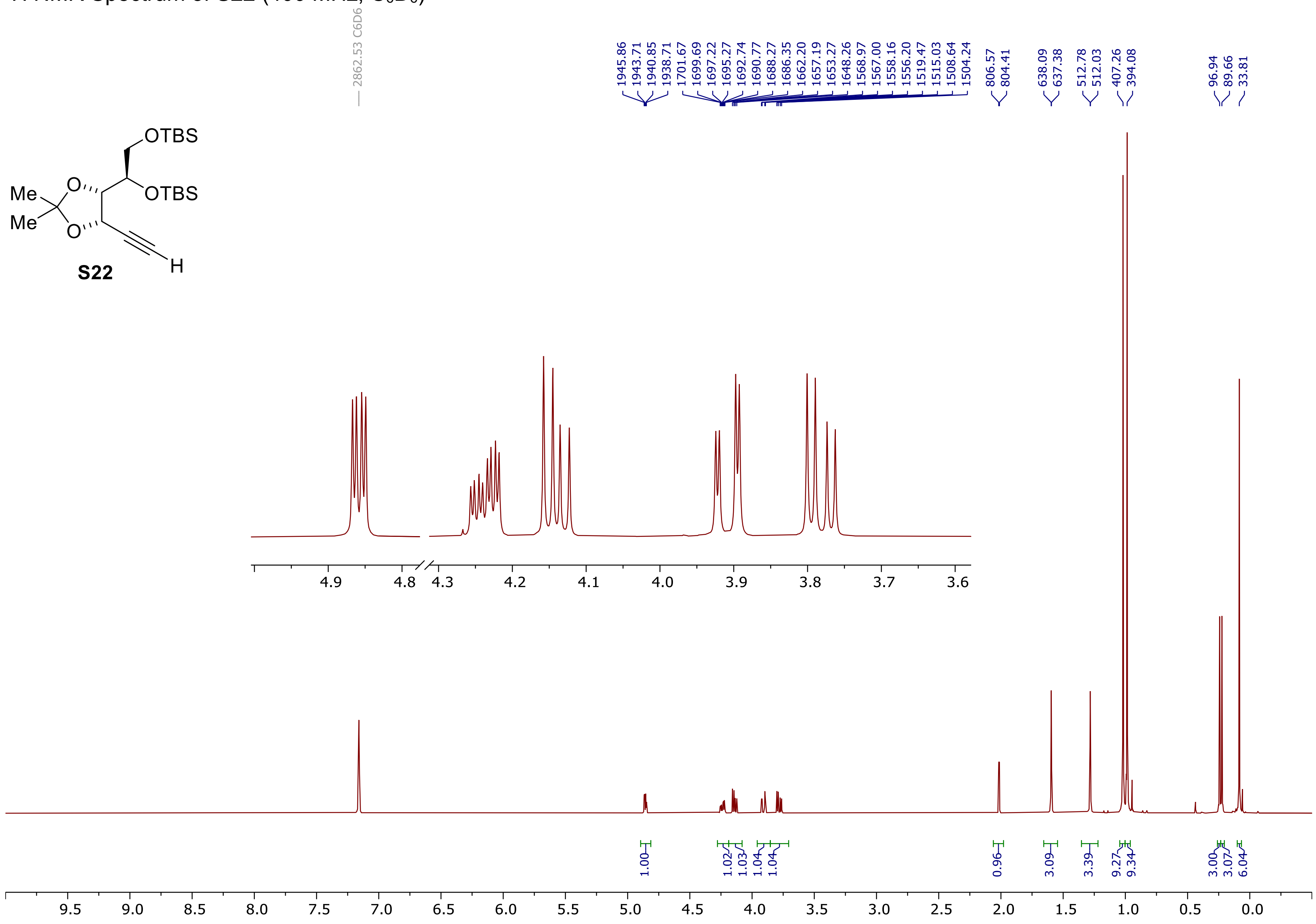

$^{13}C\{^1H\}$ NMR Spectrum of **S22** (101 MHz, C_6D_6)

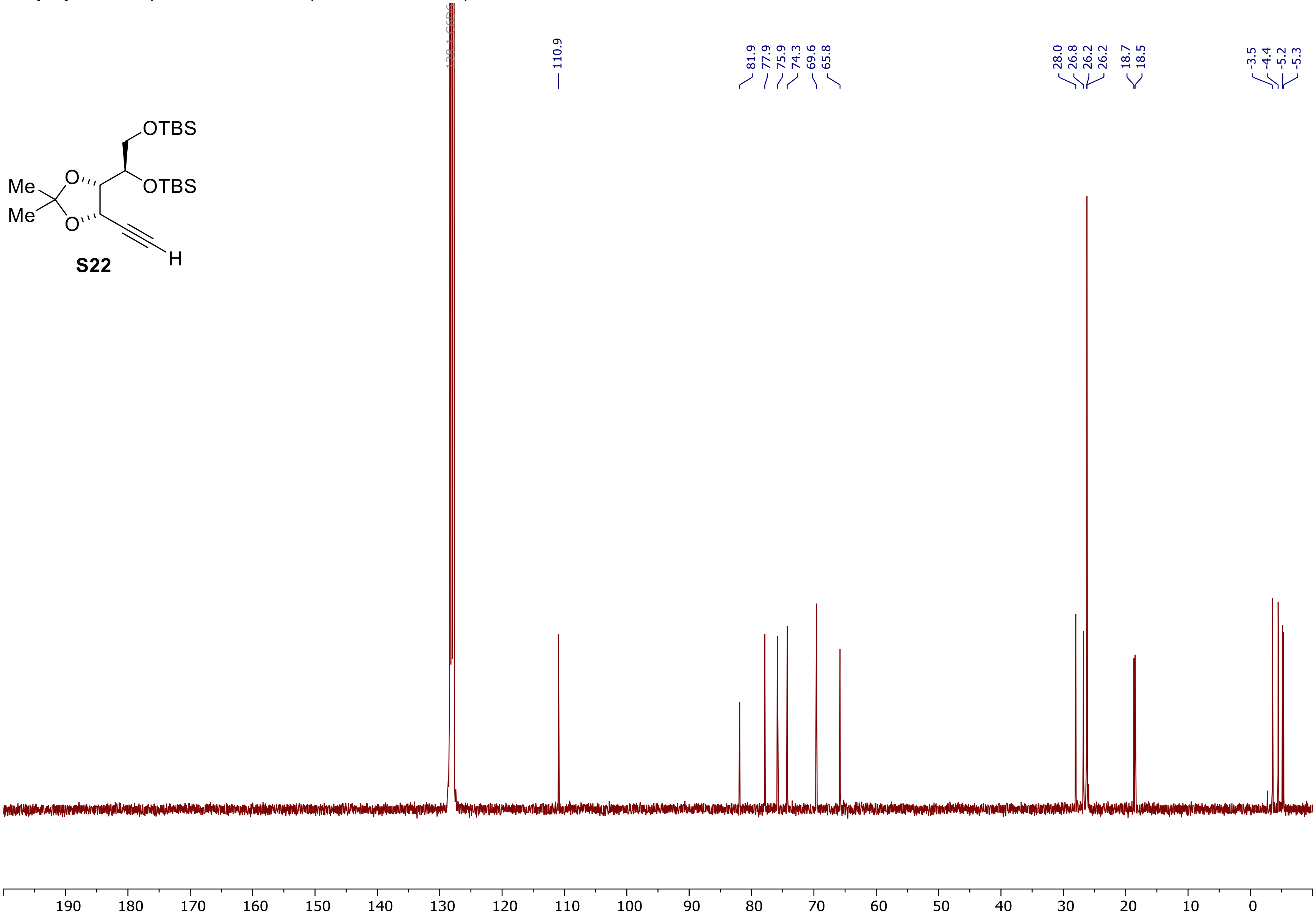

^{1}H NMR Spectrum of (*E*)-**21** (400 MHz, C_6D_6)

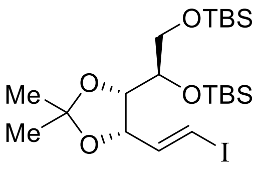

(*E*)-**21**

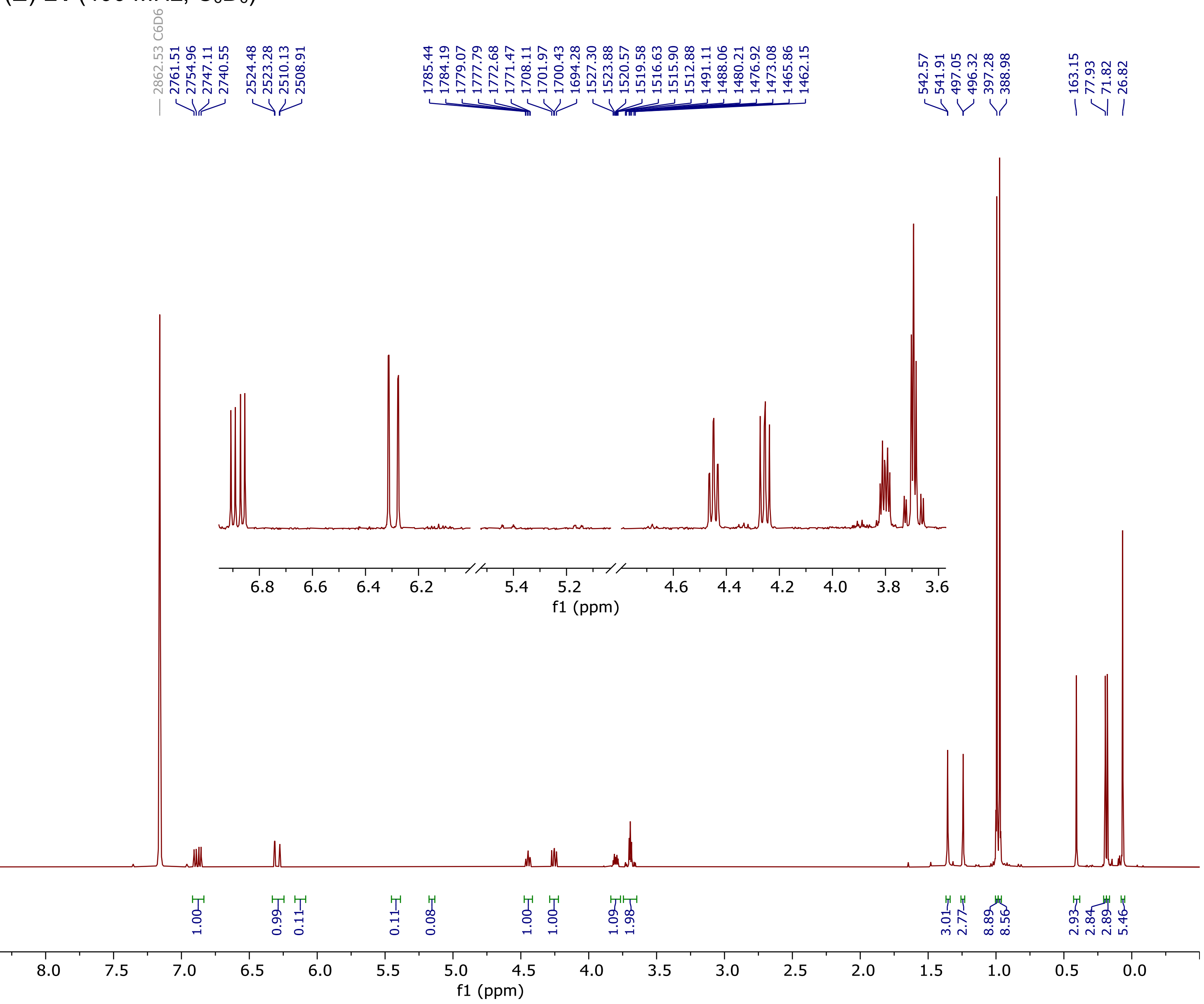

$^{13}C\{^1H\}$ NMR Spectrum of (*E*)-**21** (101 MHz, C_6D_6)

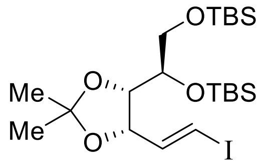

(*E*)-**21**

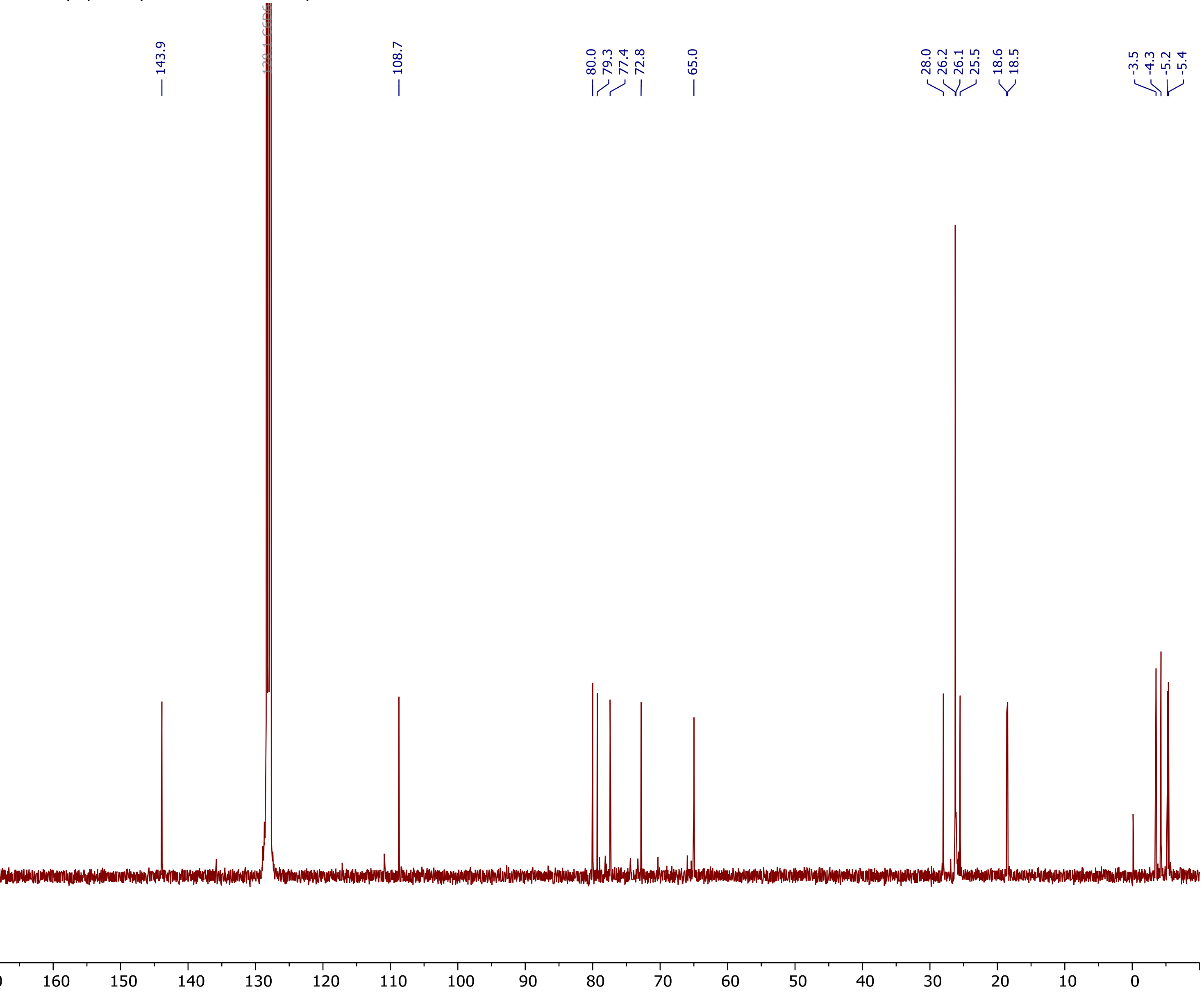

^{1}H NMR Spectrum of **S25** (400 MHz, C_6D_6), with minor amounts of **S22**

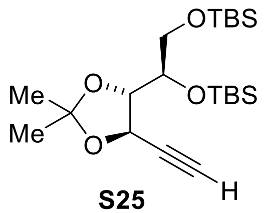

S25

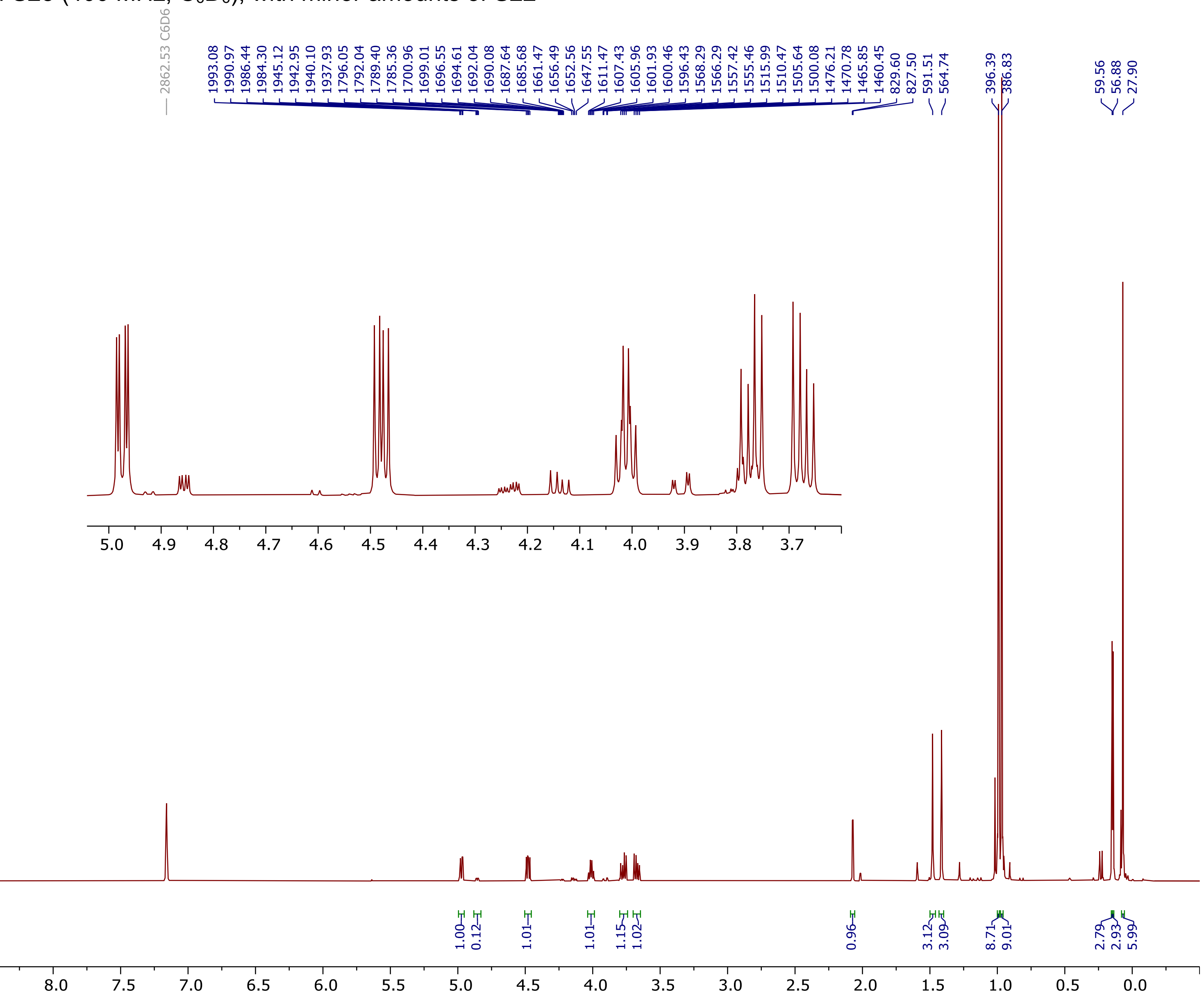

$^{13}C\{^{1}H\}$ NMR Spectrum of **S25** (101 MHz, C_6D_6), with minor amounts of **S22**

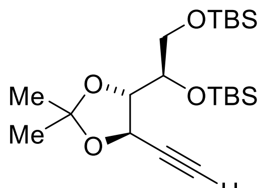

S25

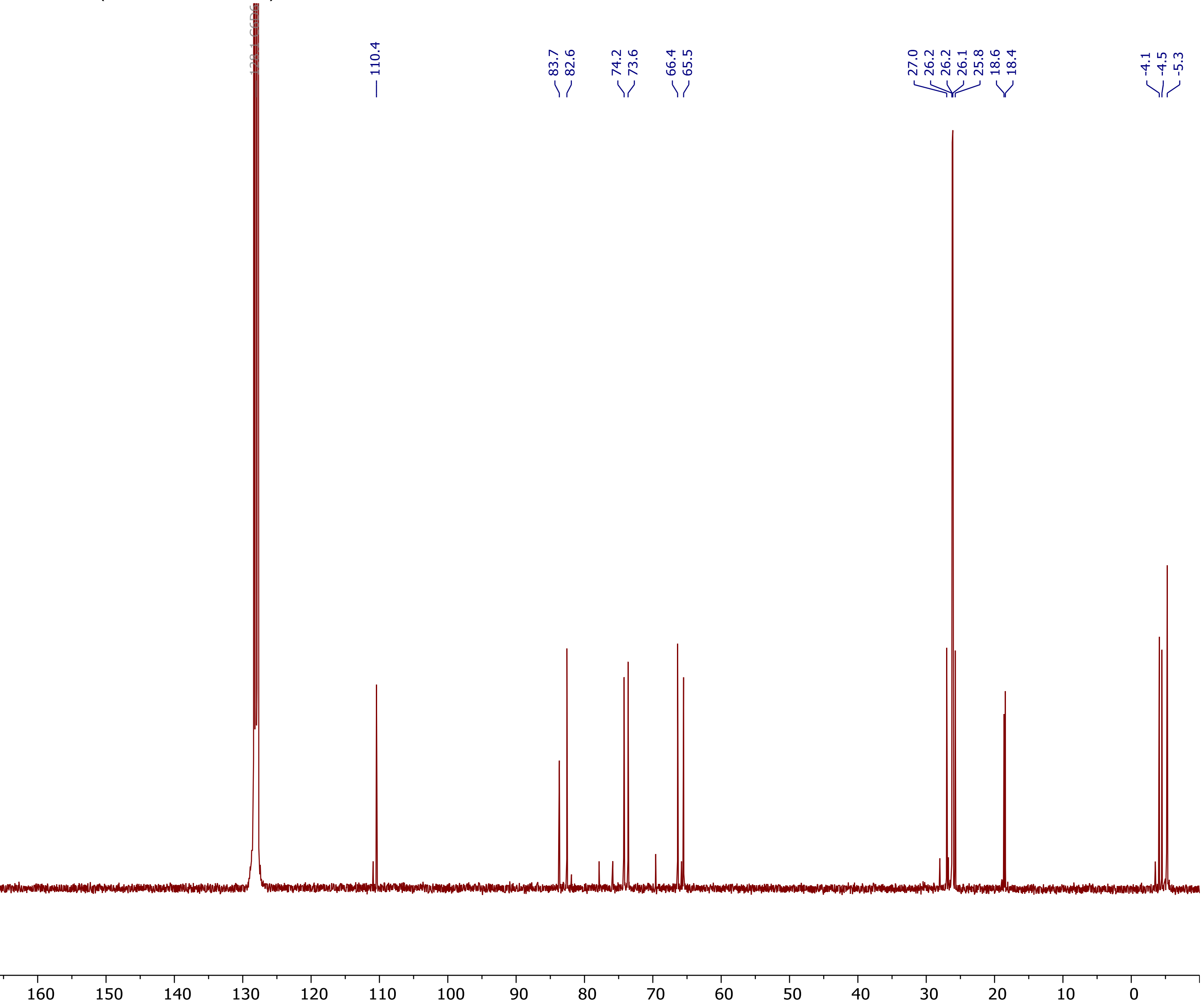

^{1}H NMR Spectrum of (*E*)-**22** (400 MHz, C_6D_6)

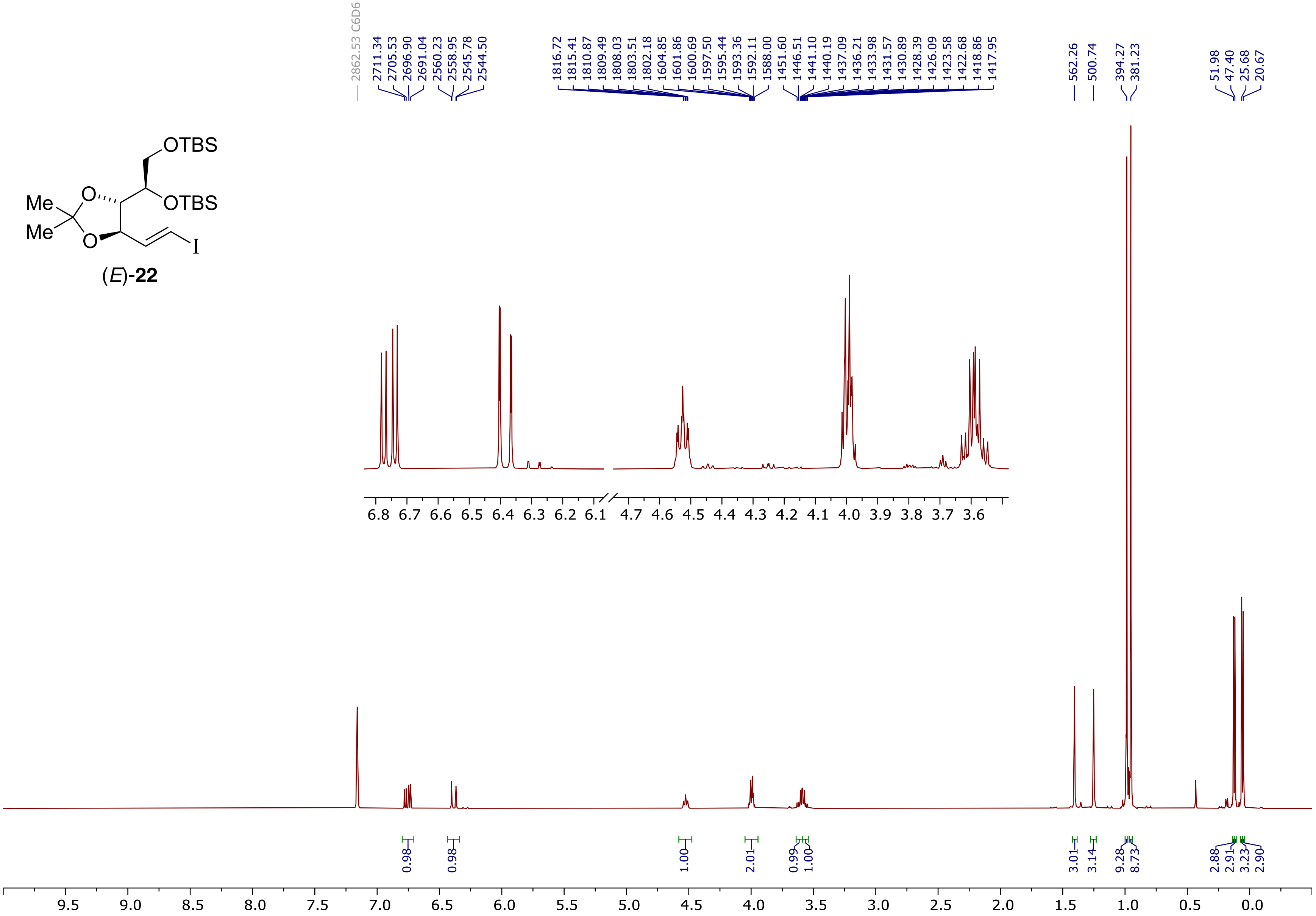

$^{13}C\{^1H\}$ NMR Spectrum of (*E*)-**22** (101 MHz, C_6D_6)

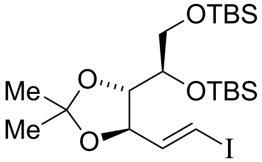

(*E*)-**22**

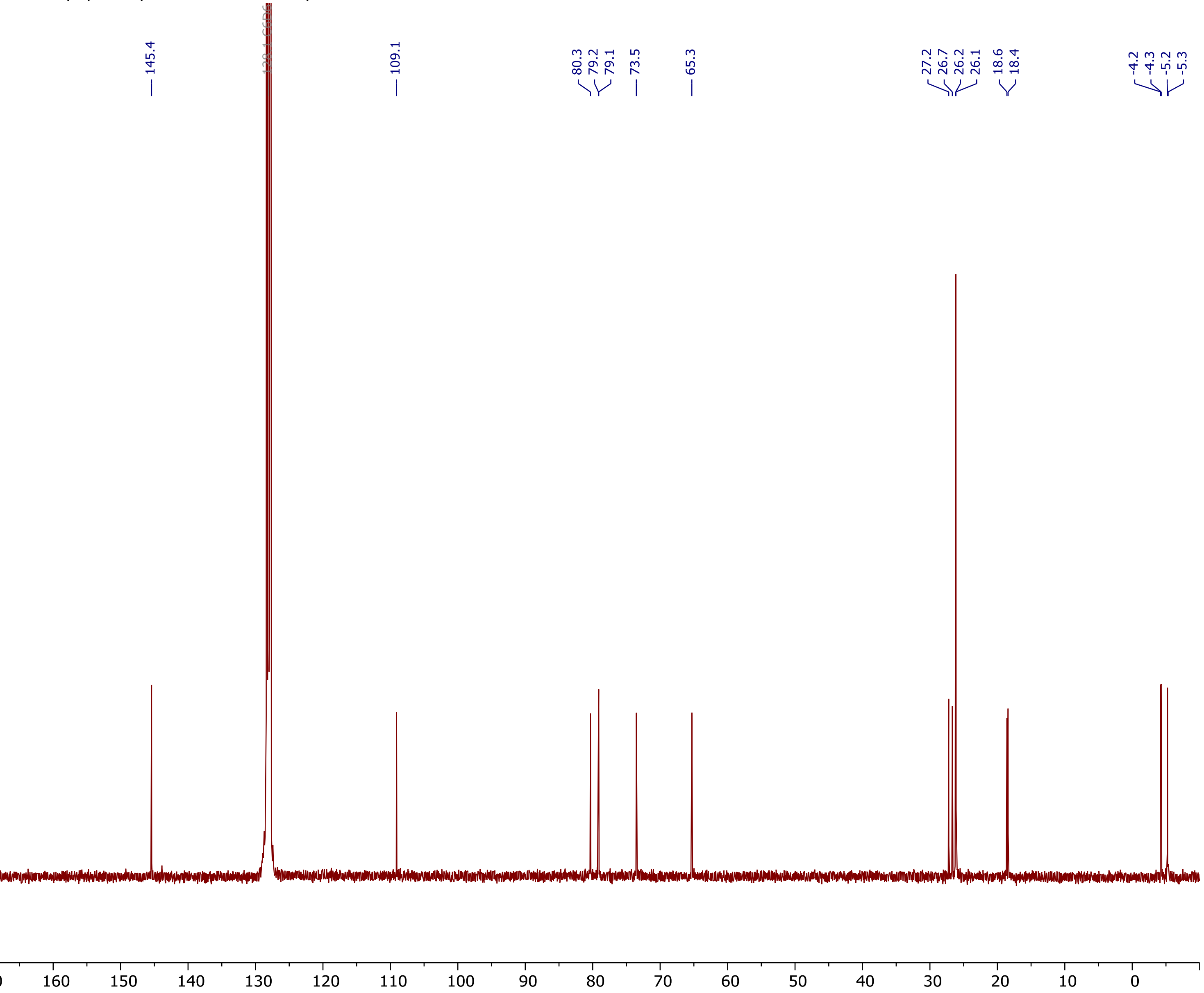

^{1}H NMR Spectrum of (*E*)-**10** (800 MHz, C_6D_6) – full spectrum

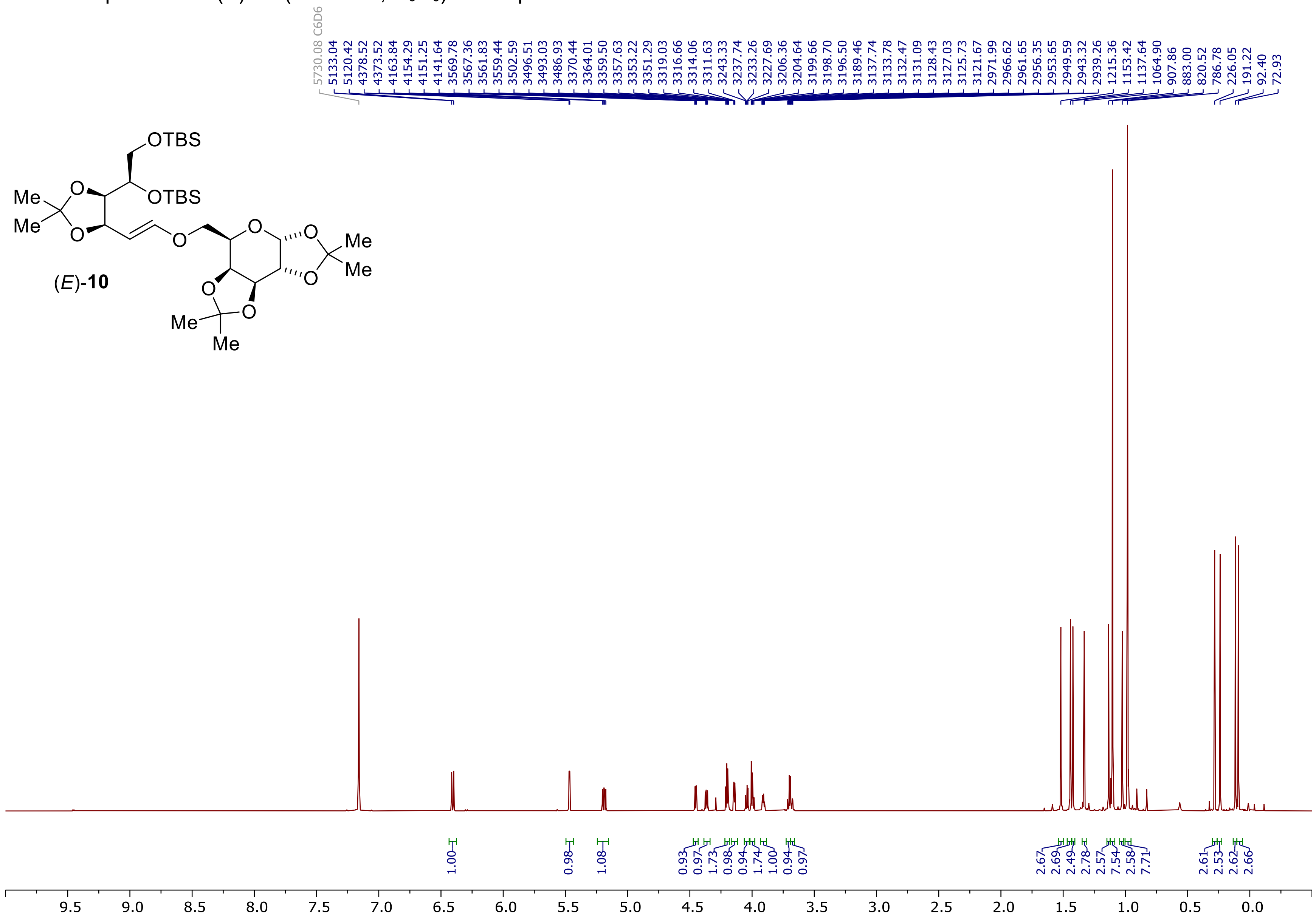

^{1}H NMR Spectrum of (*E*)-**10** (800 MHz, C_6D_6) – expansion

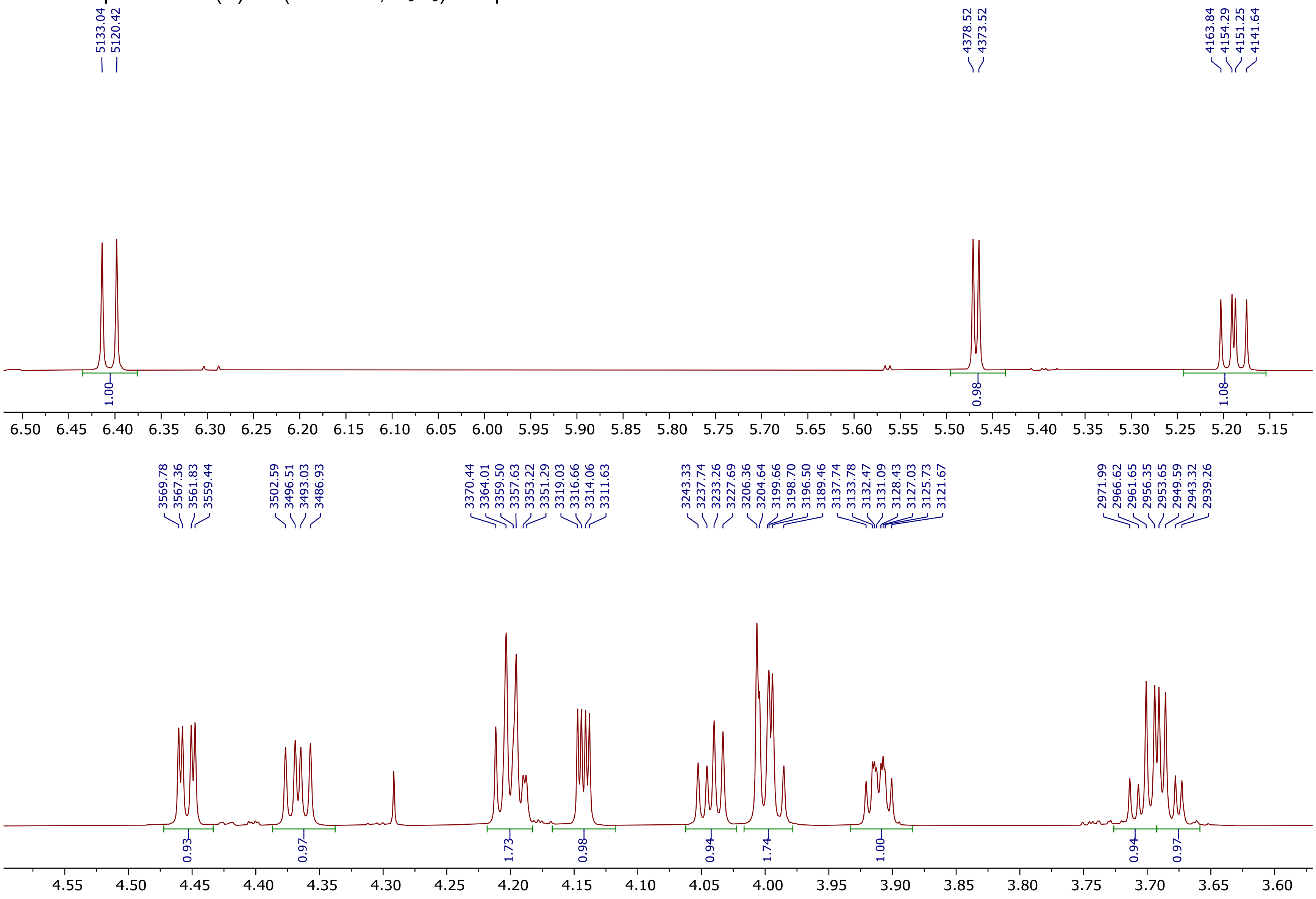

$^{13}C\{^{1}H\}$ NMR Spectrum of (*E*)-**10** (101 MHz, C_6D_6)

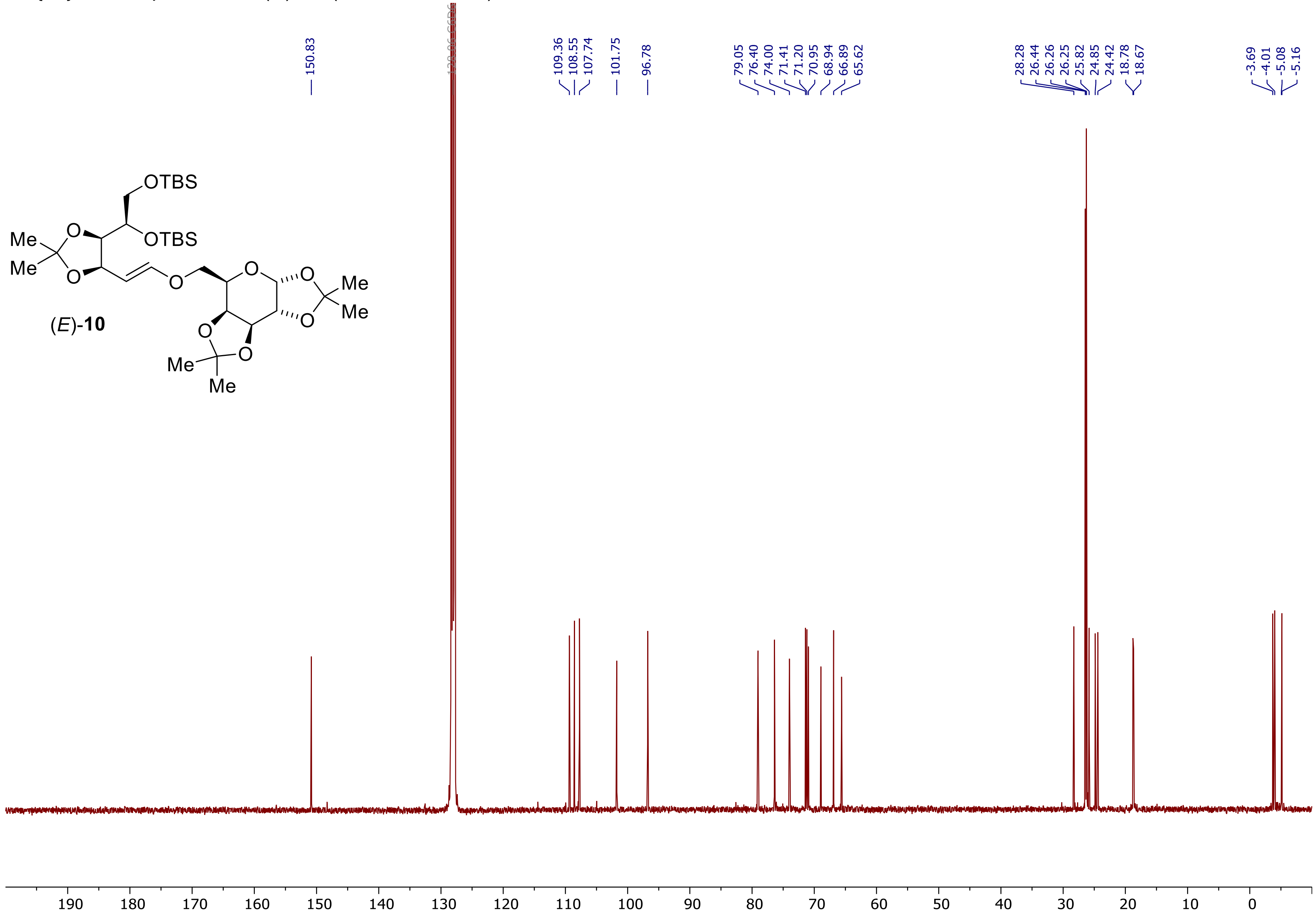

COSY spectrum of (*E*)-**10** (800 MHz, C_6D_6) – full spectrum

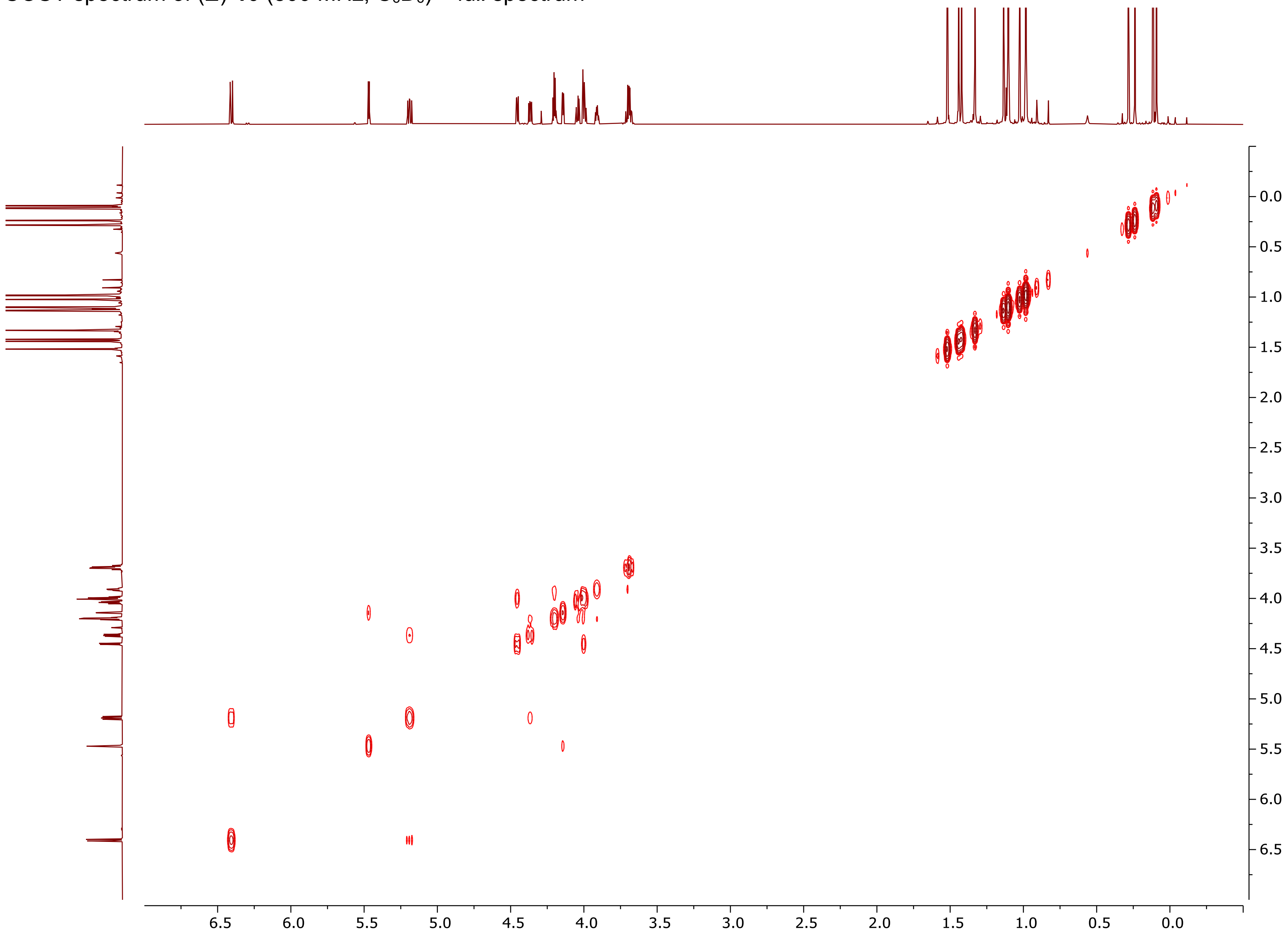

COSY spectrum of (*E*)-**10** (800 MHz, C_6D_6) – expansion

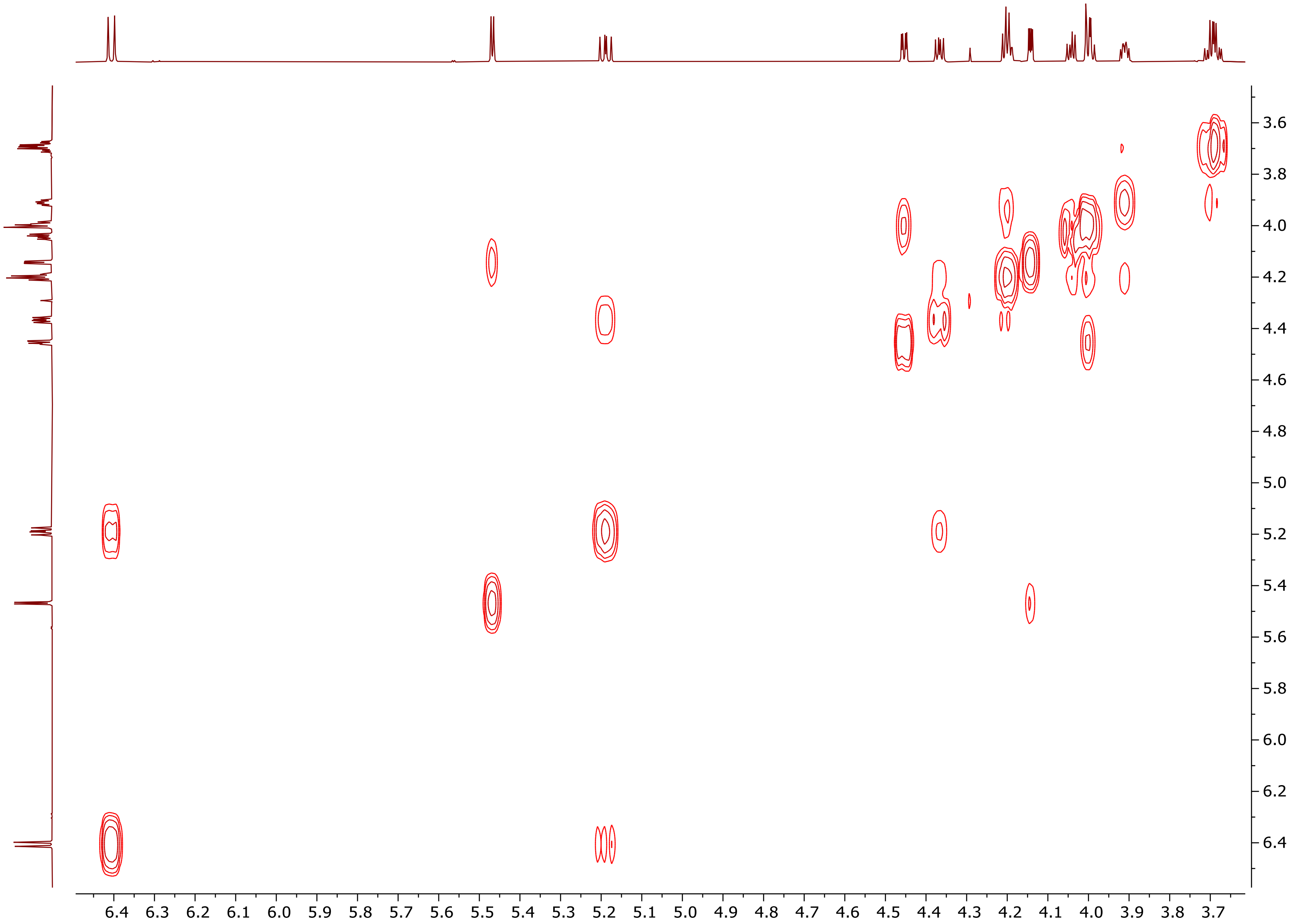

HSQC spectrum of (*E*)**-10** (800 MHz, C_6D_6) – full spectrum

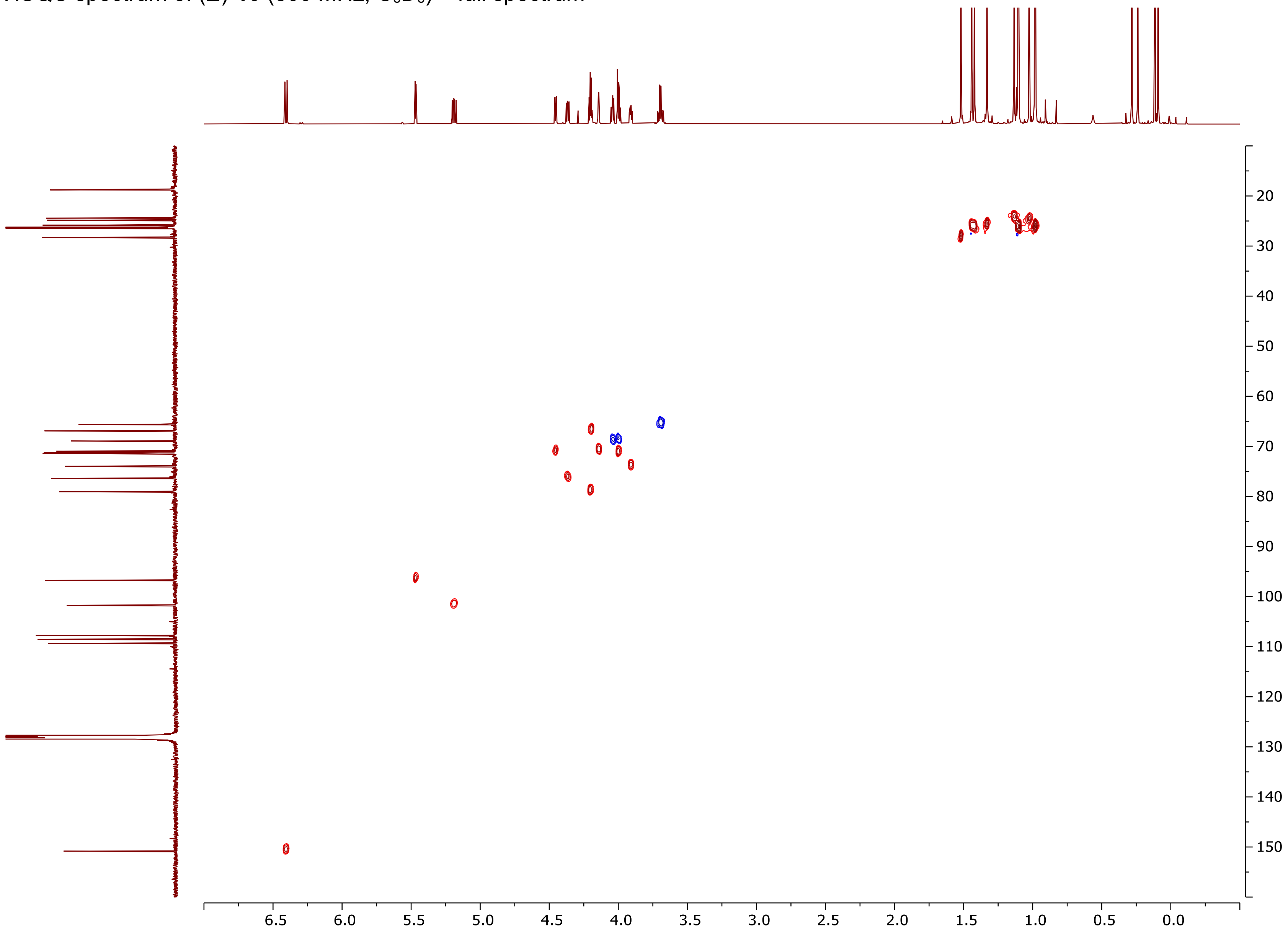

HSQC spectrum of (*E*)**-10** (800 MHz, C_6D_6) – expansion

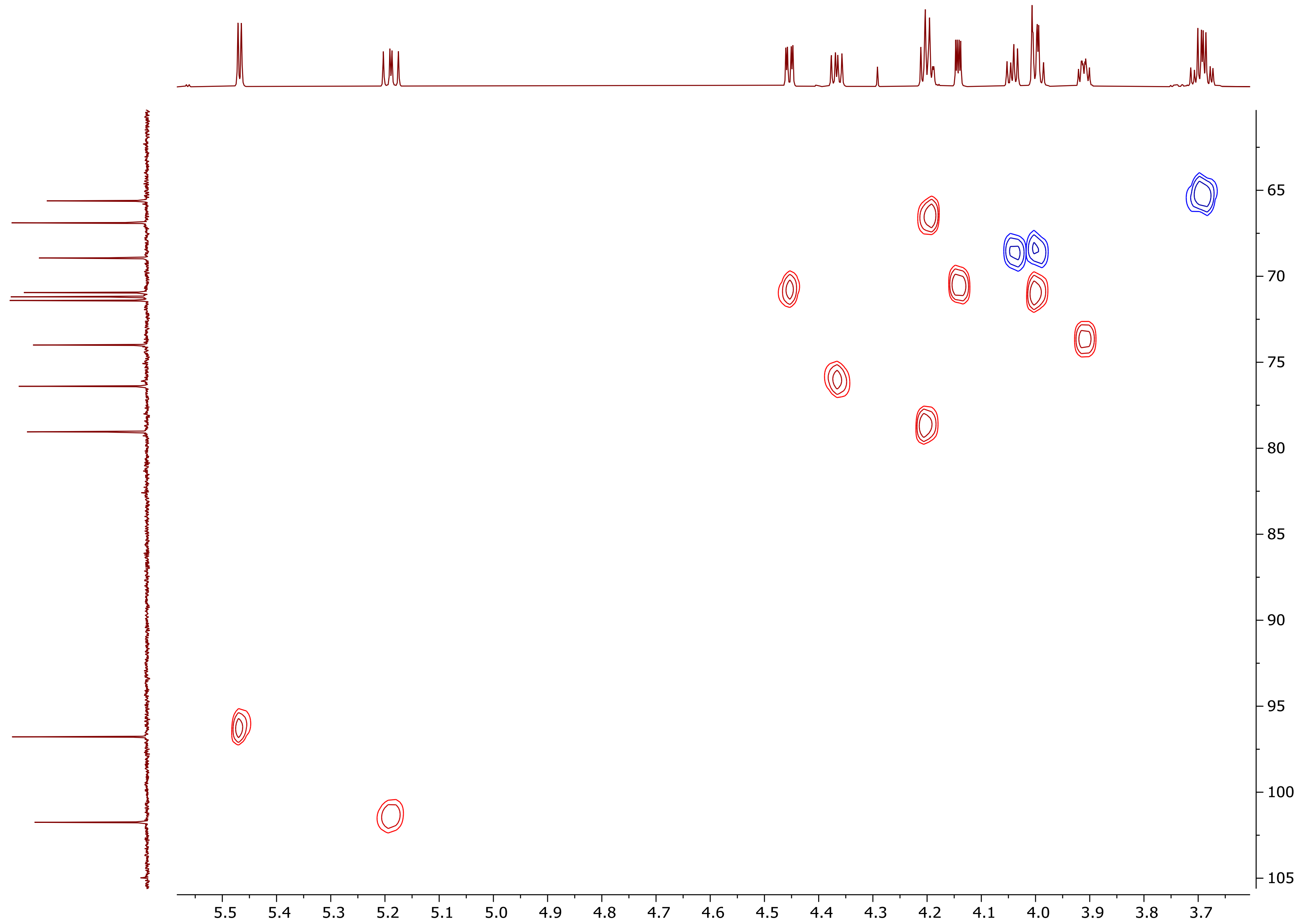

HMBC spectrum of (*E*)**-10** (800 MHz, C_6D_6) – full spectrum

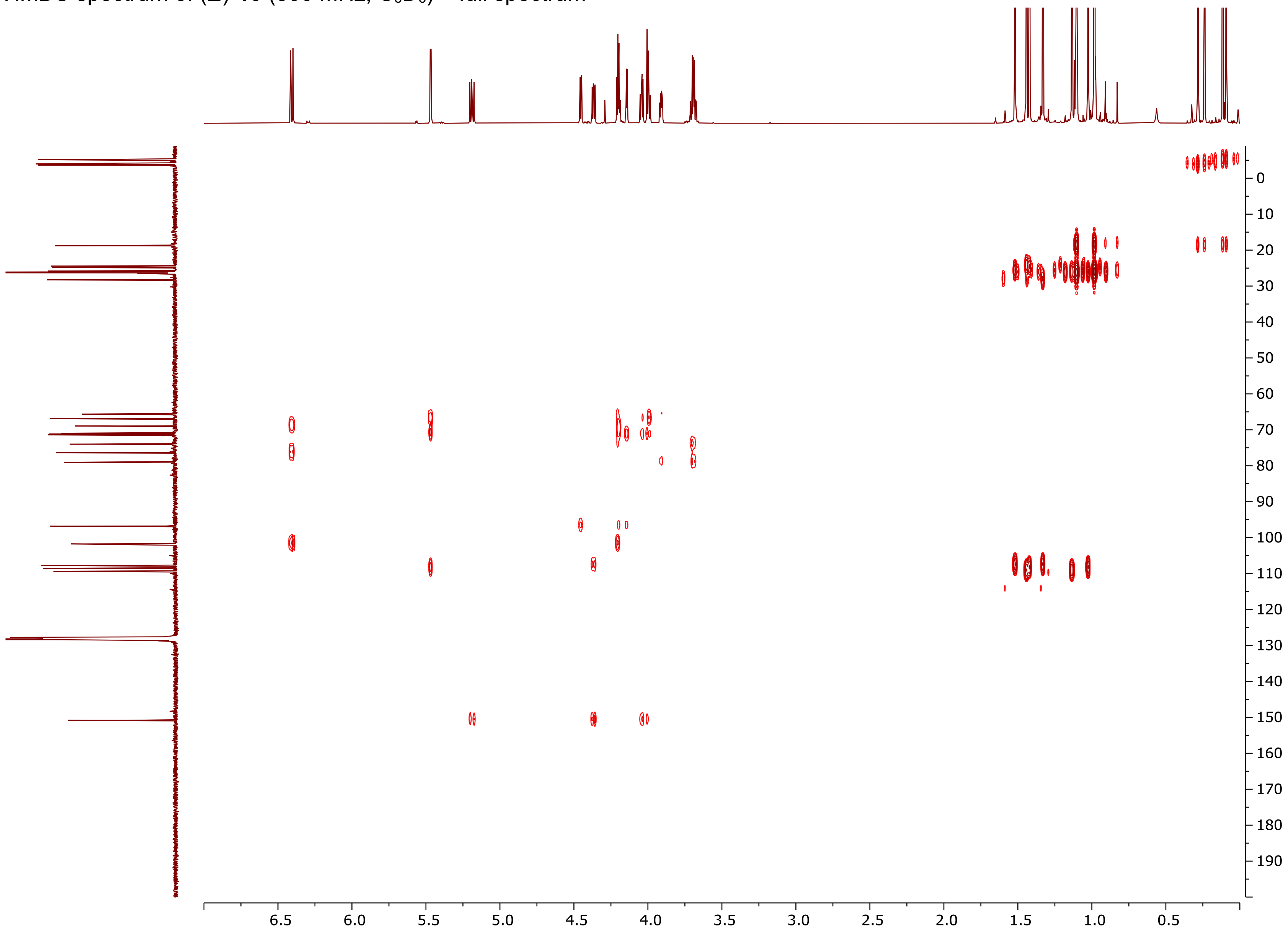

HMBC spectrum of (*E*)**-10** (800 MHz, C_6D_6) – expansion

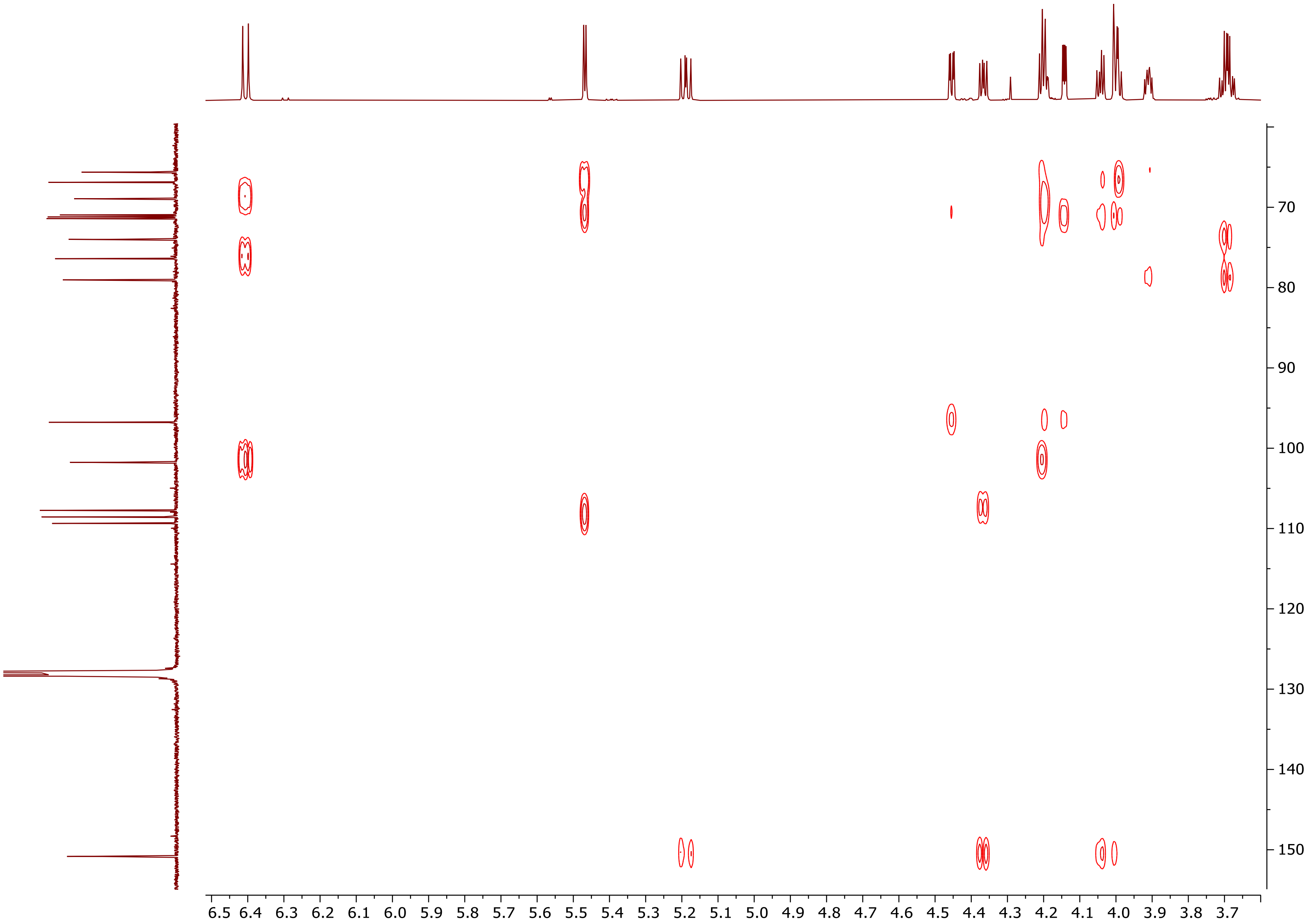

^{1}H NMR spectrum of (*E*)**-23** (800 MHz, C_6D_6) – full spectrum

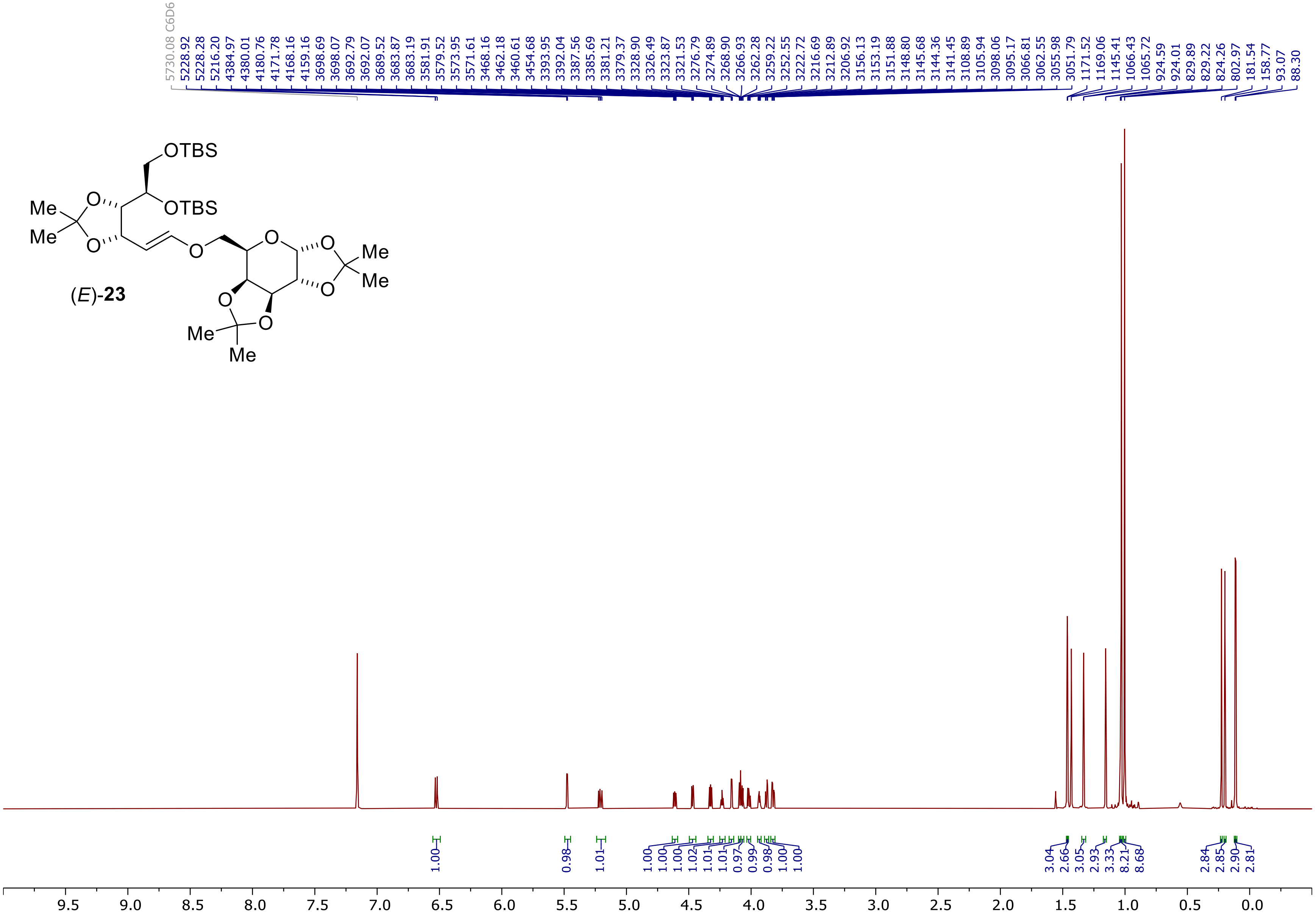

^{1}H NMR spectrum of (*E*)-**23** (800 MHz, C_6D_6) – expansion

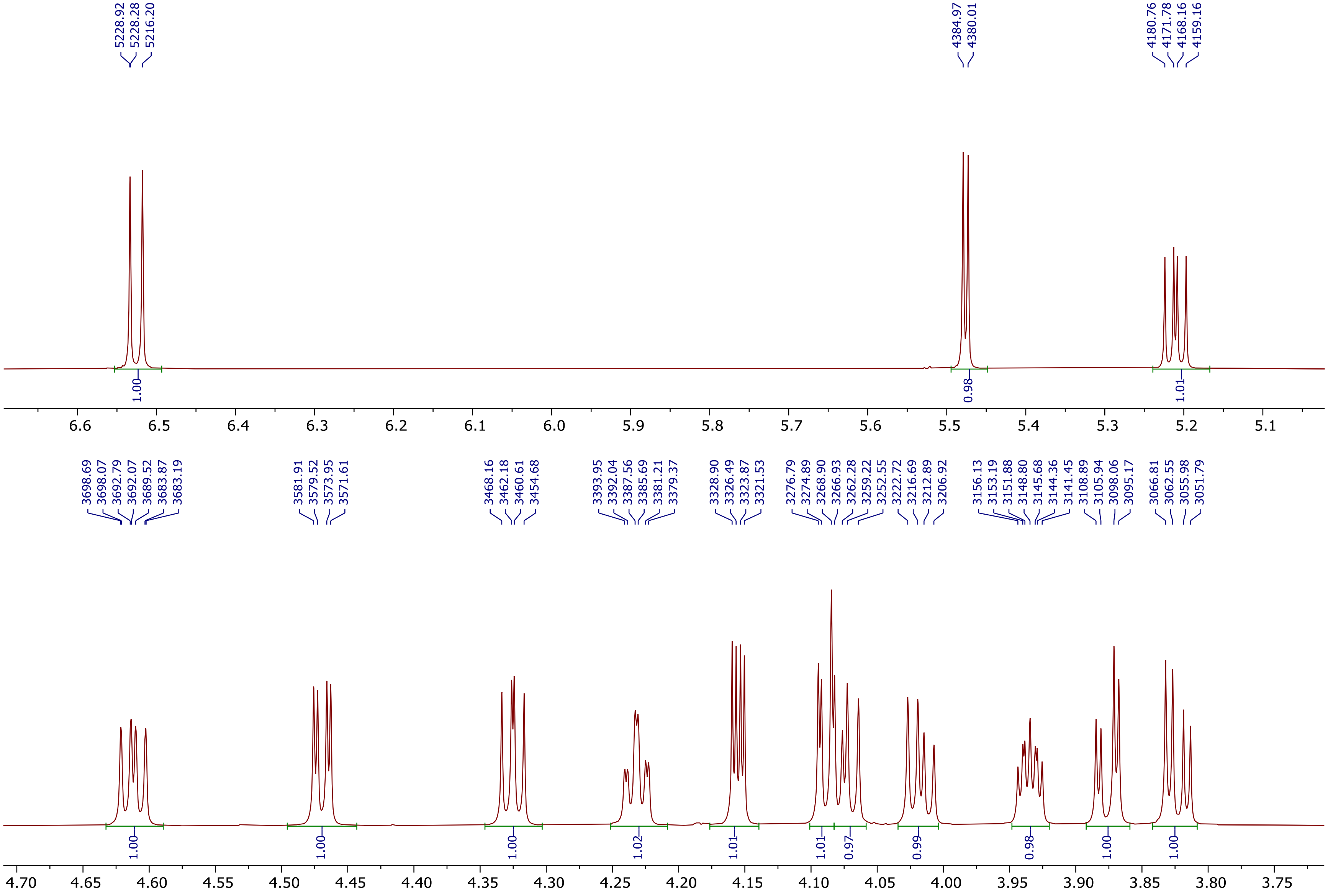

$^{13}C\{^1H\}$ NMR Spectrum of (*E*)-**23** (101 MHz, C_6D_6)

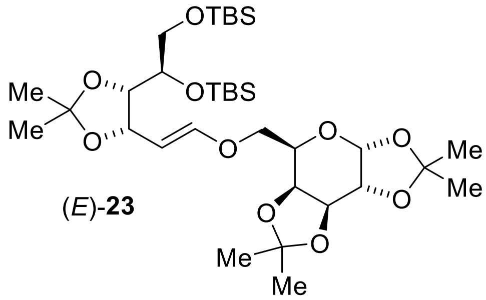

(*E*)-**23**

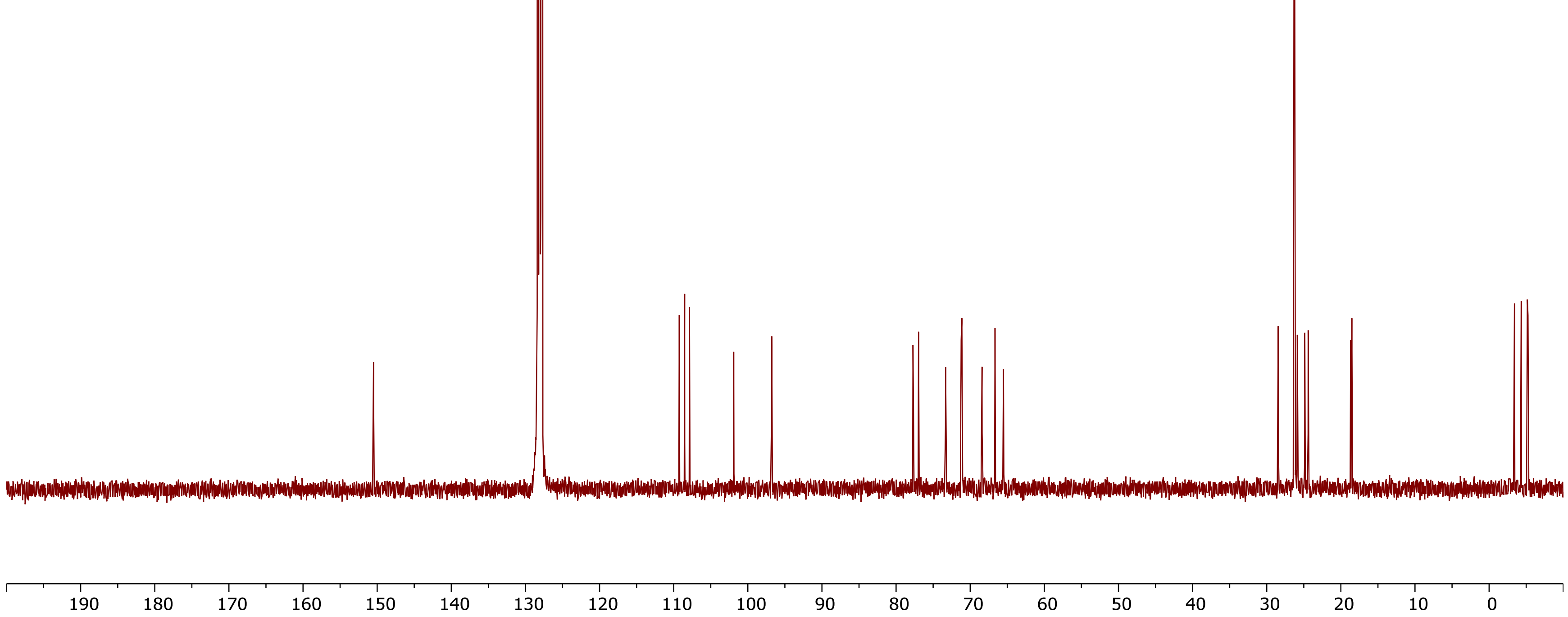

COSY spectrum of (*E*)-**23** (800 MHz, C_6D_6) – full spectrum

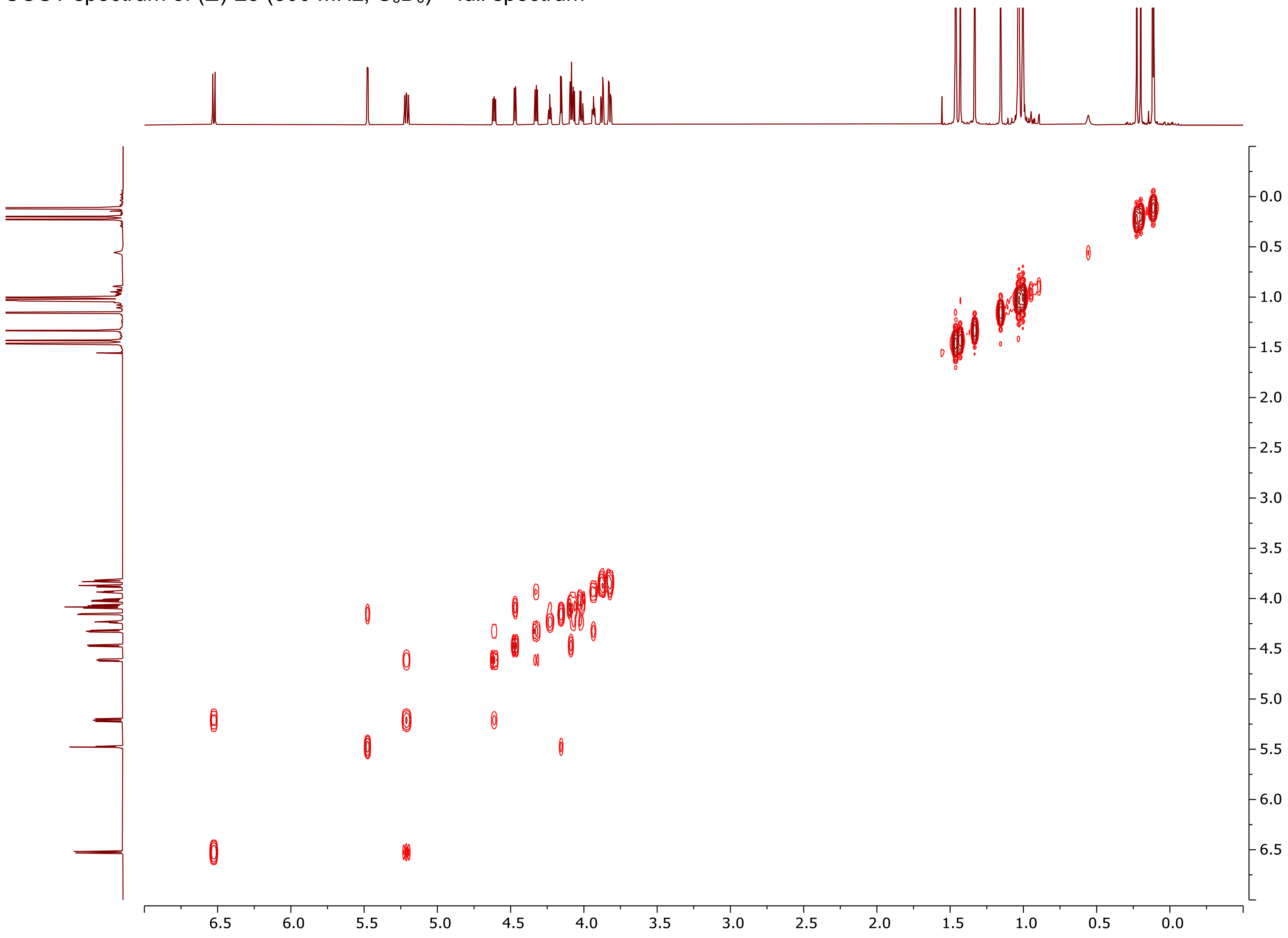

COSY spectrum of (*E*)-**23** (800 MHz, C_6D_6) – expansion

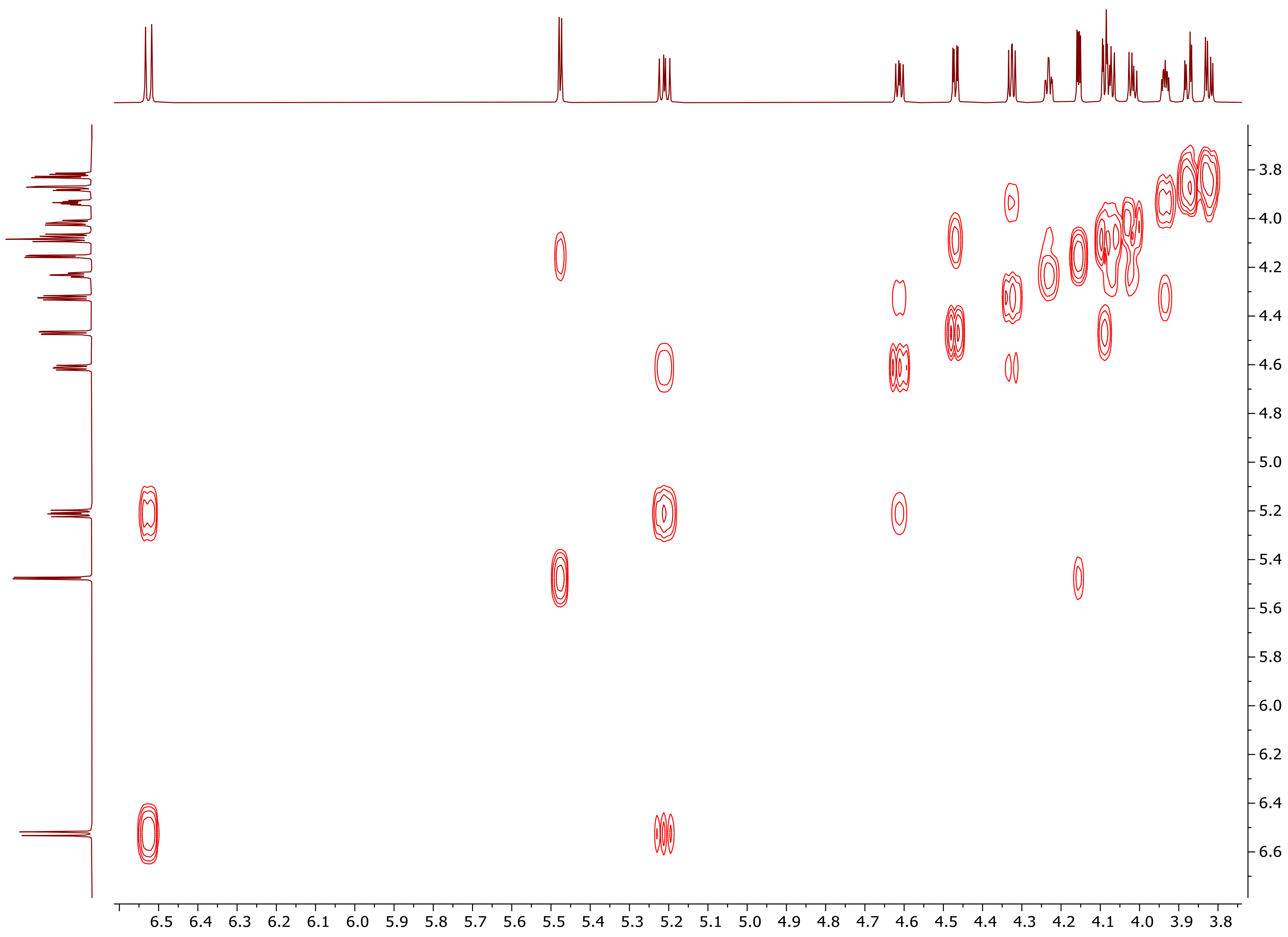

HSQC spectrum of (*E*)-**23** (800 MHz, C_6D_6) – full spectrum

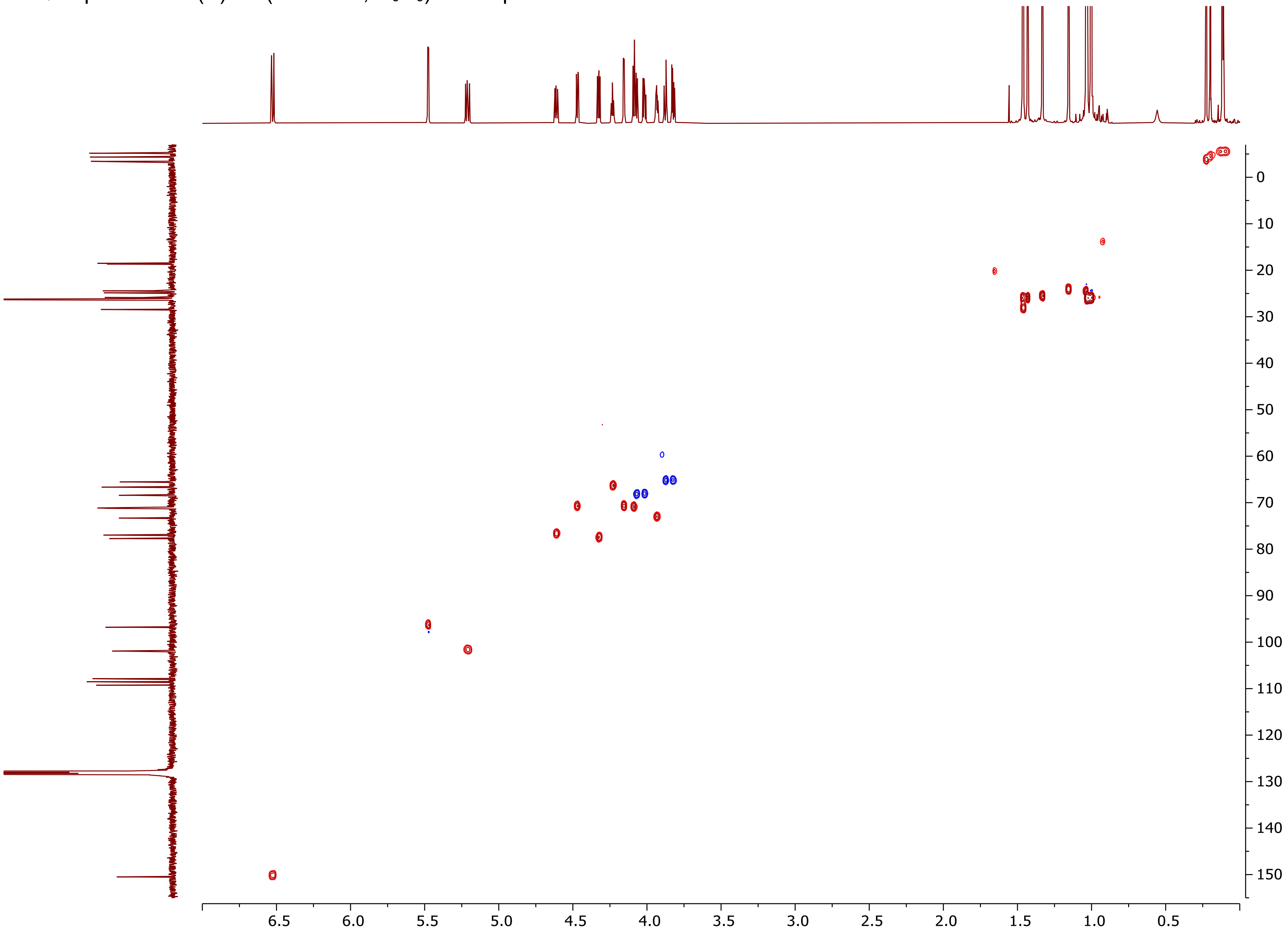

HSQC spectrum of (*E*)-**23** (800 MHz, C_6D_6) – expansion

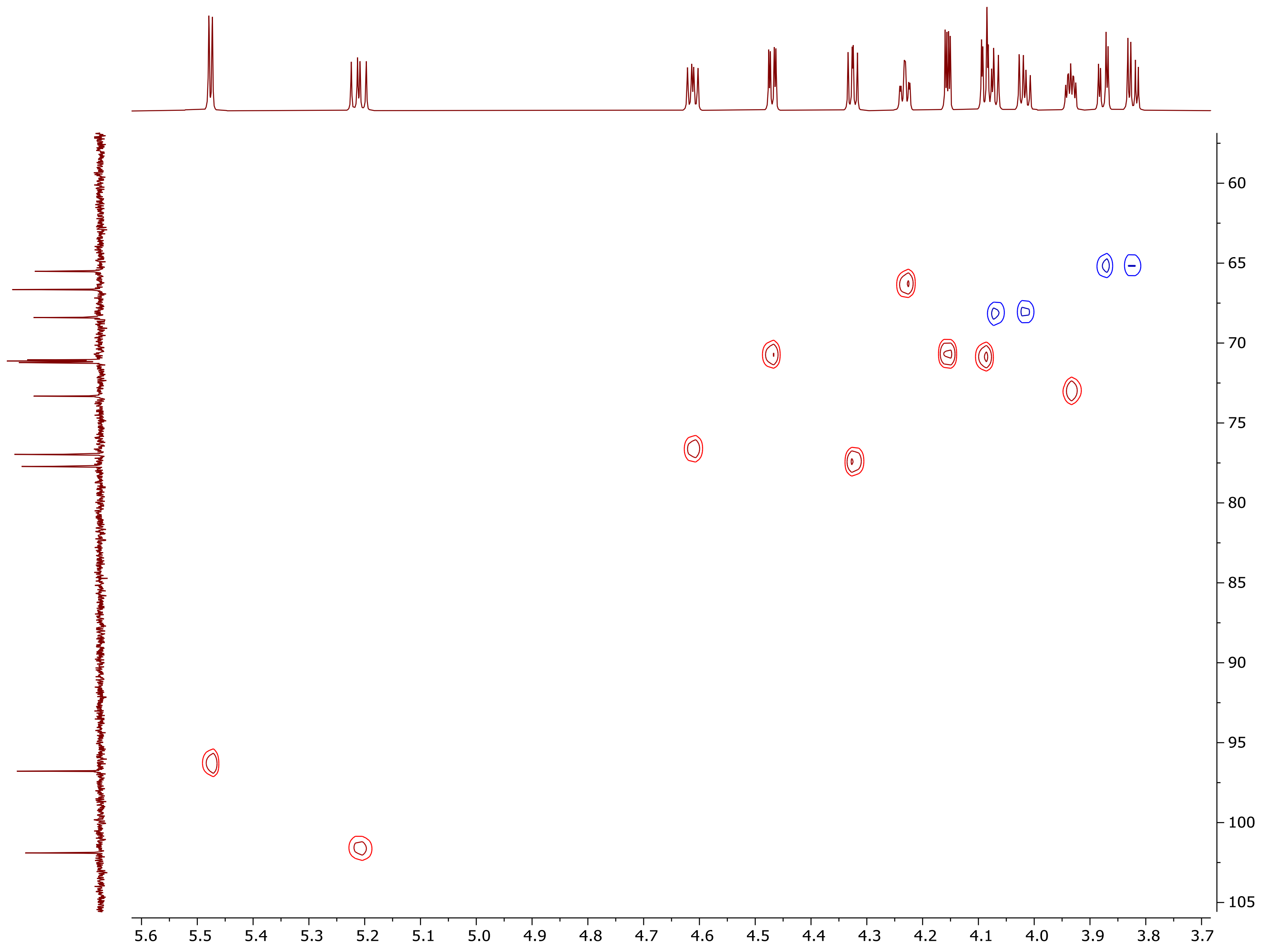

HMBC spectrum of (*E*)-**23** (800 MHz, C_6D_6) – full spectrum

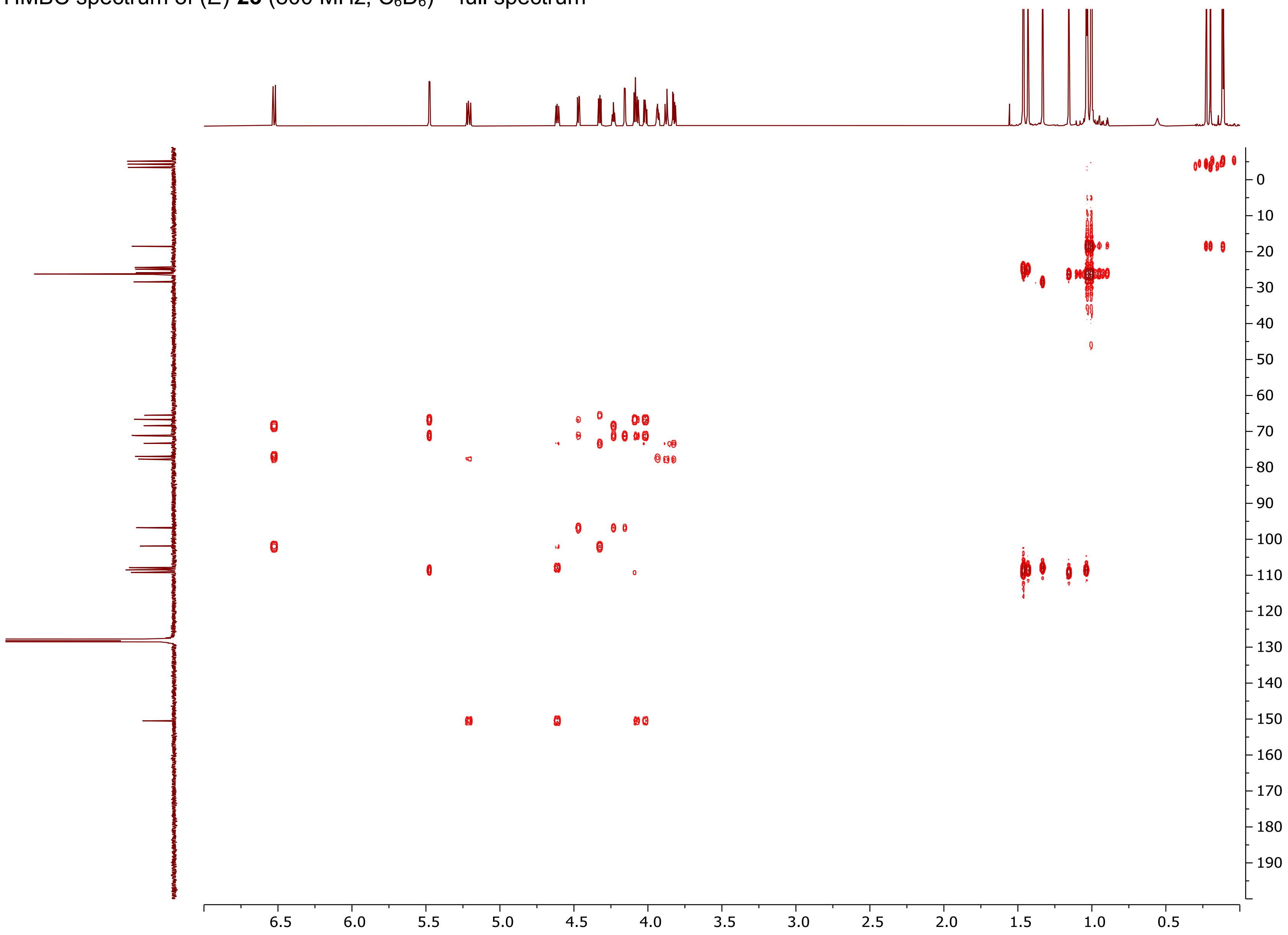

HMBC spectrum of (*E*)-**23** (800 MHz, C_6D_6) – expansion

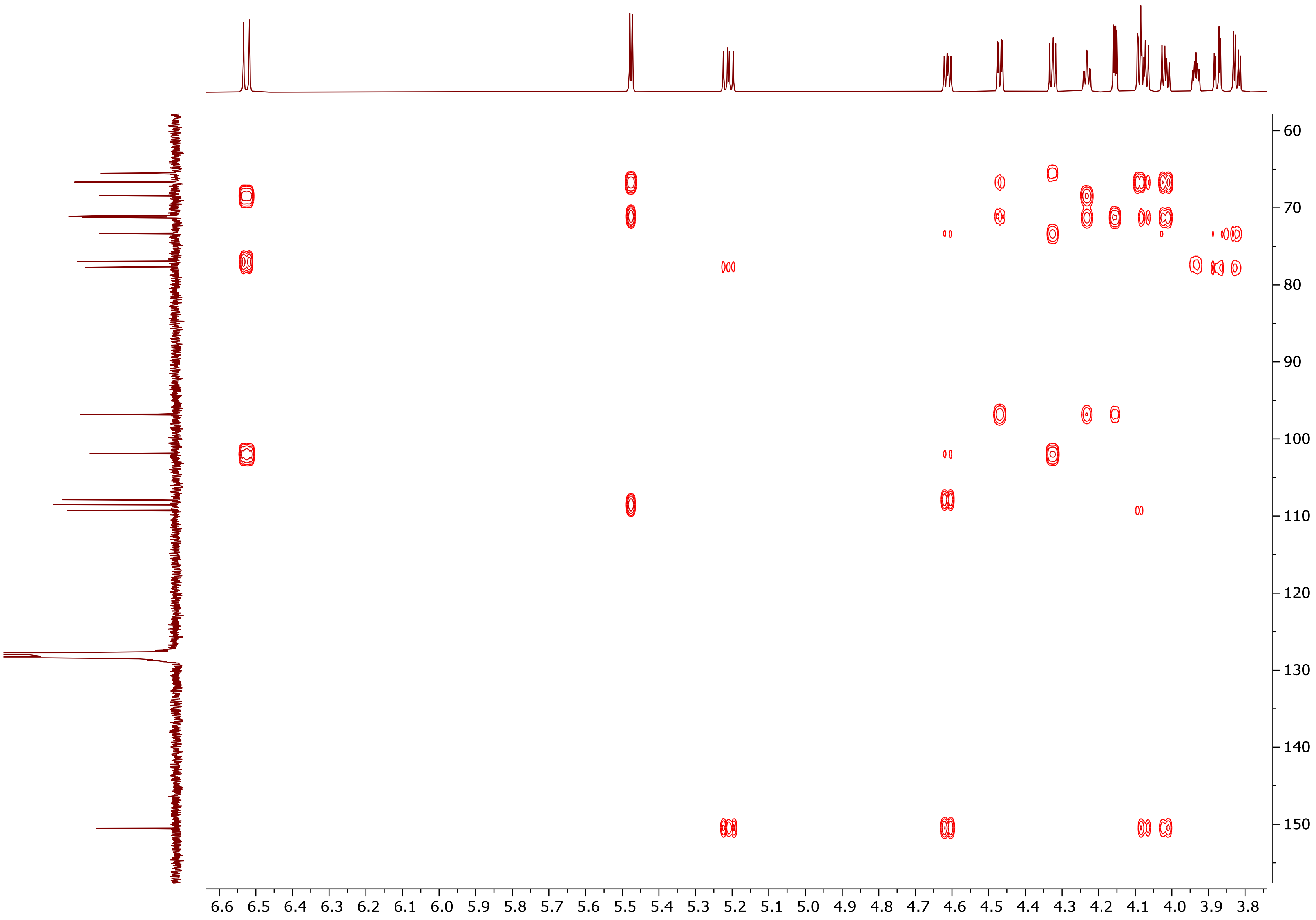

^{1}H NMR Spectrum of (*E*)**-24** (800 MHz, C_6D_6)- full spectrum

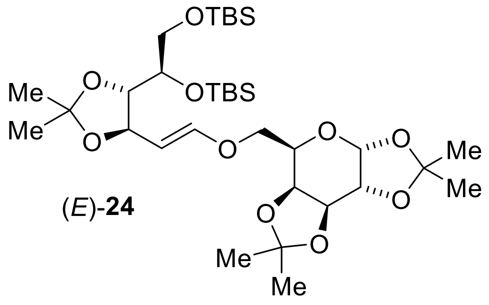

(*E*)-**24**

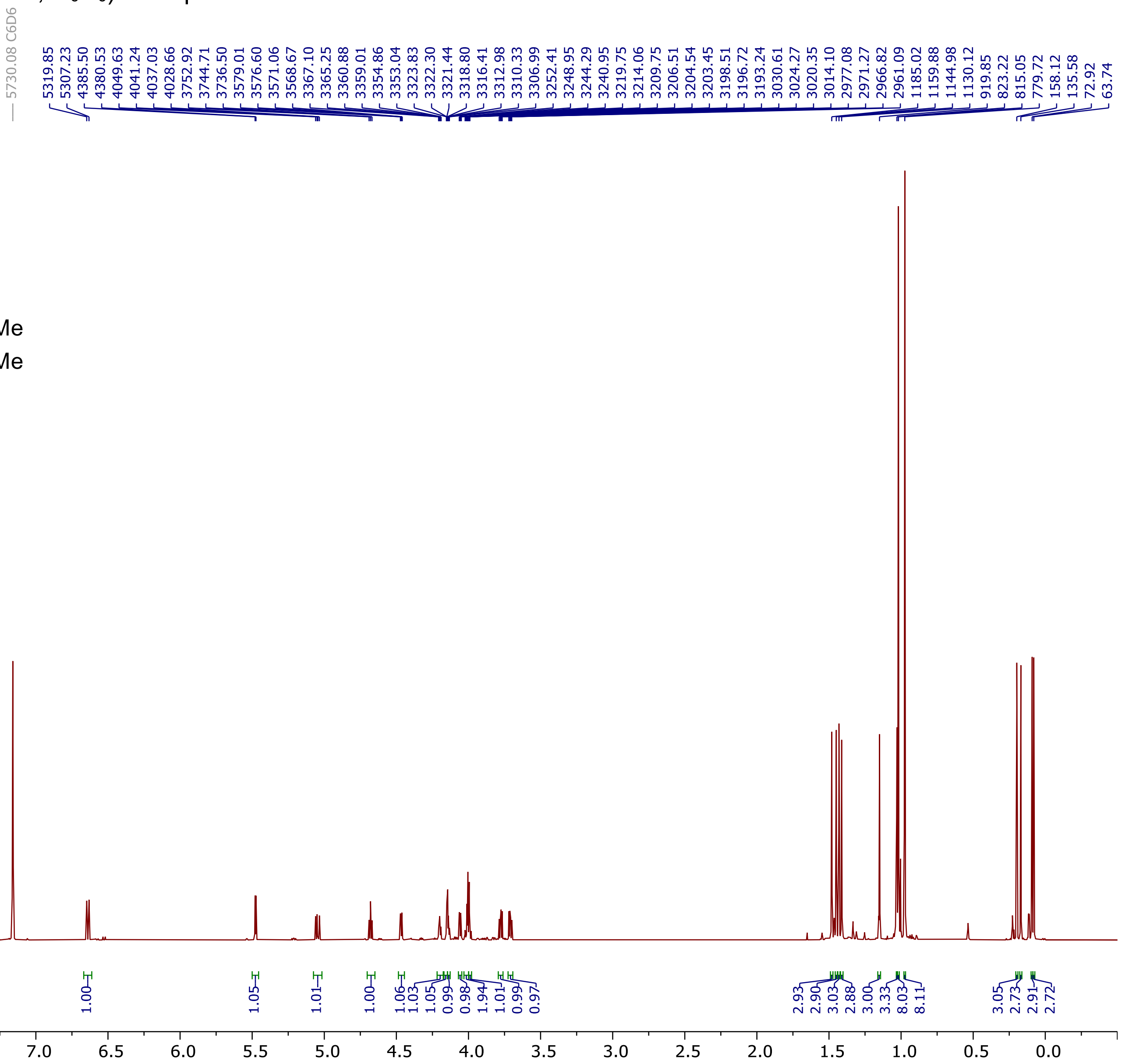

^{1}H NMR Spectrum of (*E*)**-24** (800 MHz, C_6D_6) – expansion

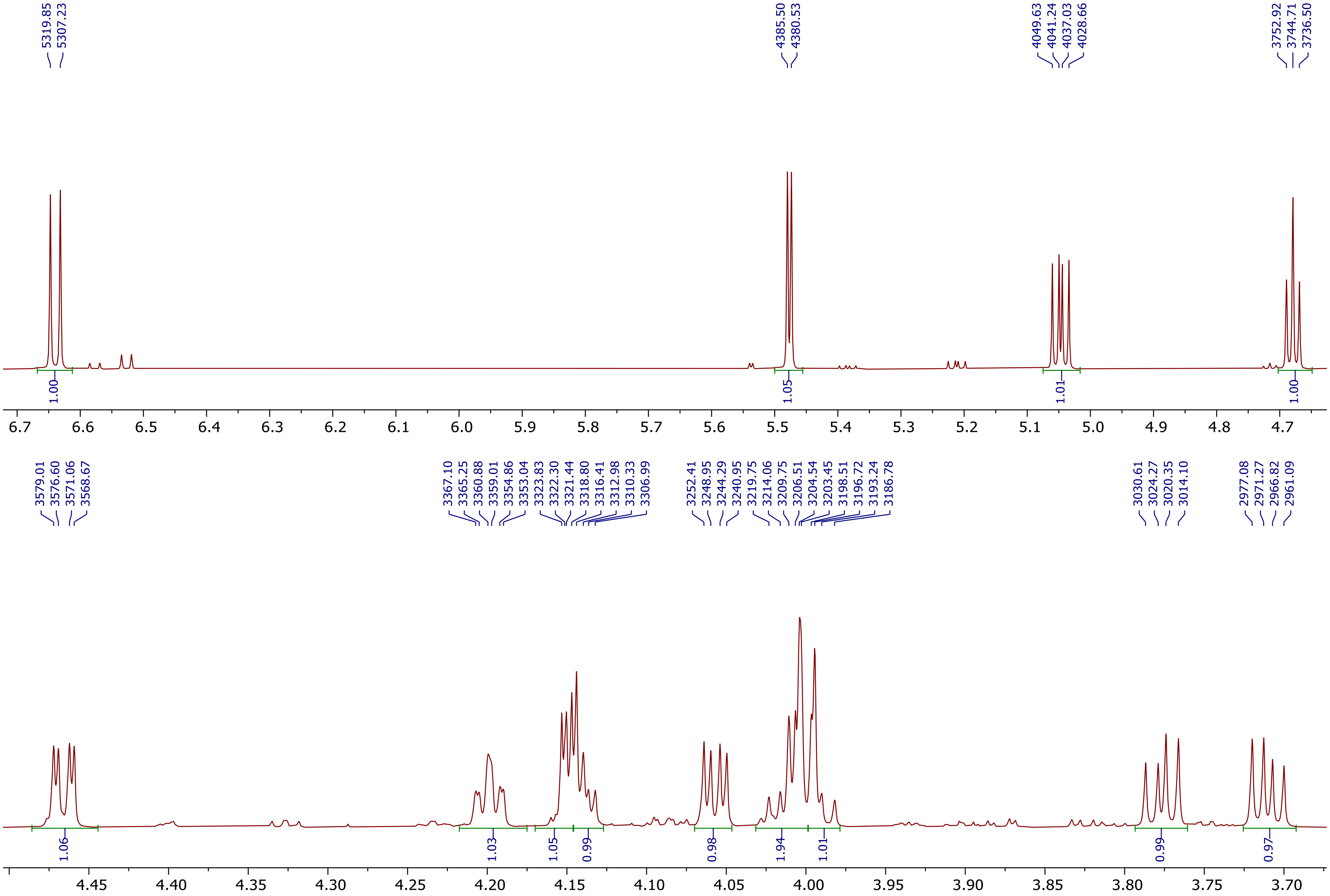

$^{13}C\{^{1}H\}$ NMR Spectrum of (*E*)**-24** (101 MHz, C_6D_6)

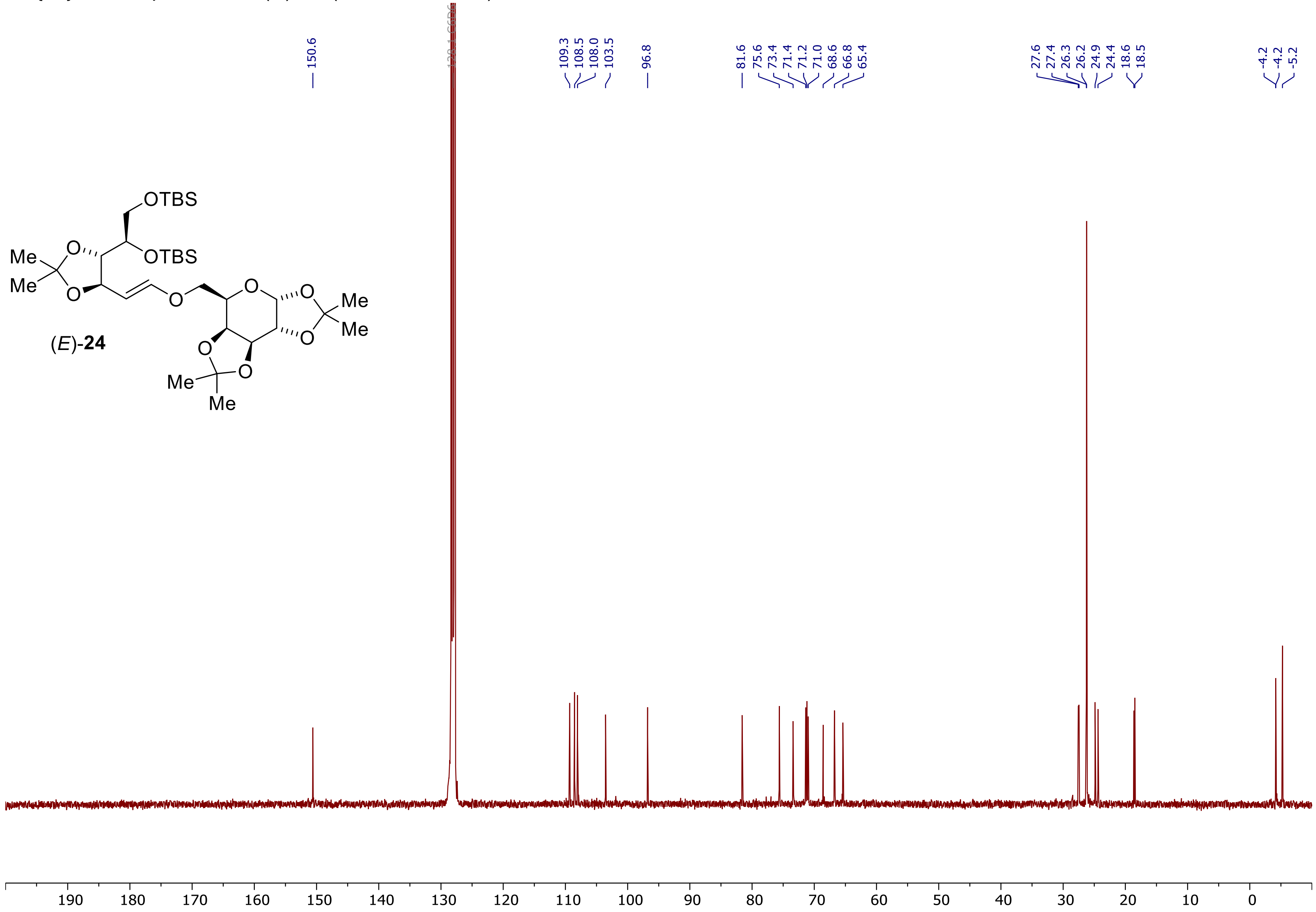

^{1}H NMR Spectrum of (*E*)**-12** (800 MHz, C_6D_6) – full spectrum

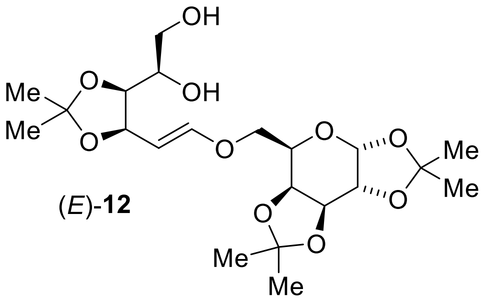

(*E*)-**12**

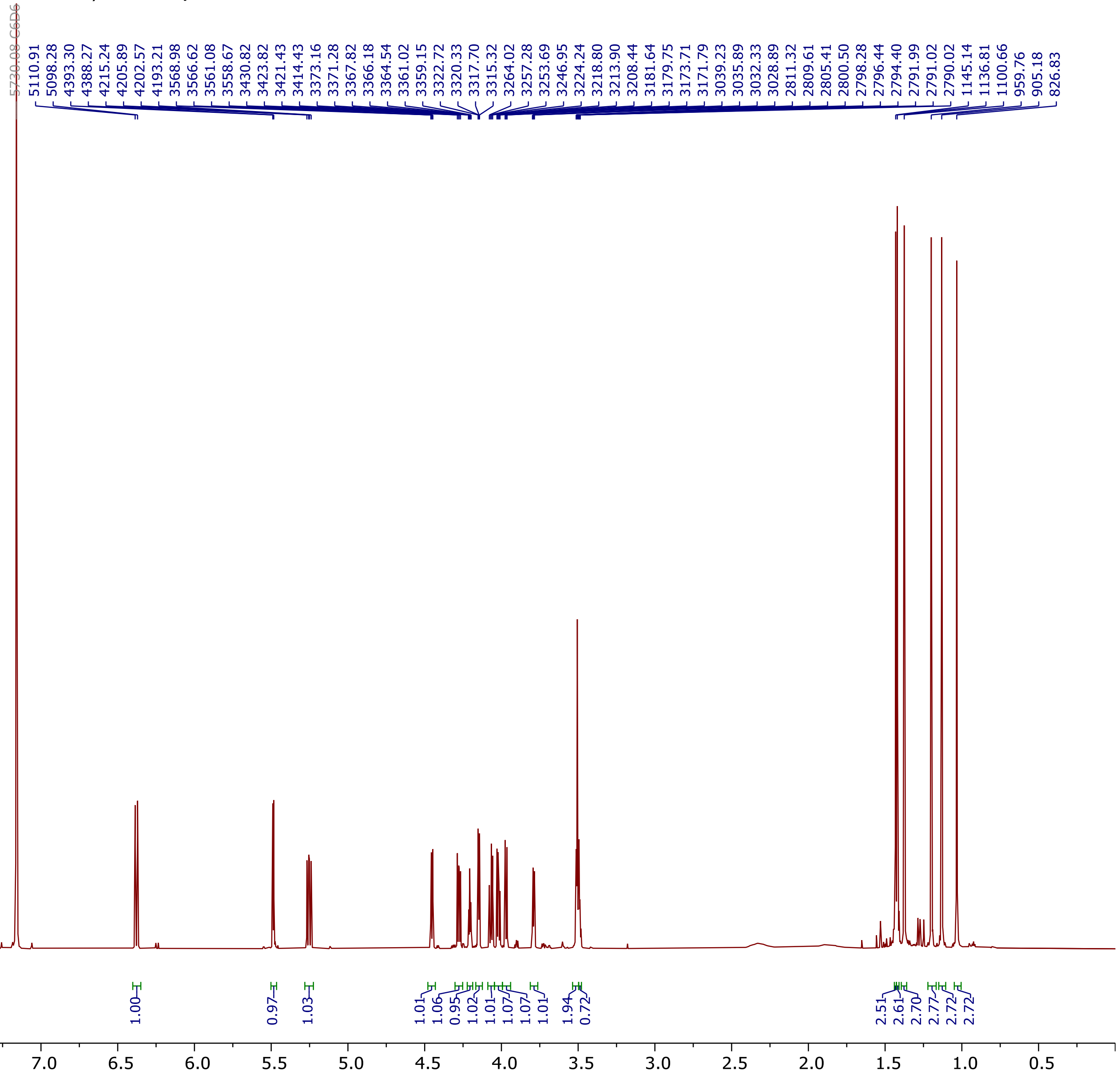

^{1}H NMR Spectrum of (*E*)-**12** (800 MHz, C_6D_6) – expansion

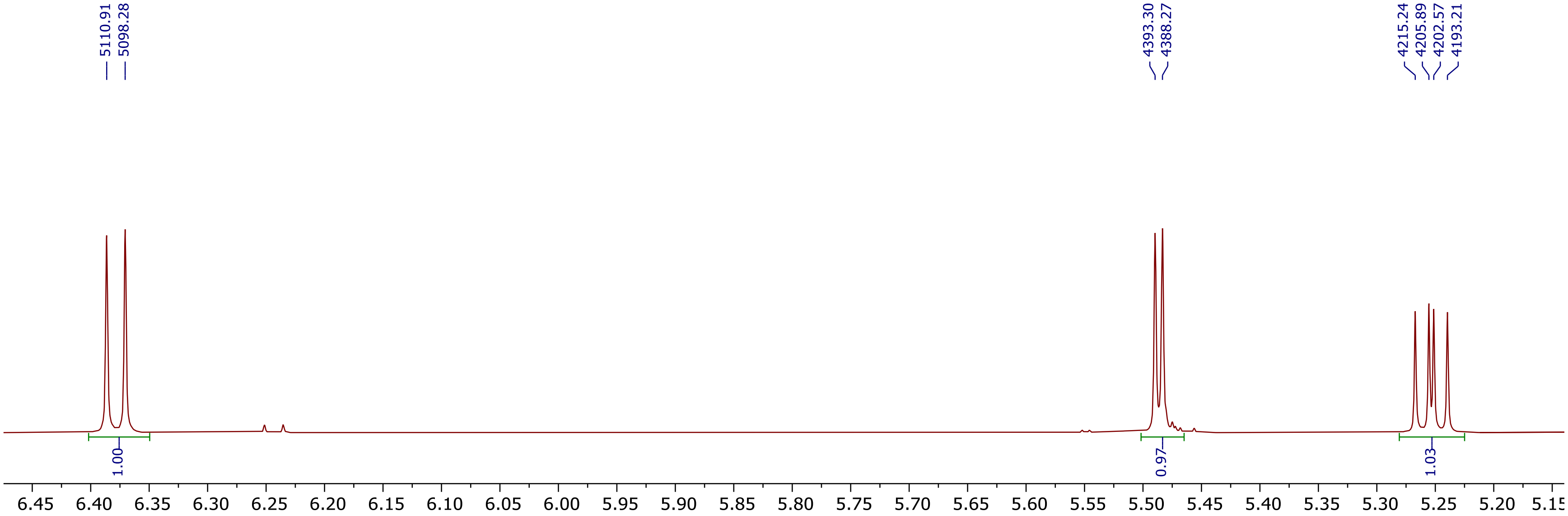

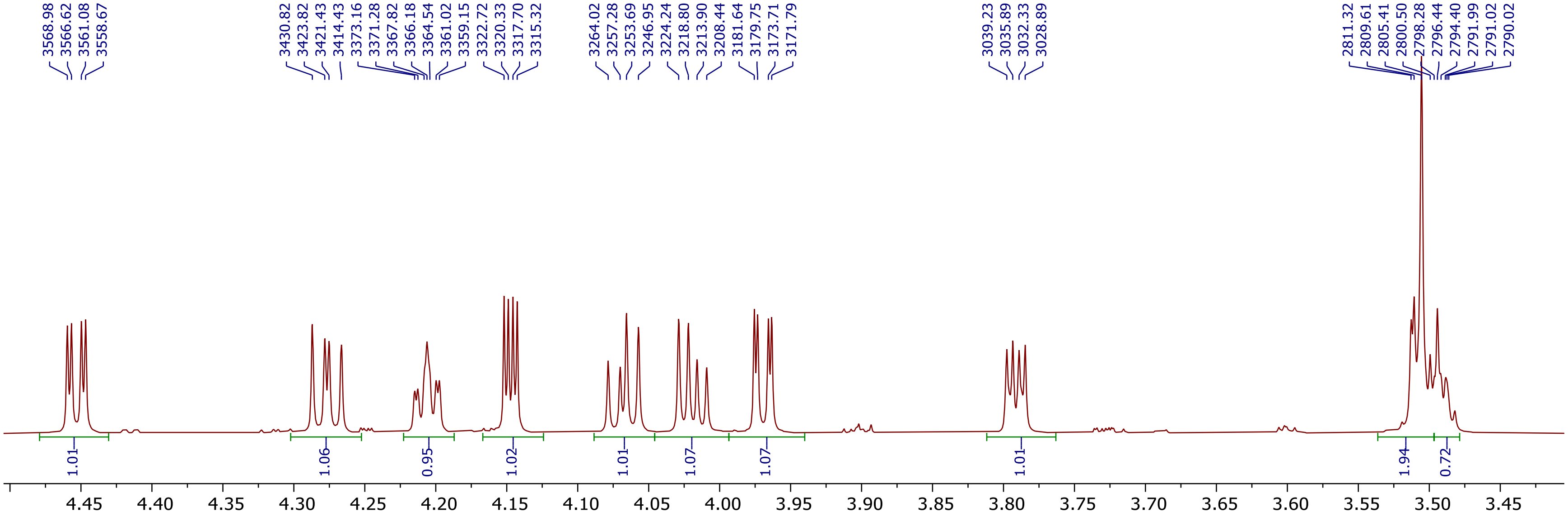

$^{13}C\{^1H\}$ NMR Spectrum of (*E*)-**12** (101 MHz, C_6D_6)

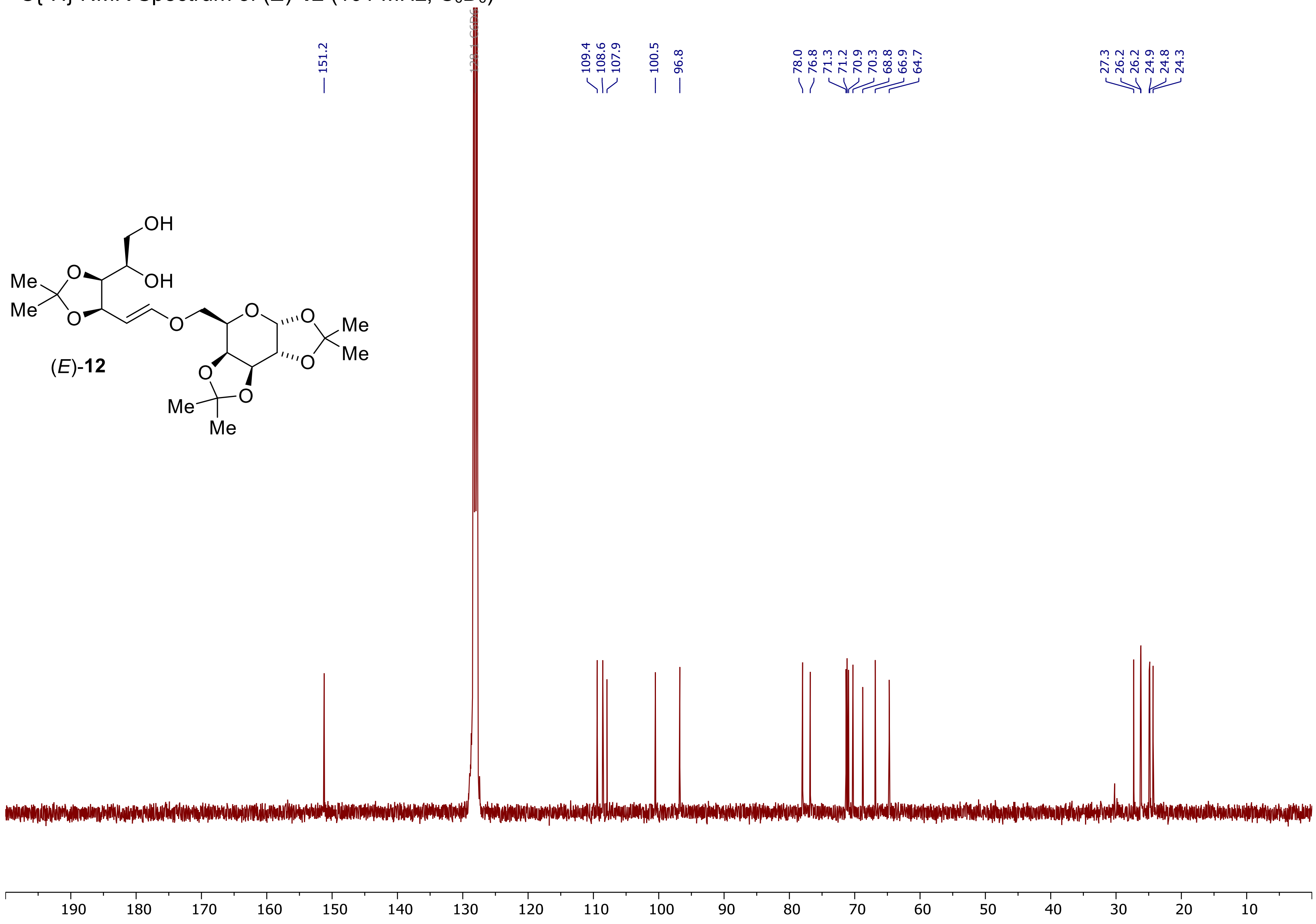

^{1}H NMR Spectrum of (*E*)-**25** (800 MHz, C_6D_6) – full spectrum

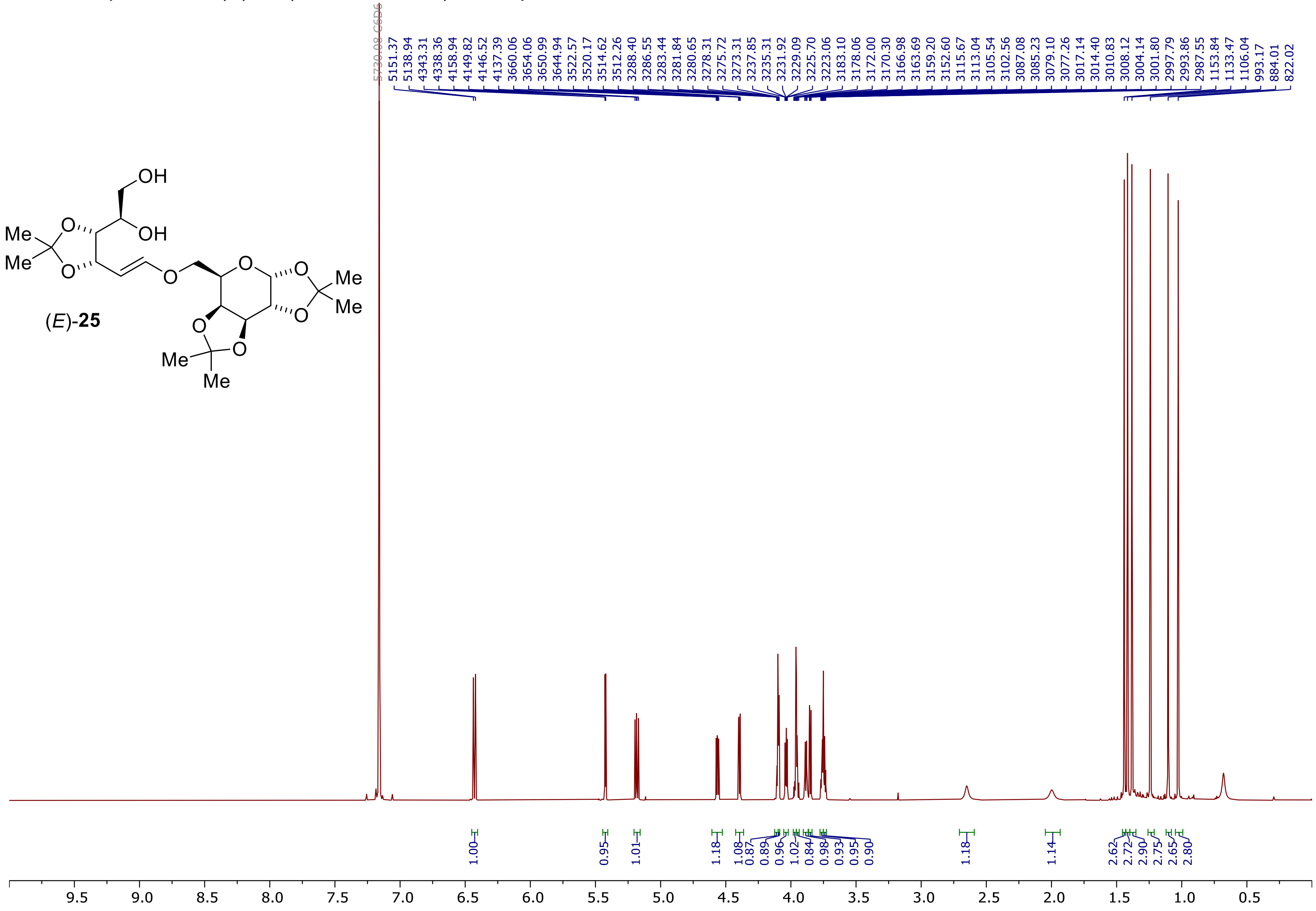

^{1}H NMR Spectrum of (*E*)-**25** (800 MHz, C_6D_6) – expansion

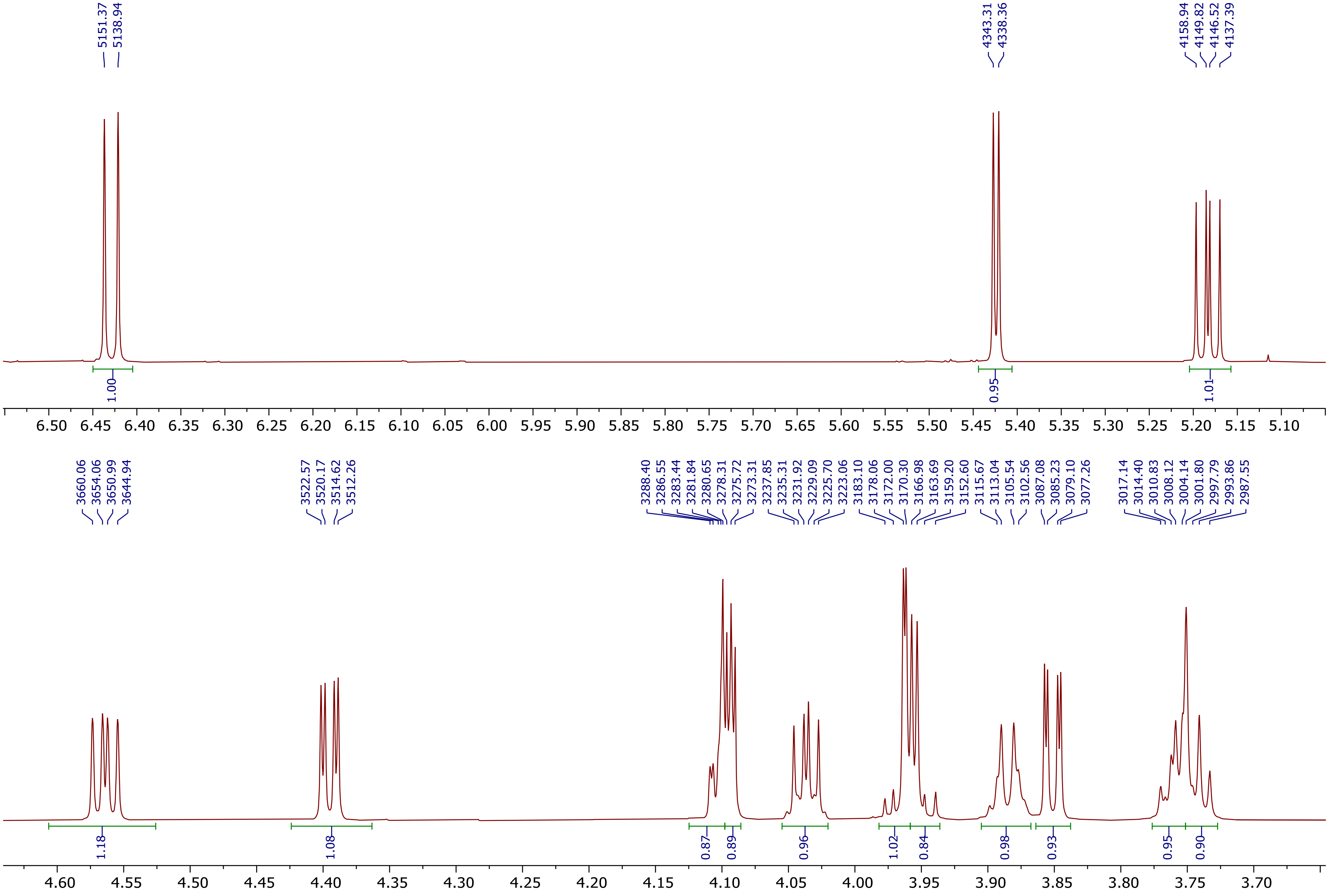

$^{13}C\{^{1}H\}$ NMR Spectrum of (*E*)-**25** (101 MHz, C_6D_6)

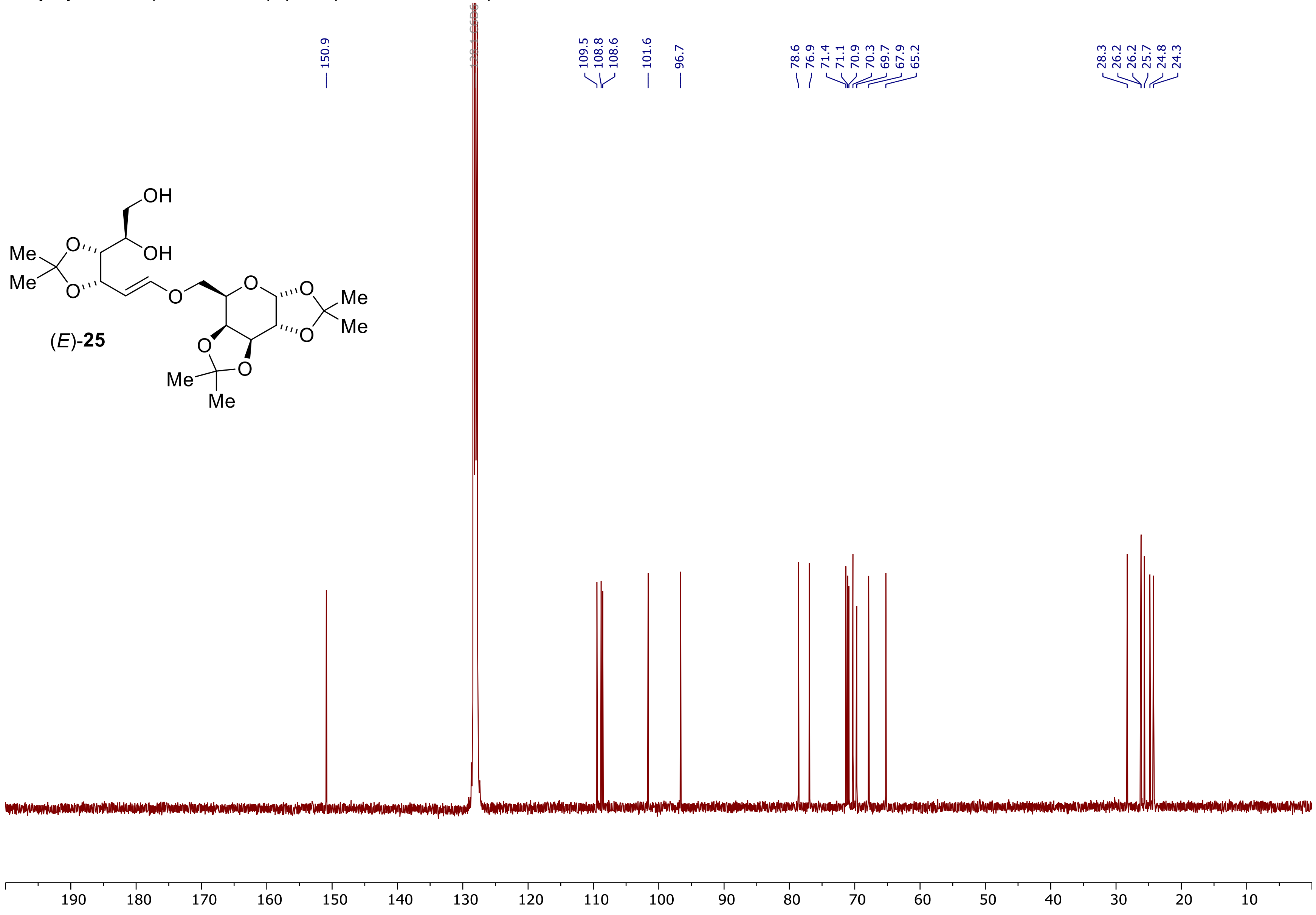

(*E*)-**25**

^{1}H NMR Spectrum of **26** (400 MHz, C_6D_6)

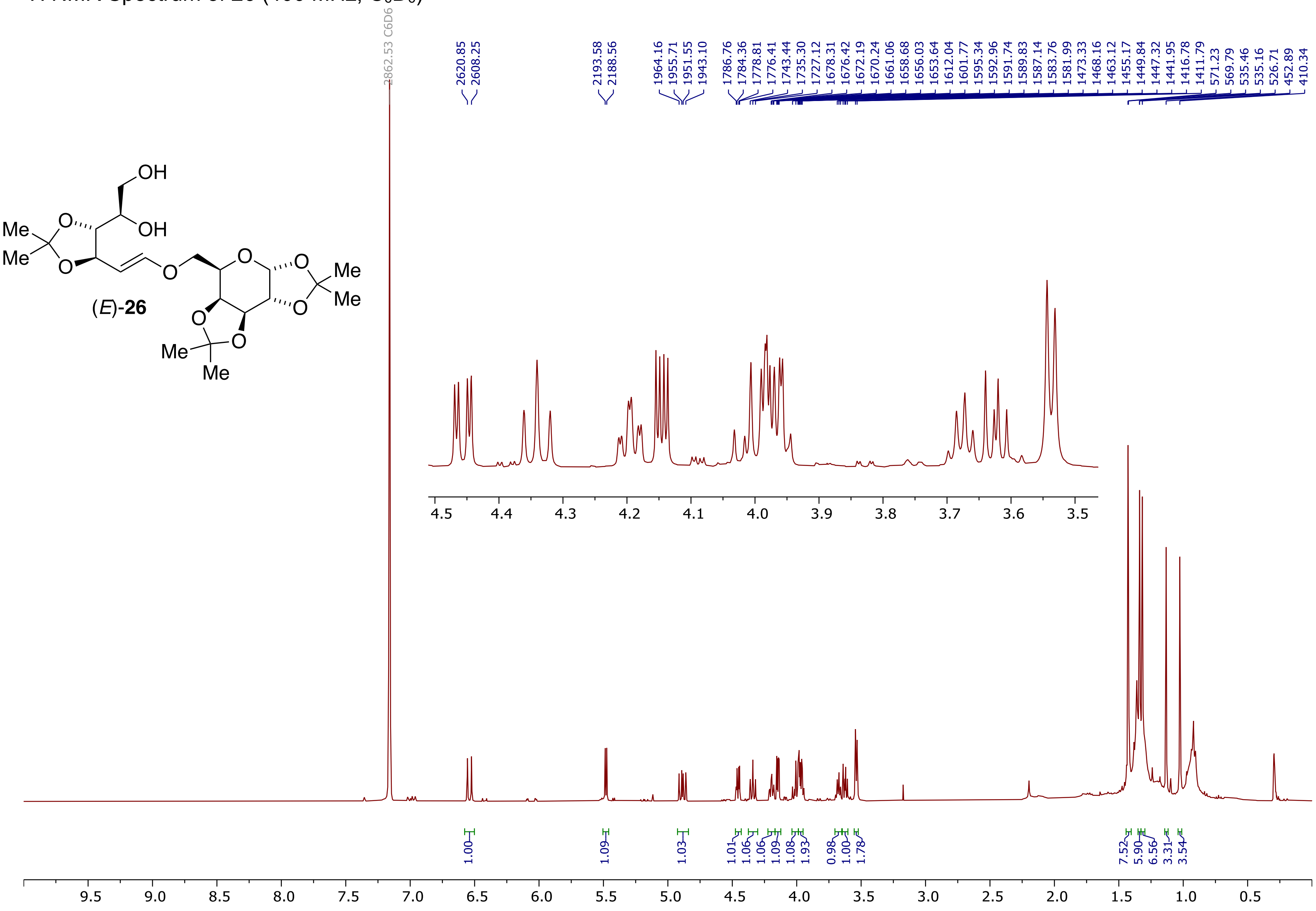

$^{13}C\{^{1}H\}$ NMR Spectrum of **26** (101 MHz, C_6D_6)

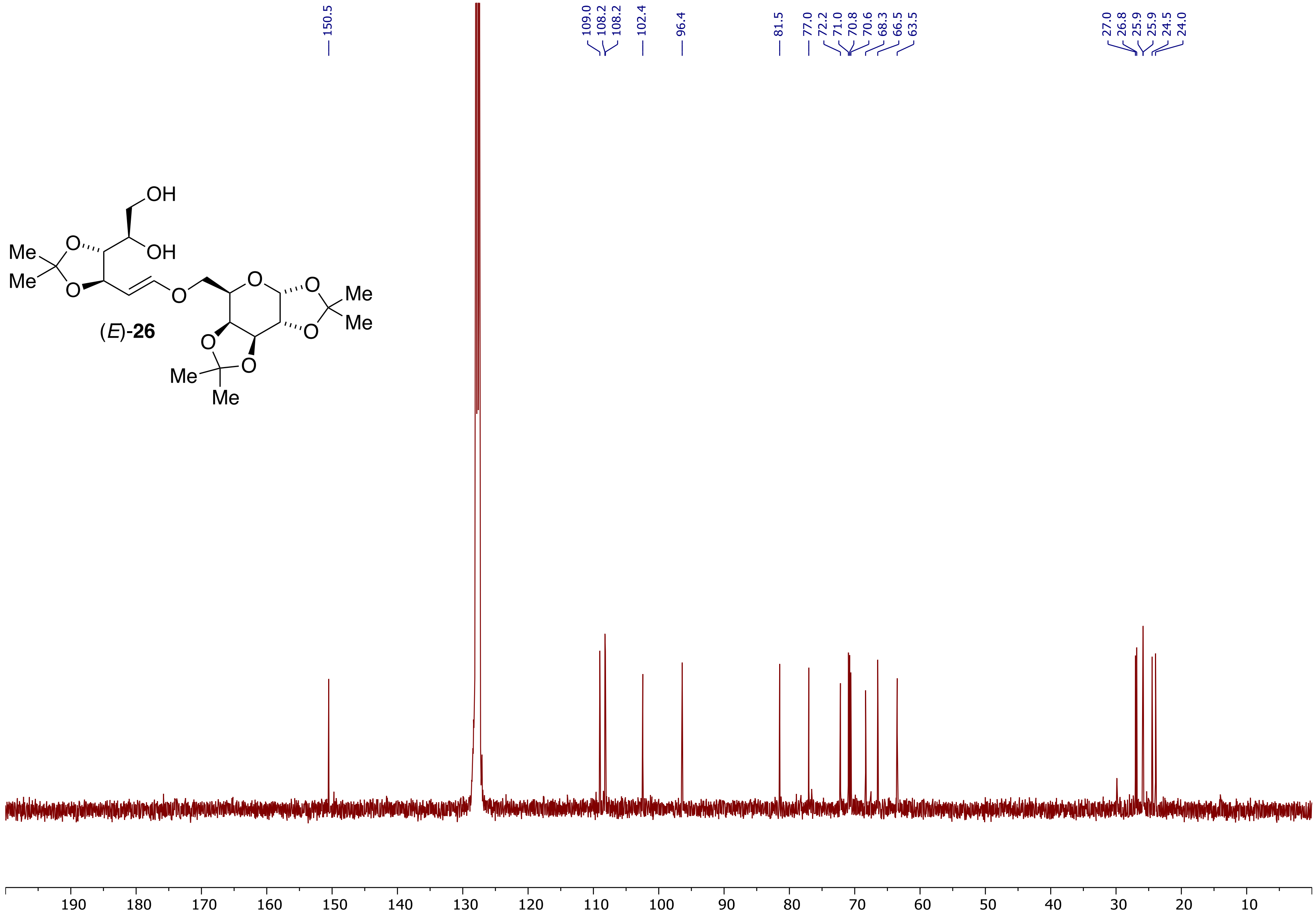

^{1}H NMR Spectrum of **15** from (*E*)-**12** (800 MHz, C_6D_6) – full spectrum

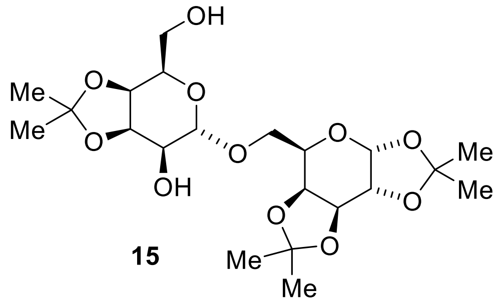

15

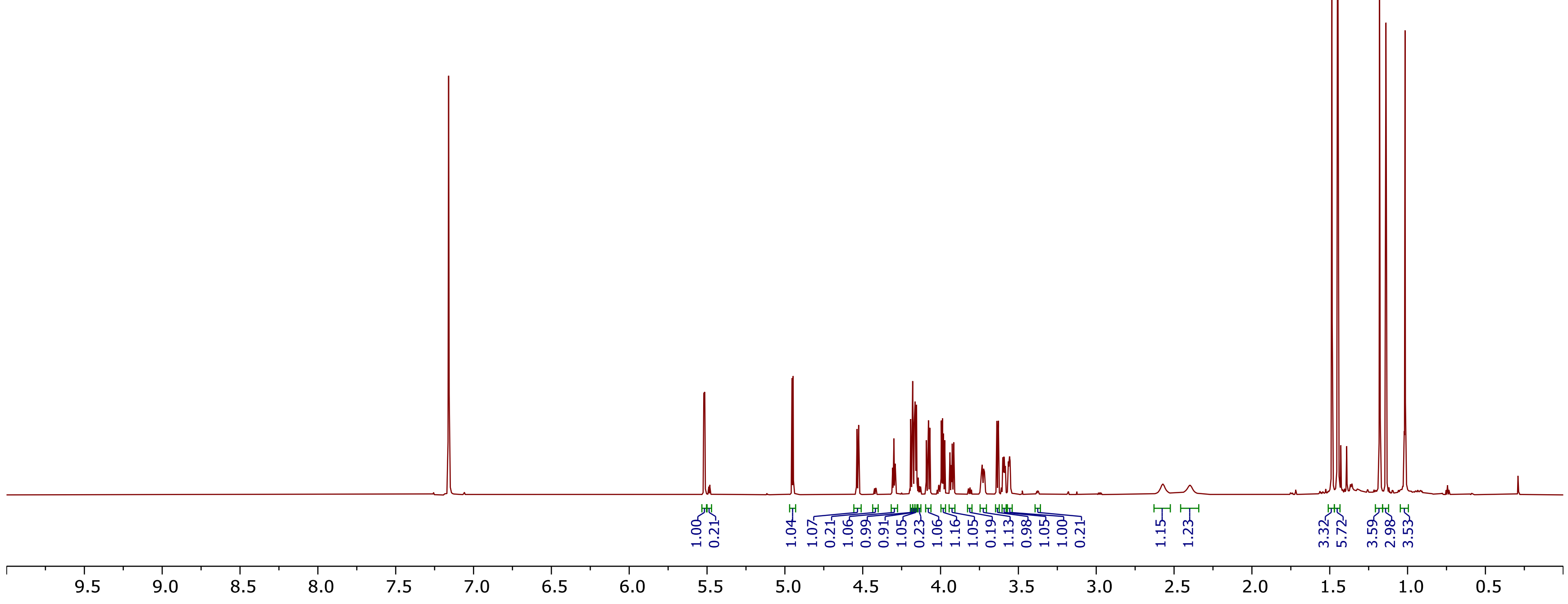

^{1}H NMR Spectrum of **15** from (*E*)-**12** (800 MHz, C_6D_6) – expansion

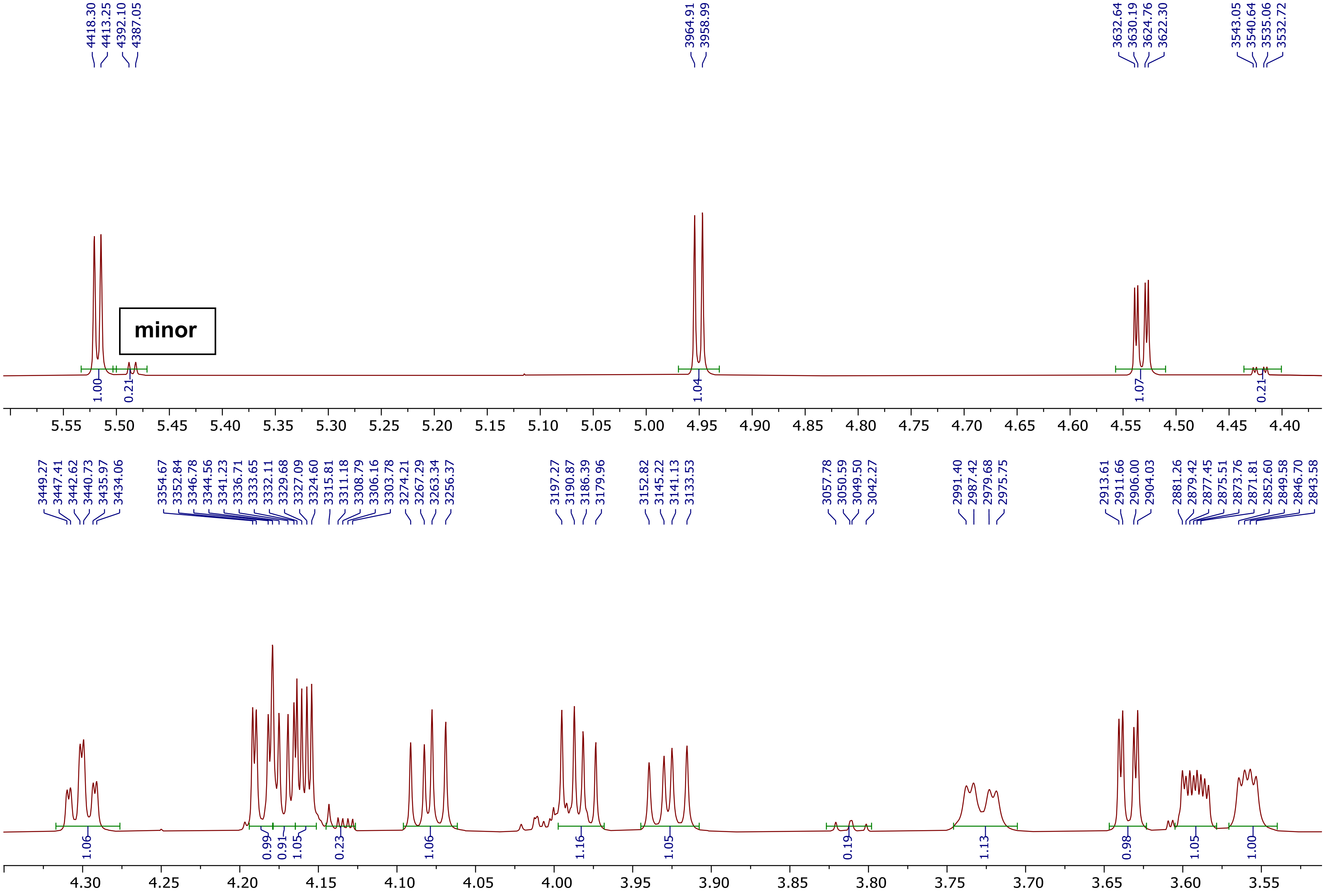

$^{13}C\{^{1}H\}$ NMR Spectrum of **15** from (*E*)-**12** (101 MHz, C_6D_6)

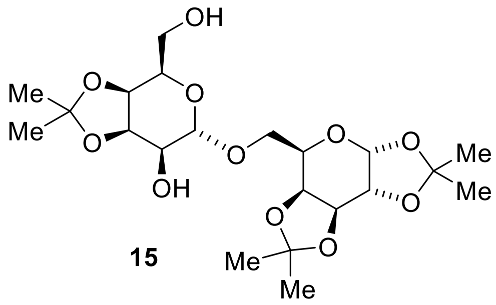

15

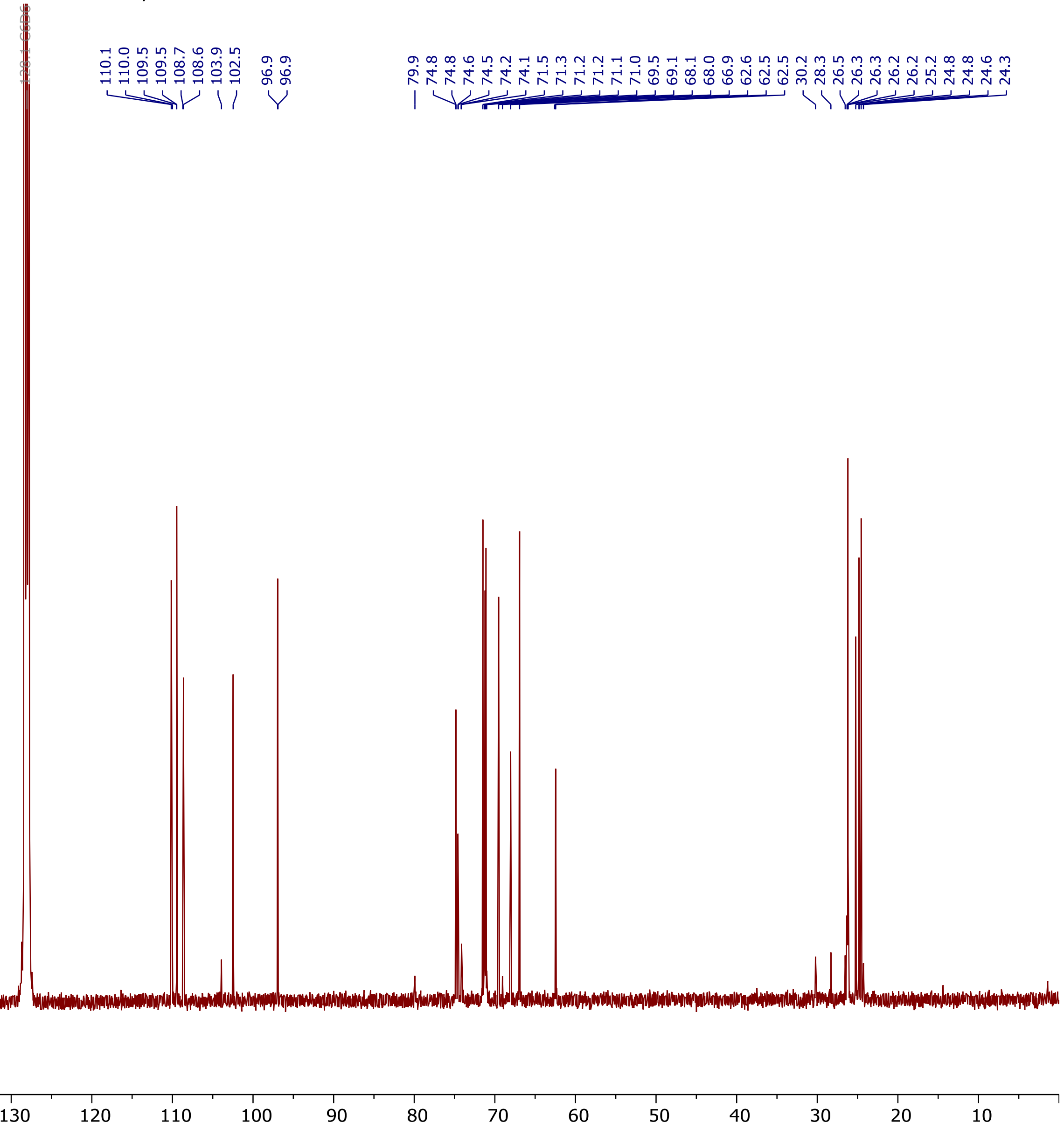

COSY spectrum of **15** from (*E*)-**12** (800 MHz, C_6D_6) – full spectrum

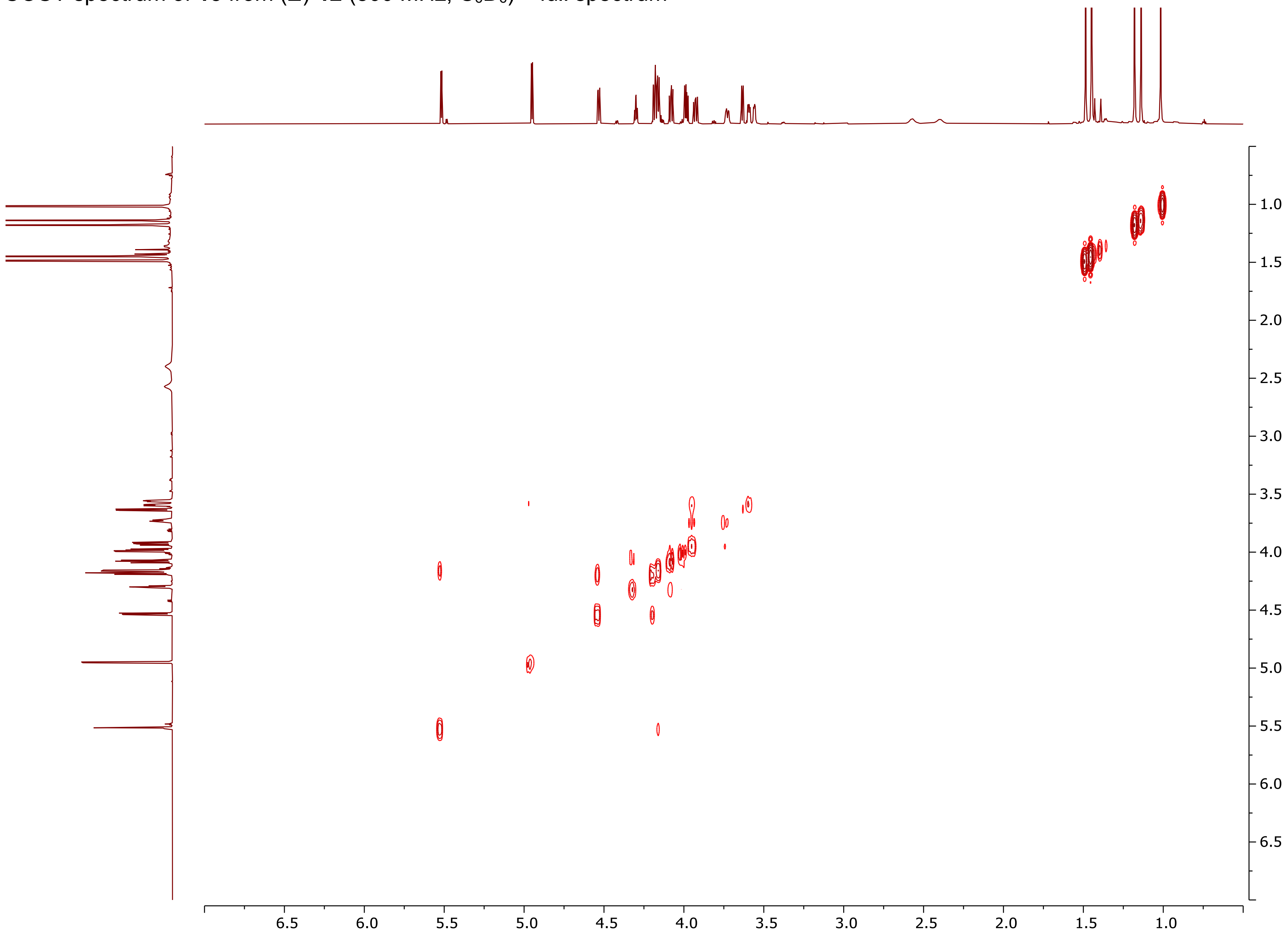

COSY spectrum of **15** from (*E*)-**12** (800 MHz, C_6D_6) – expansion

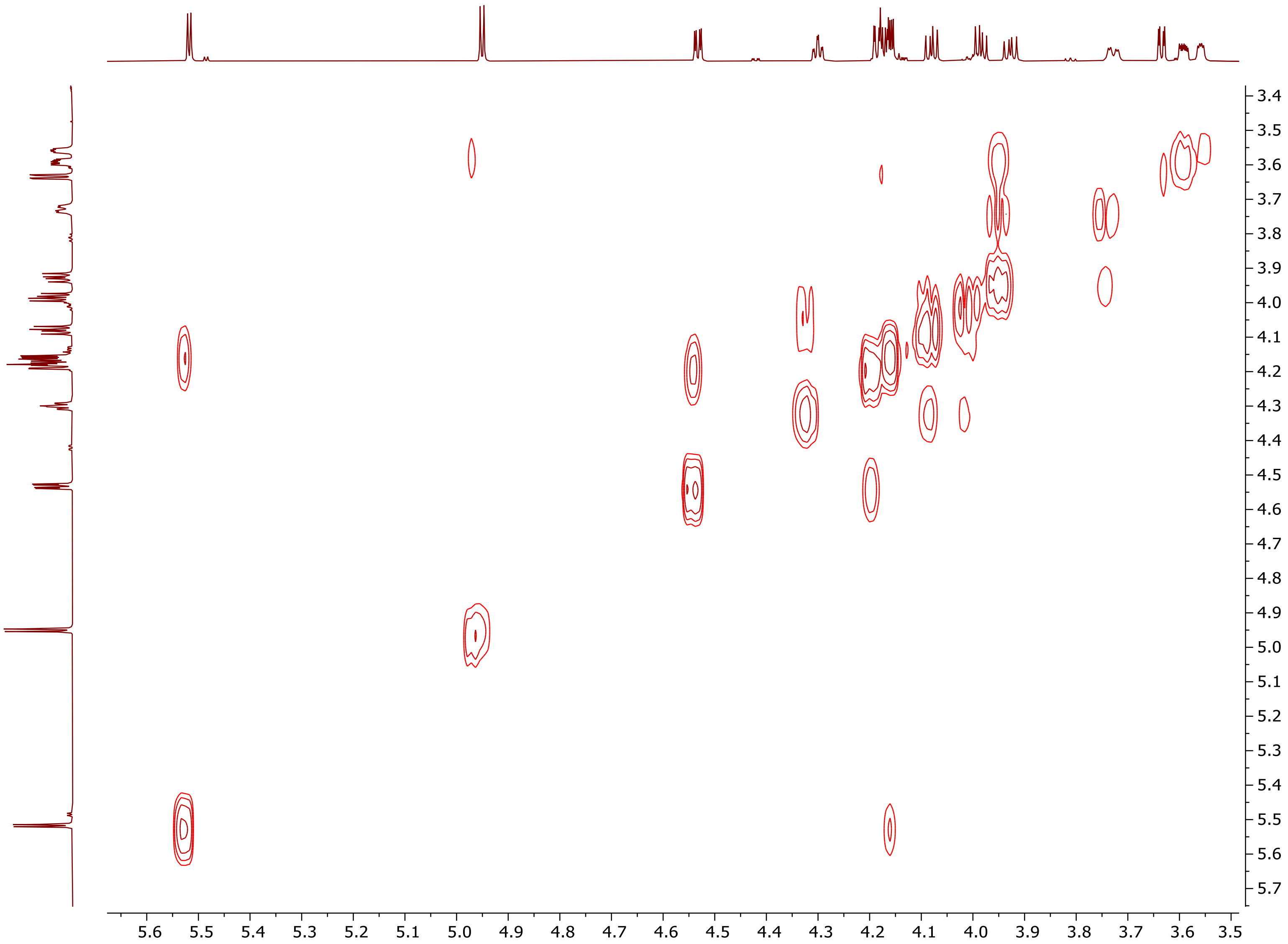

HSQC spectrum of **15** from (*E*)-**12** (800 MHz, C_6D_6) – full spectrum

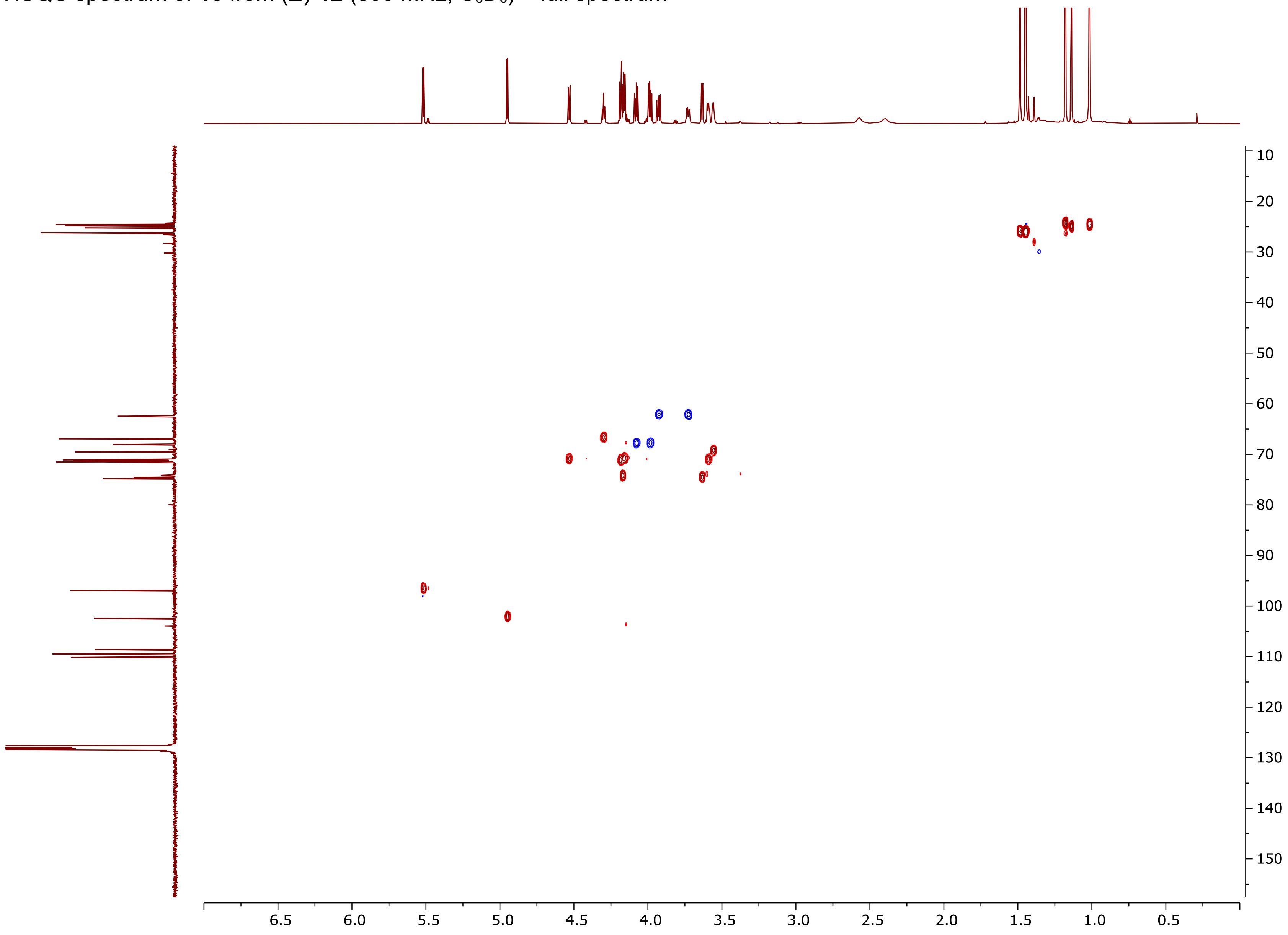

HSQC spectrum of **15** from (*E*)-**12** (800 MHz, C_6D_6) – expansion

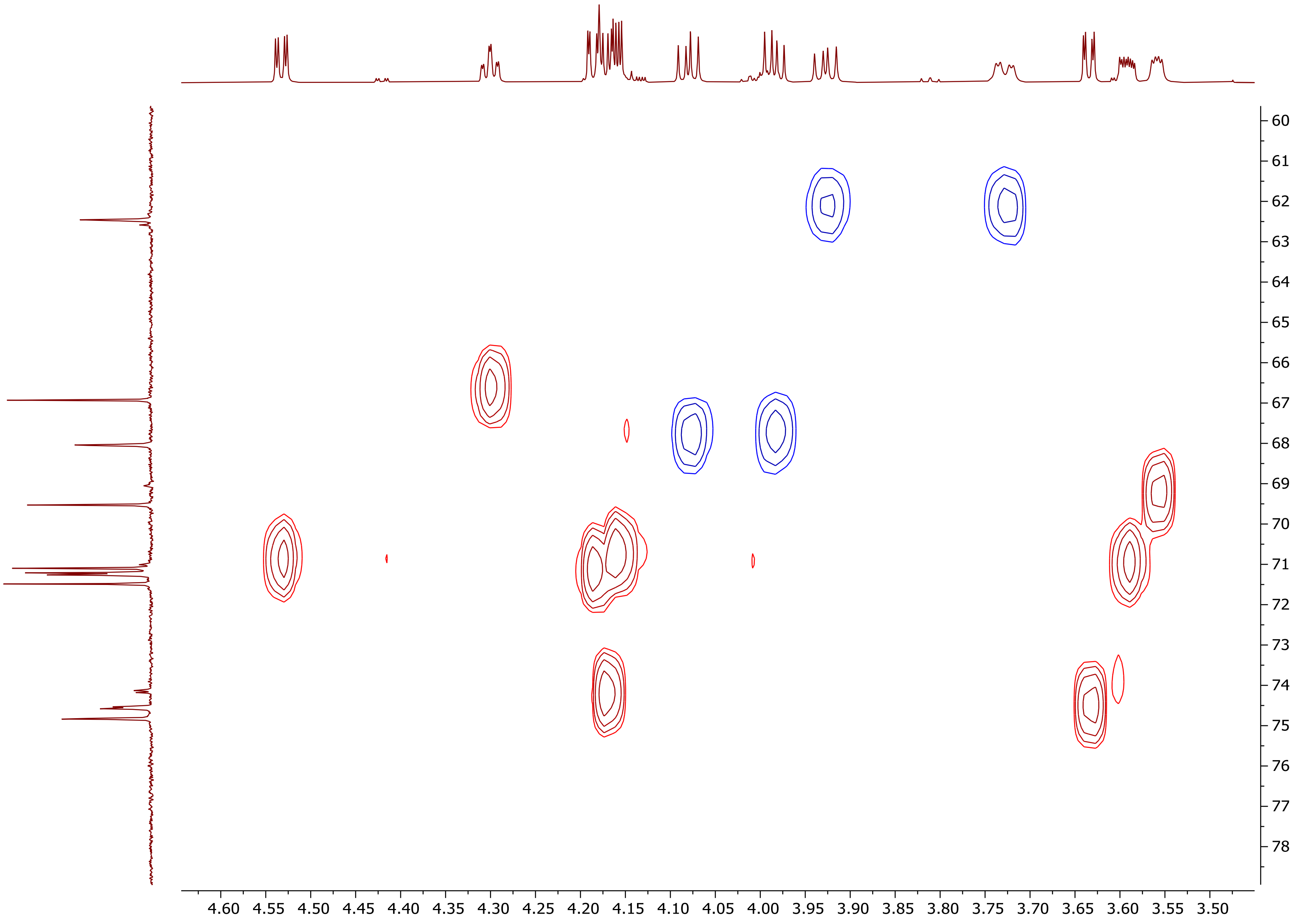

HMBC spectrum of **15** from (*E*)-**12** (800 MHz, C_6D_6) – full spectrum

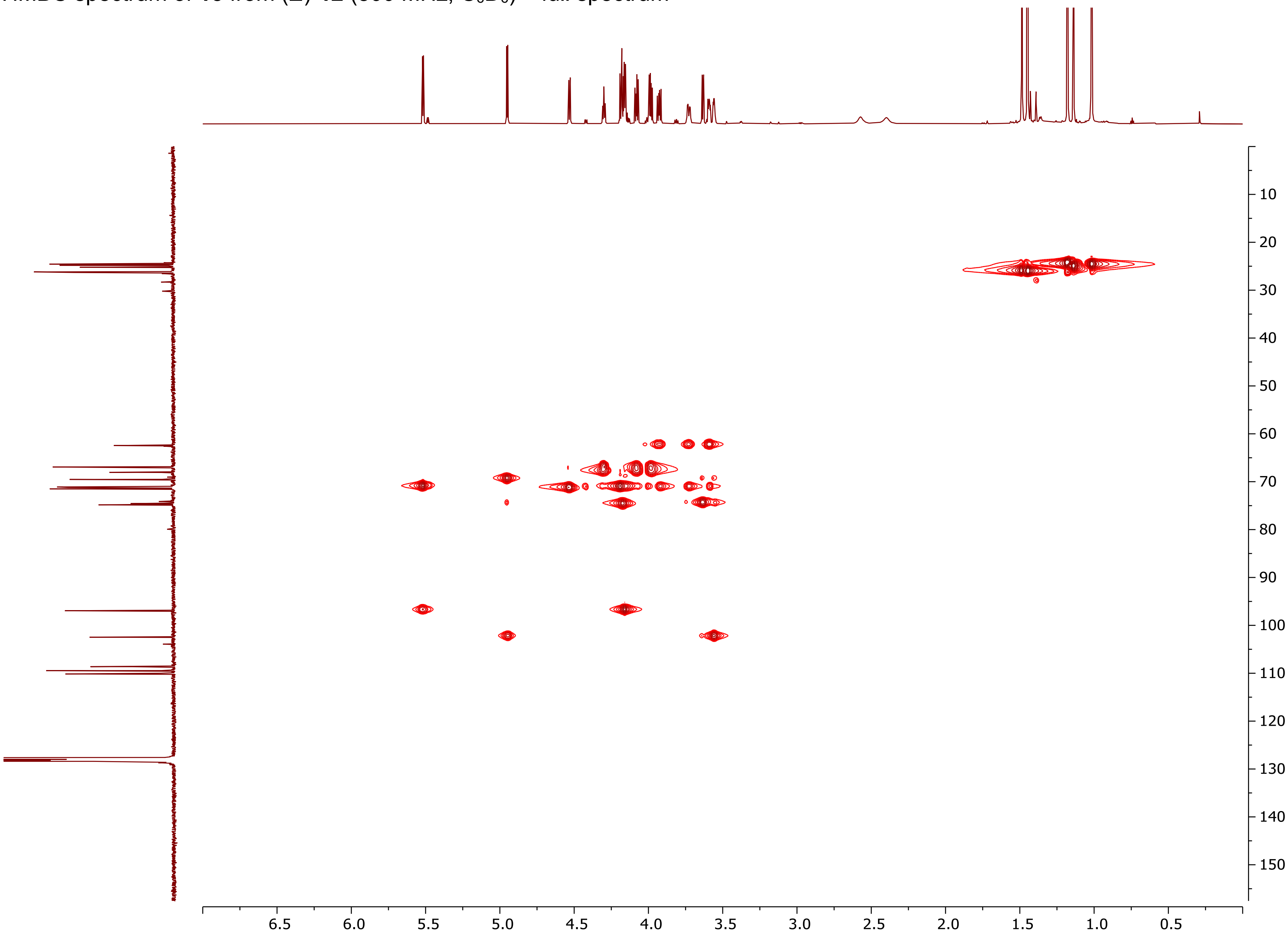

HMBC spectrum of **15** from (*E*)-**12** (800 MHz, C_6D_6) – expansion

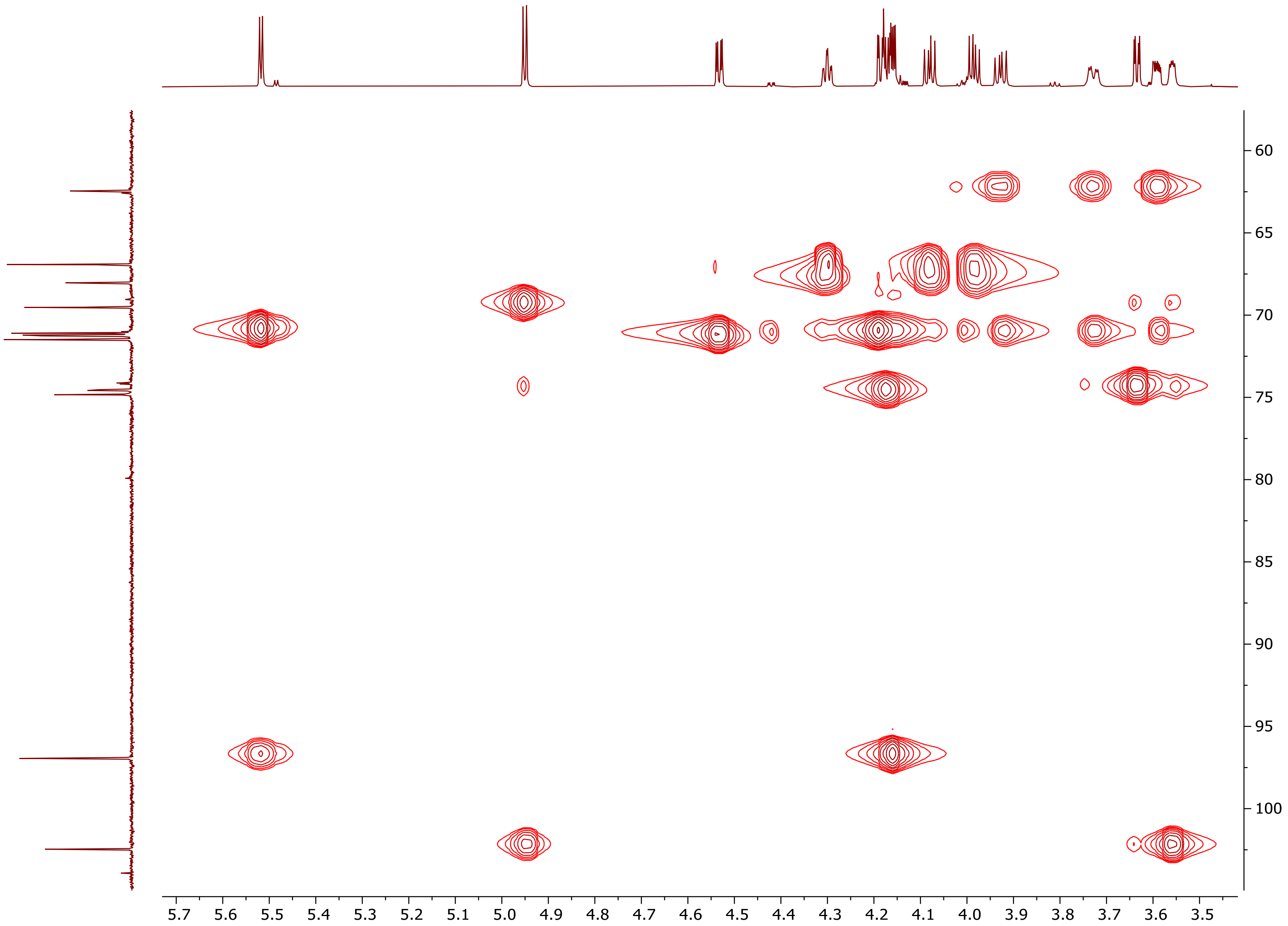

^{1}H NMR Spectrum of **27** (800 MHz, C_6D_6) – full spectrum

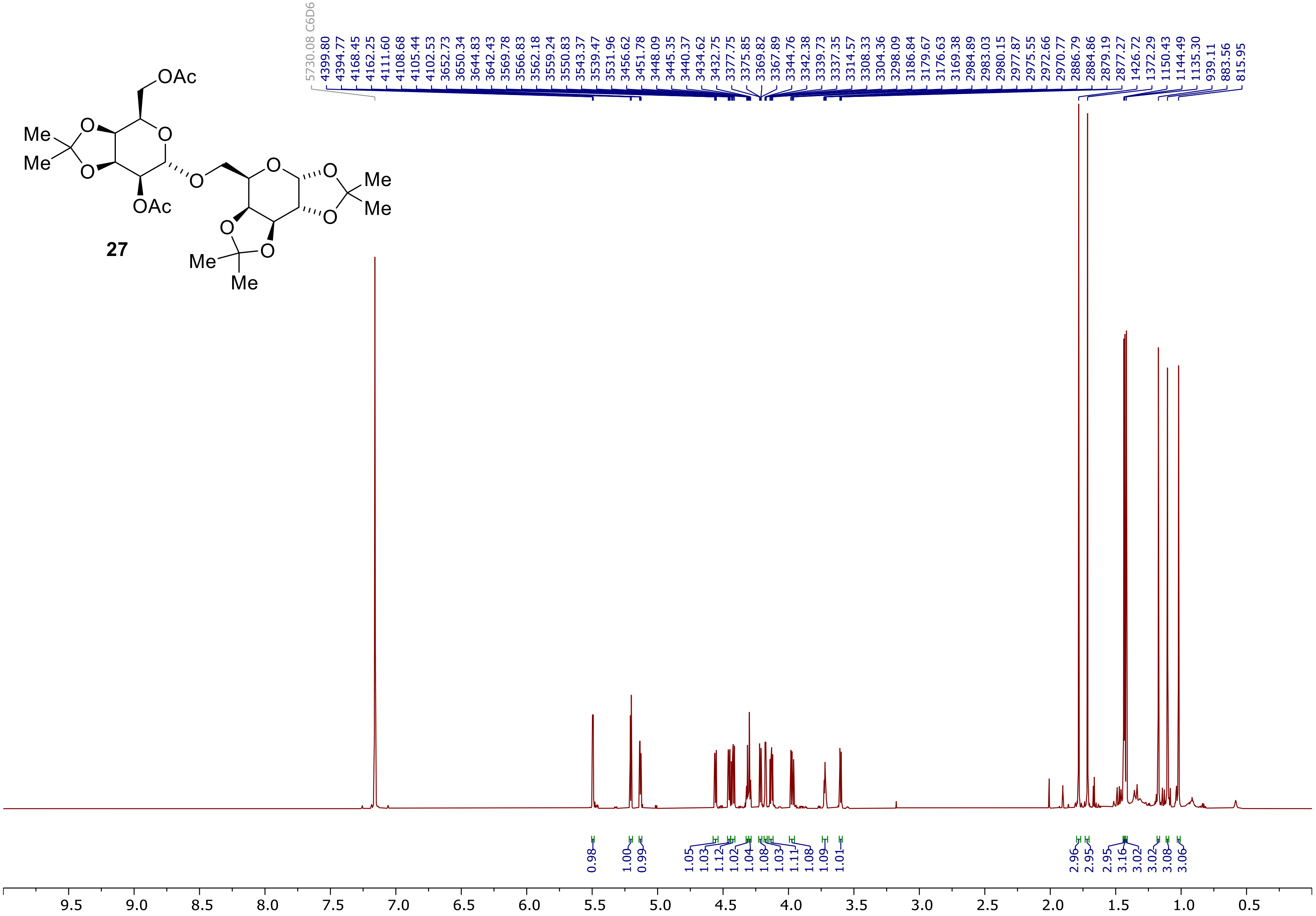

^{1}H NMR Spectrum of **27** (800 MHz, C_6D_6) – expansion

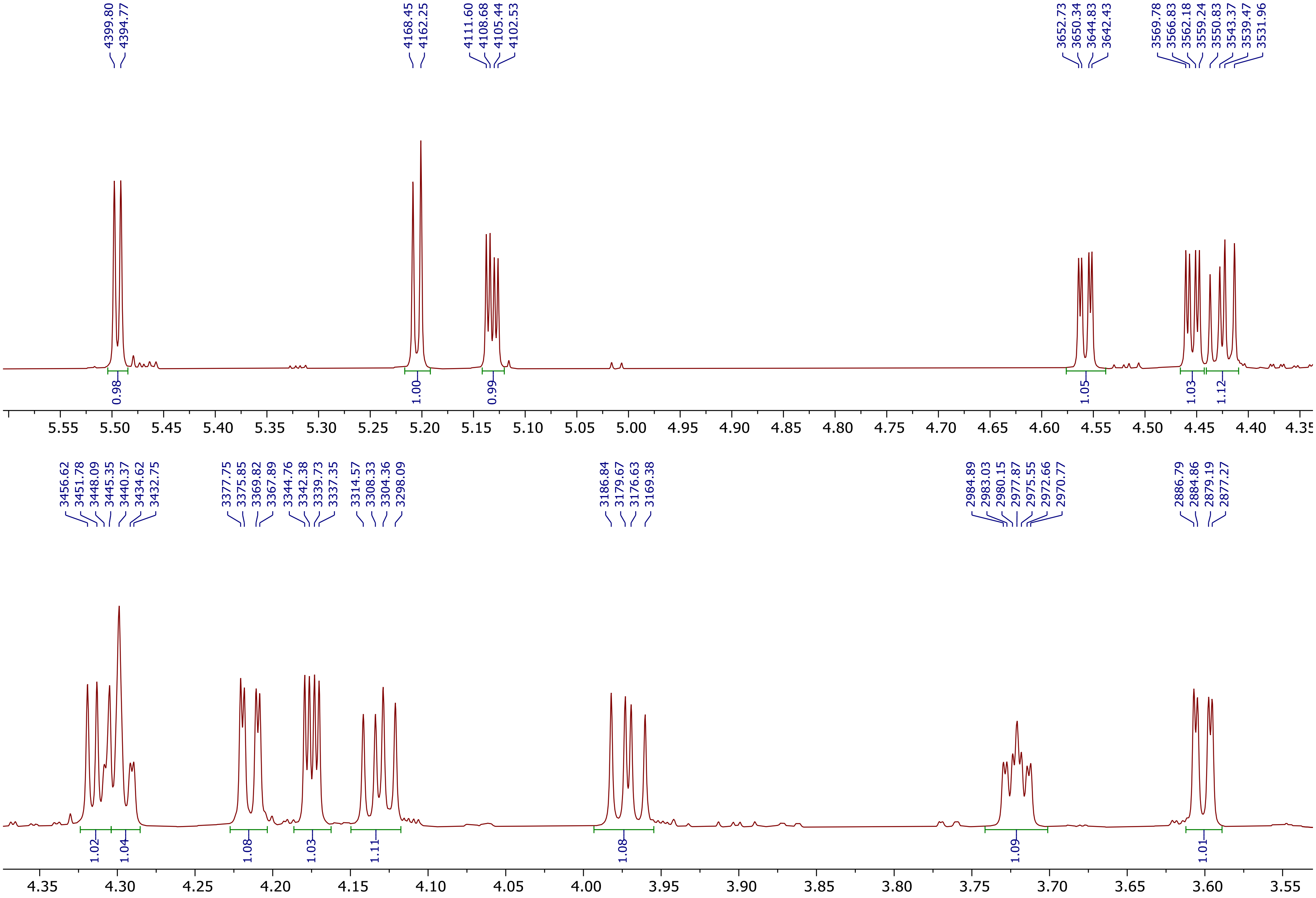

$^{13}C\{^{1}H\}$ NMR Spectrum of **27** (101 MHz, C_6D_6)

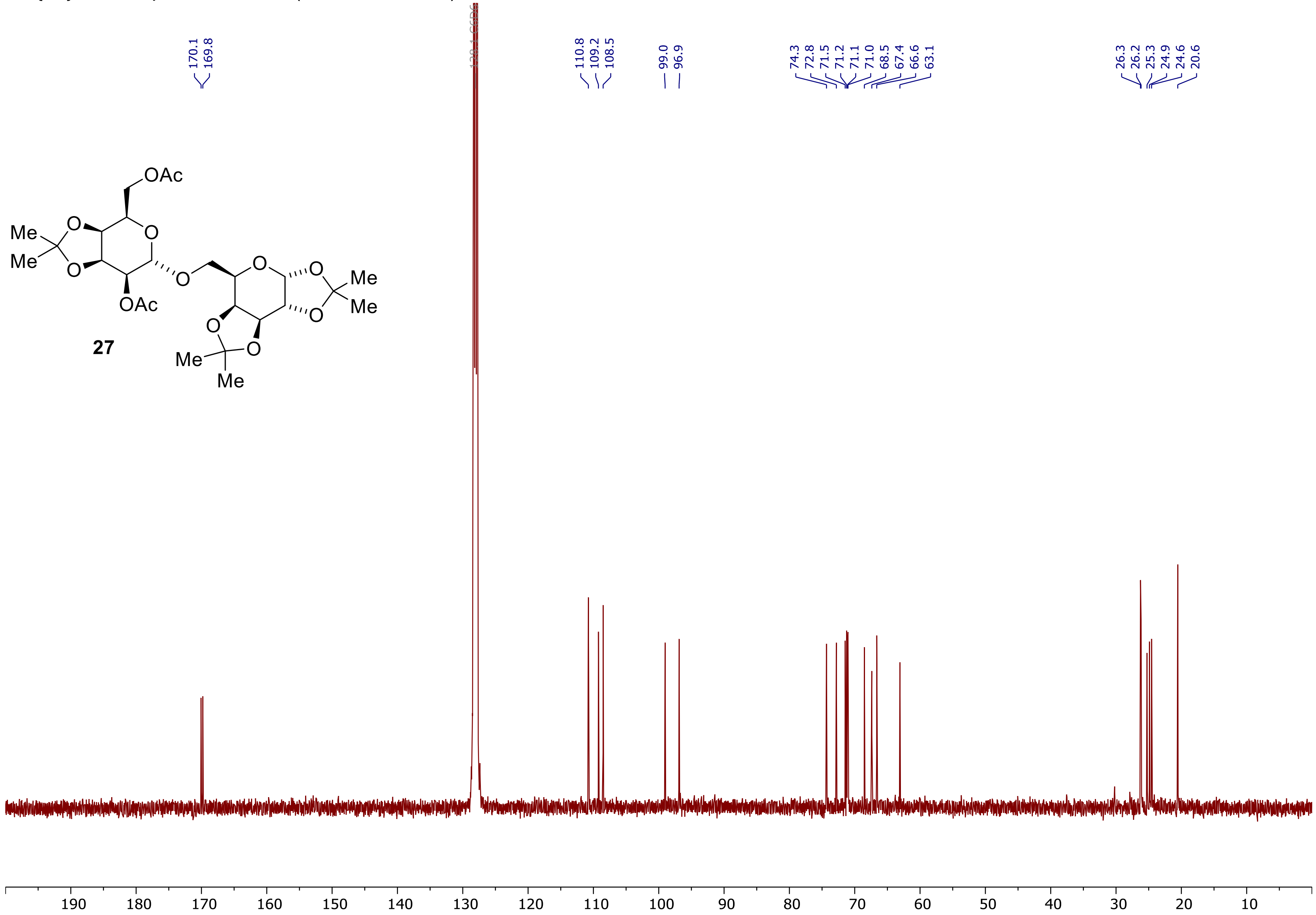

COSY spectrum of **27** (800 MHz, C_6D_6) – full spectrum

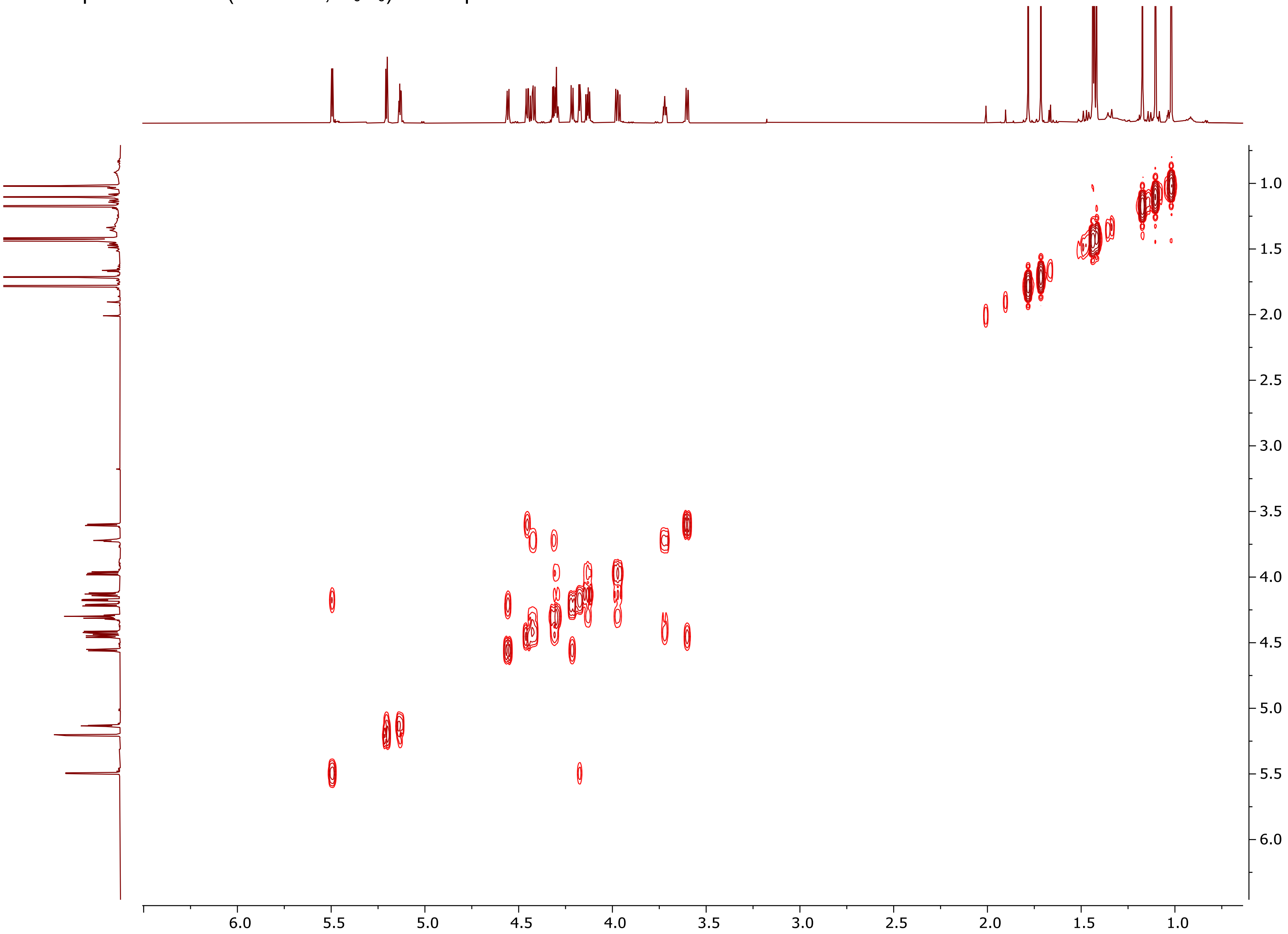

COSY spectrum of **27** (800 MHz, C_6D_6) – expansion

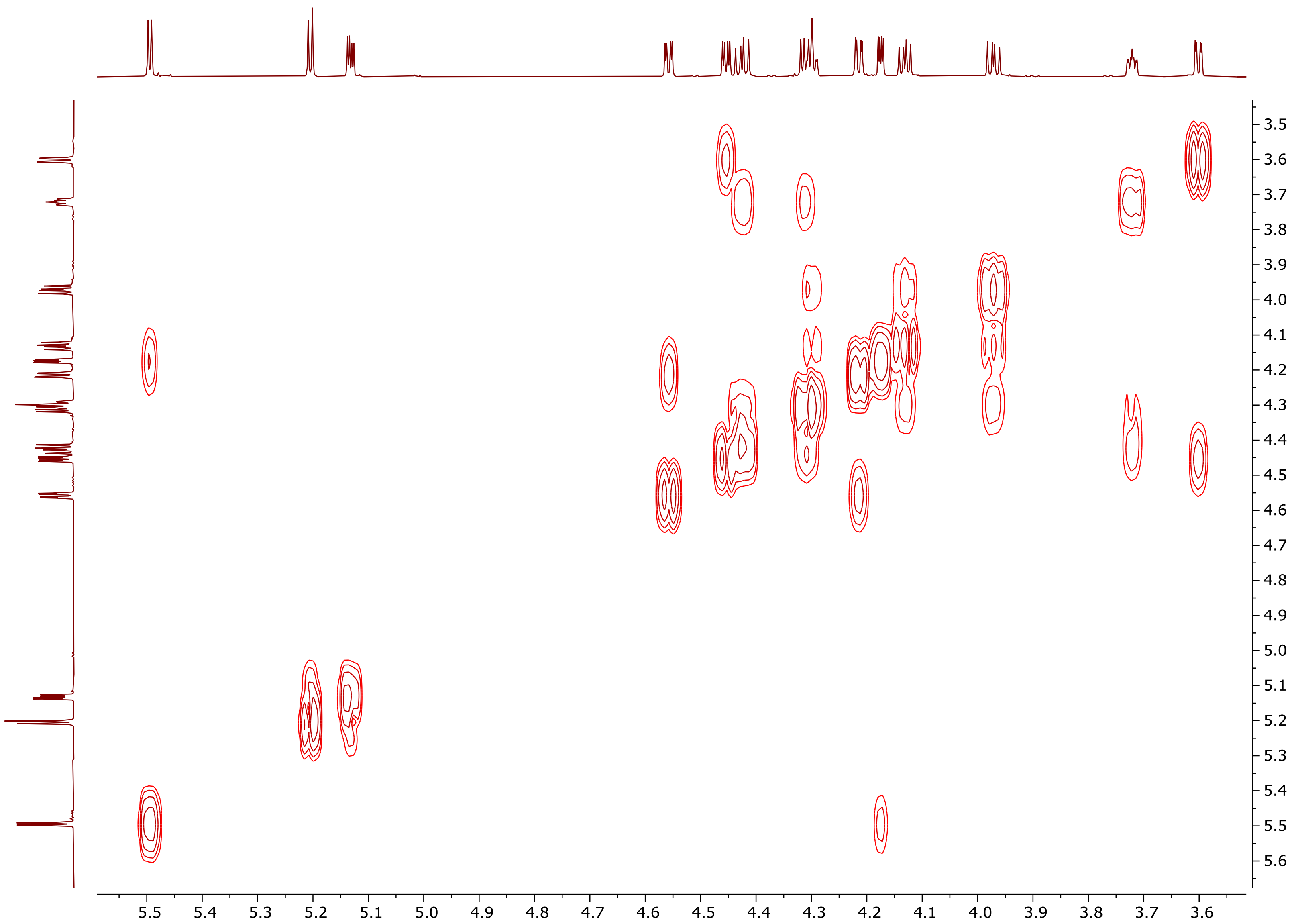

HSQC spectrum of **27** (800 MHz, C_6D_6) – full spectrum

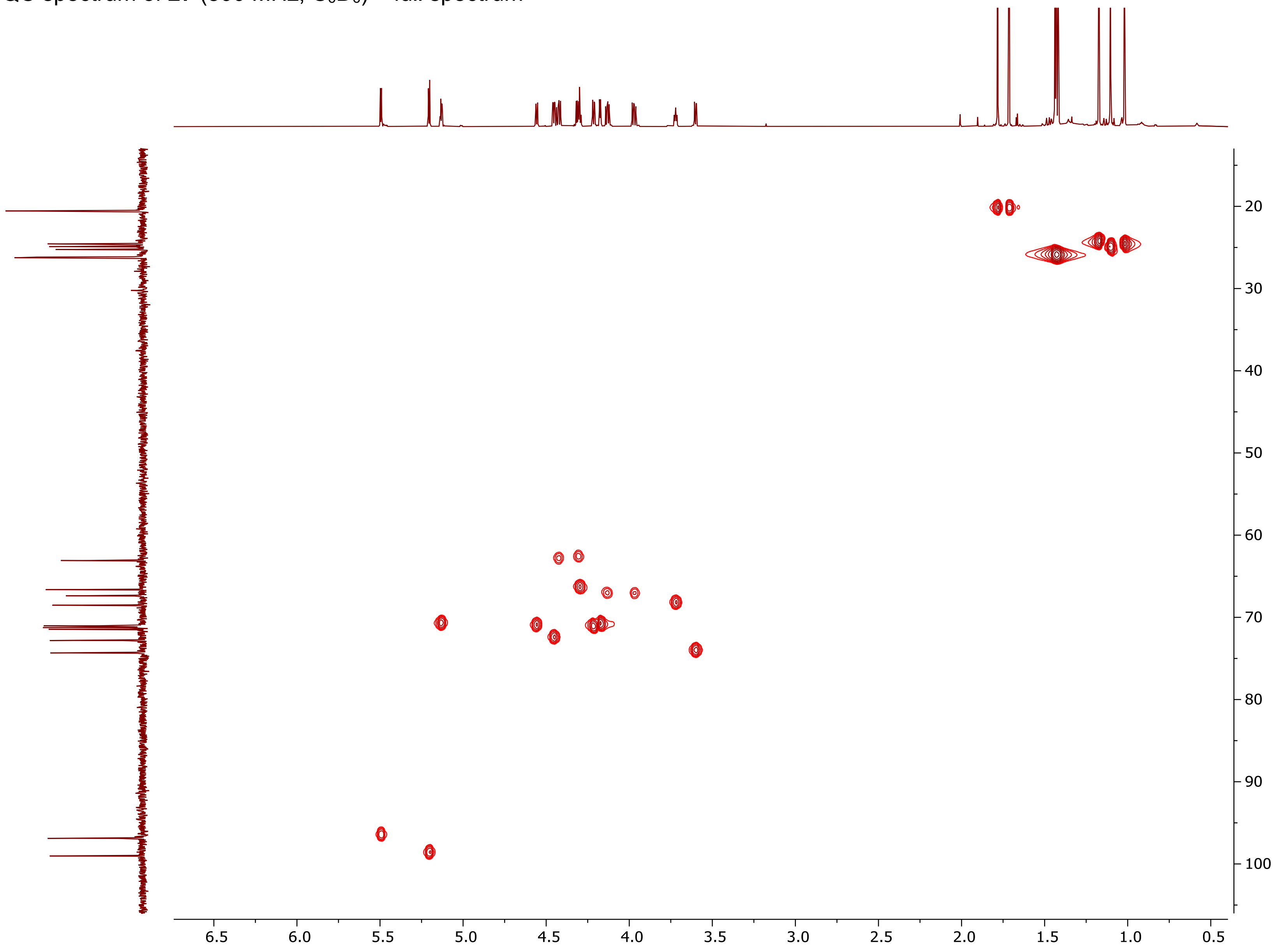

HSQC spectrum of **27** (800 MHz, C_6D_6) – expansion

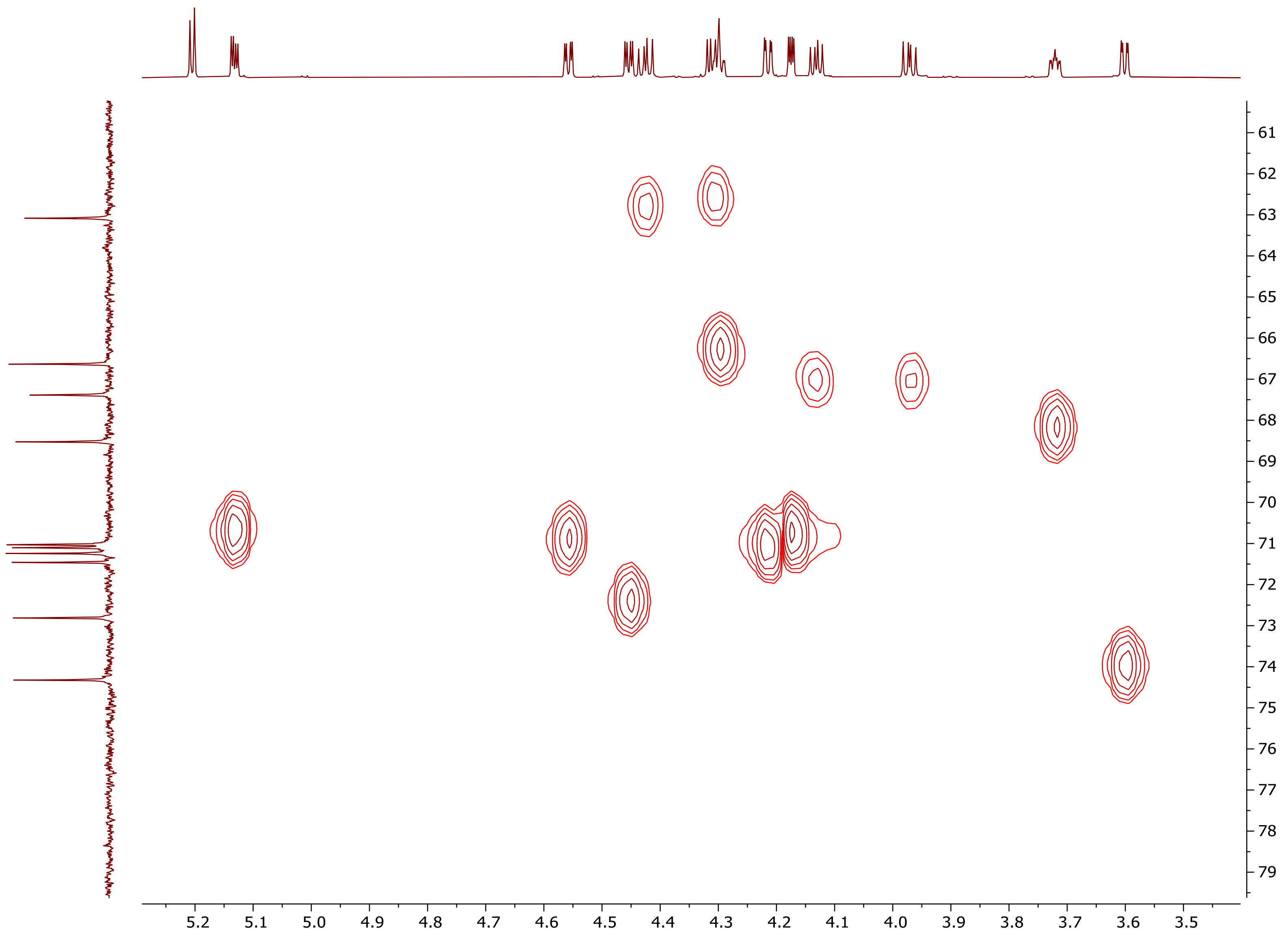

HMBC spectrum of **27** (800 MHz, C_6D_6) – full

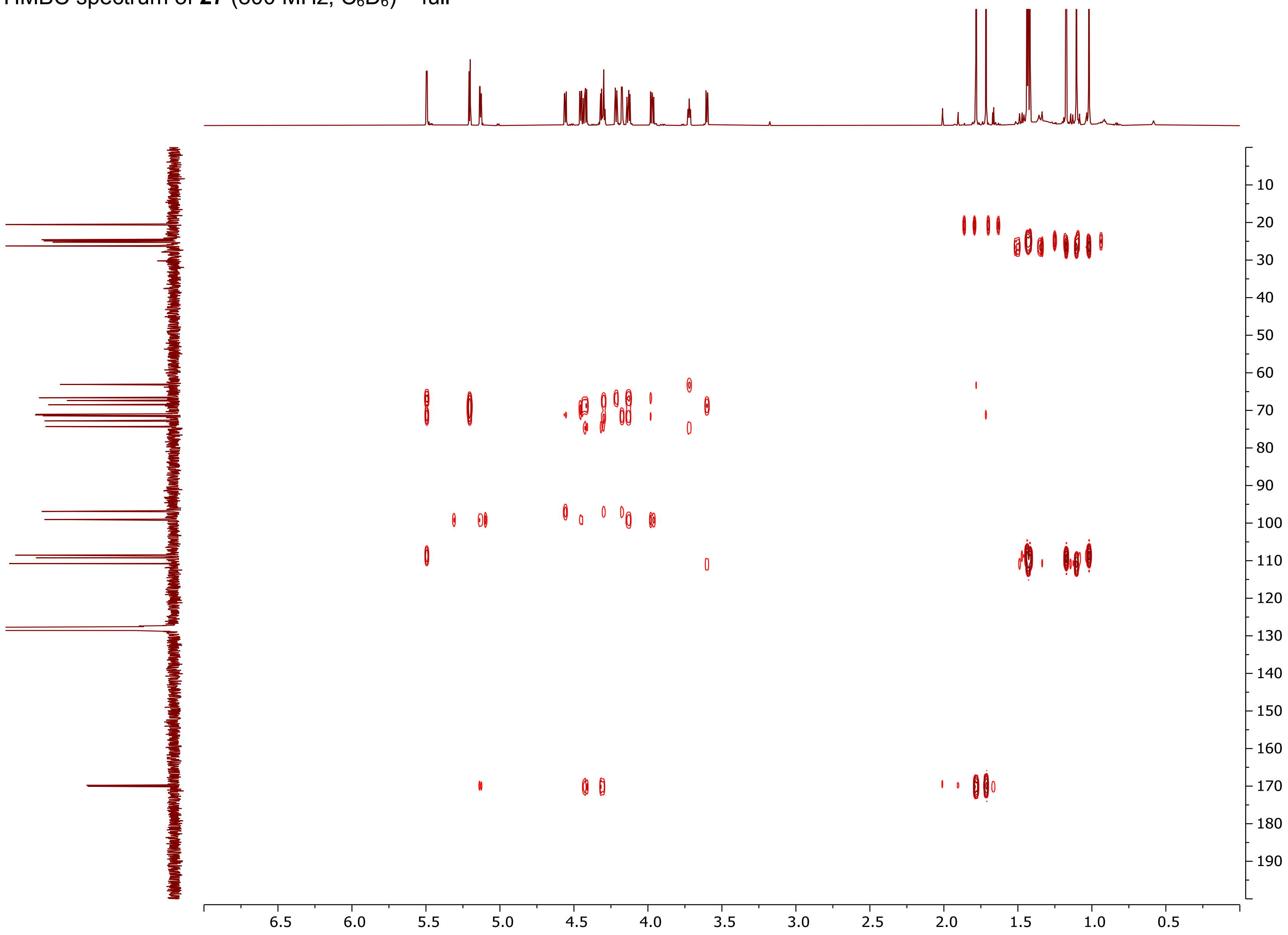

HMBC spectrum of **27** (800 MHz, C_6D_6) – expansion

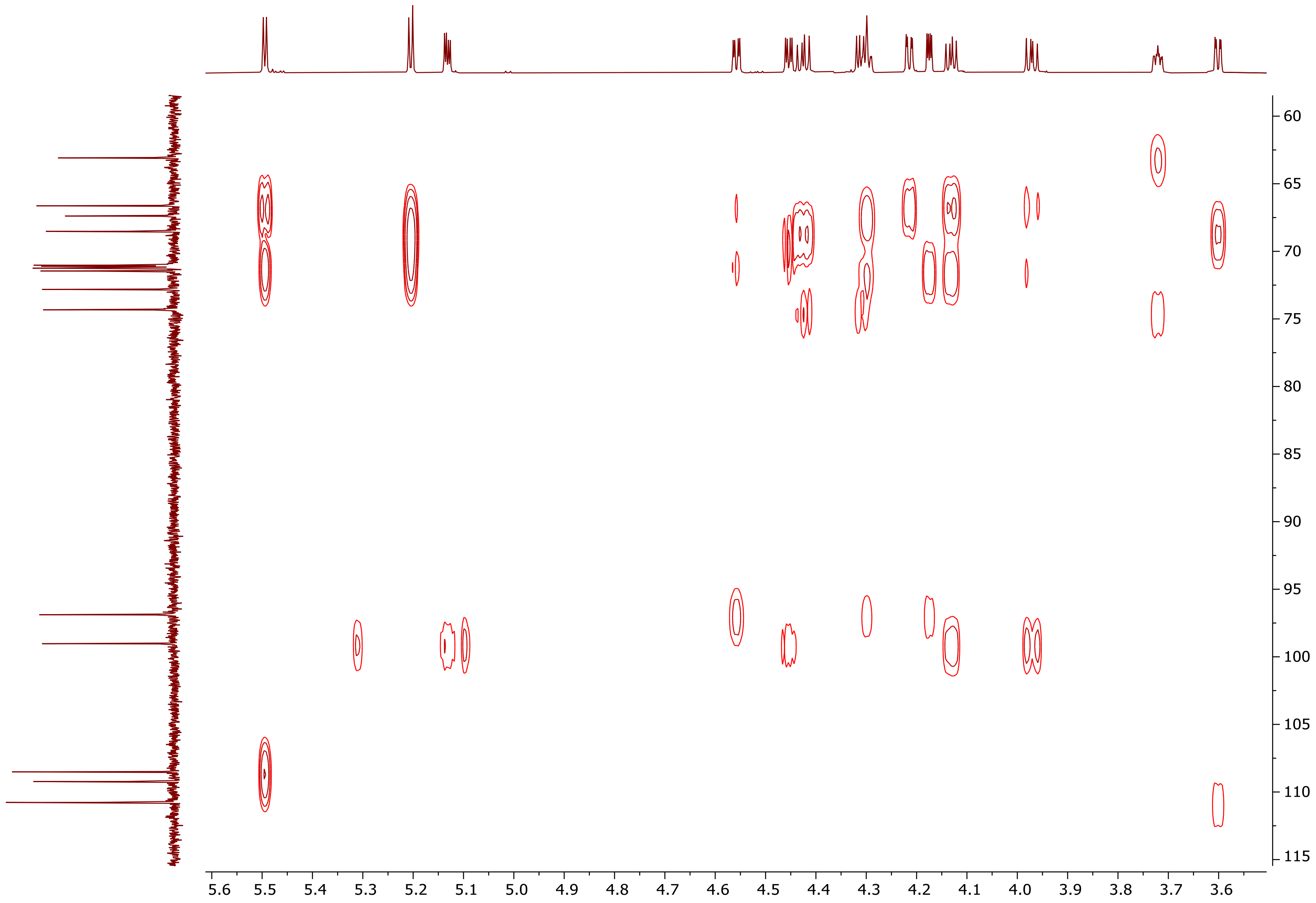

^{1}H NMR Spectrum of **28** (800 MHz, C_6D_6) – full spectrum

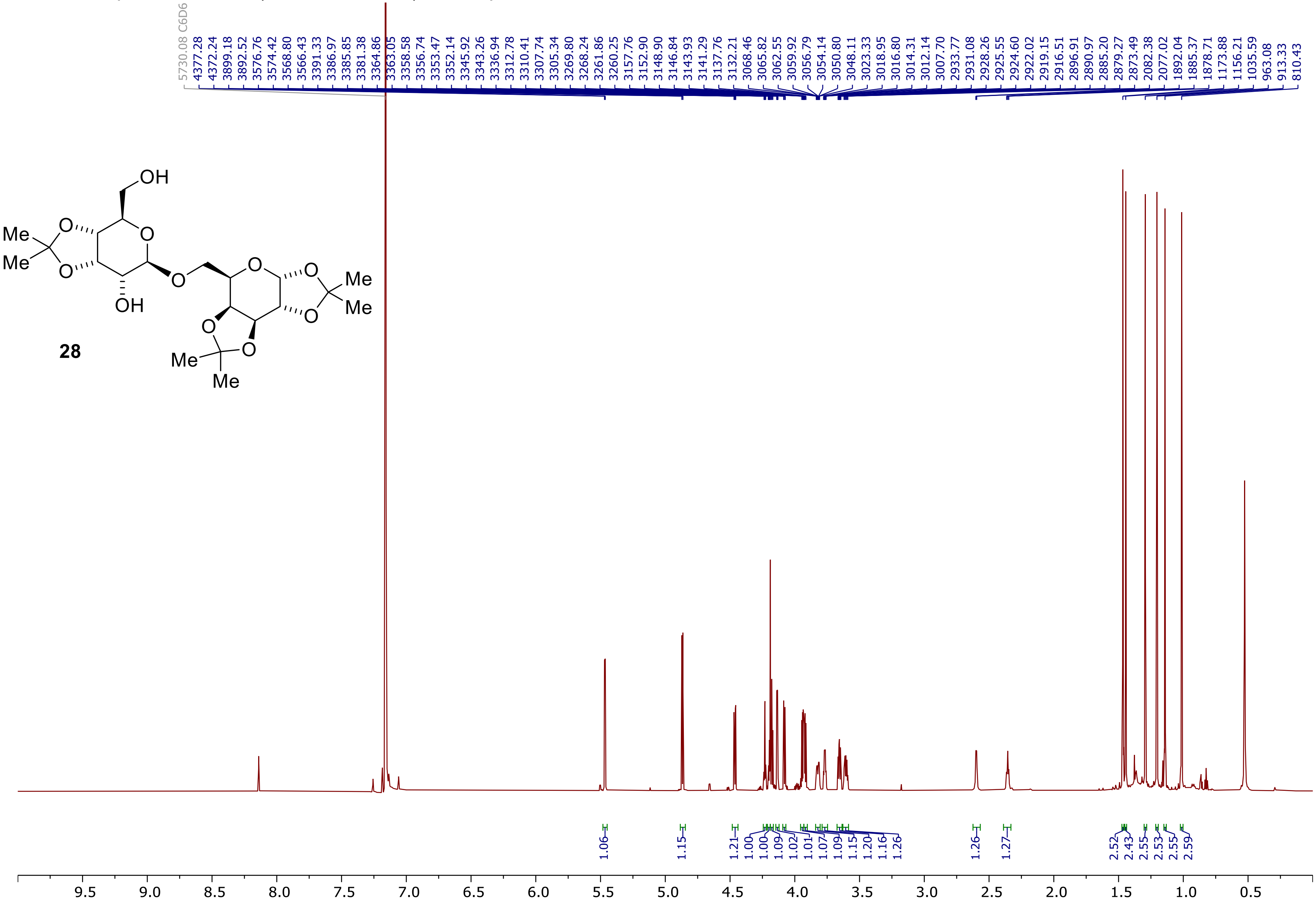

^{1}H NMR Spectrum of **28** (800 MHz, C_6D_6) – expansion

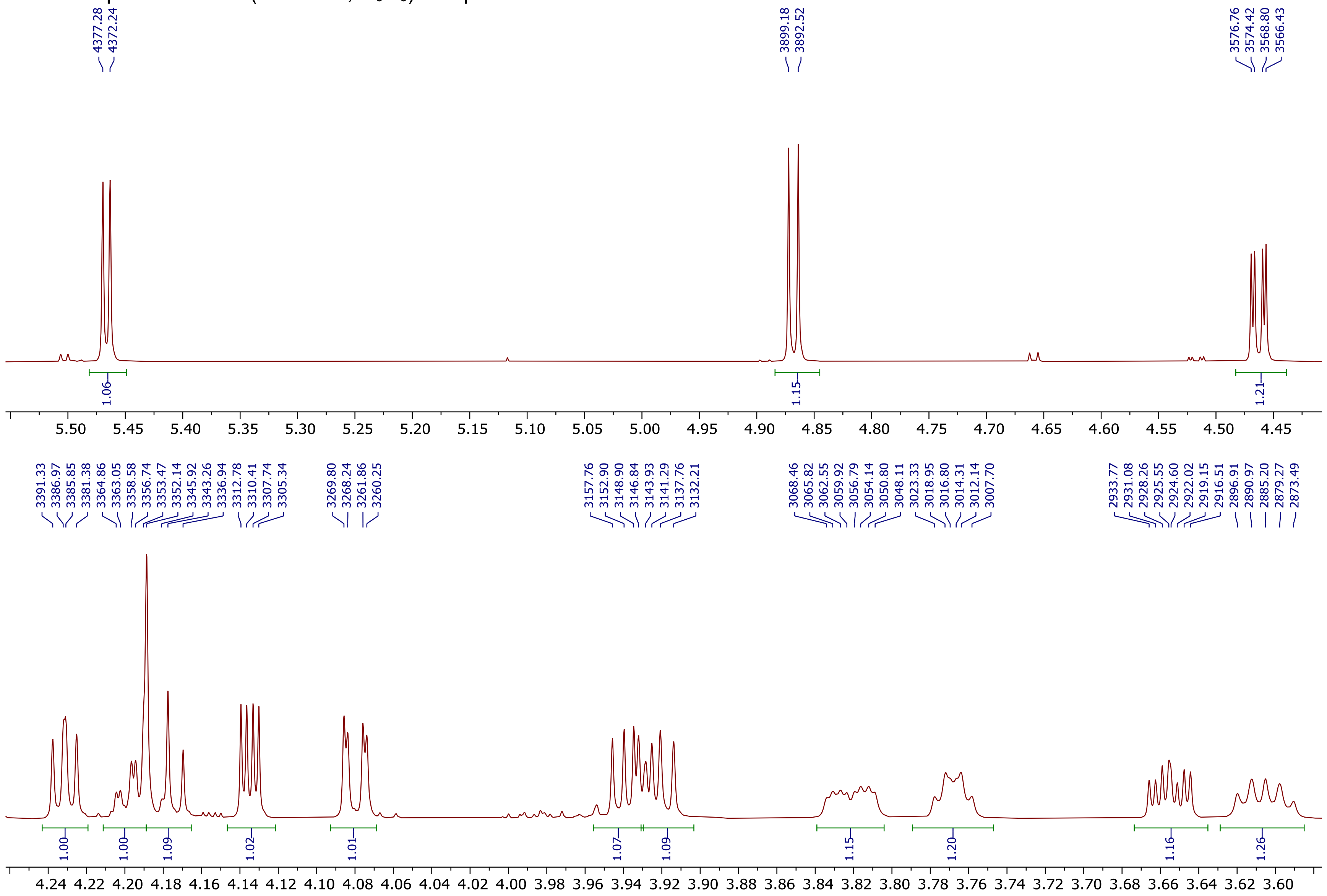

$^{13}C\{^{1}H\}$ NMR Spectrum of **28** (101 MHz, C_6D_6)

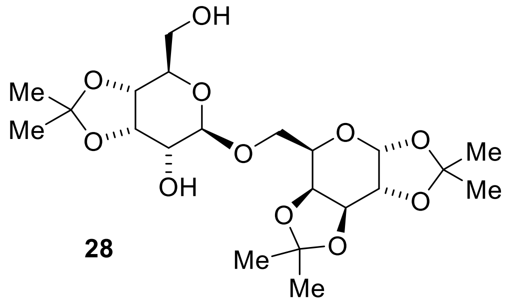

28

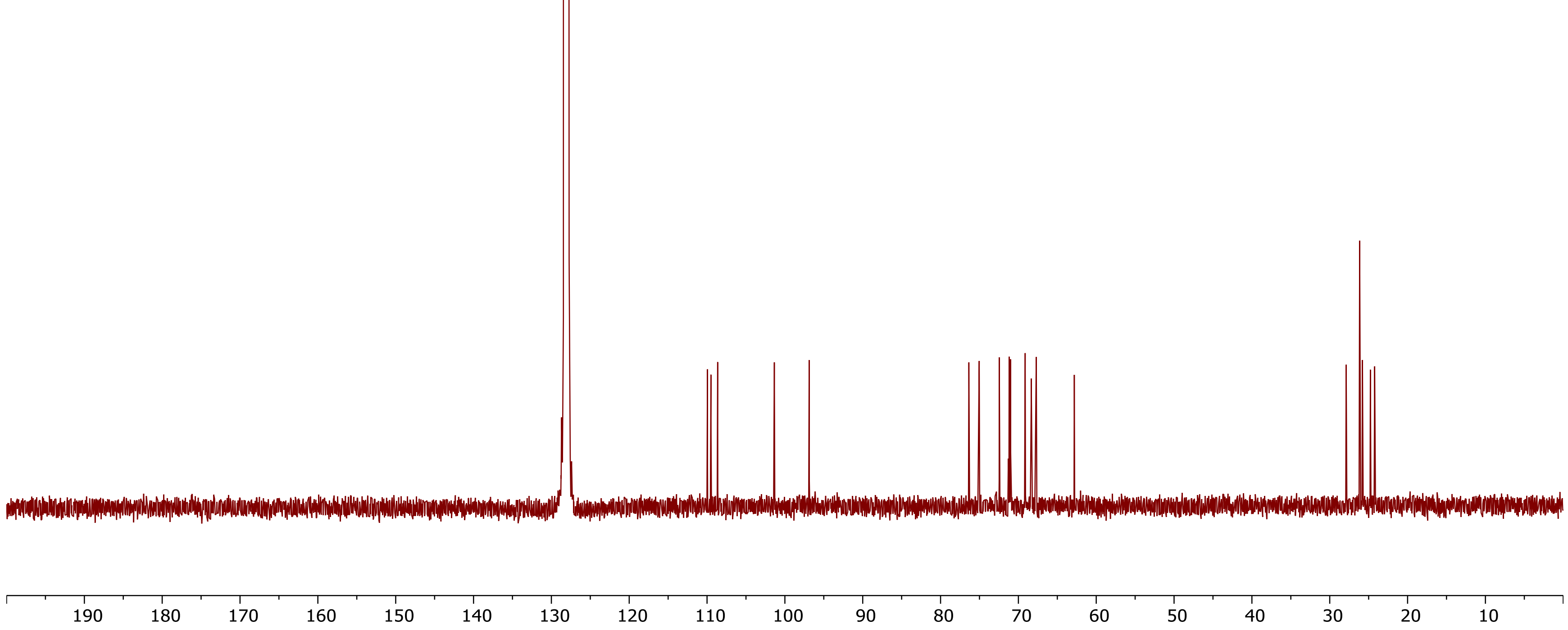

COSY spectrum of **28** (800 MHz, C_6D_6) – full spectrum

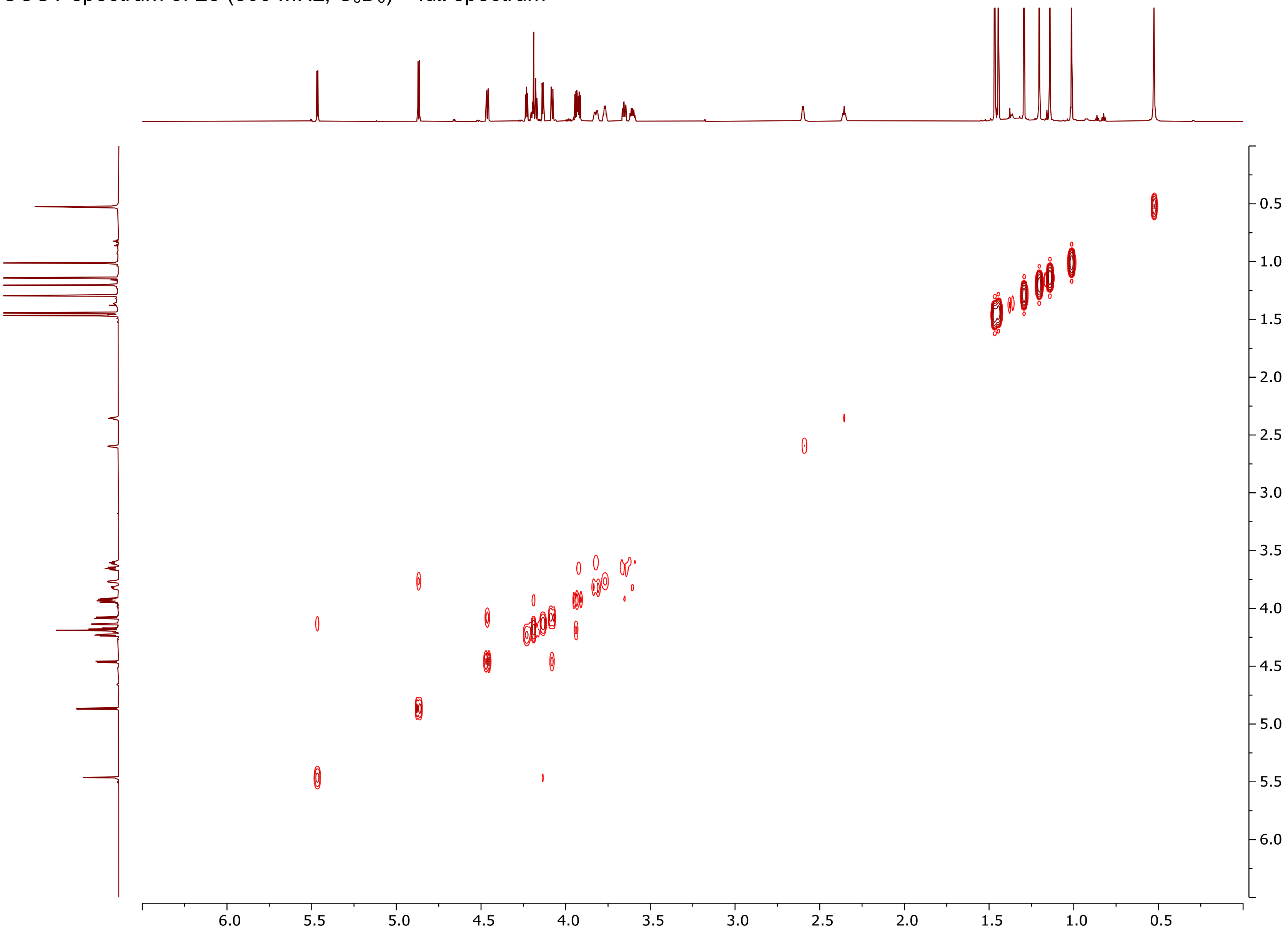

COSY spectrum of **28** (800 MHz, C_6D_6) – expansion

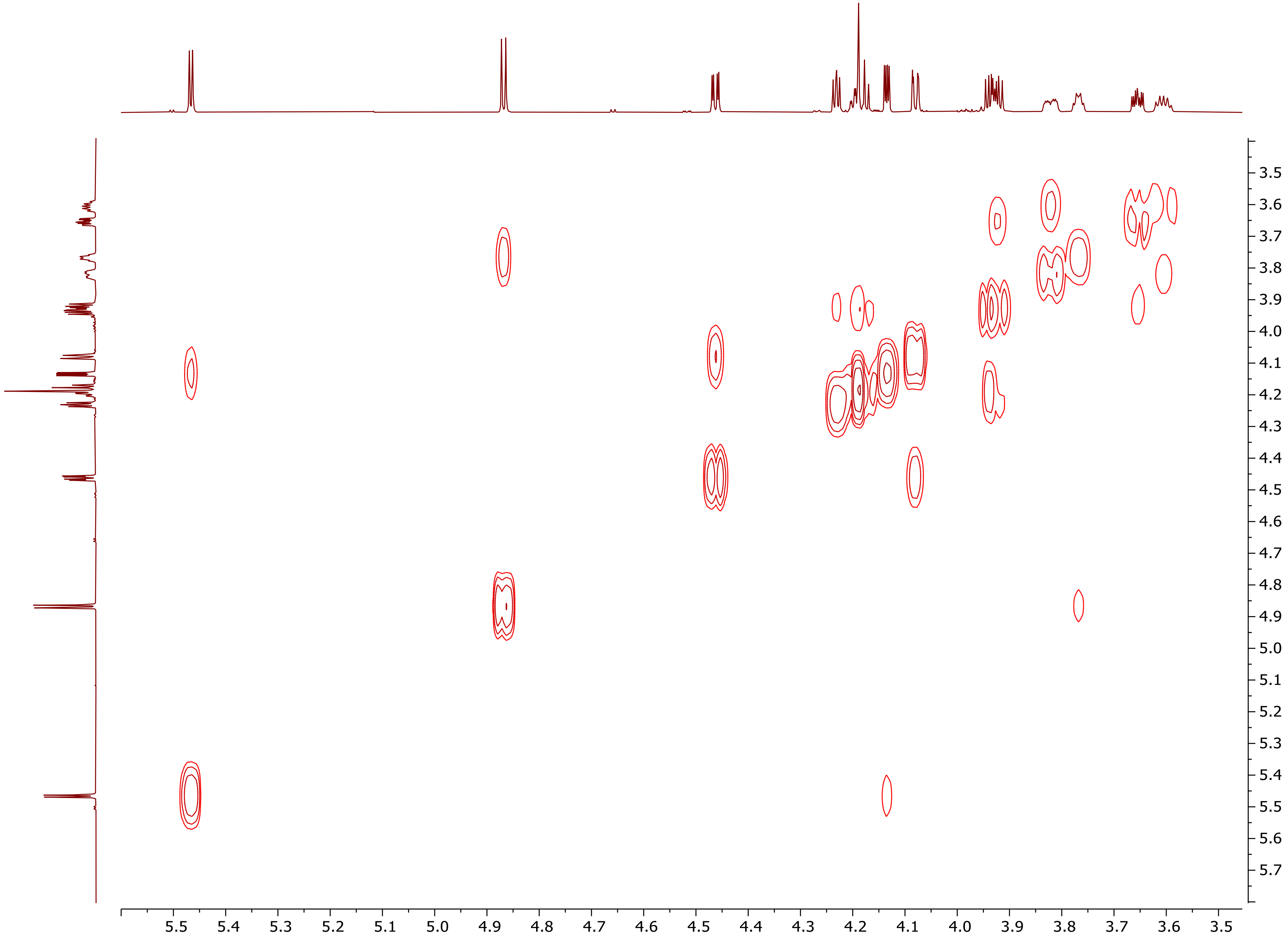

HSQC spectrum of **28** (800 MHz, C_6D_6) – full spectrum

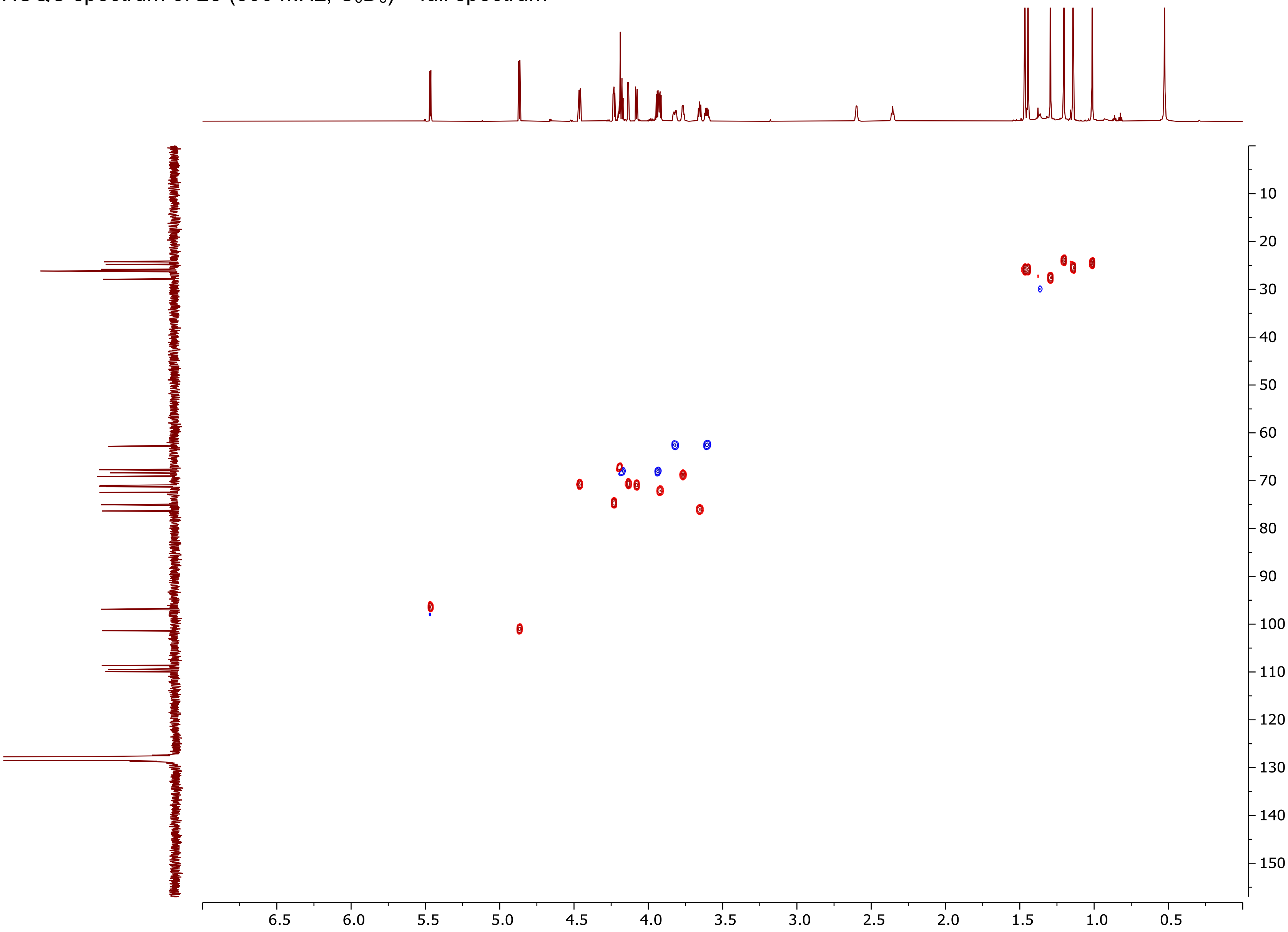

HSQC spectrum of **28** (800 MHz, C_6D_6) – expansion

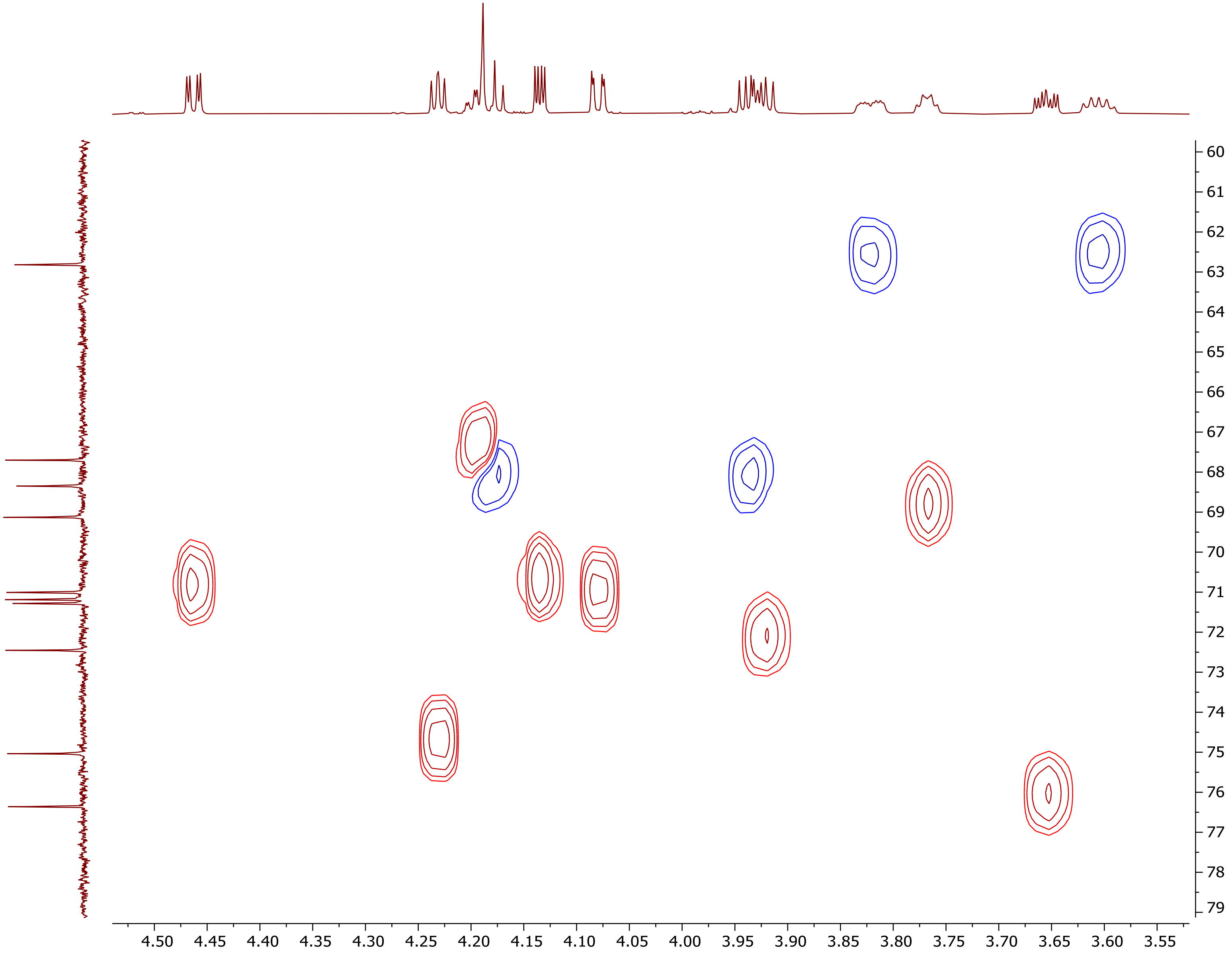

HMBC spectrum of **28** (800 MHz, C_6D_6) – full spectrum

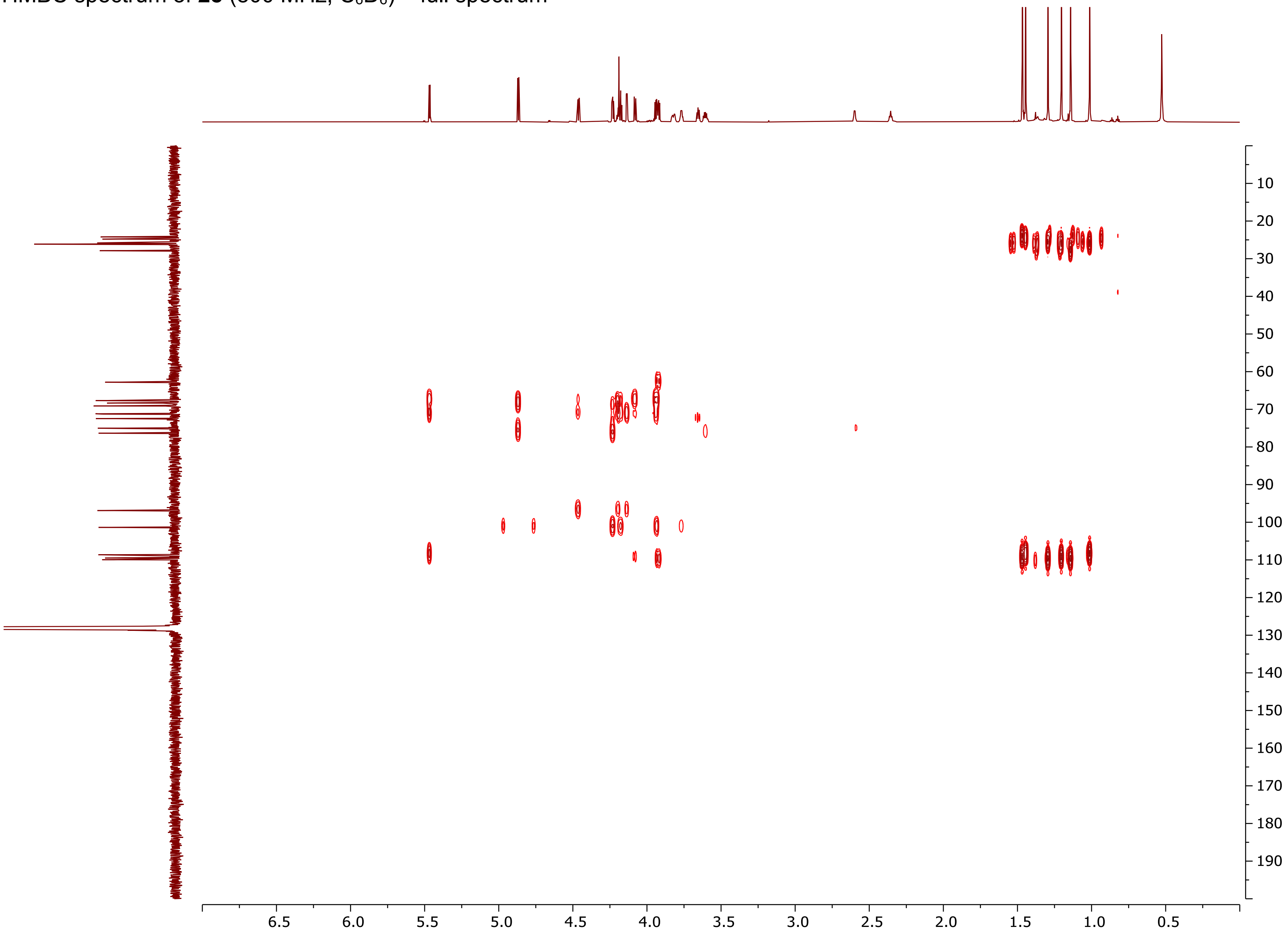

HMBC spectrum of **28** (800 MHz, C_6D_6) – expansion

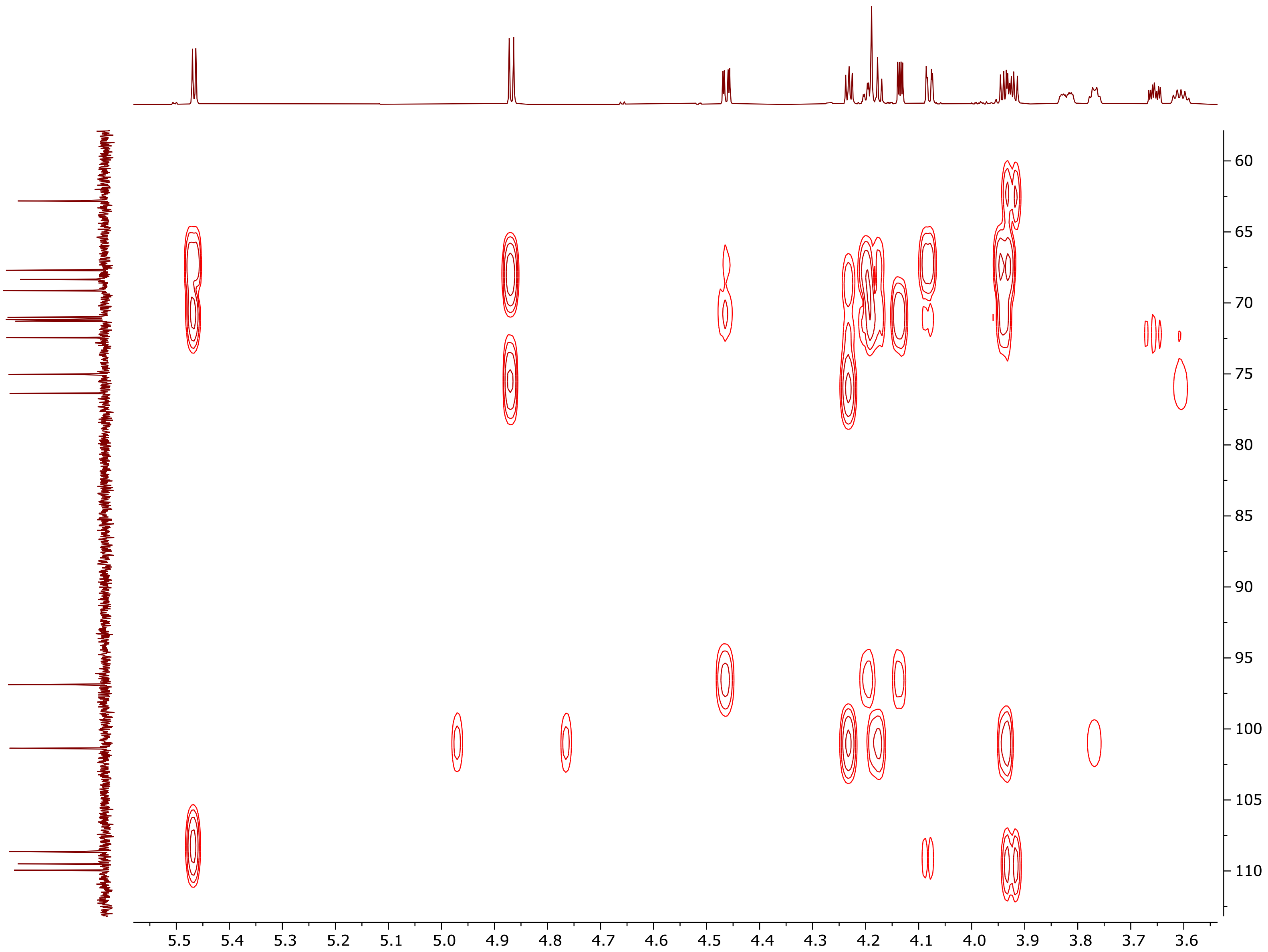

^{1}H NMR Spectrum of **29** (800 MHz, C_6D_6) – full spectrum

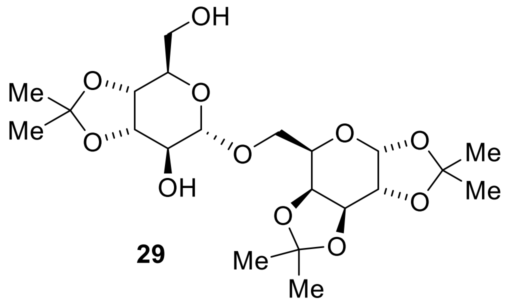

29

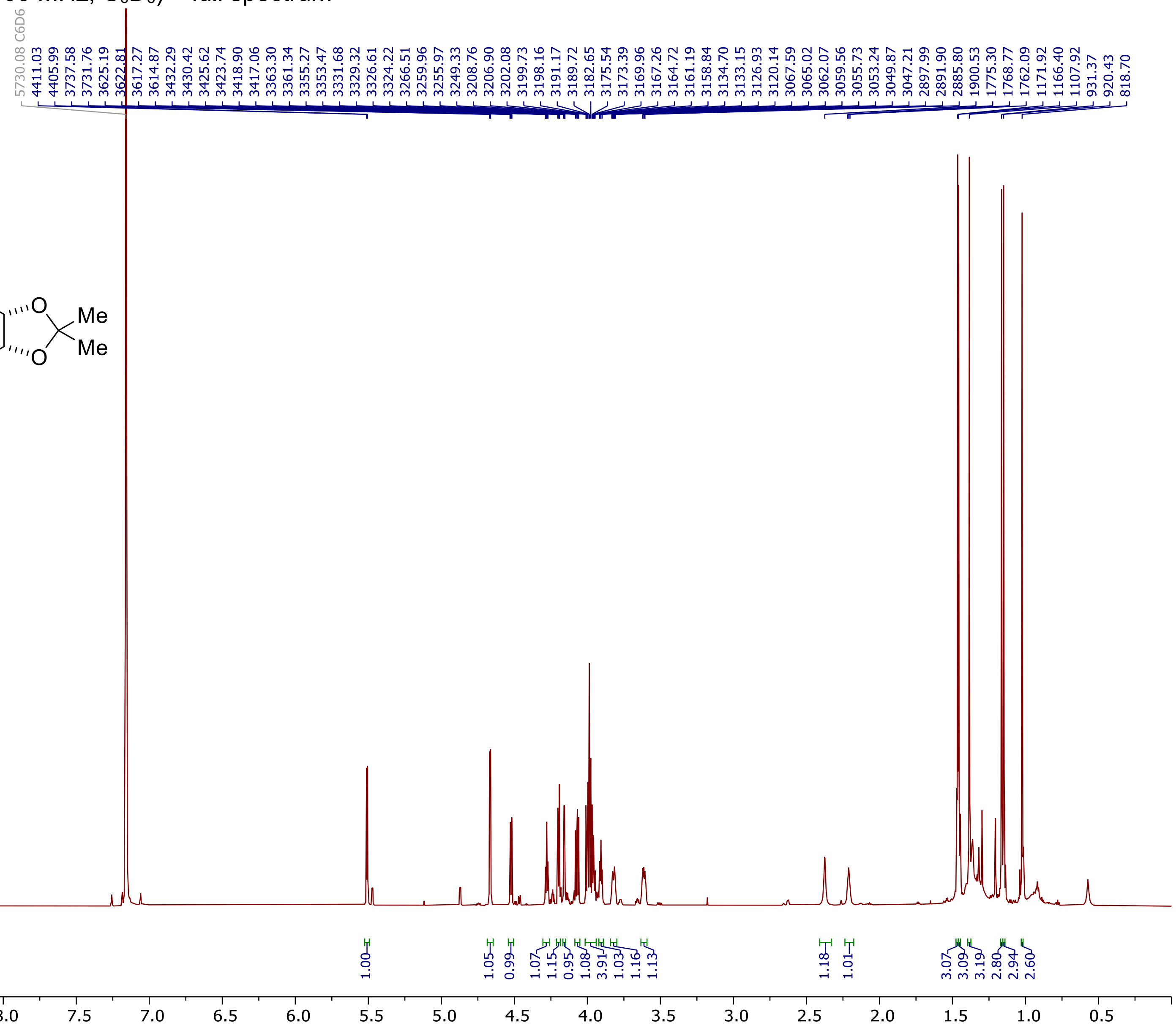

^{1}H NMR Spectrum of **29** (800 MHz, C_6D_6) – expansion

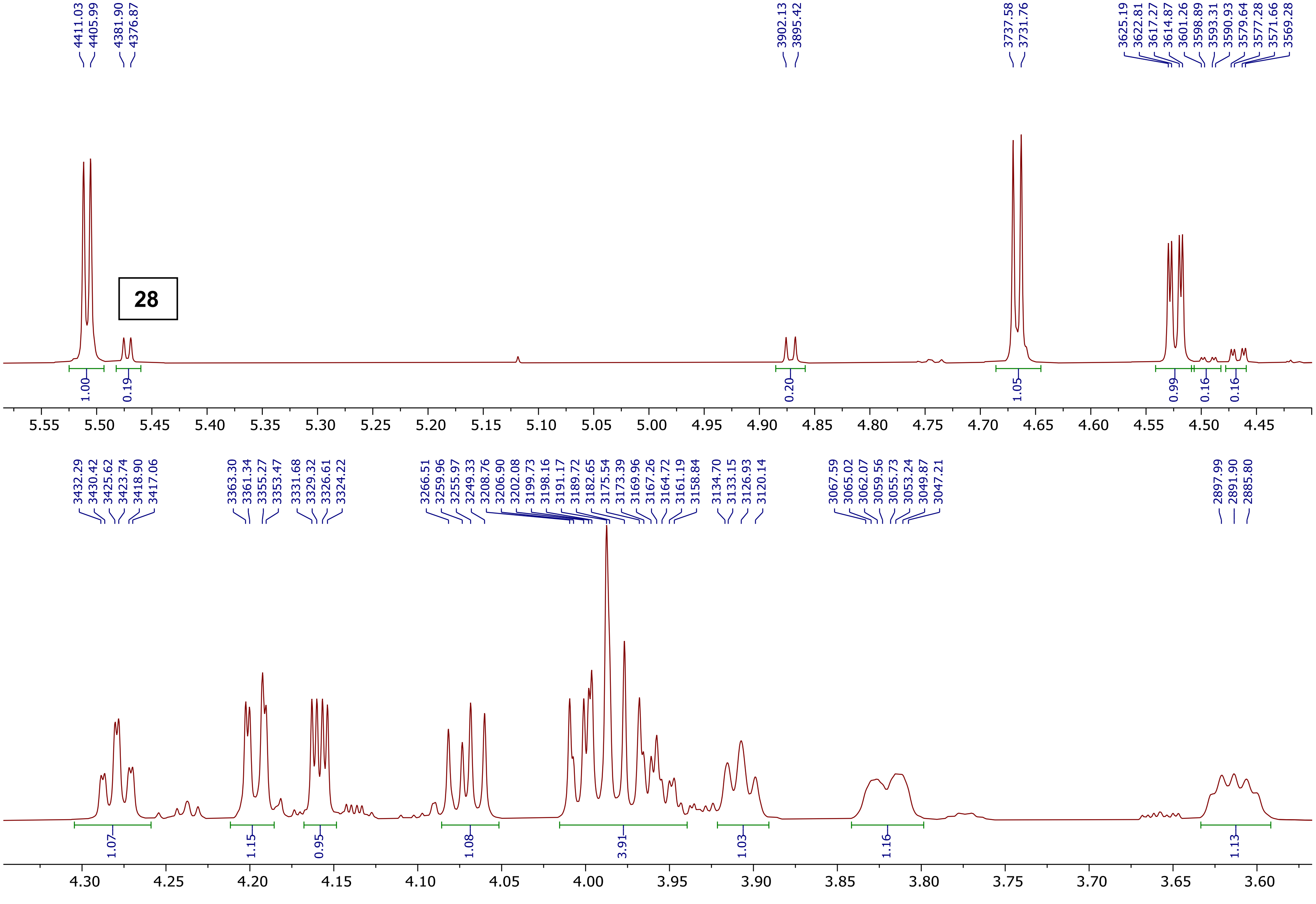

$^{13}C\{^{1}H\}$ NMR Spectrum of **29** (101 MHz, C_6D_6)

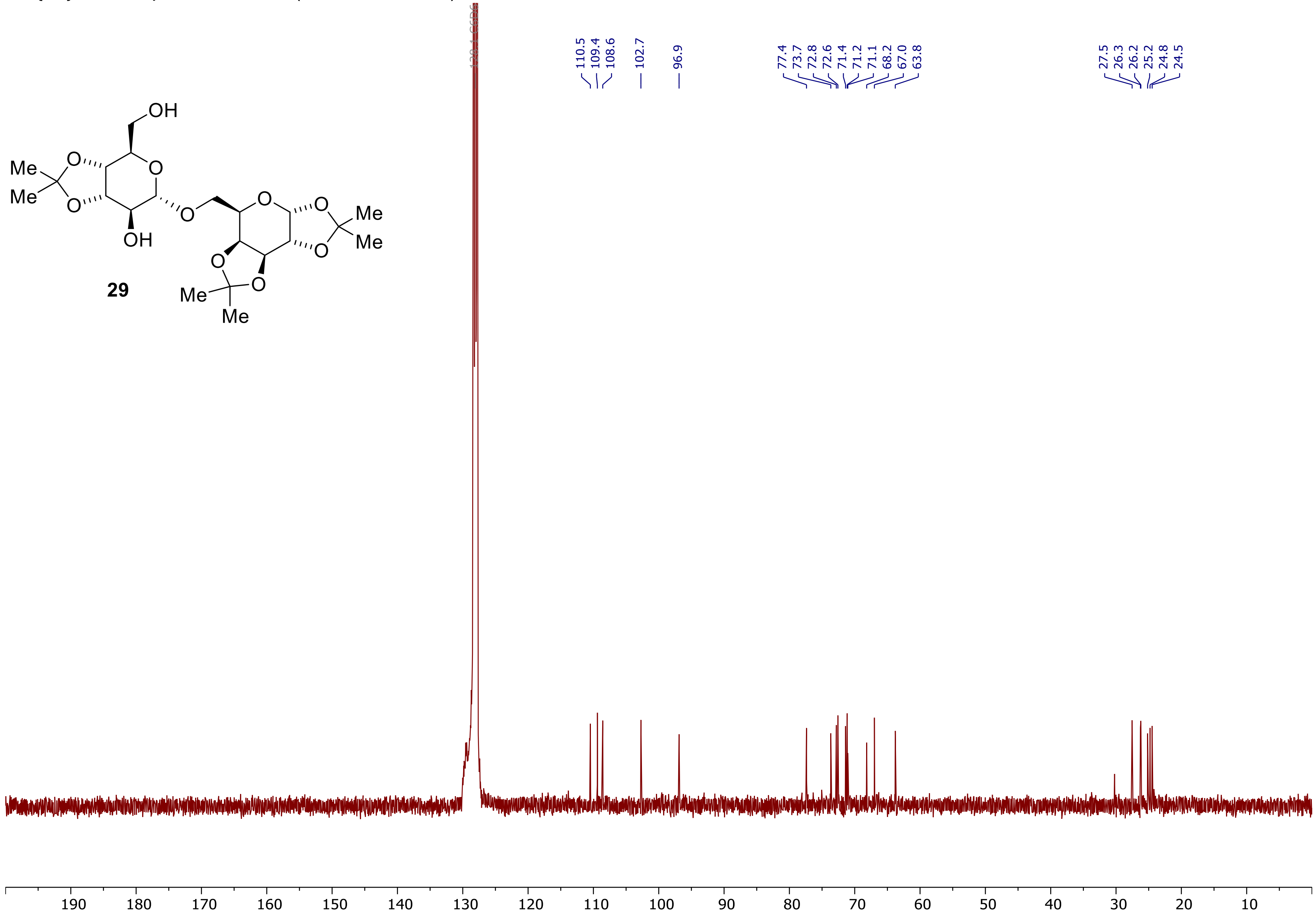

^{1}H NMR Spectrum of **30** (800 MHz, C_6D_6) – full spectrum

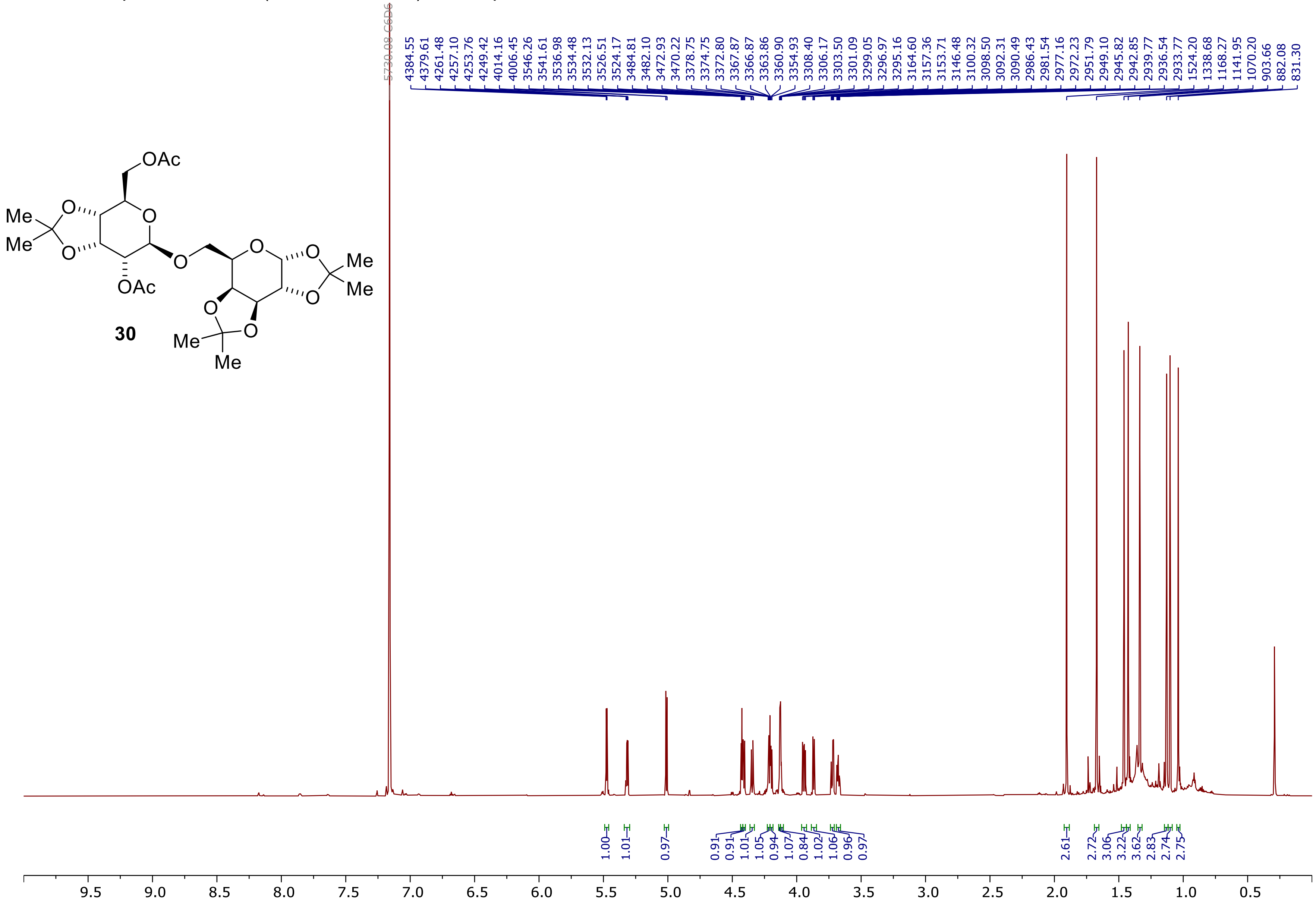

30

^{1}H NMR Spectrum of **30** (800 MHz, C_6D_6) – expansion

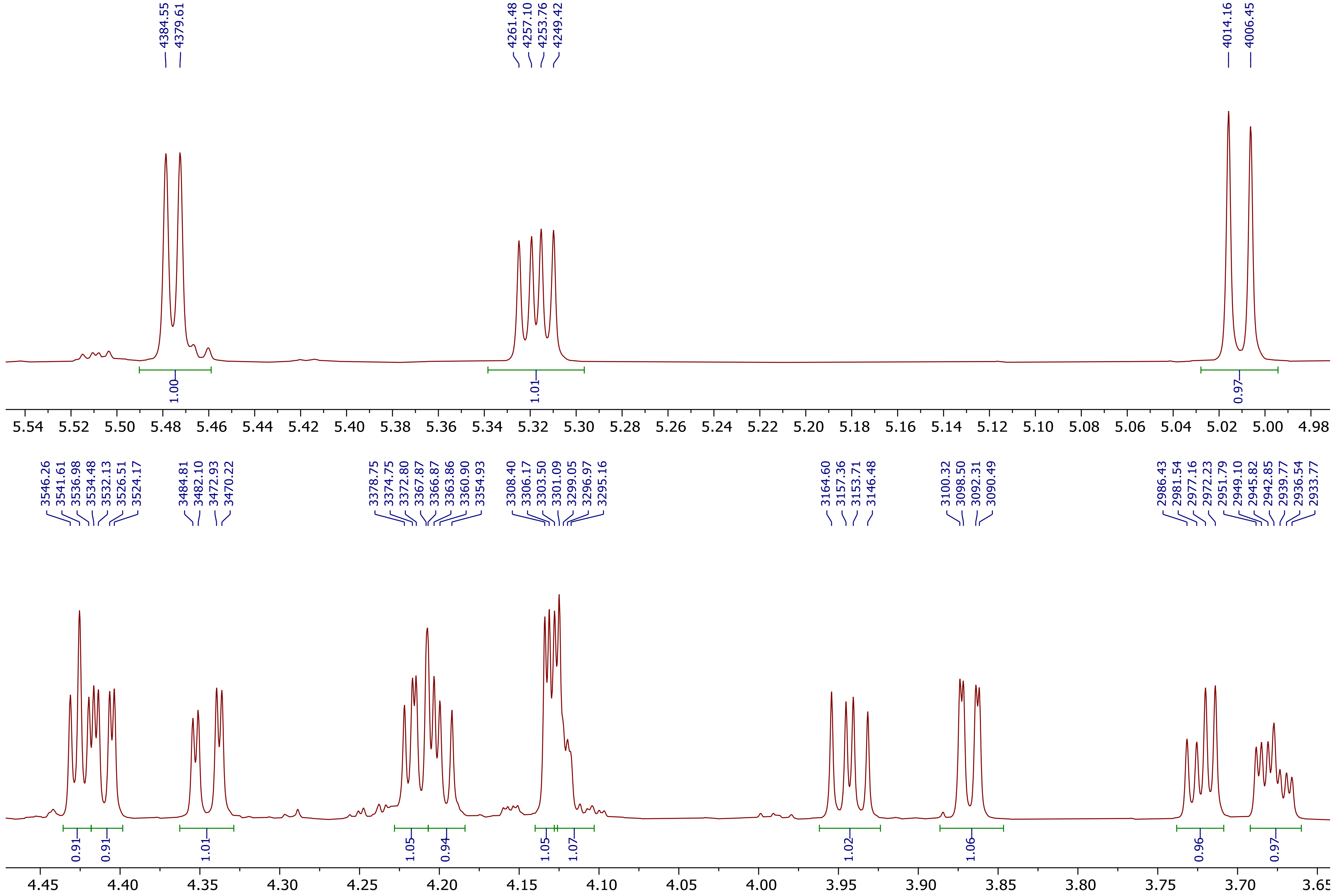

$^{13}C\{^{1}H\}$ NMR Spectrum of **30** (101 MHz, C_6D_6)

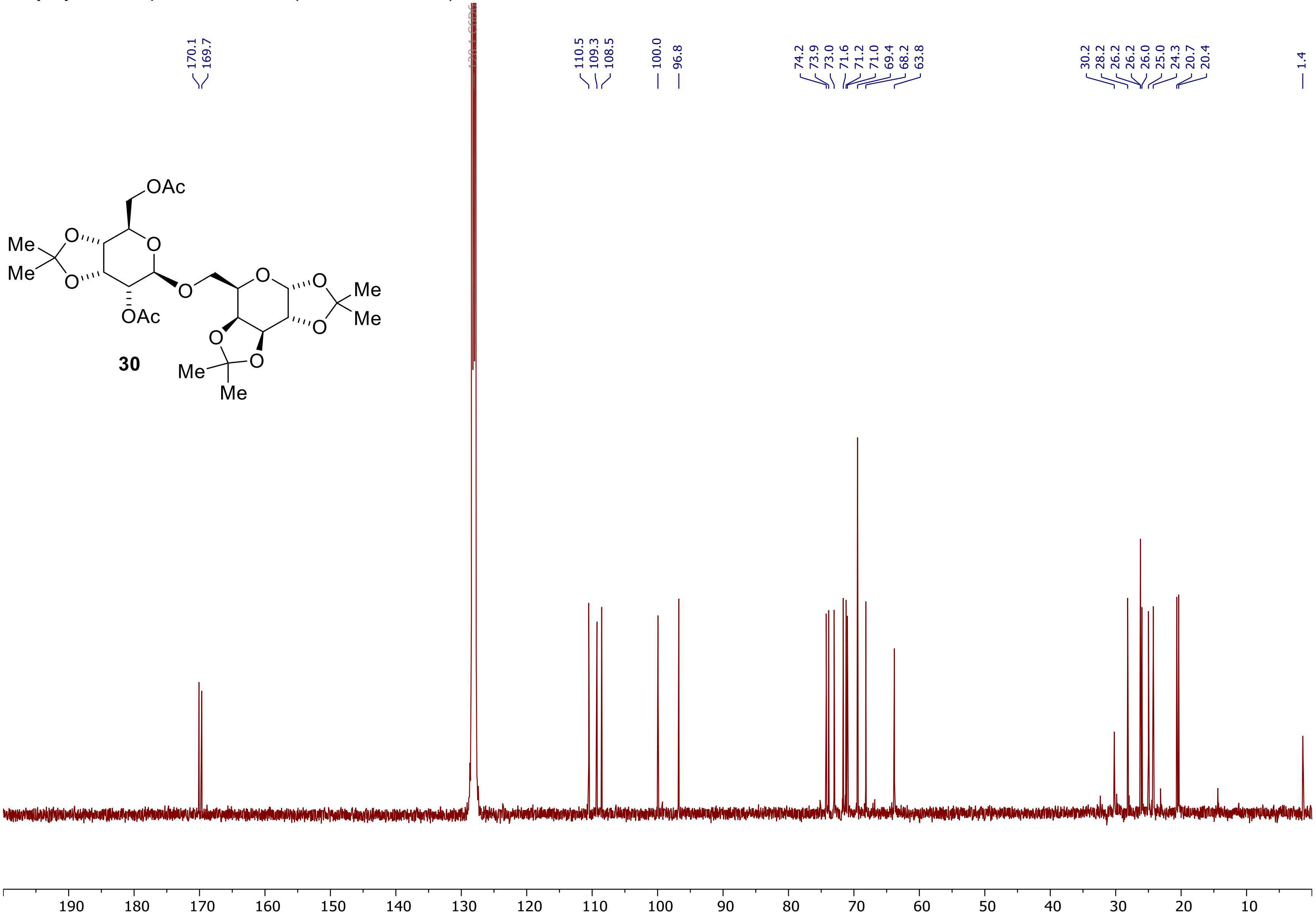

COSY spectrum of **30** (800 MHz, C_6D_6) – full spectrum

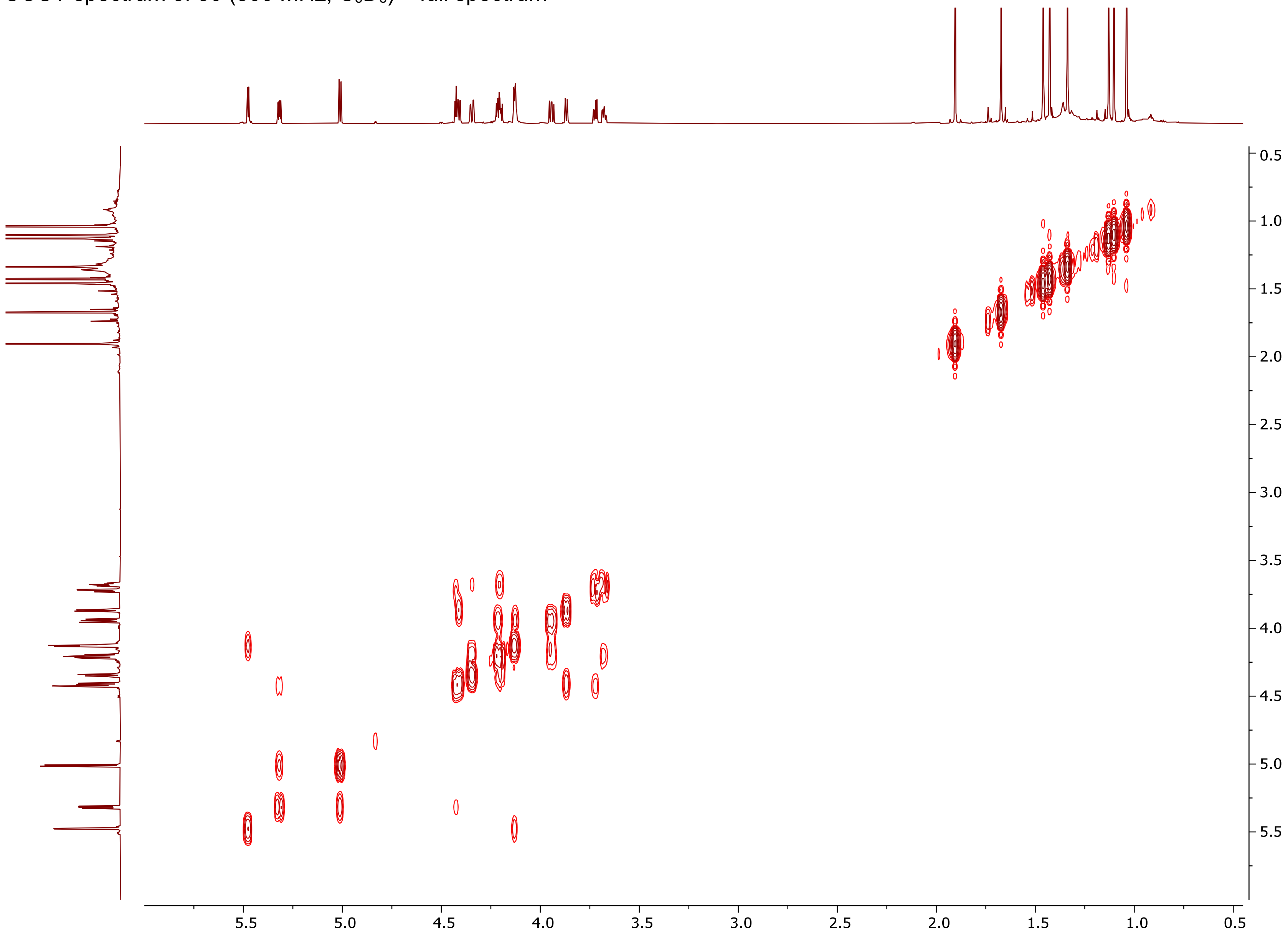

COSY spectrum of **30** (800 MHz, C_6D_6) – expansion

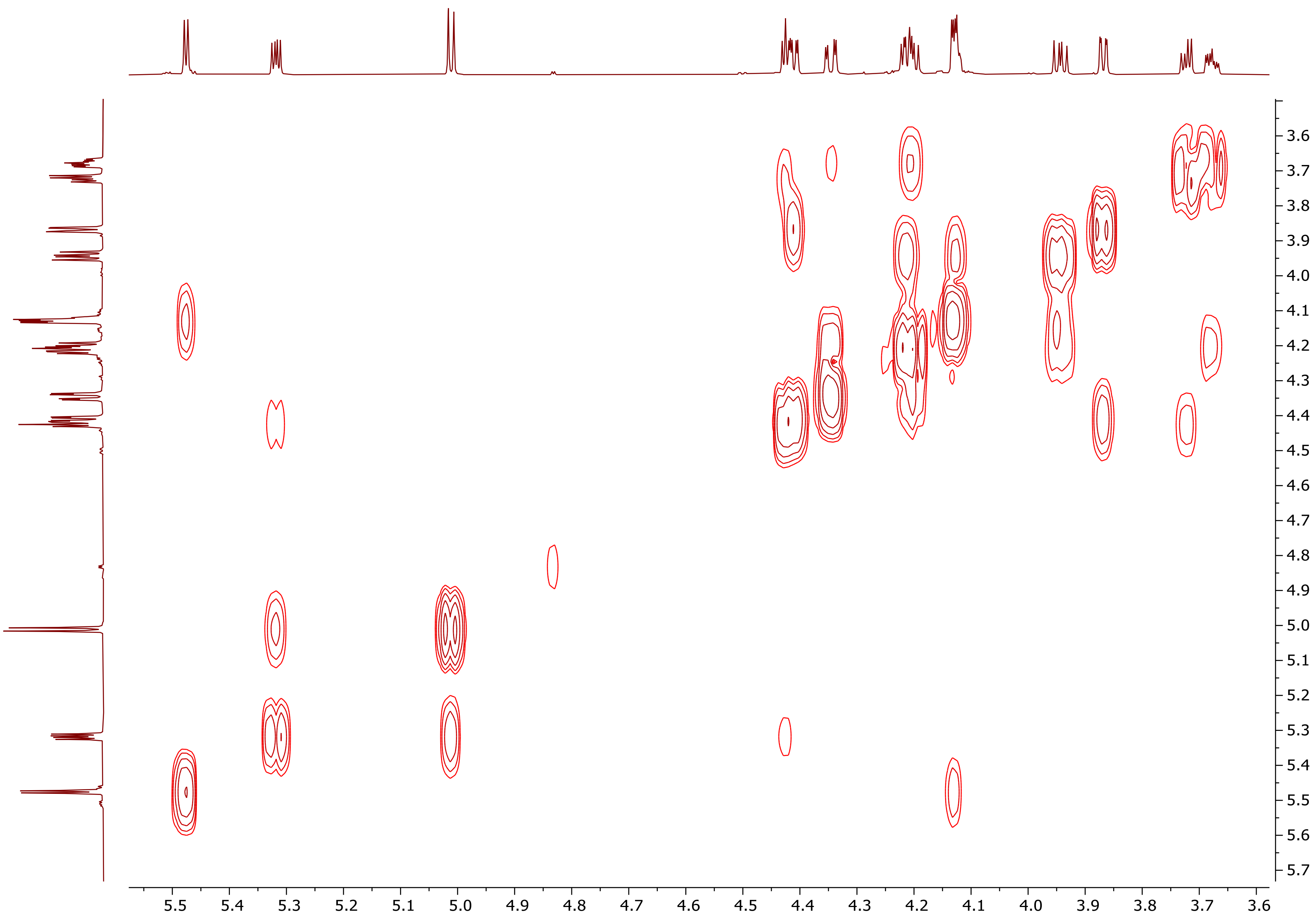

HSQC spectrum of **30** (800 MHz, C_6D_6) – full spectrum

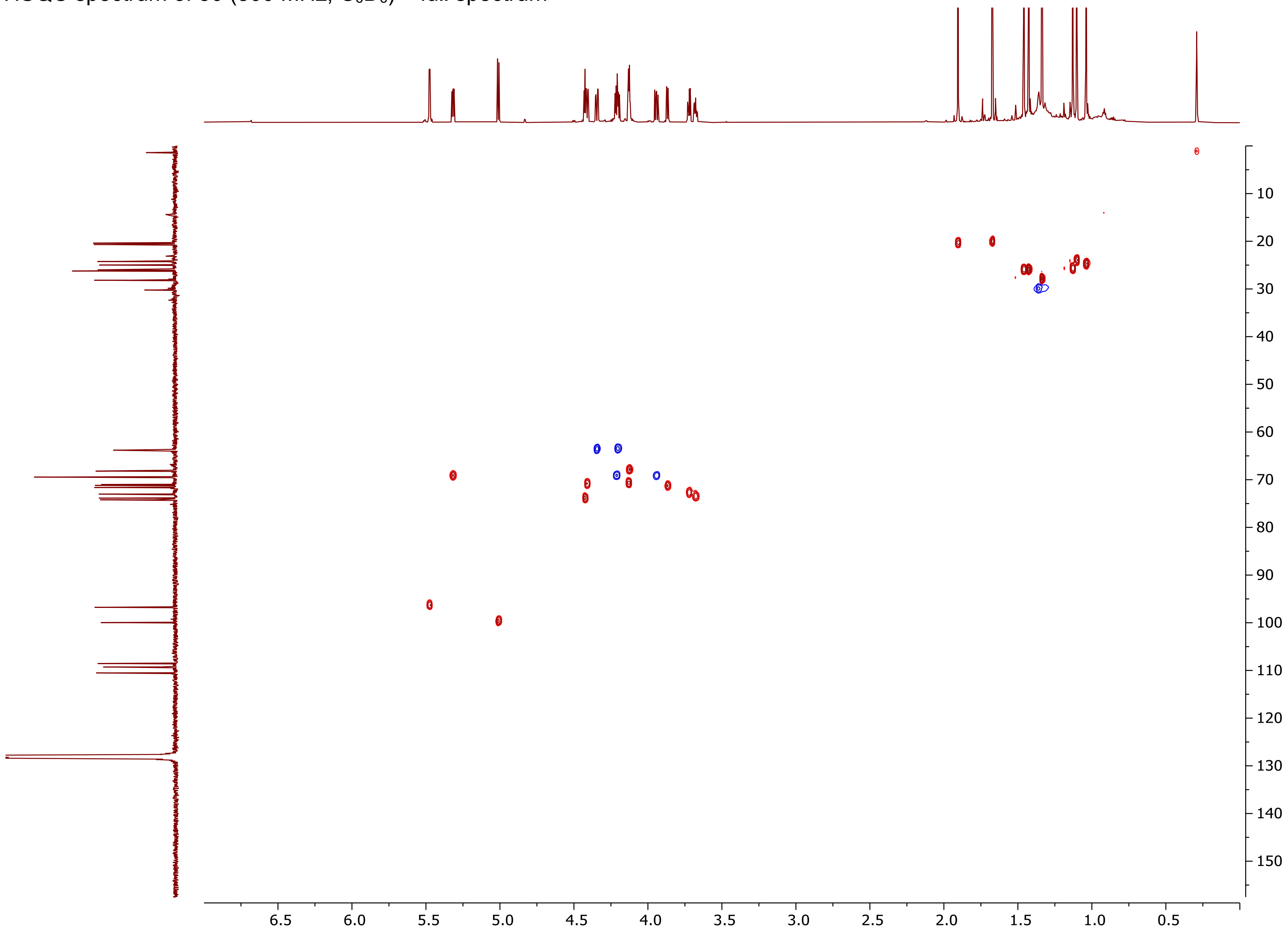

HSQC spectrum of **30** (800 MHz, C_6D_6) – expansion

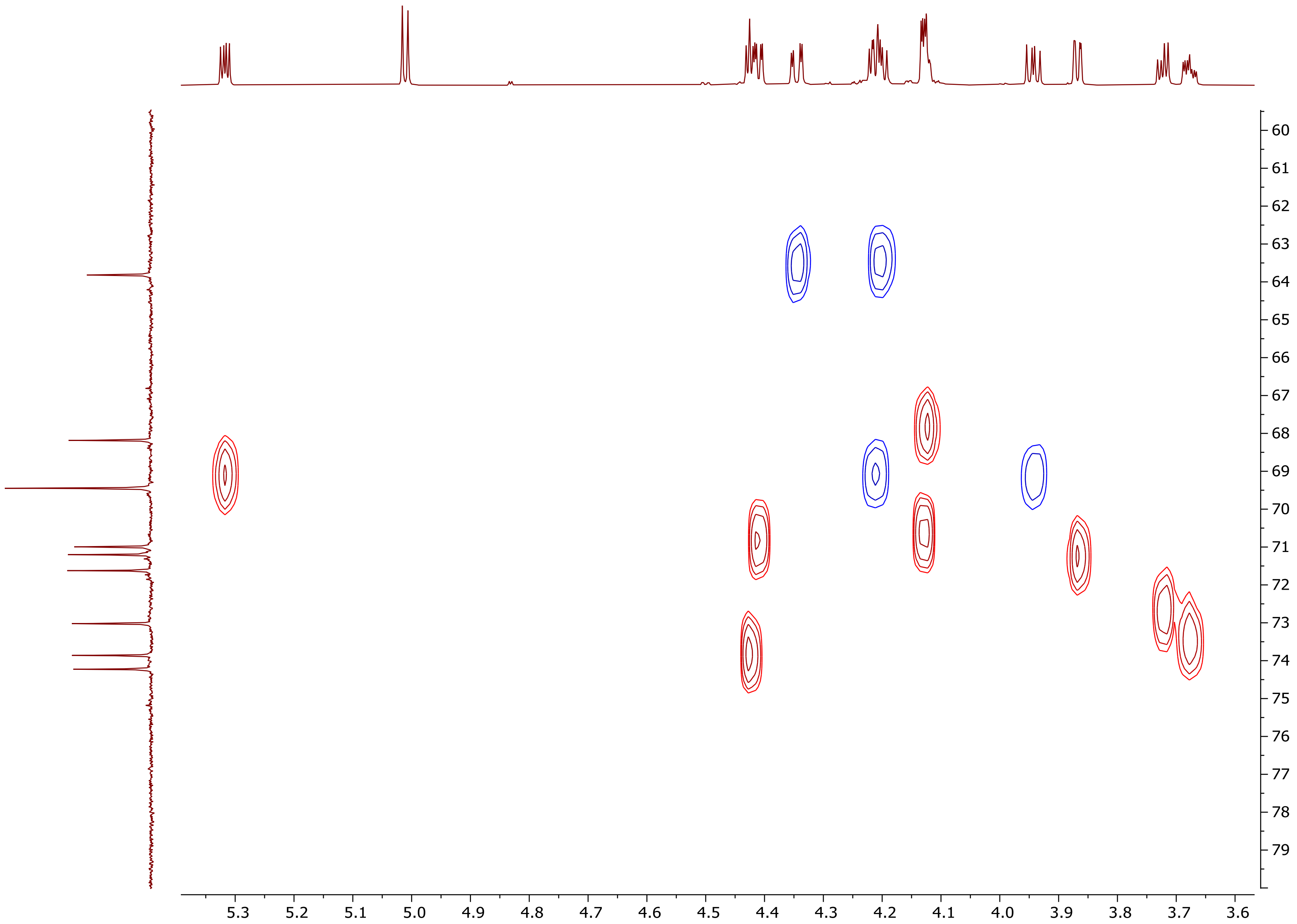

HMBC spectrum of **30** (800 MHz, C_6D_6) – full spectrum

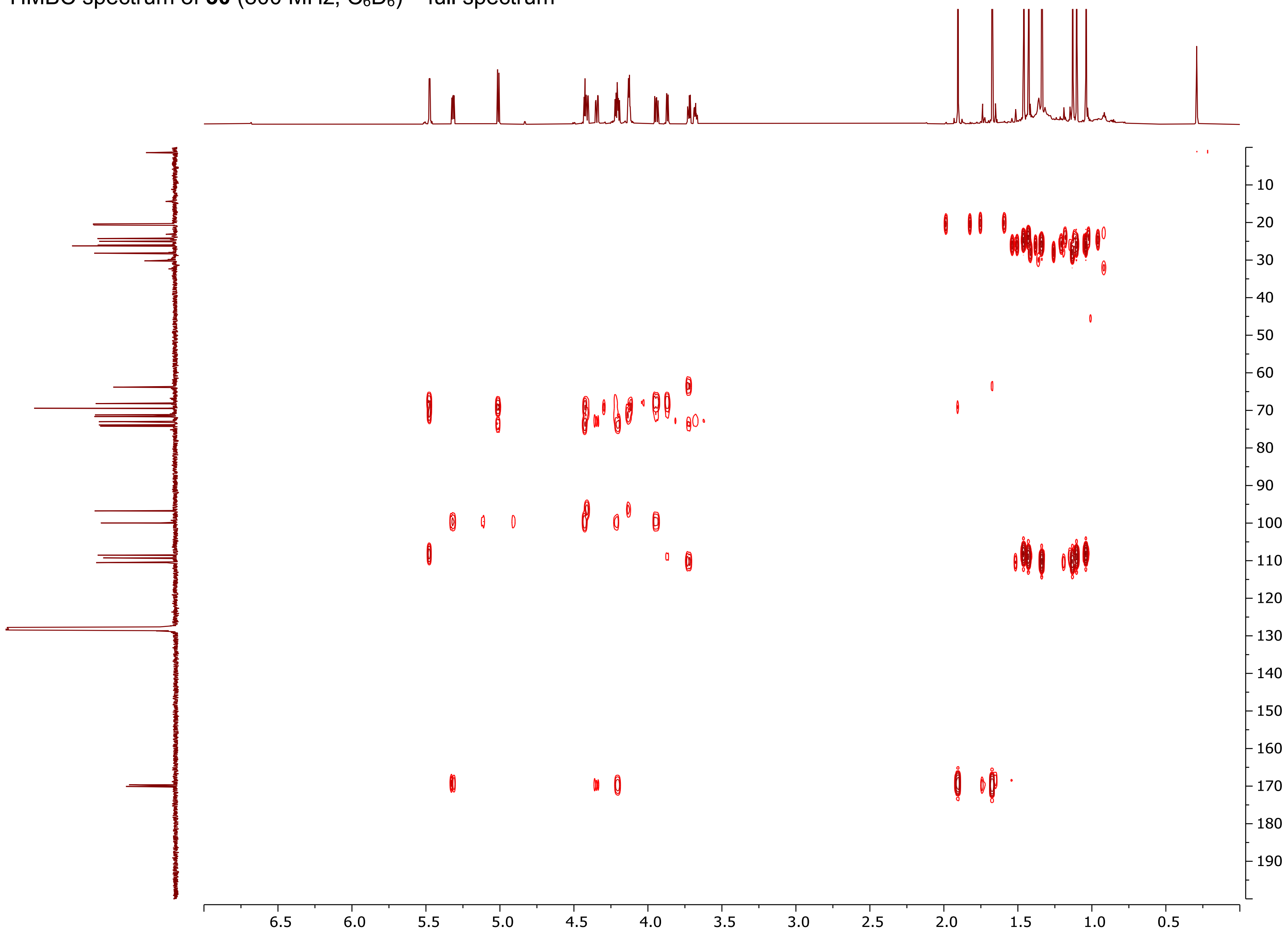

HMBC spectrum of **30** (800 MHz, C_6D_6) – expansion

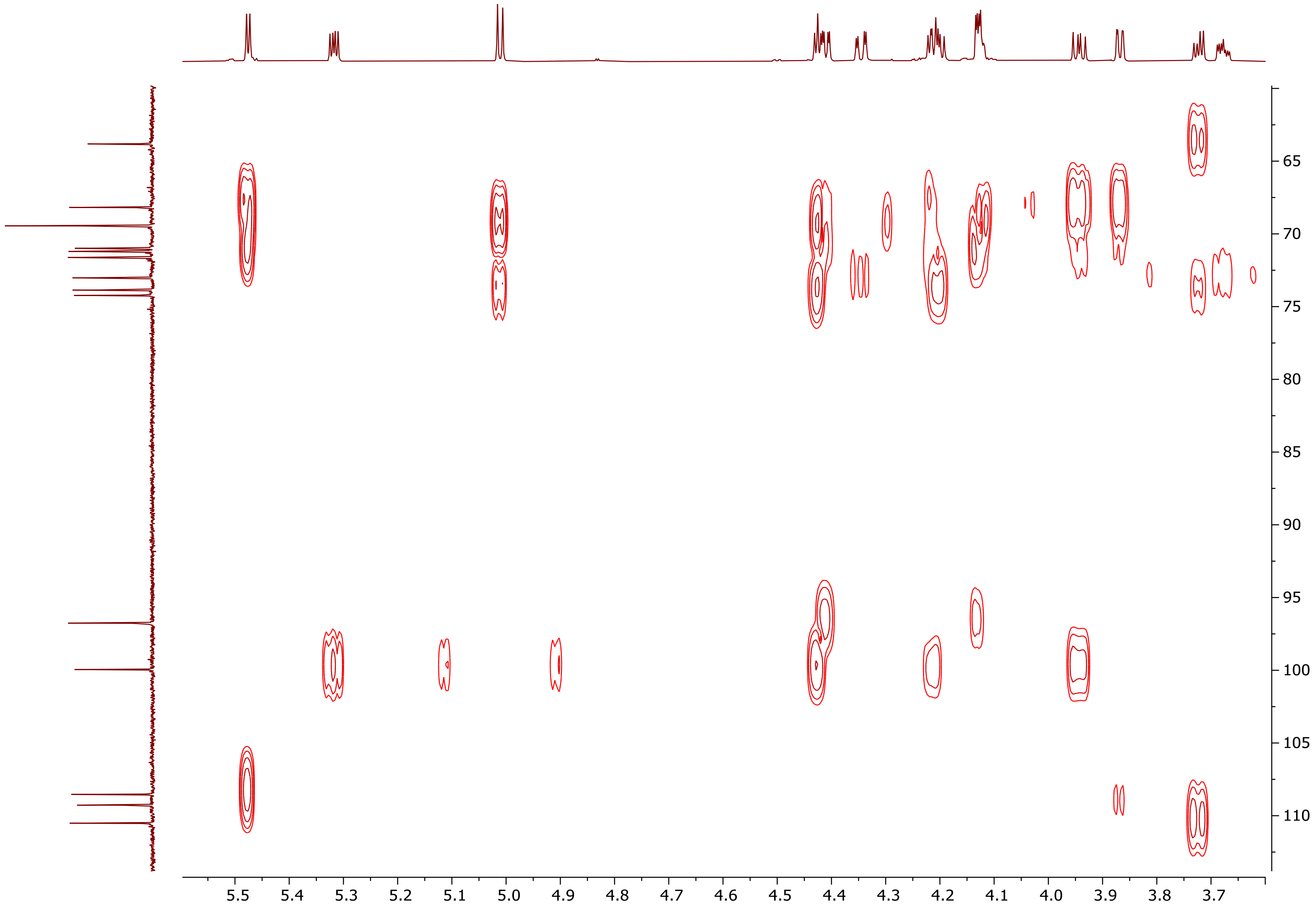

^{1}H NMR Spectrum of **31** (800 MHz, C_6D_6) – full spectrum

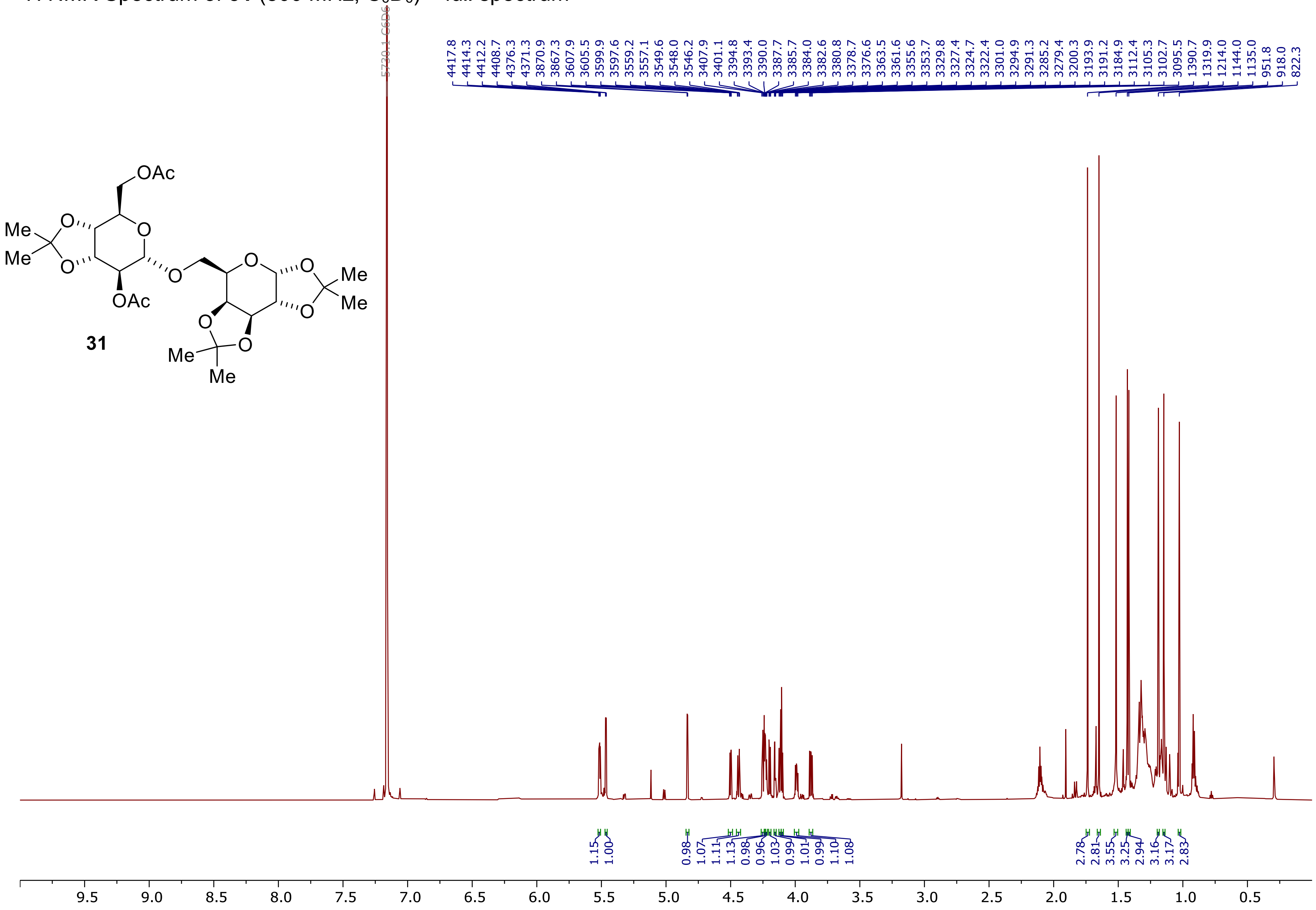

^{1}H NMR Spectrum of **31** (800 MHz, C_6D_6) – expansion

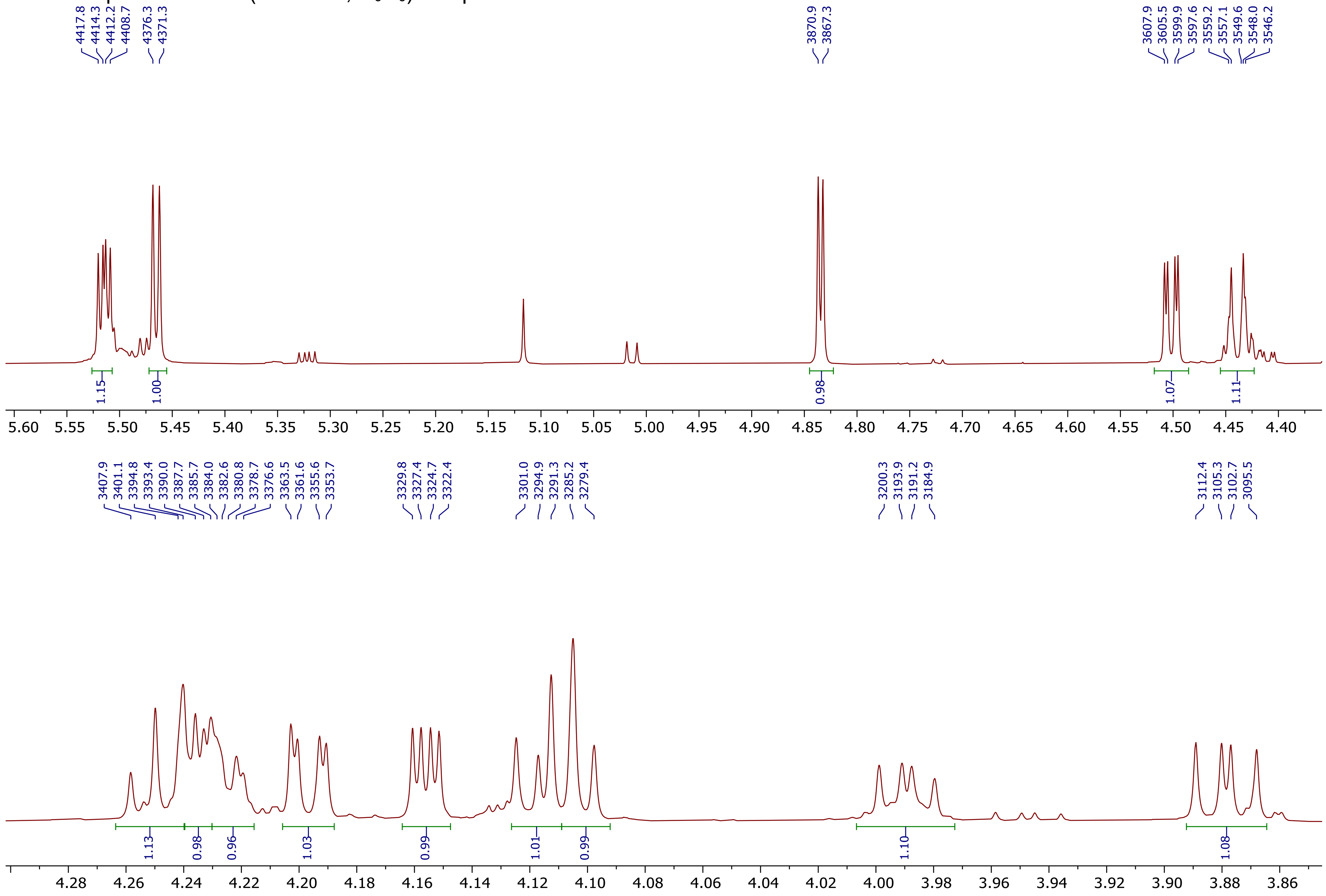

$^{13}C\{^{1}H\}$ NMR Spectrum of **31** (101 MHz, C_6D_6)

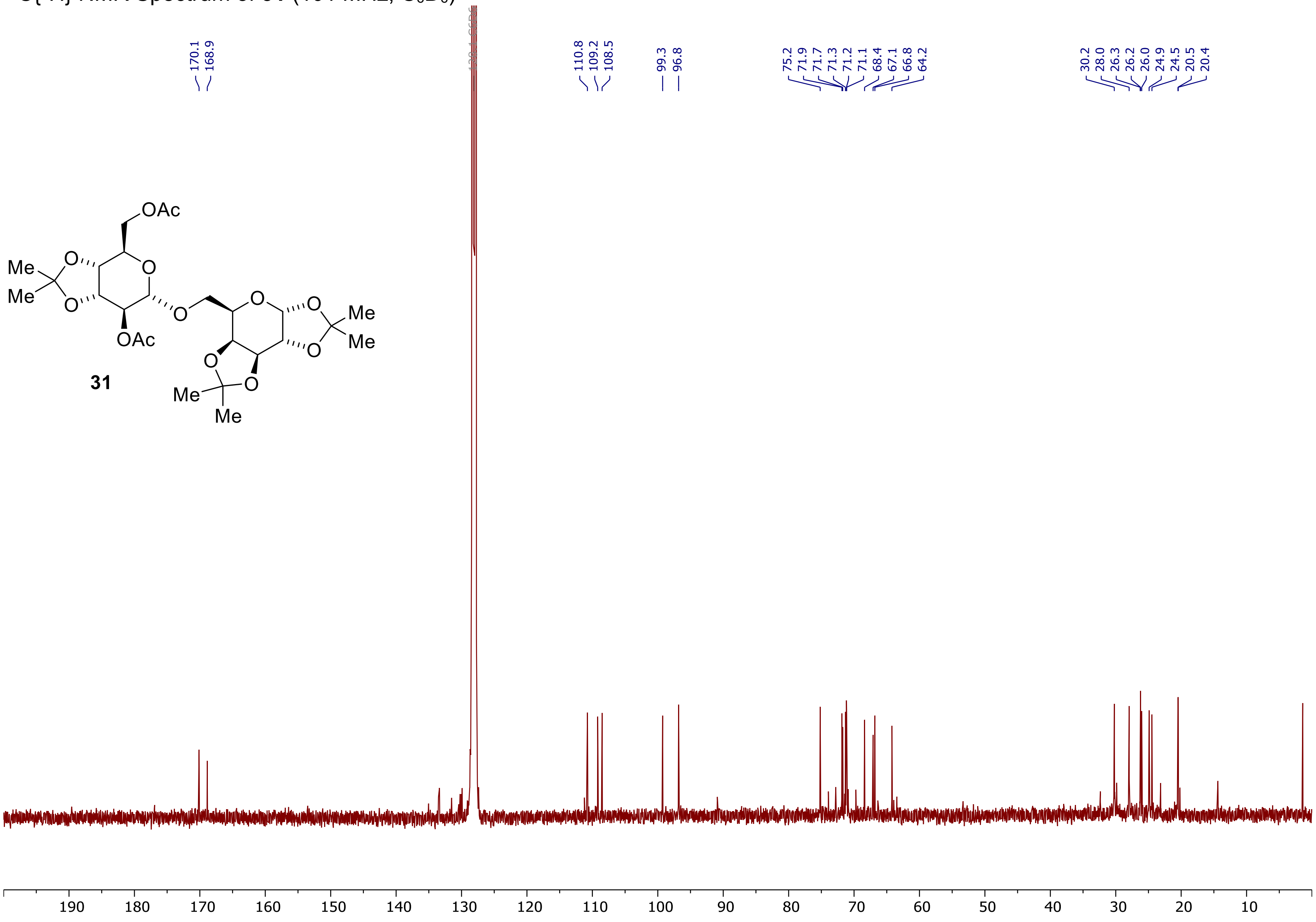

COSY spectrum of **31** (800 MHz, C_6D_6) – expansion

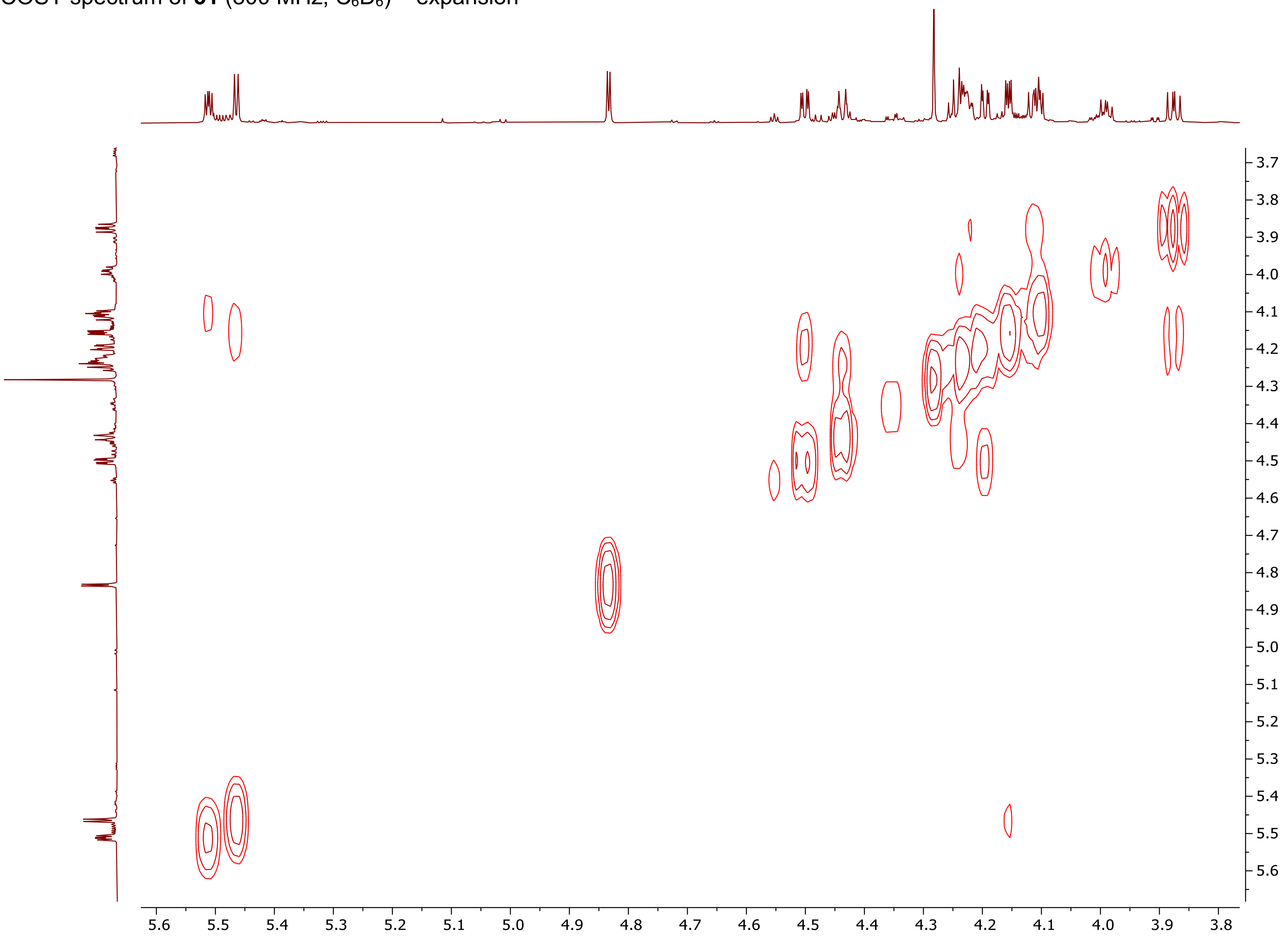

HSQC spectrum of **31** (800 MHz, C_6D_6) – expansion

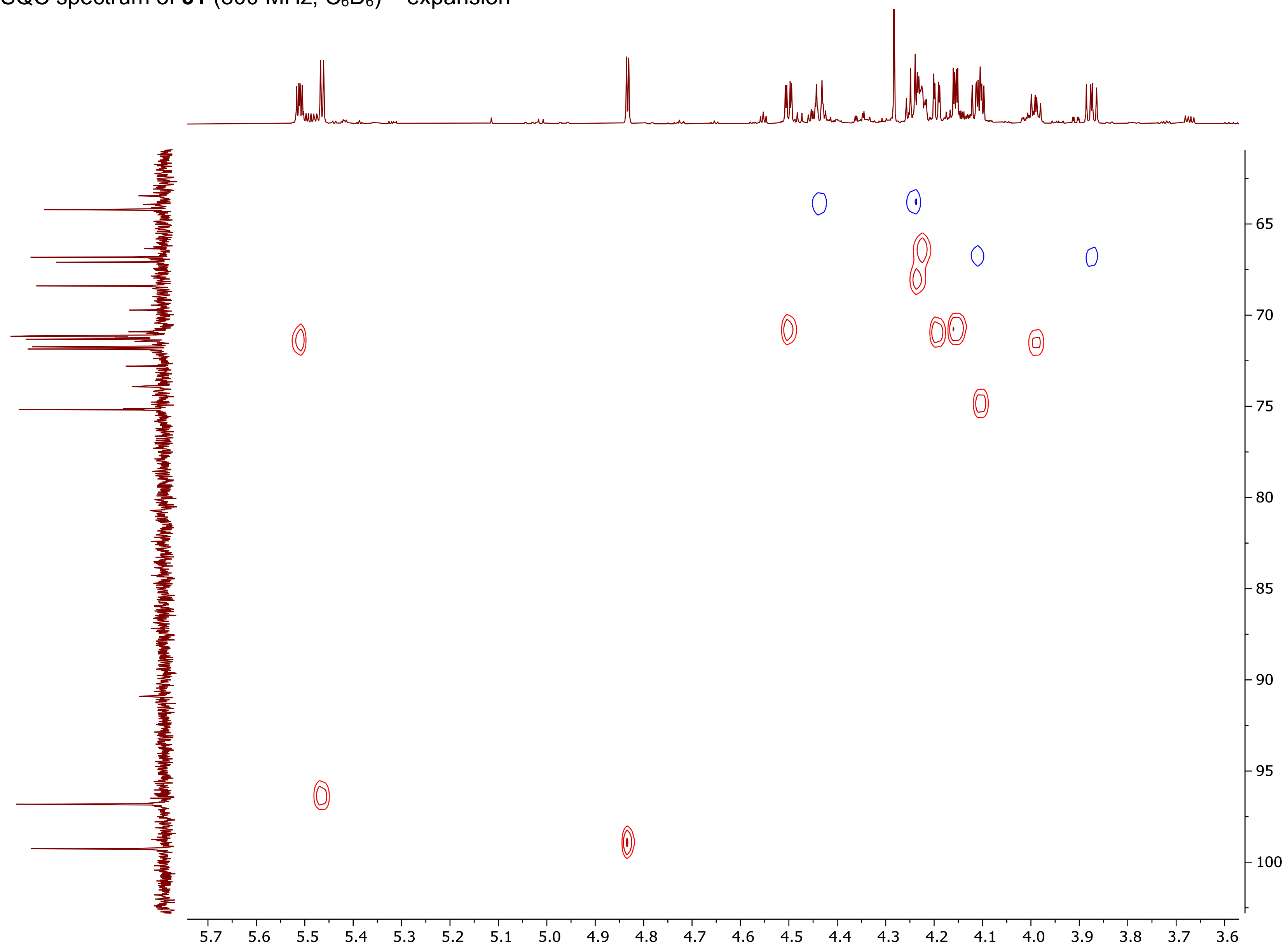

HMBC spectrum of **31** (800 MHz, C_6D_6) – expansion

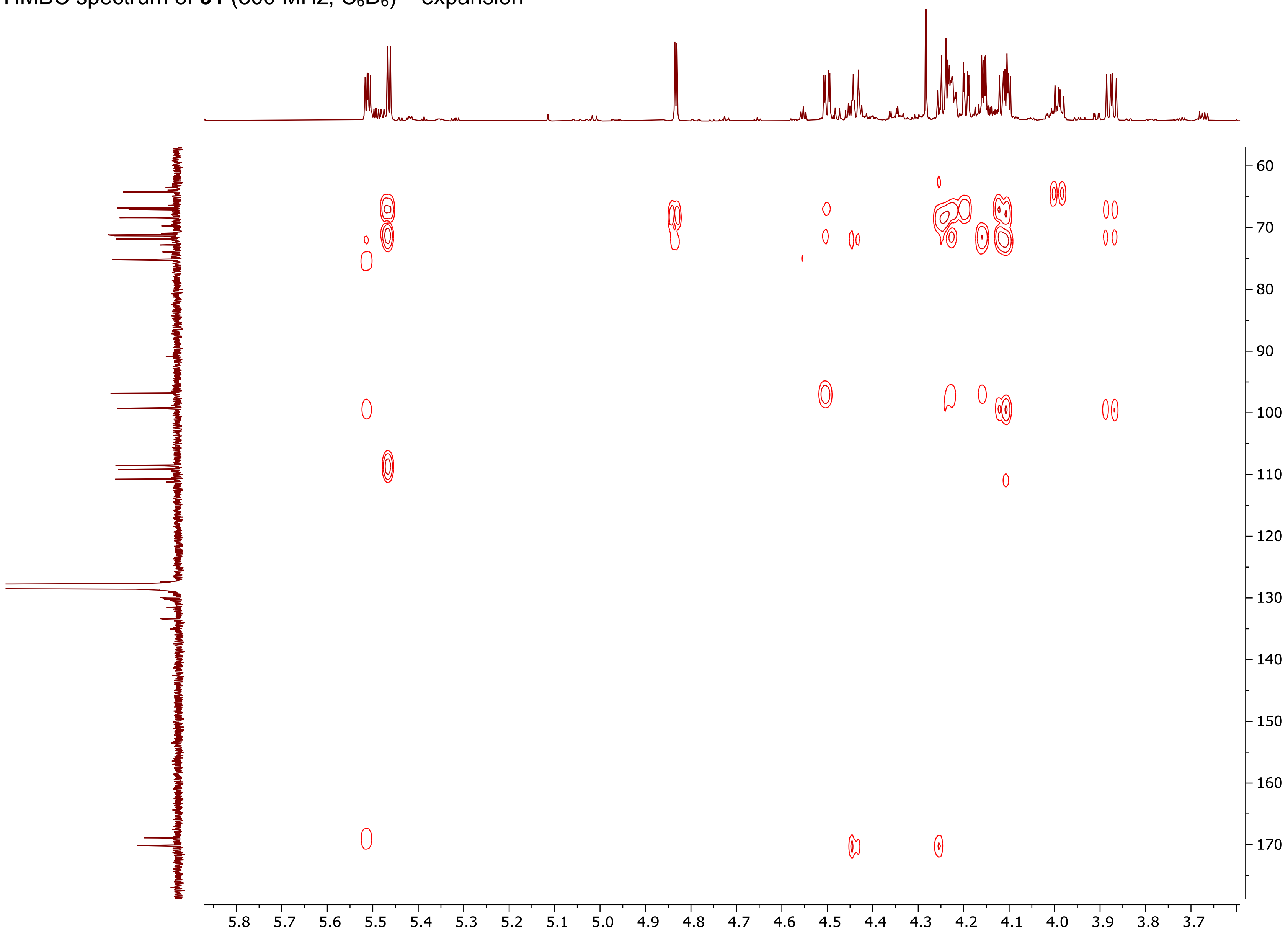

Supplement: Supplementary file 2 — jo4c02176_si_002.pdf [file jo4c02176_si_002.pdf]
